# Coordination diversity in hydrogen-bonded homoleptic fluoride–alcohol complexes modulates reactivity†
†Electronic supplementary information (ESI) available: Detailed procedures for the synthesis of fluoride–alcohol complexes, ^1^H and ^13^C NMR spectra, crystal structures and reaction kinetics. CCDC 1401765–1401778. For ESI and crystallographic data in CIF or other electronic format see DOI: 10.1039/c5sc01812a



**DOI:** 10.1039/c5sc01812a

**Published:** 2015-06-22

**Authors:** Keary M. Engle, Lukas Pfeifer, George W. Pidgeon, Guy T. Giuffredi, Amber L. Thompson, Robert S. Paton, John M. Brown, Véronique Gouverneur

**Affiliations:** a Chemistry Research Laboratory , Department of Chemistry , Oxford University , OX1 3TA , UK . Email: veronique.gouverneur@chem.ox.ac.uk ; Fax: +44 (0)1865 285002

## Abstract

The nucleophilic reactivity of fluoride ion is altered in the presence of hydrogen-bond donors, including alcohols. Relatively little is known about the coordination involved; to rectify this, the X-ray structures of fourteen novel fluoride–alcohol complexes with tetrabutylammonium as the counterion have been determined. The coordination number varies from two to four depending on the steric bulk of the alcohol and is closely linked to trends in reactivity. This diversity in coordination stoichiometry is unprecedented but significant, as it implies differences in the ability of the fluoride–alcohol complexes to dissociate in solution with release of a more active and/or selective fluoride source.

## Abstract 2

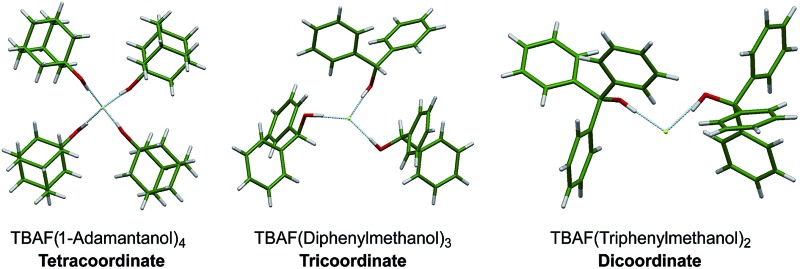
The X-ray structures of fourteen novel fluoride–alcohol complexes with tetrabutylammonium as the counterion show coordination diversity varying from four to two depending on the steric bulk of the alcohol.

## Introduction

**Fig. 1:**
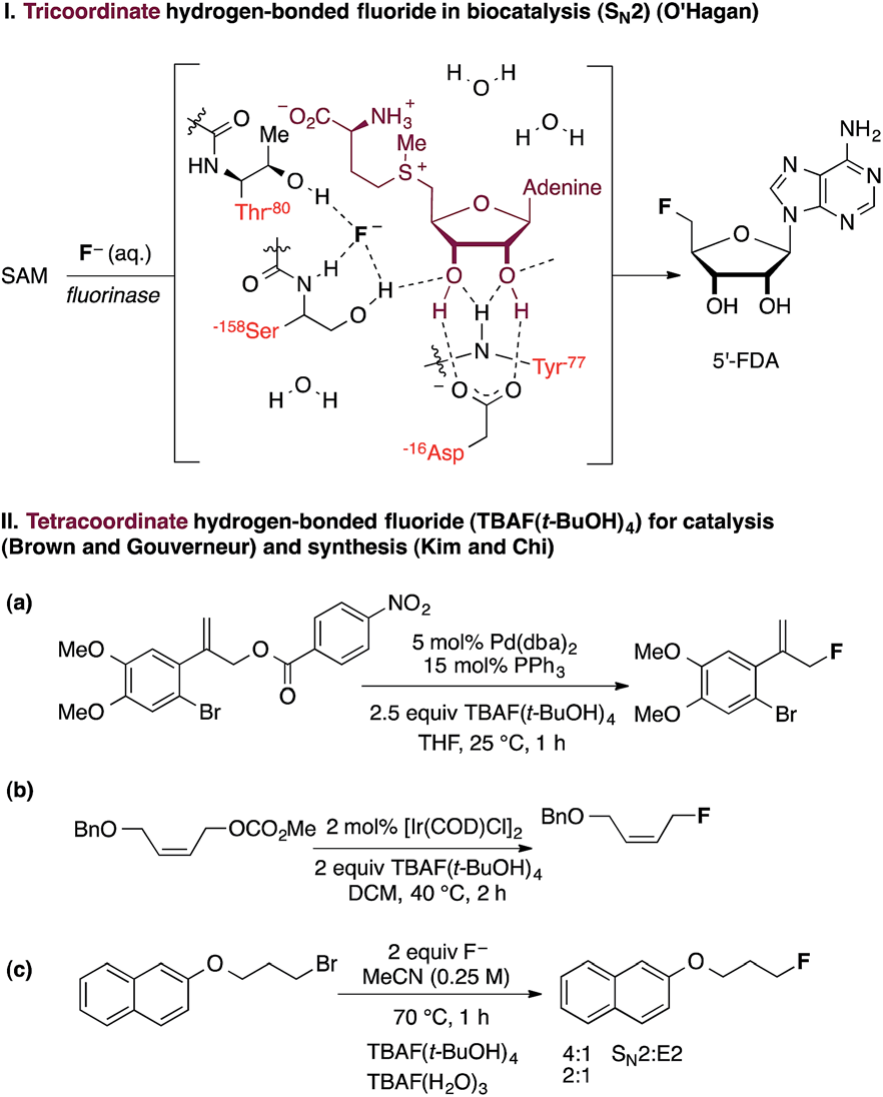
Hydrogen bonding and fluoride reactivity.

Atom-economical fluorination processes are highly sought-after, especially those using readily available starting materials and inexpensive fluoride sources. The renewed interest in “F^–^” chemistry has also been driven by the global growth of the radiopharmaceutical industry and the increasing demand for ^18^F-fluoride based radiochemistry for applications in positron emission tomography.^1^ The fluoride salt fluorite (also called fluorspar) is an important industrial chemical for the production of hydrogen fluoride, a precursor of many fluorine-containing fine pharmaceuticals.^2^ In research laboratories, inexpensive anionic fluoride sources are increasingly used as an alternative to F^+^ reagents for transition metal-catalyzed reactions leading to C(sp^2^)–F and C(sp^3^)–F bond construction.^3^ Examples of metal-free catalytic nucleophilic fluorinations with fluoride are rare. A remarkable exception is the native fluorinase enzyme, with its ability to produce 5′-fluoro-5′-deoxyadenosine from fluoride in aqueous medium (Fig. 1(I)).^4^ This unique enzyme increases fluoride nucleophilicity within the active site through desolvation for substitution at the preactivated C-center of *S*-adenosylmethionine (SAM). Single-crystal X-ray diffraction studies of substrate- and product-bound structures revealed that fluoride forms two hydrogen bonds to Ser-158 when it binds in the active site.^5^ Subsequent substrate (SAM) binding encourages fluoride ion dehydration, thereby facilitating nucleophilic fluorination. An additional hydrogen-bonding interaction of fluoride with Thr-80 likely stabilizes the transition state of the S_N_2 fluorination process. This enzymatic fluorination reaction is highly significant because the use of fluoride ion for C–F bond formation is not trivial and is often met with complications. One challenge is the poor solubility of common fluoride salts in organic solvent. Moreover, fluoride is strongly basic in its unsolvated form, and solvation through hydrogen bonding typically lowers nucleophilicity. Numerous strategies have been considered to augment the scope of fluoride-based chemistry, either by diversifying the range of fluoride ion sources or by achieving controlled fluoride release in solution from neutral reagents. Our own contribution to catalytic nucleophilic fluorination processes established that the use of TBAF(*t*-BuOH)_4_ is critically important in Pd- and Ir-catalyzed fluorination of allylic *p*-nitrobenzoates and carbonates.^6,7^ This reagent is by far the most suitable fluoride source for these reactions; neither ammonium fluoride nor a range of inorganic alkali fluorides led to effective product formation (Fig. 1(IIa) and 1(IIb)).

These findings raise the question of how hydrogen bonding to fluoride influences reactivity. Such a study may facilitate the development of superior F^–^ reagents by design, and inform the development of a biomimetic fluorinase catalyst capable of broad substrate tolerance, with no compromise on efficacy. The ability of fluoride to engage in hydrogen bonding has been previously evoked as a parameter that influences fluoride reactivity,^8^ but no detailed analysis is available on how the coordination sphere of hydrogen-bonded fluoride complexes correlates with reactivity and product distribution (S_N_2/E2 selectivity). In 1994, the first study examining the effect of hydrogen bonding on fluoride reactivity was disclosed by Yonezawa and co-workers, who prepared a series of hydrogen-bonded TBAF complexes from TBAF(H_2_O)_3_ using alcohol solvents as hydrogen bond donors.^9^ A study of their reactivity in a model S_N_2 reaction with benzyl bromide revealed that the reaction rate was positively correlated with the steric bulk of the alcohol (*t*-BuOH ≫ i-PrOH > *n*-BuOH ∼ *n*-PrOH > H_2_O). Kim and co-workers subsequently reported that kinetic reactivity and S_N_2 *versus* E2 selectivity were enhanced when CsF was used in the presence of bulky tertiary alcohols (*e.g.*, *t*-BuOH, *t*-AmylOH and 3-methyl-3-pentanol) (Fig. 1(IIc)).^10^ In 2008, the same group published the isolation, characterization and X-ray structure of TBAF(*t*-BuOH)_4_, confirming its solid-state coordination stoichiometry and tetrahedral geometry.^11^ These preliminary data encouraged us to further study hydrogen-bonding interactions with fluoride as tool to rationally tune reactivity. This problem is of fundamental interest, particularly given that Nature has evolved a fluorinase enzyme capable of partially desolvating fluoride through hydrogen bonding to key residues at the active site to improve nucleophilicity, as discussed earlier. The importance of hydrogen bonding to fluoride extends to transformations other than C–F bond formation, including catalytic, stereoselective desilylation. Selected examples include the catalytic kinetic resolution of silyl-protected secondary benzylic alcohols using chiral hydroxyl-terminated polyether catalysts with potassium fluoride^12^ and the asymmetric acylation of silyl ketene acetals performed in the presence of a dual-function chiral thiourea organocatalyst.^13^ These processes are proposed to involve complexes in which fluoride is hydrogen-bonded to the polyether or thiourea catalyst.

The paucity of structural data on fluoride–alcohol complexes prompted us to examine in detail the coordination chemistry of fluoride–alcohol complexes with the aim of determining how structure correlates with reactivity. Complex formation between halide anions and alcohols has been investigated by gas-phase experimental methods^14^ and computational techniques.^15^ Compared to other halides, the fluoride ion stands out with the largest bonding enthalpies and the shortest X···H hydrogen-bond lengths with various hydrogen-bond donors. Detailed information on the structural properties in the solid state for complexes of fluoride with alcohols other than phenol derivatives and *t*-BuOH is surprisingly lacking.^16^ Herein, we disclose the synthesis of fourteen new fluoride–alcohol complexes and their full characterization in the solid state using single-crystal X-ray diffraction. We also present data on the relative reactivity of these fluoride ion complexes towards a model reactant, demonstrating their potential as useful fluoride reagents in organic synthesis. Many of these new complexes are easy to handle solids that are less hygroscopic than TBAF(H_2_O)_3_ and TBAF(*t*-BuOH)_4_.

## Results and discussion

### Overview

The alcohols in this study were chosen on the basis of varying steric bulk, in order to elicit a range of coordination geometries in the solid state. The complexes were prepared in good yields by adapting an established synthetic protocol; TBAF(H_2_O)_3_ was combined with the alcohol (1–4 eq.) in vigorously refluxing hexane for 2 h. The ensuing crude solid materials were characterized by ^1^H and ^13^C NMR, and recrystallized as appropriate to obtain single crystals suitable for X-ray diffraction studies (*vide infra*). Tetra-alkylammonium fluoride precursors other than TBAF(H_2_O)_3_ (*e.g.*, TMAF and TEAF) were not studied in detail because the resulting alcohol complexes were found to be more difficult to handle and crystallize. All alcohols examined gave either tetra-, tri-, or dicoordinate fluoride–alcohol complexes, with the coordination number decreasing as the degree of branching and steric bulk of the alcohol increased. This variability in coordination stoichiometry had not been observed previously (Table 1).

**Table 1:** Structurally characterized fluoride–alcohol complexes **2a–n**

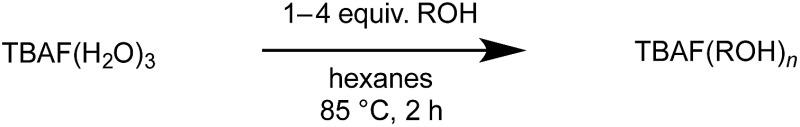				
Entry	Alcohol	Yield	Complex	C.N.^*a*^
1	1-Adamantanol **1a**	84%	**2a**	4
2	Pentaerythritol **1b**	77%	**2b**	4
3	Tris(hydroxymethyl)ethane **1c** ^*b*^	95%	**2c**	4
4	Neopentylglycol **1d**	89%	**2d**	4
5	(*R*,*R*)-di-(i-Pr)-tartrate **1e**	61%	**2e**	4
6	Mannitol derivative **1f** ^*b*^	95%	**2f**	4
7	Pinacol **1g**	93%	**2g**	4
8	(*R*)-BINOL **1h**	88%	**2h**	3
9	Cyclic hemiacetal **1i** ^*b*^	89%	**2i**	3^*c*^
10	9-Phenylfluoren-9-ol **1j**	91%	**2j**	2, 3
11	Diphenylmethanol **1k**	91%	**2k**	3
12	Triphenylmethanol **1l**	61%	**2l**	2
13	Tri-(*p*-tolyl)-methanol **1m**	76%	**2m**	2
14	Pyrrolidine **1n** ^*b*^	77%	**2n**	2^*d*^
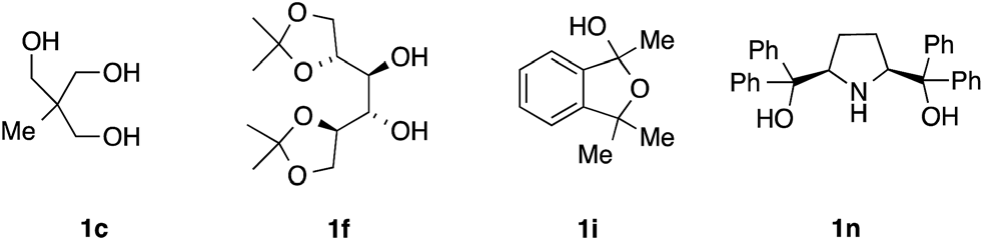				

^*a*^Coordination number in (ROH)_*n*_F^–^.

^*b*^See formulae block.

^*c*^3ROH, 1H_2_O.

^*d*^2(ROH)_2_, 1H_2_O.

### Fluoride complexes with four ROH ligands

**Fig. 2:**
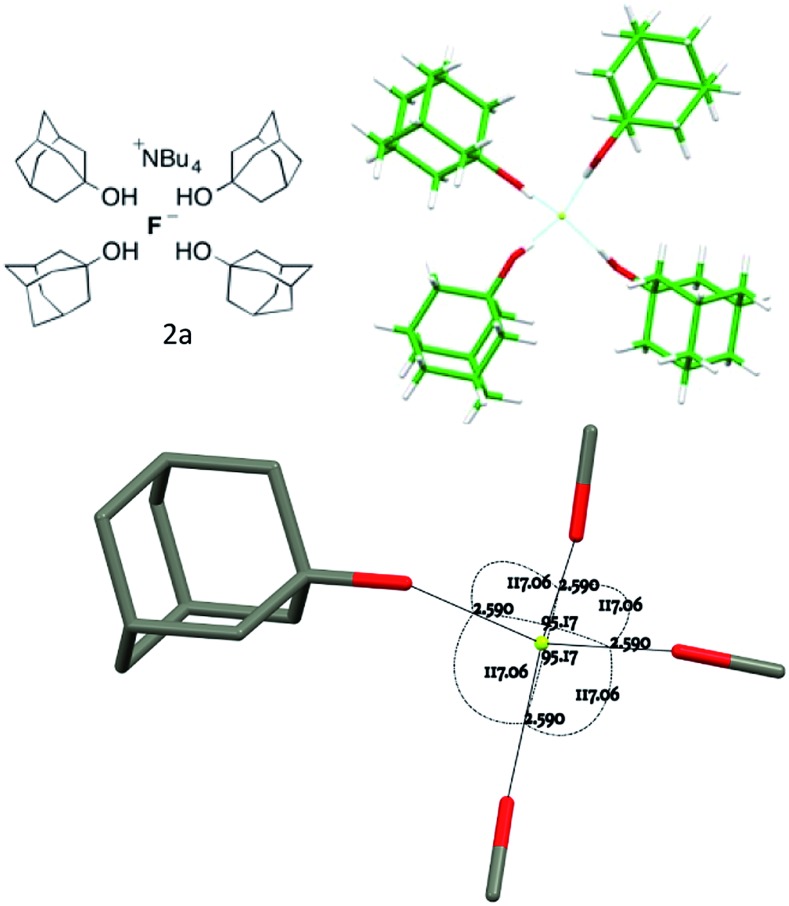
Anion formula and crystal structure together with key geometric parameters for complex **2a**.

The only closely relevant structure preceding this work is that of the tetra-alcohol complex TBAF(*t*-BuOH)_4_.^10^ Accordingly, the tertiary alcohol 1-adamantanol **1a**, which is nearly isosteric with *t*-BuOH around the hydroxyl group but has distinct packing requirements, was examined. Crystallisation of the fluoride complex **2a** and X-ray diffraction (see Experimental section) gave the anion structure shown in Fig. 2. Key geometric parameters describing the environment of one of the two closely similar but crystallographically inequivalent fluorides in the unit cell are given. The other symmetrically equivalent fluoride possesses F···O distances of 2.680(3) Å and O···F···O angles of 95.17(8)° and 117.06(5)°. These structures are in close accord with the single known homoleptic alcohol complex TBAF(*t*-BuOH)_4_ (O···F distance: 2.643(7) Å, O···F···O angles: 97.98(18)° and 115.50(18)°) reported by Kim and co-workers,^9,10^ despite the difference in steric bulk and hydrophobicity of the alcohol hydrogen-bond donors in the two cases. Structures in this class may be analysed for deviations from a formal tetrahedral structure and *T*_d_ symmetry.^17^ Within the coordination sphere, four F···O distances and six O···F···O angles can be measured, as in Fig. 2. For an individual complex, the O···F distances generally do not vary widely and pairs of chelating diols in a 2 : 1 complex exhibit similar bite angles that constrain two of the six angle parameters.

**Fig. 3:**
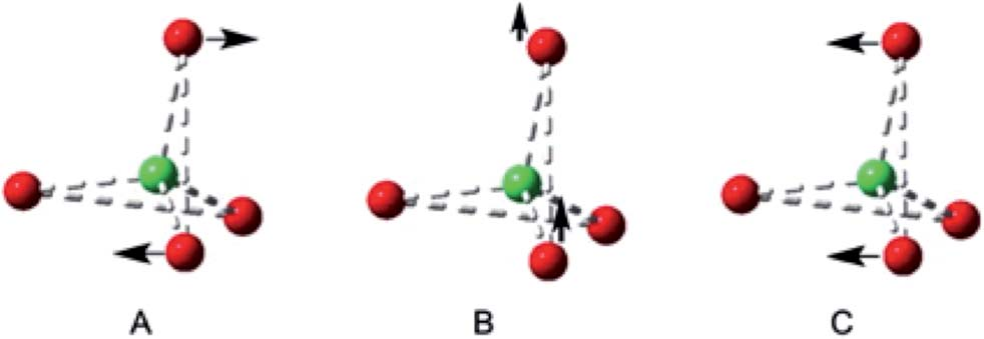
Distortions from *T*_4_ symmetry in a bis-chelated complex with comparable distances in ligation; **A** *C*_2_ twist, **B** Out-of-plane roll, **C** In-plane glide.

The tetra-(1-adamantanol) complex **2a** is the only tetracoordinate structure in this study that does not involve a chelating diol. Consider more generally the three possible coupled-pair distortions from a pure *T*_d_ structure shown in Fig. 3, which represents a structure for which two angle parameters are constrained by bis-chelation and all distances from the central atom are equal. Any possible geometry may be realised by a combination of three movements of one pair, keeping the second stationary. These three modes can be identified as twist **A**, roll **B** and glide **C**. Using this analysis, the two independent molecules in the unit cell of complex **2a** may both be fully described by simply imposing a slight *C*_2_ distortion **A** on the *T*_d_ model so that two of the O···F···O angles become smaller than the remaining four.^18^

**Fig. 4:**
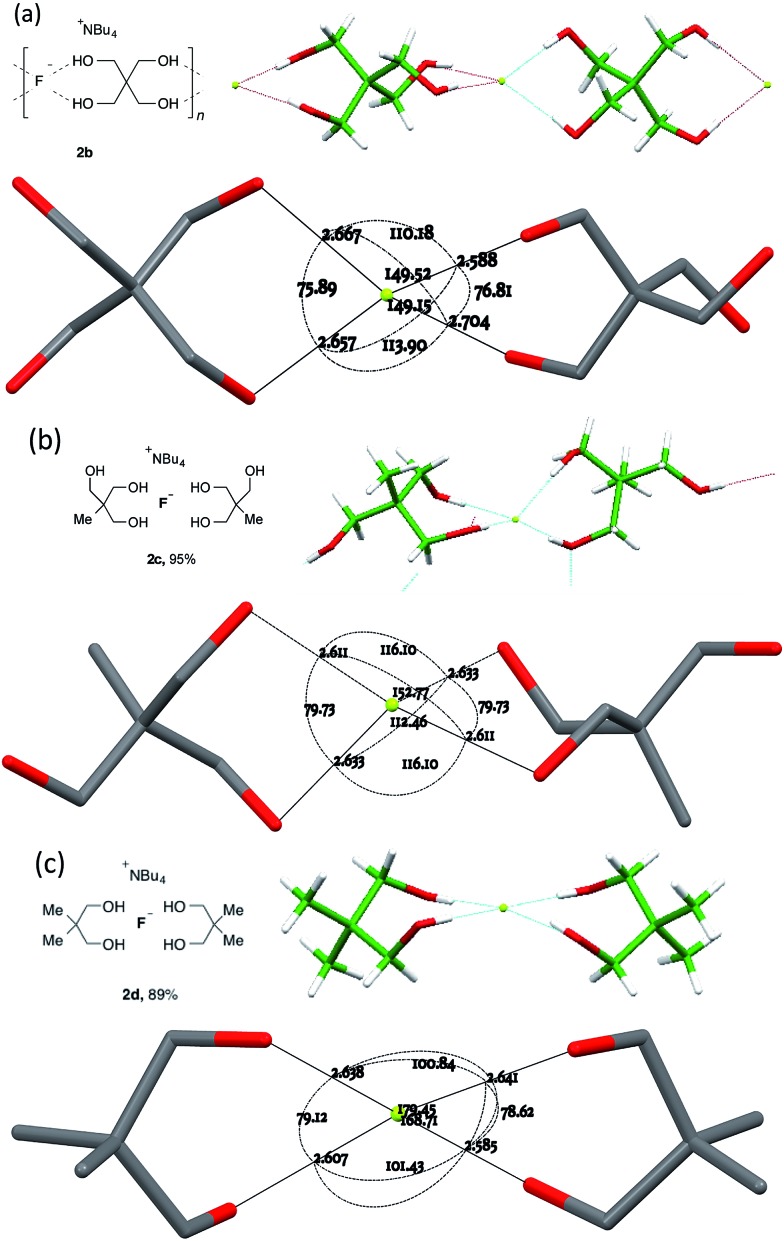
Anion formulae and crystal structures, together with key geometric parameters for the O···F···O cores of **2b** in (a), **2c** in (b) and **2d** in (c).

For chelating diol-based structures, the results are more diverse (Fig. 4).

The three 1,3-diol based structures that were obtained form a closely related set in molecular terms but exhibit distinct coordination modes. The most clear-cut case is **2b**, derived from pentaerythritol **1b**. This 2 : 1 fluoride complex crystallized as its DCM solvate, forming a one-dimensional linear hydrogen-bonded coordination polymeric structure (Fig. 4a) The individual F(diol)_2_ units of the 1D ribbons experience a simple *C*_2_ twist **A** from an ideal orthogonal geometry, resulting in an interplanar angle between the two O···F···O units of 50.54(12)°.

For the structure of complex **2c**, derived from triol **1c** (Fig. 4b), two hydrogen bonds to fluoride per **1c** molecule were observed, with the third hydroxyl group forming an additional hydrogen bond that activates and shortens the neighbouring O–H···F bond. At 81.32(6)°, the plane between the pairs of O···F···O angles of the chelates is close to that of an undistorted tetrahedron. In effect however, through roll and glide motions **B** and **C**, one of the two donor –OH groups from one diol remains approximately in its tetrahedral position while the other diol has been rotated away, leading to an arrangement where one oxygen is in the O···F···O plane of the first diol ligand.

The third member of the series, **2d** derived from neopentyl glycol **1d**, is again distinct, possessing four different O···F···O distances, as shown in Fig. 4c. The two chelate units are close to coplanarity with an interplanar angle of 11.51(8)° but further modified by a significant contribution of roll distortion **B**.

**Fig. 5:**
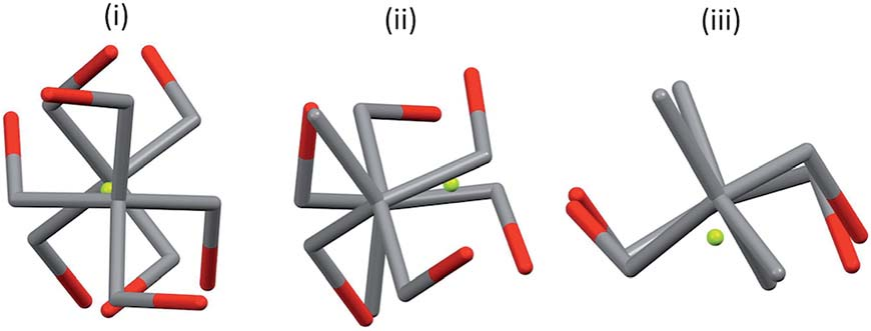
Structures (i) **2b**, (ii) **2c**, (iii) **2d**: view along the axis linking quaternary carbons.

Three ostensibly similar fluoride anion complexes with the same counterion thus show quite distinct geometrical parameters. The variation observed points to a structural model for which the overall lattice geometry is primarily determined by the TBAF cation and the alcohols, with fluoride ion demonstrating a capacity to fit within that structure. The O···F distances, however, vary only to a small extent in any given structure. Fig. 5 illustrates these tendencies for the three structures discussed above. Viewing the structures through an axis between the central C–C bonds emphasises the marked variation in fluoride ion location relative to its ligands.

**Fig. 6:**
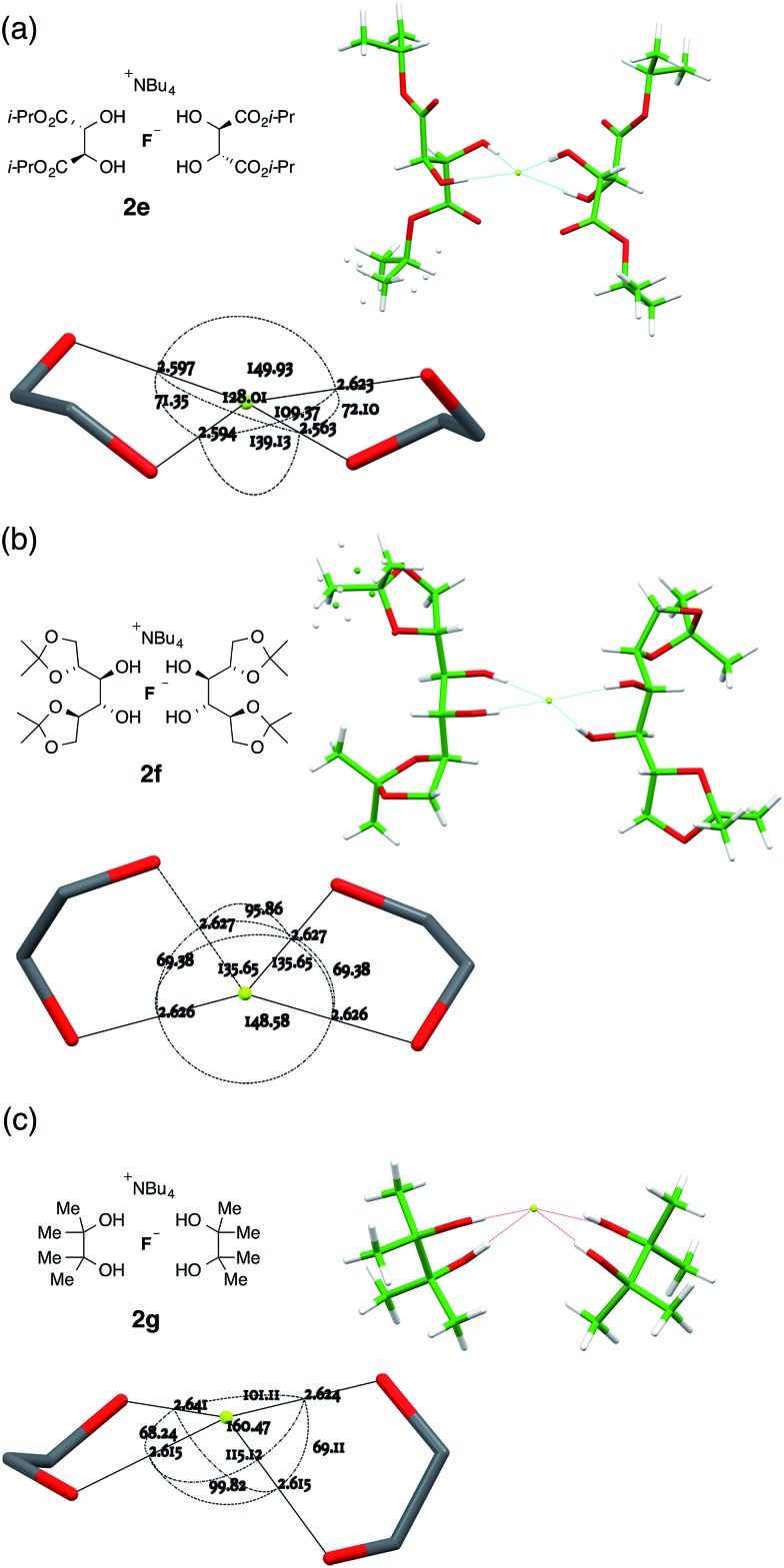
Anion formulae and X-ray structures, together with key geometric parameters for the O···F···O cores of **2e**, **2f** and **2g**.

Three analogous 1,2-diol complexes were prepared, and their crystal structures were analysed similarly. Complex **2e**, derived from the enantiomerically pure hydrogen-bond donor (*R*,*R*)-di-isopropyl tartrate **1e**, was crystallized as a hexane solvate. The anion in the ensuing 2 : 1 complex is *C*_2_-symmetric with the two O···F···O planes oriented at 60.05(7)° to one another. In this geometry the two central C–C bonds are very nearly coplanar, with the F atom close to equidistant from the carbon atoms of these bonds and just 0.1391(11) Å from their mean plane. The basic geometry of the complex is imposed by its overall *C*_2_ symmetry (Fig. 6a).

The related complex **2f**, derived from 1,2,5,6-(*R*,*S*,*S*,*R*)-di-isopropylidene mannitol **1f**, crystallized as an EtOAc solvate. This structure also possesses local *C*_2_ symmetry about the anion, and here the interplanar twist of the two O···F···O subunits is very similar to **2e** at 61.03(5)°. The actual geometry, however, is quite distinct from **2e** through substantial rolling distortion. When one subunit is aligned in plane, the oxygen atoms of the other subunit are respectively 0.7222(8) Å above and 1.7874(9) Å below that plane. The central C–C bonds of the two ligands are no longer co-planar (Fig. 6b).

More significant structural variation was observed in the complex **2g**, derived from pinacol, where two of the four O···F distances are equal to one another and distinct from the remaining two. Here the twist angle between the two O···F···O subunit planes is 64.96(8)°, but all four OH ligands are now clearly confined to one coordination hemisphere (Fig. 6c). With respect to one subunit plane, the oxygen atoms of the second diol ligand are respectively 0.7417(13) Å and 2.3880(13) Å, both below that plane. If the subunit planes are created directly from the hydroxyl H···F···H positions, they still occupy a single hemisphere. In order to pursue this observation further, the corresponding tetraethylammonium and tetramethylammonium complexes were synthesized, but both resisted attempts to prepare X-ray diffraction quality crystals.

### Fluoride complexes with three ROH ligands

**Fig. 7:**
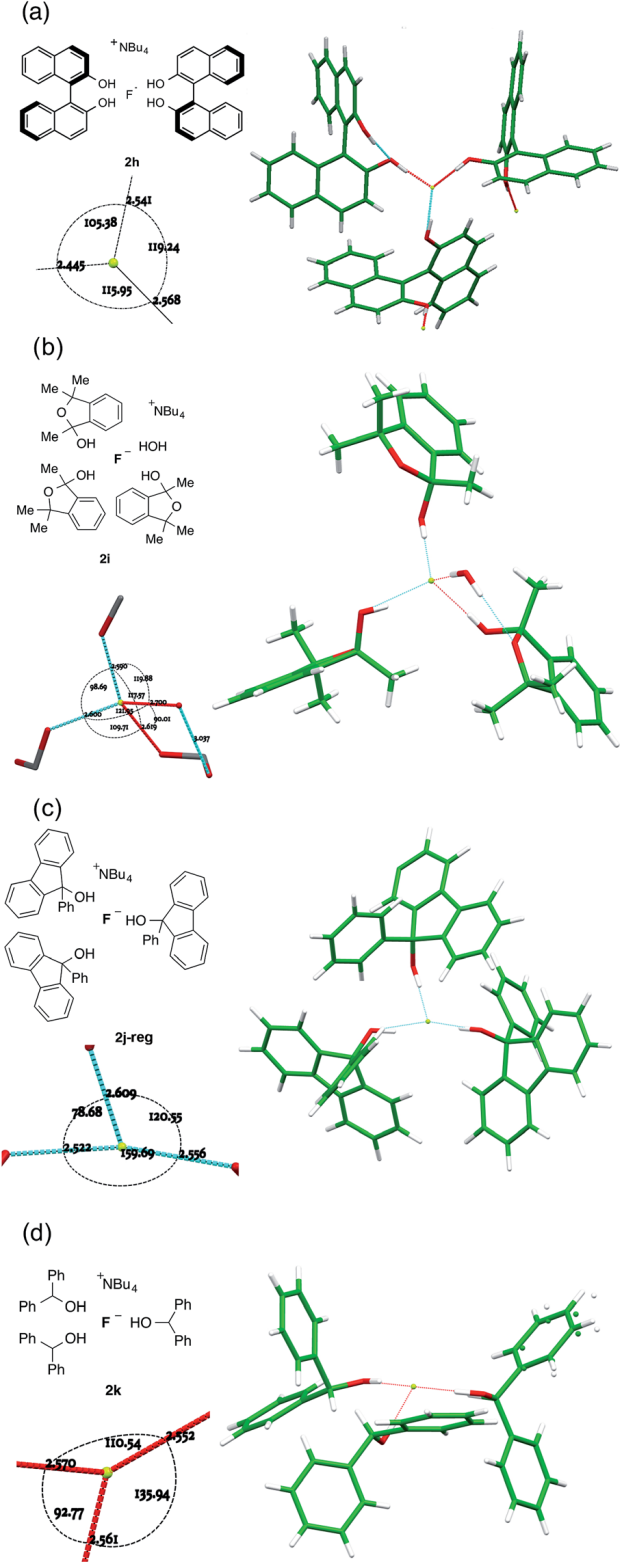
Anion formulae and X-ray structures, together with key geometric parameters for the O···F···O cores of **2h**, **2i**, **2j-reg** and **2k**.

For complexes where the alcohol is sufficiently bulky to permit just three or fewer O–H···F bonds to fluoride, different patterns emerge depending on the alcohol structure (Fig. 7).

There is a tendency towards alternative bonding modes that permit higher coordination numbers.^16*a*^ For the (*R*)-binol 2 : 1 complex **2h** shown in Fig. 7a, three different molecules participate in bonding to a single fluoride ion as part of an extended network linked by interligand hydrogen bonding. This results in a flattened tetrahedral geometry for fluoride with an unoccupied site, where F^–^ is 0.6501(6) Å distant from the plane described by the three alcohol oxygens. There is an *ortho*-aryl C–H close to the fourth apex with a C···F distance of 3.3398(12) Å, but it is not well directed for hydrogen bonding (C–H···F = 125.59(3)°).

Trimethylisobenzofuran-2-ol **1i** forms complex **2i** shown in Fig. 7b. The three-ligand motif is modified here by incorporation of a single ligating water molecule; the resulting structure is close to tetrahedral with all O···F···O angles between 90° and 120° and with fluoride ion 0.8942(8) Å out of the plane of the three oxygen atoms of the donor groups. The hemiacetal is chiral, although the complex crystallizes in an achiral space group. Thus, each individual anion has alternatively (*R*,*R*,*S*) or (*S*,*S*,*R*) configuration. There are two independent motifs in the crystal structure of the 9-phenylfluoren-9-ol 3 : 1 complex **2j**, with respectively three and two donor alcohols per fluoride ion (Fig. 7c). The first **2j-reg** is approximately T-shaped with O···F···O angles of 160.40(4)°, 120.00(4)° and 78.30(3)°, and the fluoride ion is just 0.1325(8) Å out of the plane of the three oxygen donor atoms; the second **2j-alt** is discussed below. The 3 : 1 diphenylmethanol complex **2k** falls into this group, with the three donor oxygens as part of a flattened tetrahedron with the fluoride 0.6483(11) Å out of plane (Fig. 7d). The remaining apex is occupied by an α-C–H bond from the TBA cation, with a C···F distance of 3.3331(18) Å, and a C–H···F angle of 161.92(4)°.

### Fluoride–alcohol complexes with two ROH ligands

**Fig. 8:**
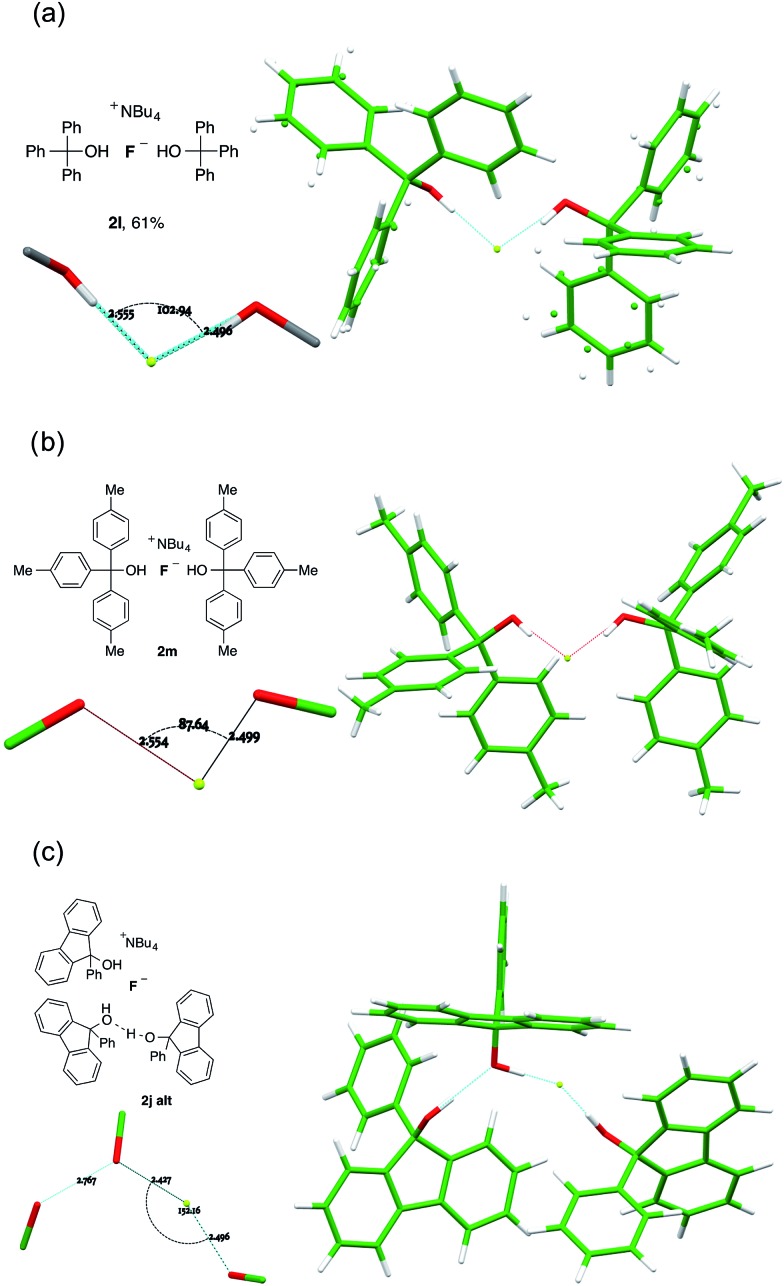
Anion formulae and X-ray structures, together with key geometric parameters for the O···F···O cores of **2l**, **2m**, **2j-alt**.

Dicoordinate complexes of fluoride ion are observed as the sole structural unit only in the bulky triarylmethanol complexes **2l** and **2m**, and as the alternative structural motif found in the unit cell of **2j** (**2j-alt**). In the first of these (**2l**), the O···F···O angle is 102.94(4)°, augmented by donation from an α-C–H of the cation, for which the C···F distance is 3.181(12) Å; the C···F vector makes angles of 117.3(3)° and 133.8(3)° with the two coordinated O-atoms (Fig. 8a). There are further weak hydrogen bonds from two *ortho*-C–H atoms of proximal phenyl groups, where the corresponding C-atoms are 3.247(2) Å and 3.2945(18) Å distant from fluoride and the spatial orientation is favourable.^19^ These two phenyl rings are well ordered whilst the remaining four exhibit librational disorder.

For **2m**, there are two closely related alcohol-complexed anions in the crystal, and both show the same characteristic features as **2l**, with an O···F···O angle of 85.21(3)° and a C···F distance of 3.0983(14) Å from one of the C–H groups α to nitrogen in the first crystallographically distinct equivalent cation (Fig. 8b). These parameters are respectively 87.63(3)° and 3.1207(14) Å in the otherwise similar second anion. This is the least coordinated example in the series and is also the most reactive nucleophile (*vide infra*).

The second structural motif (**2j-alt**) in the unit cell of crystalline **2j** is dicoordinate, with the third molecule of the alcohol involved in hydrogen bonding to one of the donor ligands, but not to fluoride (Fig. 8c). A far wider O···F···O angle is observed, at 151.97(5)°.

**Fig. 9:**
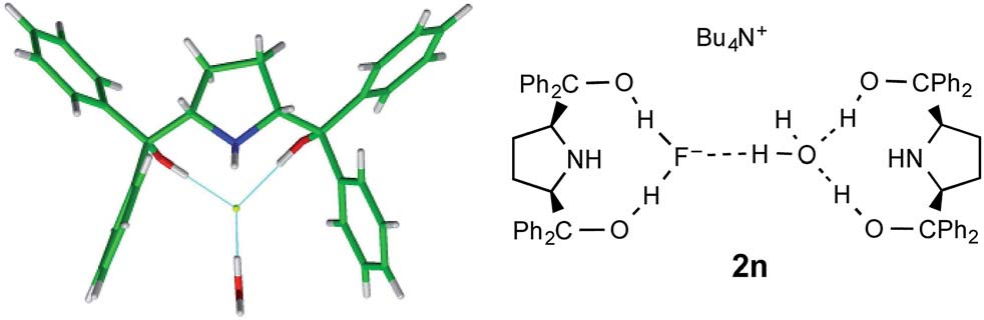
Part of the crystal structure of the 2 : 1 complex anion formed from **1n** and the environment of fluoride ion therein.

One further dicoordinate alcohol fluoride complex was characterized and provides a distinct category. Unlike the diol complexes discussed above, diol **1n** forms crystals of a monohydrated anion, with the water molecule acting as an H-bond acceptor to both hydroxyl groups of a second diol. The secondary amine is not engaged in hydrogen bonding. The geometry of this second diol is almost identical to the first, such that the assembly refines as a single unit with very similar locations for the water oxygen and the fluoride atoms; the two diol ligands are distinct only in the positioning of one phenyl group. Fig. 9 shows the fluoride anion location in this complex.

In general, the coordination number of fluoride complexes is largely determined by the steric bulk of the ligand but was never less than 2 in the series covered in this paper. In accord with the characterized crystal structures of hydrated fluoride ion,^20^ an optimum coordination of 4 hydrogen-bonding ligands is observed here. Computational studies suggest that water association up to hexacoordination is feasible.^21^ In the two published examples where the hydrated fluoride ion is unconstrained by further complexation, the structure of the complexed anion lies between tetrahedral and square planar so that only the twist distortion **A** from the *T*_4_ structure is involved; the O···F···O interplanar angles in those structures are respectively 35° and 37°.

**Fig. 10:**
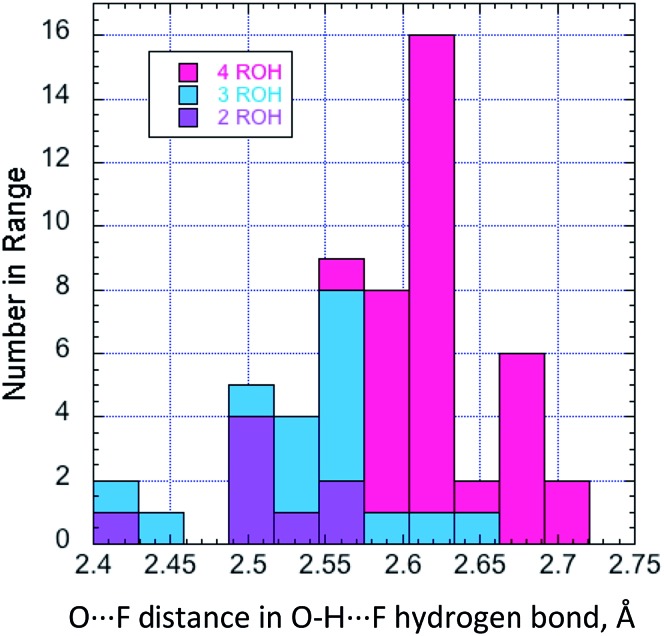
The relationship between O···F distance in hydrogen-bonding alcohols (all examples described here), and the ROH coordination at F^–^.

The larger ligands involved in the present study elicit a far wider structural range. Whilst O···F, and by implication H···F, distances are similar for a given structure, there is a trend towards significantly shorter values with lower coordination numbers, illustrated in Fig. 10.

With 1,2- and 1,3-diols, the interligand angles vary widely, indicating that the geometry of the coordination sphere is far more strongly influenced by packing forces than through any predisposition to an ideal tetrahedral geometry. With low coordination numbers, there is a tendency for fluoride in these complexes to form weak C–H···F bonds.^22^ This is unambiguous for coordination of one or more α-protons of TBAF in three cases; with **2l**, **2m**, and **2k**, the C–H···F angle is 160–166° and the C–F distance is between 3.10 and 3.33 Å. Other interactions involving proximal aromatic C–H protons, seen in structures of low coordination number, will contribute to the overall stability of the complex. A striking example is provided by **2l**, which requires the specific orientation of two phenyl rings for optimal C–H hydrogen bonding, where the other phenyl rings in the structure are disordered.

### Relative nucleophilic reactivity of ROH fluoride complexes

In the original studies of TBAF(*t*-BuOH)_4_ as a fluorinating agent, Kim and co-workers examined displacement reactions of **3a** and **3b**.^9,10^ The bromide was less selective than the mesylate and gave mixtures of the alkene **5** and fluoride **4** in which the latter predominated. The conditions used in this prior work provided a basis for systematic examination of several of the compounds characterized by X-ray diffraction as described above, as controlled sources of fluoride ion acting as nucleophile. The results show a range of reactivity of >100 fold on variation of the hydrogen-bond donor alcohol, as shown in Table 2.

**Table 2:** Reactions of alcohol–fluoride complexes with **3b** in CH_3_CN. Conditions: 2× excess of (ROH)_*a*_F^–^:**3b**, CH_3_CN, 70 °C

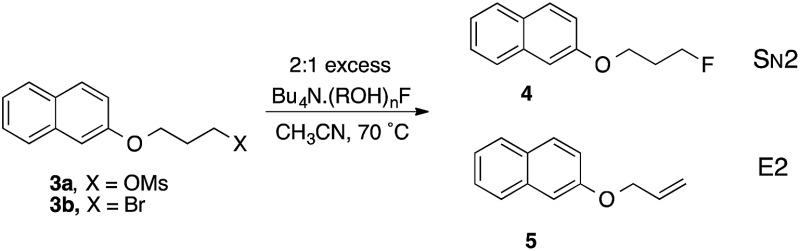						
Entry	Complex	C.N.^*a*^	*M* ^*b*^	*k*_2_, M^–1^ s^–1^	*k*_2_ (rel)	[4]/[5]
1	**2m**	2	0.50	0.0251	4.1	2.1
2	**2m** ^*c*^	2	0.13	0.0870	14	1.03
3	**2m** ^*c*^	2	0.05	0.123	20	0.74
4	**2m** ^*d*^	2	0.50	0.0142	2.3	3.6
5	**2j**	2, 3	0.50	0.0032	0.52	3.5
6	TBAF(H_2_O)_3_	3	0.50	0.0061	1.0	1.6
7	TBAF(H_2_O)_3_ ^*e*^	3	0.50	0.0075	1.2	4.2
8	TBAF(*t*-BuOH)_4_	4	0.50	0.0130	2.1	2.0
9	**2g**	4	0.50	0.0021	0.35	2.8
10	**2d**	4	0.50	0.0004	0.066	3.1
11	**2c**	4	0.50	0.0002	0.037	4.2

^*a*^Coordination no., in (ROH)_*a*_F^–^.

^*b*^(ROH)_*a*_F, *M*.

^*c*^NB low (ROH)_*a*_F concn.

^*d*^With 4× xs. ROH.

^*e*^In C_7_H_8_.

Taking first the reaction using complex **2m** (entry 1), reaction is rapid and the decline in [**3b**] follows a 2^nd^ order decay over the first 600 s, subsequently reacting more slowly. The product is partitioned between S_N_2 and E2 pathways, with the dominance of the former increasing slightly over time. Running the same reaction at higher dilution of both components demonstrates dramatic changes that increase reactivity and decrease S_N_2 selectivity (entries 2, 3). This is consistent with partial or complete dissociation of the L_2_F^–^ complex to give more reactive LF^–^, or free F^–^ that becomes kinetically dominant at low concentration. Carrying out reaction with 1 M excess alcohol **1m** present (entry 4) gives a slower rate but substantially higher S_N_2 selectivity. As a representative of the (ROH)_3_F^–^ class, the 9-phenylfluoren-9-ol derived complex **2j** reacts 8 times more slowly than **2m** and gives a lower proportion of product by the E2 pathway (entry 5).^23^

Commercial TBAF(H_2_O)_3_ was used as a benchmark of reactivity (entry 6). In CH_3_CN the reaction is relatively unselective between S_N_2 and E2 pathways, but occurs with higher S_N_2 selectivity in toluene (entry 7). Surprisingly, the *t*-BuOH complex that proved so useful in allylic fluorination,^6,7^ proved relatively unselective under these conditions (entry 8).

Interesting contrasts were observed by using fluoride-chelating diols (entries 9–11). With the pinacol complex **2g**, the rate and selectivity are comparable to **2j**. For the two 1,3-diol complexes **2d** and **2c** the rates are considerably lower, and the slower **2c** provides the highest S_N_2 selectivity observed in the series. Inspection of the crystal structure of **2g** shows that the O–C–C–O units are *gauche* with dihedral angles of –68.0(2)° and –70.5(2)°, similar to the preferred tGg′ ground state of free pinacol derived by spectroscopy and QM.^24^ For neopentyl glycol **1d**, the preferred *C*_2_ symmetric GG conformation of the chelating unit,^25^ is maintained in the X-ray structures, as preferred in the free diols. Hence there is no evidence of additional strain caused by complexation in either 1,2-diol or 1,3-diol fluoride anion complexes. The main structural difference between the 1,2- and 1,3-diol complexes lies in the chelate angle O···F···O that defines H-bonding, which is 68.24(5)° and 69.11(5)° for the two independent pinacol units in **2g**, contrasting with 78.62(4)° and 79.12(5)° for the typical 1,3-diol complex **2d**. If the wider angle in the 1,3-diol case is associated with greater stability, then the 1,2-diol complex will dissociate one pinacol more easily and hence create an active nucleophilic entity more readily. This is consistent with the observed 5–10 fold higher reactivity of **2g** compared with **2c** or **2d**.

**Fig. 11:**
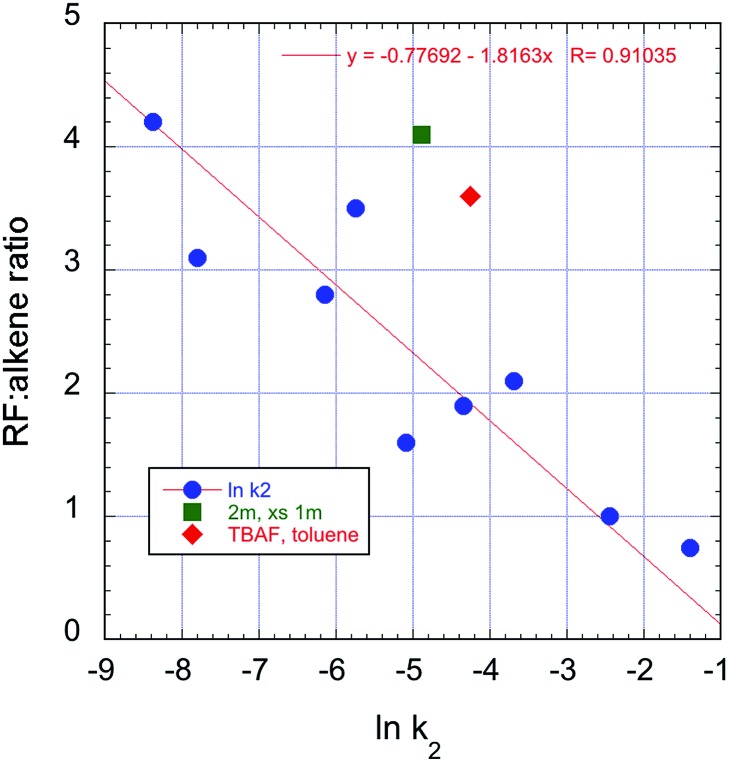
Correlation between rate and S_N_2/E2 selectivity in the reactions of **3b** with TBAF(ROH)_*n*_, 70 °C, CH_3_CN. The additional points represent entry 4 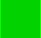 and entry 7 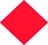, Table 2.

Overall, there is a correlation between the rates of displacement and the S_N_2/E2 selectivity. The clear trend towards reduced S_N_2 selectivity with increasing rate can be seen in Fig. 11. Stronger complexation of fluoride ion is observed with ureas, and this leads to significantly slower rates of substitution with **3b** and higher selectivity towards formation of product **4**.^26^

## Conclusions

From the large number of studies on nucleophilic fluorination, it appears that the nature of the fluoride reagent is critical for a particular transformation to succeed; the reasons why one fluoride source is superior to another are more often unknown. As a result, an empirical approach that involves the systematic screen of commercially available F^–^ reagents is typically undertaken when developing nucleophilic fluorination processes. This work provides new information on the coordination chemistry and relative reactivity of a range of novel fluoride–alcohol complexes; some key findings are listed below.

(a) The synthesis and characterization by single-crystal X-ray diffraction of fourteen fluoride–alcohol complexes derived from alcohols, 1,2-diols, 1,3-diols, triols and tetraols demonstrate that tetra-, tri-, or dicoordinate fluoride–alcohol complexes can be formed. This variability in coordination stoichiometry had not been observed previously.

(b) For alcohols, the coordination number to fluoride varies from two to four, and decreases as the degree of branching and steric bulk of the alcohol is increased.

(c) Complexes with lower coordination number tend to have shorter O···F (and therefore shorter H···F) distances.

(d) Complexes derived from 1,2- and 1,3-diols display a range of interligand angles; this suggests that the packing forces imposed by the ligand are more influential than the inclination of fluoride to form complexes of tetrahedral geometry. The complex derived from pentaerythrol is unique forming a linear polymeric structure with an interplanar angle between the O···F···O units of 50.54(12)°.

(e) The structural features in the solid state of hydrogen bonded fluoride–alcohol complexes provide insight into the ability of these complexes to dissociate in solution; such dissociation releases a more active fluoride source that influences rate and S_N_2/E2 selectivity. For fluoride complexes derived from chelating 1,2- and 1,3-diols, the ability to dissociate to give an active nucleophilic entity depends on the chelate O···F···O angle that defines hydrogen bonding since this angle influences complex stability.

(f) In solution at high dilution, the fluoride complexes L_*n*_F^–^ partially or completely dissociate; as a result, reactivity increases but S_N_2 *versus* E2 selectivity decreases.

(g) Many complexes reported here form crystalline solids that are easy to handle and are less hygroscopic than TBAF(H_2_O)_3_ and TBAF(*t*-BuOH)_4_.

This work has demonstrated that fluoride–alcohol complexes display structural diversity in the solid state; this key observation implies that there will be significant variabilities on the ability of these complexes to dissociate in solution. This observation underscores the importance of structural analysis in the solid state combined with kinetic studies as a platform to understand fluoride reactivity. Ongoing work, applying experimental and computational methods, focuses on the examination of a larger range of small-molecule hydrogen-bond donors to activate inexpensive and widely available sources of fluoride for applications in synthesis, catalysis and [^18^F] radiochemistry.^27–33^

## Experimental

For the preparation of TBAF–alcohol complexes, a flask was charged with TBAF(H_2_O)_3_ (1.0 eq.), and the alcohol (1.0–4.0 eq.) was added under an atmosphere of N_2_. Hexane was added, and the mixture was refluxed for 2 h, during which time droplets of water formed on the inside walls of the condenser, before letting it cool to RT. The solid products were collected by filtration, washed with hexane and dried under high vacuum, giving the desired complexes, which were used without further purification. Products were stored under an atmosphere of N_2_. Single-crystals suitable for X-ray analysis were obtained by recrystallization from THF, EtOAc or DCM by reducing solubility in a saturated solution through slow mixing with hexanes using a layering or vapour diffusion technique. See the ESI† for details regarding individual compounds.

Low temperature (150 K) single-crystal X-ray diffraction data,^27^ were collected using either a Nonius Kappa CCD diffractometer or an Oxford Diffraction (Agilent) SuperNova A diffractometer and reduced using the appropriate instrument manufacturer supplied software.^28^ Structures were solved using either SIR92,^29^ or SuperFlip,^30^ and refined using full-matrix least-squares refinement with CRYSTALS.^31^ In the case of **2m**, there was a small amount of diffuse residual electron density believed to be disordered solvent. This was modelled using PLATON/SQUEEZE,^32^ within CRYSTALS. On refinement of **2g**, there was a poor agreement between the observed and calculated structure factor amplitudes. Examination of the data and model using ROTAX,^33^ suggested the crystal was a pseudo-merohedral twin that was included in the refinement. For further details see the full crystallographic data (in CIF format) which are available as ESI.†

## References

[cit1] Phelps M. E. (2000). Proc. Natl. Acad. Sci. U. S. A..

[cit2] Aigueperse J., Mollard P., Devilliers D., Chemla M., Faron R., Romano R. and Cuer J.-P., Fluorine Compounds, Inorganic, Ullman's Encyclopedia of Industrial Chemistry, Wiley-VCH, Weinheim, 2005, p. 307.

[cit3] Furuya T., Kamlet A. S., Ritter T. (2011). Nature.

[cit4] O'Hagan D., Schaffrath C., Cobb S. L., Hamilton J. T. G., Murphy C. D. (2002). Nature.

[cit5] Dong C., Huang F., Deng H., Schaffrath C. (2004). Nature.

[cit6] Hollingworth C., Hazari A., Hopkinson M. N., Tredwell M., Benedetto E., Huiban M., Gee A. D., Brown J. M., Gouverneur V. (2011). Angew. Chem., Int. Ed..

[cit7] Benedetto E., Tredwell M., Hollingworth C., Khotavivattana T., Brown J. M., Gouverneur V. (2013). Chem. Sci..

[cit8] Emsley J. (1980). Chem. Soc. Rev..

[cit9] Yonezawa T., Sakamoto Y., Nogawa K. (1994). Jpn. Kokai Tokkyo Koho.

[cit10] Kim D. W., Jeong H.-J., Lim S. T., Sohn M.-H., Katzenellenbogen J. A., Chi D. Y. (2008). J. Org. Chem..

[cit11] Kim D. W., Jeong H.-J., Lim S. T., Sohn M.-H. (2008). Angew. Chem., Int. Ed..

[cit12] Yan H., Jang H. B., Lee J.-W., Kim H. K., Lee S. W., Yang J. W., Song C. E. (2010). Angew. Chem., Int. Ed..

[cit13] Birrell J. A., Desrosiers J.-N., Jacobsen E. N. (2011). J. Am. Chem. Soc..

[cit14] DeTuri V. F., Su M. A., Ervin K. M. (1999). J. Phys. Chem. A.

[cit15] Bogdanov B., McMahon T. B. (2000). J. Phys. Chem. A.

[cit16] Chen C.-H., Leung M. K. (2011). Tetrahedron.

[cit17] Alvarez S., Alemany P., Casanova D., Cirera J., Llunell M., Avnir D. (2005). Coord. Chem. Rev..

[cit18] In several cases the results arising from analysis of the coordination sphere based on O···F distances were checked by a parallel analysis treating the hydrogen-bonding H···F distances directly. The conclusions were not altered

[cit19] Desiraju G. R. and Steiner T., The Weak Hydrogen Bond in Structural Chemistry and Biology, Oxford University Press, New York, 1999.

[cit20] Wang Q.-Q., Day V. W., Bowman-James K. (2012). Angew. Chem., Int. Ed..

[cit21] Craig J. D. C., Brooker M. H. (2000). J. Solution Chem..

[cit22] Steiner T., Desiraju G. R. (1998). Chem. Commun..

[cit23] The original paper (ref. 10) indicated a higher extent of substitution with RF : alkene = 3.76 with TBAF(*t*-BuOH)_4_. We note, however, that the relevant data point from entry 8 falls on the trend line in Fig. 11

[cit24] Dahlqvist M., Hotokka M., Räsänen M. (1998). Chem. Phys..

[cit25] Granzow B., Klaeboe P., Sablinskas V. (1995). J. Mol. Struct..

[cit26] Pfeifer L., Pidgeon G. W., Engle K. M., Brown J. M. and Gouverneur V., to be published.

[cit27] Cosier J., Glazer A. M. (1986). J. Appl. Crystallogr..

[cit28] Otwinowski Z. and Minor W., in Methods in Enzymology, ed. C. W. Carter, Jr. and R. M. Sweet, Academic Press, New York, 1997, vol. 276, pp. 307–326. 10.1016/S0076-6879(97)76066-X 27754618

[cit29] Altomare A., Cascarano G., Giacovazzo C., Guagliardi A., Burla M. C., Polidori G., Camalli M. (1994). J. Appl. Crystallogr..

[cit30] Palatinus L., Chapuis G. (2007). J. Appl. Crystallogr..

[cit31] Betteridge P. W., Carruthers J. R., Cooper R. I., Prout K., Watkin D. J. (2003). J. Appl. Crystallogr..

[cit32] Spek A. L. (2003). J. Appl. Crystallogr..

[cit33] Cooper R. I., Gould R. O., Parsons S., Watkin D. J. (2002). J. Appl. Crystallogr..

[cit34] Dunitz J. D., Taylor R. (2006). Chem.–Eur. J..

