# Supplementary material for: Coordination diversity in hydrogen-bonded homoleptic fluoride–alcohol complexes modulates reactivity
Source: Chem Sci. 2015 Jun 22;6(9):5293–302. doi: 10.1039/c5sc01812a (PMC5669313; doi:10.1039/c5sc01812a)
Supplement: Supplementary file 1 [file SC-006-C5SC01812A-s001.pdf]

Electronic Supplementary Material (ESI) for Chemical Science.
This journal is © The Royal Society of Chemistry 2015

Coordination Diversity in Hydrogen-Bonded Homoleptic Fluoride–Alcohol Complexes Modulates Reactivity

Keary M. Engle[1], Lukas Pfeifer[1], George W. Pidgeon[1], Guy T. Giuffredi[1], Amber L. Thompson[1], Robert S. Paton[1], John M. Brown[1], Véronique Gouverneur[1]*

[1] Chemistry Research Laboratory, University of Oxford, 12 Mansfield Road, Oxford OX1 3TA, UK.

SUPPORTING INFORMATION

Table of Contents

General Information……………………………………………………..........pages S-2 – S-3

Experimental Section…………………………………...……….........pages S-3 – S-14

Crystal Structures………………………………………......................pages S-15 – S-16

Reaction Kinetics…………………………….……………….................pages S-17 – S-52

Alcohols as Additives………………………….…………......................page S-53

References………………………………………………………………… page S-54

NMR Spectra……………………………………………………………… pages S-55 – S-94

General Information

Unless otherwise noted, all materials were used as received from commercial sources without further purification. TMAF$(H_2O)_4$, TEAF$(H_2O)_2$, and TBAF$(H_2O)_3$ were purchased from Acros, Sigma-Aldrich, and Apollo, respectively. 1,3,3-Trimethyl-1,3-dihydroisobenzofuran-1-ol (**1i**) was synthesized from diethyl phthalate purchased from Sigma-Aldrich using the procedure described below. Pyrrolidine-2,5-diylbis(diphenylmethanol) (**1n**) was synthesized from diethyl *meso*-2,5-dibromoadipate, benzylamine and phenylmagnesium bromide, all purchased from Sigma-Aldrich, using the three-step procedure described below. All other alcohol hydrogen-bond donors were purchased from Sigma-Aldrich or Alfa Aesar. 2-Naphthol and 3-bromo-1-propanol were purchased from Sigma-Aldrich. 1,3-Dibromopropane was purchased from Fluorochem.

All NMR spectra were recorded on Bruker DPX250, AV400, AVC500, AVB500 and DRX500 spectrometers. ^{1}H and ^{13}C NMR spectra are reported as chemical shifts (δ) in parts per million (ppm) relative to the solvent peak using the Bruker internal referencing procedure (edlock). Coupling constants (*J*) are reported in units of hertz (Hz). The following abbreviations are used to describe multiplicities: s (singlet), d (doublet), t (triplet), q (quartet), qn (quintet), sx (sextet), m (multiplet), bs (broad singlet). High resolution mass spectra (HRMS, m/z) were recorded on a Bruker MicroTOF spectrometer using positive electrospray ionization (ESI^+) or on a Micromass GCT spectrometer using field ionization (FI^+) or chemical ionization (CI^+). Infrared spectra were recorded either as the neat compound or in a solution using a Bruker Tensor 27 FT-IR spectrometer. Absorptions are reported in wavenumbers (cm^{-1}), and only peaks of interest are reported. Melting points of solids were measured on a Griffin apparatus and are uncorrected. IUPAC names were obtained using the ACD/I-Lab service. All reactions were performed in a flame-dried vessel with magnetic stirring under an inert atmosphere. All solvents were dried on a column of alumina prior to use. Flash column chromatography was performed over Merck Geduran® silica gel 60 (40–63 μm) using eluent systems as described for each experiment. TBAF(*t*-BuOH)$_4$ was prepared according to the procedure of Kim.[1]

In general, low temperature[2] (150 K) single-crystal X-ray diffraction data were collected using either a Nonius Kappa CCD diffractometer or an Oxford Diffraction (Agilent) SuperNova A diffractometer. Raw frame data were reduced using the appropriate instrument manufacturer supplied software, either DENZO-SMN/SCALEPACK[3] or CrysAlisPro.[4] All structures could be solved *ab initio* using either SIR92[5] or SuperFlip,[6] and full-matrix least-squares refinement was carried out using CRYSTALS.[7,8,9] In general, all non-hydrogen atoms were refined using anisotropic displacement ellipsoids, and hydrogen atoms were visible in the difference map. Once the heavy atoms structure was complete, hydrogen atoms were positioned geometrically then refined separately using soft restraints prior to inclusion in the final refinement using a riding model.[10] Where disorder was identified, the structure was modelled with multiple sites using same distance and thermal similarity restraints to maintain a sensible model. In the case of **2m**, there was a small amount of diffuse residual electron density believed to be disordered solvent. This was modelled using PLATON/SQUEEZE[11] within CRYSTALS. On refinement of **2g**, there was a poor agreement between the observed and calculated structure factor amplitudes. Examination of the data and model using ROTAX[12] suggested the crystal was a pseudo-merohedral twin which was included in the refinement. For further details see the CIF. F···O distances and O···F···O angles were calculated with CRYSTALS using the full variance-

covariance matrix, and the dihedral angles were calculated using PLATON.[13,14] All structures have been deposited with the Cambridge Crystallographic Data Centre (reference codes CCDC 1401765–1401778); these data can be obtained free of charge via www.ccdc.cam.ac.uk/data_request/cif.

Experimental Section

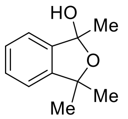

1i

1,3,3-Trimethyl-1,3-dihydroisobenzofuran-1-ol (1i): The title compound was prepared using a modified literature precedent.[15,16] To a flame-dried 250 mL three-neck flask containing a magnetic stir bar, were added anhydrous Et_2O (100 mL) and diethyl phthalate (2.22 g, 10.0 mmol). The solution was cooled to 0 °C in an ice bath, and MeMgBr solution (3.0 M in Et_2O, 20.0 mL, 60.0 mmol) was added slowly, during which time the reaction mixture thickened and became cloudy. The solution was allowed to warm to room temperature and stir for 6 h. The reaction was carefully quenched with 2.0 N HCl solution (50 mL). The phases were separated, and aqueous phase was extracted with Et_2O (2 × 100 ml). The combined organic layers were washed sequentially with sat. $NaHCO_3$ solution (100 mL) and brine (100 mL), dried over Na_2SO_4, filtered and concentrated *in vacuo*. The resulting white solid was purified by silica gel column chromatography (10:1 hexanes:EtOAc → 2:1 hexanes:EtOAc), giving **1i** as a white solid (806 mg, 41%). **M.p.** = 95–98 °C; **^{1}H NMR** (400 MHz, $CDCl_3$) δ 7.40–7.15 (m, 4H), 2.91 (bs, 1H), 1.78 (s, 3H), 1.60 (s, 3H), 1.50 (s, 3H); **^{13}C NMR** (100 MHz, $CDCl_3$) δ 146.87, 141.24, 129.16, 127.98, 122.02, 120.57, 105.94, 84.49, 30.76, 29.26, 28.15; **IR** (film, $CDCl_3$) ν 3405, 2972, 2927, 1456, 1352, 1133, 1076, 965, 756 cm^{-1}; **HRMS** (EI/FI) *m/z* Calcd for $C_{11}H_{14}O_2$ $[M]^+$ 178.0994, found 178.0994.

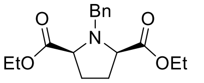

Diethyl 1-benzylpyrrolidine-2,5-dicarboxylate: The title compound was prepared according to a procedure described by Fraenkel.[17] A flame-dried 100 mL two-neck flask containing a magnetic stir bar was charged with diethyl *meso*-2,5-dibromoadipate (10.0 g, 27.8 mmol). Benzene (30 mL) was added to dissolve the starting material before the resulting solution was heated to gentle reflux. The heat was removed and benzylamine (10.6 mL, 97.2 mmol) was added dropwise maintaining reflux. After complete addition the mixture was stirred at reflux for 16 h. After cooling to room temperature solids were removed by filtration, the mixture was concentrated *in vacuo* and silica gel column chromatography (10:1 hexanes:EtOAc → 5:1 hexanes:EtOAc) gave the title compound (3.26 g, 38%) as a colorless oil. **^{1}H NMR** (400 MHz, $CDCl_3$) δ 7.35–7.30 (m, 2H), 7.30–7.24 (m, 2H), 7.24–7.18 (m, 1H), 4.07–3.95 (m, 4H), 3.93 (s, 2H), 3.45–3.37 (m, 2H), 2.11–2.00 (m, 4H), 1.18 (t, *J* = 7.2 Hz, 6H); **^{13}C NMR** (100 MHz, $CDCl_3$) δ 173.23, 137.40, 129.35, 127.84, 127.02, 65.42, 60.39, 57.63, 28.52, 13.97; **IR** (neat) ν 3086, 3063, 3028, 2981, 2939, 2904, 2874, 2831, 2361, 2340, 1741, 1727, 1604, 1586, 1495, 1454, 1447, 1371, 1349, 1272, 1178, 1148, 1097, 1072, 1028, 969, 913, 852, 811, 751, 700, 619 cm^{-1}; **HRMS** (ESI) *m/z* Calcd for $C_{17}H_{24}NO_4$ $[M+H]^+$ 306.1700, found 306.1699.

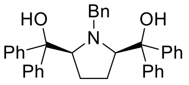

(1-Benzylpyrrolidine-2,5-diyl)bis(diphenylmethanol): To a 100 mL round-bottom flask containing a magnetic stir bar, was added phenylmagnesium bromide (2.8 M in Et_2O, 21.6 mmol, 7.72 mL), before it was cooled to 0 °C. Diethyl 1-benzylpyrrolidine-2,5-dicarboxylate (4.90 mmol, 1.50 g) dissolved in Et_2O (20 mL) was added dropwise, and the resulting mixture was allowed to warm to room temperature. It was then heated to reflux for 4 h, cooled to 0 °C and quenched with sat. NH_4Cl solution (20 mL). The phases were separated, and the aqueous layer was extracted with DCM (3 × 30 mL). The combined organic layers were dried over $MgSO_4$, filtered and concentrated *in vacuo*. Silica gel column chromatography (20:1 hexanes: EtOAc → 10:1 hexanes:EtOAc) gave the title compound (1.94 g, 75%) as a white solid. **M.p.** = 175 °C; **^{1}H NMR** (400 MHz, $CDCl_3$) δ 7.63 (d, *J* = 7.6 Hz, 4H), 7.53 (d, *J* = 7.3 Hz, 4H), 7.35–7.25 (m, 1H), 7.23–7.14 (m, 4H), 6.95–6.90 (m, 2H), 4.74 (s, 2H), 4.55–4.48 (m, 2H), 2.39 (s, 2H), 1.96–1.84 (m, 4H); **^{13}C NMR** (100 MHz, $CDCl_3$) δ 147.01, 146.39, 138.35, 128.74, 128.20, 128.09, 127.92, 126.74, 126.59, 126.37, 126.09. 126.01, 80.15, 70.29, 55.77, 28.88; **IR** (solid) ν 3411, 3293, 3085, 3060, 3029, 2959, 2930, 2879, 2361, 2341, 1599, 1493, 1447, 1389, 1360, 1325, 1312, 1255, 1226, 1188, 1156, 1128, 1078, 1066, 1043, 1032, 1001, 979, 929, 891, 859, 846, 805, 769, 747, 696, 659, 636 cm^{-1}; **HRMS** (ESI) *m/z* Calcd for $C_{37}H_{36}NO_2$ $[M+H]^+$ 526.2741, found 526.2732.

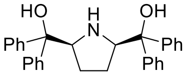

1n

Pyrrolidine-2,5-diylbis(diphenylmethanol) (1n): The title compound was prepared using a modified literature precedent.[18] To a 100 mL round-bottom flask containing a magnetic stir bar, were added (1-benzylpyrrolidine-2,5-diyl)bis(diphenylmethanol) (1.60 g, 3.04 mmol), $Pd(OH)_2$ (20% on C, 395 mg, 0.56 mmol) and MeOH (40 mL). The flask was evacuated and backfilled with H_2 (×3), and the reaction was left to stir under a H_2 atmosphere at room temperature for 48 h. The catalyst was removed by filtration through Celite, and the filtrate was washed with MeOH (80 mL) to remove impurities. Washing with EtOAc (210 mL) and removing this solvent *in vacuo* gave the crude product which was purified by silica gel column chromatography (10:1 hexanes:EtOAc → 4:1 hexanes:EtOAc) to give **1n** (730 mg, 55%) as a white solid. **M.p.** = 148–149 °C; **^{1}H NMR** (400 MHz, $CDCl_3$) δ 7.52 (d, *J* = 7.6 Hz, 4H), 7.45 (d, *J* = 7.6 Hz, 4H), 7.31–7.24 (m, 8H), 7.20–7.13 (m, 4H), 4.42 (t, *J* = 5.4 Hz, 2H), 3.69 (s, 2H), 1.89 (bs, 1H), 1.80–1.68 (m, 2H), 1.54–1.43 (m, 2H); **^{13}C NMR** (100 MHz, $CDCl_3$) δ 147.01, 144.91, 128.40, 128.00, 126.82, 126.51, 125.76, 125.34, 77.36, 64.10, 25.33; **IR** (solid) ν 3421, 3059, 3025, 2960, 2874, 2361, 2341, 2186, 2163, 1620, 1598, 1582, 1491, 1448, 1401, 1340, 1302, 1275, 1244, 1185, 1130, 1059, 1032, 1011, 1004, 986, 970, 910, 874, 820, 793, 747, 695, 667, 660, 633 cm^{-1}; **HRMS** (ESI) *m/z* Calcd for $C_{30}H_{30}NO_2$ $[M+H]^+$ 436.2271, found 436.2268.

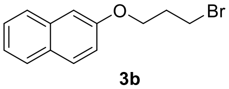

3b

2-(3-bromopropoxy)naphthalene (3b): The title compound was prepared according to a modified version of a procedure developed by Kim and coworkers.[1] To a 250 mL round-bottom flask containing a magnetic stir bar, were added 2-naphthol (2.02 g, 14.0 mmol), 1,3-dibromopropane (280 mmol, 28.6 mL), K_2CO_3 (140 mmol, 19.3 g), and DMF (2.0 mL). The resulting slurry solution was stirred at 50 °C for 48 h. The reaction mixture was cooled to room temperature, and water (100 mL) was added. The

resulting biphasic mixture was extracted with CH_2Cl_2 (3 × 100 mL). The combined organic layers were washed with brine (100 mL), dried over $MgSO_4$, filtered, and then concentrated *in vacuo*. Excess 1,3-dibromopropane (b.p. = 166–167 °C) was removed using a rotary evaporator connected to a well-maintained vacuum pump and a water bath at elevated temperature (70–80 °C). The resulting residue was further dried under high vacuum for several hours. Purification via silica gel column chromatography (10:1 hexanes:DCM → 4:1 hexanes:DCM) delivered analytically pure **3b** as a colorless oil, which over time transformed into a white crystalline solid (2.56 g, 69% yield). **^{1}H NMR** (400 MHz, $CDCl_3$) δ 7.78–7.72 (m, 3H), 7.45 (ddd, J_1 = 8.4 Hz, J_2 = 7.2 Hz, J_3 = 1.2 Hz, 1H), 7.35 (ddd, J_1 = 8.0 Hz, J_2 = 6.8 Hz, J_3 = 1.2 Hz, 1H), 7.17–7.13 (m, 2H), 4.23 (t, J = 6.0 Hz, 2H), 3.66 (t, J = 6.8 Hz, 2H), 2.39 (qn, J = 6.0 Hz, 2H); **^{13}C NMR** (100 MHz, $CDCl_3$) δ 156.60, 134.49, 129.44, 129.02, 127.63, 126.74, 126.40, 123.59, 118.77, 106.71, 65.30, 32.33, 30.07. **IR** (solid) ν 3060, 2949, 2899, 1629, 1599, 1510, 1461, 1390, 1254, 1215, 1182, 1027, 841, 816, 748 cm^{-1}; **HRMS** (EI/FI) *m/z* Calcd for $C_{13}H_{13}BrO$ $[M]^+$ 264.0150, $[(M+2)]^+$ 266.0130, found 264.0146, 266.0153 $[M]^+$: $[(M+2)]^+$ = 1:1.

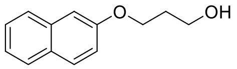

3-(naphthalen-2-yloxy)propan-1-ol: The title compound was prepared according to a procedure described by Shi and He.[19] To a 250 mL round bottom flask equipped with a magnetic stir bar, were added distilled water (100 mL) and naphthol (7.21 g, 50 mmol), giving a suspension. NaOH pellets were added one-by-one until the mixture became a transparent, homogenous, purple solution. 3-Bromopropanol (2.70 mL, 30 mmol) was added dropwise. The flask was capped with a rubber septum, and the reaction mixture was allowed to stir at room temperature for 24 h. The aqueous reaction mixture was extracted with CH_2Cl_2 (3 × 100 mL). The combined organic layers were washed with H_2O (3 × 100 mL), dried over $MgSO_4$, filtered, and concentrated *in vacuo* to give the crude product as an off-white solid in approximately 60% yield by mass. To remove trace quantities of 3-bromopropanol, which proved to be difficult to separate via silica gel column chromatography, the crude product was recrystallized (10:1 Et_2O:hexanes), giving analytically pure title compound as an off-white solid (852 mg, 14% yield). **^{1}H NMR** (400 MHz, $CDCl_3$) δ 7.78–7.72 (m, 3H), 7.44 (at, J = 8.0 Hz, 1H), 7.34 (at, J = 8.0 Hz, 1H), 7.16–7.13 (m, 2H), 4.25 (t, J = 6.0 Hz, 2H), 3.92 (t, J = 6.0 Hz, 2H), 2.12 (qn, J = 6.0 Hz, 2H), 1.70 (s, 1H); **^{13}C NMR** (100 MHz, $CDCl_3$) δ 156.68, 134.50, 129.41, 128.99, 127.63, 126.74, 126.38, 123.66, 118.77, 106.68, 65.74, 60.58, 31.96; **IR** (film, $CDCl_3$) ν 3267, 2955, 2878, 1629, 1600, 1509, 1467, 1389, 1261, 1219, 1184, 1055, 1040, 837, 815, 749 cm^{-1}; **HRMS** (ESI) *m/z* Calcd for $C_{13}H_{14}NaO_2$ $[M+Na]^+$ 255.0886, found 225.0880.

General Procedure for Synthesizing [Tetraalkylammonium Fluoride–Alcohol] Complexes: To a flame-dried round bottom flask under a nitrogen atmosphere and equipped with a stir bar and reflux condenser, were added hexanes (0.033 M), tetraalkylammonium fluoride hydrate and the alcohol hydrogen-bond donor. The resulting suspension was allowed to stir in an oil bath at vigorous reflux (80–85 °C) for 2 h, during which time droplets of water were observed on the inside walls of the reflux condenser. During the course of the reaction the persistent presence of

a white solid precipitate was observed. The solution was allowed to cool to room temperature, filtered quickly, washed with hexanes (3 × 50 ml), and dried under high vacuum, giving the desired [tetraalkylammonium fluoride–alcohol] complex, which was used without further purification.

Note: In all ^{13}C NMR spectra of [tetraalkylammonium fluoride–alcohol] complexes taken in $CDCl_3$, a singlet was observed at approximately 77.20 ppm, which we attribute to $CHCl_3$. We surmise that $CHCl_3$ is formed *in situ* from $CDCl_3$ via proton exchange, which is presumably mediated by fluoride acting as a Brønsted base. This signal has been omitted from the peak listings below.

$$Bu_4NF\cdot(H_2O)_n \xrightarrow[\text{hexanes, reflux, 2 h}]{\text{X eq. R'OH}} Bu_4NF\cdot(R'OH)_m$$

Scheme S1: General procedure for synthesizing [tetraalkylammonium fluoride–alcohol] complexes.

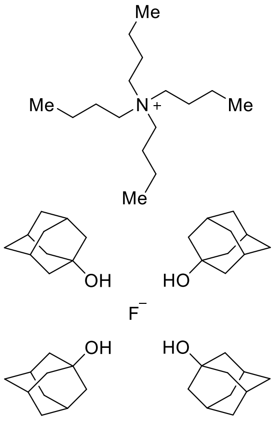

TBAF(1-Ada-OH)$_4$, **2a**

Tetra-*N*-butylammonium fluoride tetra(1-adamantanol) (TBAF(1-Ada-OH)$_4$, 2a): The title compound was prepared according to the general procedure with 4.0 mmol of TBAF(H_2O)$_3$ and 16.0 mmol of 1-adamantanol. The complex was thus obtained as crystalline white solid (2.92 g, 84% yield). **^{1}H NMR** (400 MHz, $CDCl_3$) δ 3.37–3.34 (m, 8H), 2.12 (as, 12H), 1.70–1.55 (m, 56H), 1.47–1.40 (m, 8H), 1.02–0.97 (m, 12H); **^{13}C NMR** (100 MHz, $CDCl_3$) δ 66.84, 58.23, 44.97, 35.86, 30.33, 23.73, 19.34, 13.33; **IR** (solid) ν 3088, 2903, 2846, 1428, 1348, 1300, 1281, 1127, 1090, 934, 881 cm^{-1}. **X-ray** (single-crystal) Colorless needles of X-ray diffraction quality were obtained by vapor diffusion of hexanes into a saturated solution of **2a** in EtOAc, which had previously been prepared by dissolving **2a** in refluxing EtOAc (approximately 77 °C) until visibly saturated, filtering while hot, and then allowing to stand for approximately 1 min to cool to near room temperature. Experimental details are provided below.

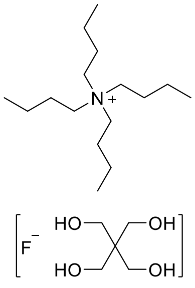

TBAF·PERT, **2b**

Tetra-*N*-butylammonium fluoride pentaerythritol (TBAF•PERT, 2b): The title compound was prepared according to the general procedure with 2.0 mmol of TBAF(H_2O)$_3$ and 2.0 mmol of pentaerythritol. The complex was thus obtained as a crystalline white solid (616 mg, 77% yield). **^{1}H NMR** (400 MHz, CD_2Cl_2) δ 6.18 (bs, 2H), 3.47 (s, 8H), 3.21–3.17 (m, 8H), 3.04 (bs, 2H), 1.63 (qn, J = 7.2 Hz, 8H), 1.42 (sx, J = 7.2 Hz, 8H), 1.01 (t, J = 7.2 Hz, 12H); **^{13}C NMR** (100 MHz, CD_2Cl_2) δ 63.94, 59.27, 46.24, 24.39, 20.25, 13.95; **IR** (solid) ν 3197, 2961, 2930, 2873, 1489, 1463, 1374, 1124, 1017, 886, 742 cm^{-1}; **X-ray** (single-crystal) Colorless plate-like crystals of X-ray diffraction quality were obtained by carefully layering hexanes onto a saturated solution of **2b** in DCM, which had

previously been prepared by dissolving **2b** in refluxing DCM (approximately 40 °C) until visibly saturated, filtering while hot, and then allowing to stand for approximately 1 min to cool to near room temperature. The crystal was found to be a DCM solvate. Experimental details are provided below.

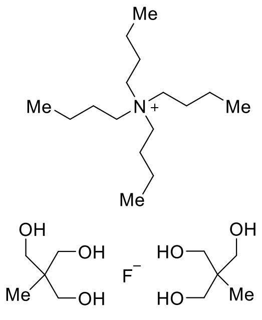

TBAF(TME)$_2$, **2c**

Tetra-*N*-butylammonium fluoride bis(1,1,1-tris(hydroxymethyl)ethane) (TBAF(TME)$_2$, 2c): The title compound was prepared according to the general procedure with 4.0 mmol of TBAF(H_2O)$_3$ and 8.0 mmol of 1,1,1-tris(hydroxymethyl)ethane. After heating at reflux for 2 h, a viscous oil was observed at the bottom of the reaction vessel, which solidified when allowed to stand at room temperature for 8 h. Following the standard work-up, **2c** was obtained as a chalky white solid (1.90 g, 95% yield). **^{1}H NMR** (400 MHz, $CDCl_3$) δ 3.61 (s, 12H), 3.25–3.21 (m, 8H), 1.67–1.59 (m, 8H), 1.43 (sx, *J* = 7.2 Hz, 8H), 1.01 (t, *J* = 7.2 Hz, 12H), 0.66 (s, 6H), **^{13}C NMR** (100 MHz, $CDCl_3$) δ 67.71, 58.71, 41.00, 23.89, 19.70, 17,38, 13.63; **IR** (solid) ν 3337, 3181, 2962, 2929, 2876, 1473, 1039, 885 cm^{-1}. **X-ray** (single-crystal) Colorless block crystals of X-ray diffraction quality were obtained by vapor diffusion of hexanes into a saturated solution of **2c** in THF, which had previously been prepared by dissolving **2c** in refluxing THF (approximately 66 °C) until visibly saturated, filtering while hot, and then allowing to stand for approximately 1 min to cool to near room temperature. Experimental details are provided below.

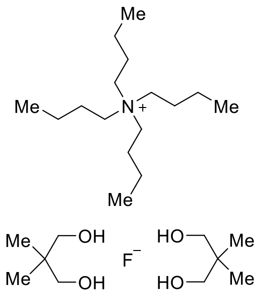

TBAF(NPG)$_2$, **2d**

Tetra-*N*-butylammonium fluoride bis(neopentyl glycol) (TBAF(NPG)$_2$, 2d): The title compound was prepared according to the general procedure with 4.0 mmol of TBAF(H_2O)$_3$ and 8.0 mmol of neopentylglycol. The complex was thus obtained as a crystalline white solid (1.76 g, 89% yield). **^{1}H NMR** (400 MHz, $CDCl_3$) δ 3.27 (s, 8H), 3.20–3.16 (m, 8H), 1.61–1.53 (m, 8H), 1.35 (sx, *J* = 7.6 Hz, 8H), 0.95 (t, *J* = 7.2 Hz, 12H), 0.74 (s, 12H); **^{13}C NMR** (100 MHz, $CDCl_3$) δ 68.74, 58.42, 36.90, 23.75, 21.79, 19.55, 13.52; **IR** (solid) ν 3177, 2960, 2865, 1485, 1354, 1049, 884, 739 cm^{-1}. **X-ray** (single-crystal) Colorless plate-like crystals of X-ray quality were obtained by vapor diffusion of hexanes into a saturated solution of **2d** in THF, which had previously been prepared by dissolving **2d** in THF at room temperature and filtering to remove residual solid. Experimental details are provided below.

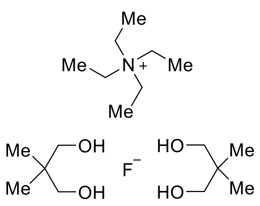

TEAF(NPG)$_2$

Tetra-*N*-ethylammonium fluoride bis(neopentyl glycol) (TEAF(NPG)$_2$): The title compound was prepared according to the general procedure with 4.0 mmol of TEAF(H_2O)$_2$ and 8.0 mmol of neopentylglycol. The complex was thus obtained as a flocculent white solid (1.25 g, 87% yield). **^{1}H NMR** (400 MHz, $CDCl_3$) δ 3.38–3.32 (m, 16H), 1.35 (t, *J* = 7.2 Hz, 12H), 0.83 (s, 12H); **^{13}C NMR** (100 MHz, $CDCl_3$) δ 69.69, 52.43, 36.86, 21.74, 7.53; **IR** (solid) ν 3192, 2985, 1904, 2863,1470, 1393, 1043, 1003, 747 cm^{-1}.

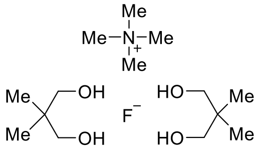

TMAF(NPG)

Tetra-*N*-methylammonium fluoride bis(neopentyl glycol) (TMAF(NPG)$_2$): The title compound was prepared according to the general procedure with 4.0 mmol of TMAF(H_2O)$_4$ and 8.0 mmol of neopentylglycol. The complex was thus obtained as a crystalline white solid (1.11 g, 92% yield). **^{1}H NMR** (200 MHz, $CDCl_3$) δ 3.29 (as, 20H), 0.77 (s, 12H), **^{13}C NMR** (100 MHz, $CDCl_3$) δ 68.68, 55.66, 37.00, 21.80; **IR** (solid) ν 3113, 2955, 2867, 1489, 1392, 1357, 1042, 990 cm^{-1}.

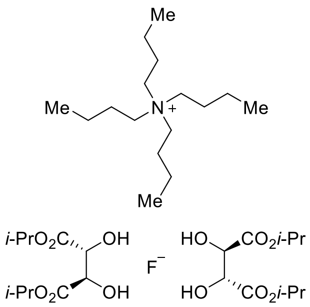

TBAF((+)-DIPT)$_2$, **2e**

Tetra-*N*-butylammonium fluoride bis((+)-diisopropyl L-tartrate)) (TBAF((+)-DIPT)$_2$, 2e): The title compound was prepared according to the general procedure with 4.0 mmol of TBAF(H_2O)$_3$ and 8.0 mmol of (+)-diisopropyl tartrate. After heating at reflux for 2 h, a viscous colorless oil was observed at the bottom of the reaction vessel. The reaction was allowed to cool to room temperature, and the solvent was removed *in vacuo*. After further drying under high vacuum for 8 h, the complex was transferred to a –20 °C for 8 h, at which point it was observed to have solidified. The resulting material was warmed to room temperature and washed with hexanes (×3) on a sinter funnel, then dried under high vacuum. The complex was thus obtained as sticky colorless solid (1.79 g, 61% yield). **^{1}H NMR** (400 MHz, $CDCl_3$) δ 5.09–5.01 (m, 4H), 4.45 (s, 4H), 3.29–3.25 (m, 8H), 1.61 (qn, *J* = 7.6 Hz, 8H), 1.39 (sx, *J* = 7.6 Hz, 8H), 1.25–1.21 (m, 24H), 0.95 (t, *J* = 0.72 Hz, 12H); **^{13}C NMR** (100 MHz, $CDCl_3$) δ 171.43, 72.79, 69.25, 58.48, 23.87, 21.69, 21.69, 19.59, 13.58; **IR** (solid) ν 3143, 2962, 2875, 1744, 1468, 1375, 1265, 1217, 1197, 1150, 1096 cm^{-1}; **X-ray** (single crystal) Colorless block crystals of X-ray diffraction quality were obtained by carefully layering hexanes onto a saturated solution of **2e** in THF, which had previously been prepared by dissolving **2e** in refluxing THF (approximately 66 °C) until visibly saturated, filtering while hot, and then allowing to stand for approximately 1 min to cool to near room temperature. The crystal was found to be an *n*-hexane solvate. Experimental details are provided below.

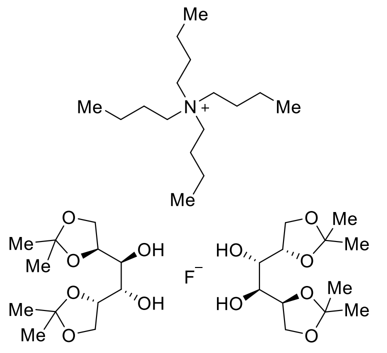

TBAF(1,2:5,6-Di-*O*-isopropylidene-D-mannitol)$_2$, **XX**

Tetra-*N*-butylammonium fluoride bis(1,2:5,6-Di-*O*-isopropylidene-D-mannitol) (TBAF(1,2:5,6-Di-*O*-isopropylidene-D-mannitol)$_2$, 2f): The title compound was prepared according to the general procedure with 1.0 mmol of TBAF(H_2O)$_3$ and 2.0 mmol of 1,2:5,6-Di-*O*-isopropylidene-D-mannitol. After heating at reflux for 2 h, a viscous colorless oil was observed at the bottom of the reaction vessel. The reaction was allowed to cool to room temperature, and the solvent was removed *in vacuo*. After further drying under high vacuum for 8 h, solidification was observed. The resulting material was warmed to room temperature and washed with hexanes (×3) on a sinter funnel, then dried under high vacuum. The complex was thus obtained as chalky white solid (795 mg, 95% yield). **^{1}H NMR** (400 MHz, $CDCl_3$) δ 4.18 (td, J_1 = 5.6 Hz, J_2 = 8.6 Hz, 4H), 4.07–3.97 (m, 8H), 3.54 (d, *J* = 8.6 Hz, 4H), 3.24–3.11 (m, 8H), 1.57 (qn, *J* = 7.6 Hz, 8H), 1.40 (sx, *J* = 7.6 Hz, 8H), 1.33 (s, 12H), 1.28 (s, 12H), 0.98 (t, *J* = 7.3 Hz, 12H); **^{13}C NMR** (100 MHz, $CDCl_3$) δ 108.67, 75.46, 71.88, 67.56, 58.27, 26.90, 25.54, 23.72, 19.56, 13.55; **IR** (solid) ν 3462, 3302, 3201, 2982, 2960, 2936, 2875, 2360, 2342, 1492, 1456, 1431, 1381, 1370, 1327, 1251, 1214, 1149, 1113, 1064, 988, 948, 919, 884, 844, 783, 738, 692, 669, 651 cm^{-1}; **X-ray** (single-crystal) Colorless block crystals of X-ray diffraction quality were obtained by carefully layering hexanes onto a saturated solution of **2f** in EtOAc, which had previously been prepared by dissolving **2f** in refluxing EtOAc (approximately 77 °C) until visibly saturated, filtering while hot, and then allowing to stand for approximately 1 min to cool to near room temperature. The crystal was found to be an EtOAc solvate. Vapor diffusion of hexanes into a saturated solution of **2f** in EtOAc, which had previously been prepared by dissolving **2f** in EtOAc at room temperature and filtering to remove residual solid, also gave crystals of suitable quality. Experimental details are provided below.

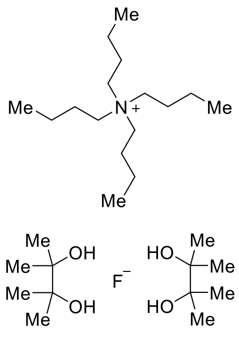

TBAF(Pin)$_2$, **2g**

Tetra-*N*-butylammonium fluoride bis(pinacol) (TBAF(Pin)$_2$, 2g): The title compound was prepared according to the general procedure with 4.0 mmol of TBAF(H_2O)$_3$ and 8.0 mmol of pinacol. The complex was thus obtained as flocculent white solid (1.86 g, 93% yield). **^{1}H NMR** (400 MHz, $CDCl_3$) δ 3.28–3.24 (m, 8H), 1.64–1.56 (m, 8H), 1.40 (sx, *J* = 7.6 Hz, 8H), 1.15 (s, 24H), 0.96 (t, *J* = 7.2 Hz, 12H); **^{13}C NMR** (100 MHz, $CDCl_3$) δ 74.46, 58.42, 25.17, 23.95, 19.64, 13.62; **IR** (solid) ν 3159, 2965, 2944, 2878, 1463, 1380, 1150, 953, 887 cm^{-1}. **X-ray** (single-crystal) Colorless needles of X-ray diffraction quality were obtained by carefully layering hexanes onto a saturated solution of **2g** in THF, which had previously been prepared by dissolving **2g** in refluxing THF (approximately 66 °C) until visibly saturated, filtering while hot, and then allowing to stand for approximately 1 min to cool to near room temperature. Experimental details are provided below.

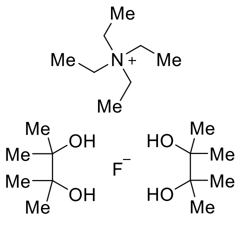

TEAF(pin)$_2$

Tetra-*N*-ethylammonium fluoride bis(pinacol) (TEAF(Pin)$_2$): The title compound was prepared according to the general procedure with 4.0 mmol of TEAF(H_2O)$_2$ and 8.0 mmol of pinacol. The complex was thus obtained as a chalky white solid (1.19 mg, 77% yield). **^{1}H NMR** (400 MHz, $CDCl_3$) δ 3.40 (q, *J* = 7.2 Hz, 8H), 1.34 (t, *J* = 7.2 Hz, 12H), 1.18 (s, 24H); **^{13}C NMR** (100 MHz, $CDCl_3$) δ 74.65, 52.39, 25.09, 7.64; **IR** (solid) ν 3170, 2974, 2939, 1490, 1379, 1149, 1002, 952, 787 cm^{-1}.

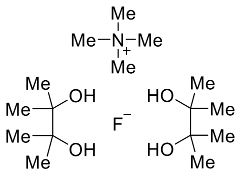

TMAF(pin)$_2$

Tetra-*N*-methylammonium fluoride bis(pinacol) (TMAF(Pin)$_2$): The title compound was prepared according to the general procedure with 4.0 mmol of TMAF(H_2O)$_4$ and 8.0 mmol of pinacol. The complex was thus obtained as a chalky white solid (964 mg, 73% yield). **^{1}H NMR** (400 MHz, $CDCl_3$) δ 3.37 (s, 12H), 1.16 (s, 24H); **^{13}C NMR** (100 MHz, $CDCl_3$) δ 74.82, 55.76, 25.12; **IR** (solid) ν 3182, 2983, 2937, 1489, 1381, 1358, 1191, 1150, 949, 762 cm^{-1}.

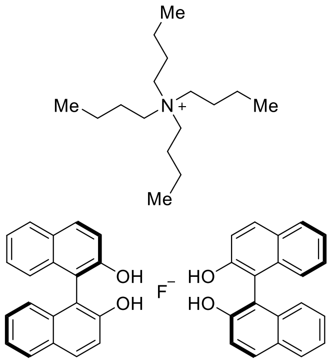

TBAF((*R*)-BINOL)$_2$, **2h**

Tetra-*N*-butylammonium fluoride bis((*R*)-(+)-1,1'-Bi(naphtol)) (TBAF(*R*-BINOL)$_2$, 2h): The title compound was prepared according to the general procedure with 1.0 mmol of TBAF(H_2O)$_3$ and 2.0 mmol of *R*-BINOL. The complex was thus obtained as a white solid (736 mg, 88% yield). **^{1}H NMR** (400 MHz, $CDCl_3$) δ 8.73 (bs, 4H), 7.80–7.73 (m, 8H), 7.52 (d, *J* = 8.8 Hz, 4H), 7.23 (ddd, J_1 = 8.1 Hz, J_2 = 6.6 Hz, J_3 = 1.5 Hz, 4H), 7.14 (ddd, J_1 = 8.6 Hz, J_2 = 6.6 Hz, J_3 = 1.2 Hz, 4H), 7.12–7.08 (m, 4H), 2.23–2.04 (m, 8H), 1.03–0.83 (m, 16H), 0.72–0.62 (m, 12H); **^{13}C NMR** (100 MHz, $CDCl_3$) δ 154.09, 134.26, 129.38, 128.52, 127.85, 125.93, 125.26, 122.42, 119.10, 114.19, 57.68, 23.04, 19.11, 13.42; **IR** (solid) ν 3052, 2959, 2930, 2872, 2732, 2656, 2360, 2341, 2164, 1857, 1620, 1582, 1505, 1480, 1459, 1432, 1400, 1339, 1302, 1276, 1244, 1213, 1179, 1145, 1129, 1071, 1024, 986, 968, 945, 934, 872, 864, 828, 821, 816, 792, 784, 774, 749, 690, 678, 667, 629 cm^{-1}. **X-ray** (single-crystal) Colorless block crystals of X-ray diffraction quality were obtained by carefully layering hexanes onto a saturated solution of **2h** in EtOAc, which had previously been prepared by dissolving **2h** in refluxing EtOAc (approximately 77 °C) until visibly saturated, filtering while hot, and then allowing to stand for approximately 1 min to cool to near room temperature. Experimental details are provided below.

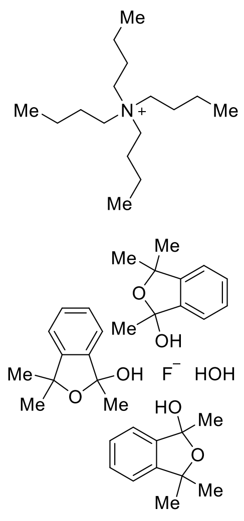

TBAF(1,3,3-trimethyl-1,3-dihydroisobenzofuran-1-ol)$_3$·(H_2O), **2i**

Tetra-*N*-butylammonium fluoride tris(1,3,3-trimethyl-1,3-dihydroisobenzofuran-1-ol) hydrate (TBAF(1,3,3-trimethyl-1,3-dihydroisobenzofuran-1-ol)$_3$•(H_2O), 2i): The title compound was prepared using the standard procedure with 0.75 mmol of TBAF(H_2O)$_3$ and 3.0 mmol of **1i**. After heating at reflux for 2 h, the reaction mixture was observed to be homogeneous and pale brown in color. The reaction was allowed to cool to room temperature, and the solvent was removed *in vacuo*. The resulting pale brown solid was washed with hexanes (×3) on a sinter funnel and dried under high vacuum, giving the complex as a flocculent pale brown solid (544 mg, 89% yield). Single-crystal X-ray diffraction of a recrystallized sample (see below) established that the complex is a monohydrate **^{1}H NMR** (400 MHz, $CDCl_3$) δ 7.37–7.32 (m, 9H), 7.15–7.12 (m 3H), 3.34–3.30 (m, 8H), 1.76 (s, 9H), 1.65 (qn, J = 7.6 Hz, 8H), 1.58 (s, 9H), 1.49 (s, 9H), 1.43 (sx, J = 7.6 Hz, 8H), 0.99 (t, J = 7.8 Hz, 12H); **^{13}C NMR** (100 MHz, $CDCl_3$) δ 146.86, 141.75, 128.76, 127.72, 122.00, 120.37, 105.74, 84.02, 58.48, 30.63, 29.22, 28.28, 23.86, 19.55, 13.56; **IR** (solid) ν 3075, 2967, 2932, 2876, 1456, 1374, .1322, 1296, 1277, 1232, 1159, 1130, 1113, 1070, 1019, 968, 943, 897, 755 cm^{-1}; **X-ray** (single-crystal) Colorless plate-like crystals of X-ray diffraction quality were obtained by vapor diffusion of hexanes into a saturated solution of **2i** in Et_2O, which had previously been prepared by dissolving **2i** in refluxing Et_2O (approximately 35 °C) until visibly saturated, filtering while hot, and then allowing to stand for approximately 1 min to cool to near room temperature.

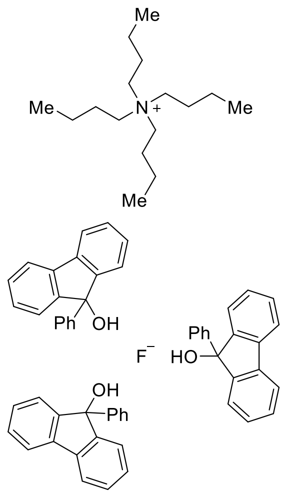

TBAF(9-phenyl-9-fluorenol)$_3$, **2j**

Tetra-*N*-butylammonium fluoride tris(9-phenyl-9-fluorenol) (TBAF(9-phenyl-9-fluorenol)$_3$, 2j): The title compound was prepared according to the general procedure with 4.0 mmol of TBAF(H_2O)$_3$ and 16.0 mmol of 9-phenyl-9-fluorenol. The complex was thus obtained as a chalky off-white solid (3.76 g, 91% yield). **^{1}H NMR** (400 MHz, $CDCl_3$) δ 7.67 (d, J = 7.6 Hz, 6H), 7.41 (d, J = 8.0 Hz, 6H), 7.36 (t, J = 7.6 Hz, 12H), 7.27–7.19 (m, 15H), 3.27–2.23 (m, 8H), 1.59–1.51 (m, 8H), 1.35 (sx, J = 7.2 Hz, 8H), 0.95 (t, J = 7.2 Hz, 12H); **^{13}C NMR** (100 MHz, $CDCl_3$) δ 150.79, 143.67, 139.55, 128.81, 128.32, 128.09, 126.99, 125.45, 124.92, 119.90, 83.43, 58.65, 24.00, 19.65, 13.65; **IR** (solid) ν 3276, 3058, 2960, 2937, 2872, 2555, 1738, 1478, 1442, 1180, 1054, 1030, 924, 889, 730, 694 cm^{-1}. **X-ray** (single-crystal) Colorless block crystals of X-ray diffraction quality were obtained by carefully layering hexanes onto a saturated solution of **2j** in EtOAc, which had previously been prepared by dissolving **2j** in refluxing EtOAc (approximately 77 °C) until visibly saturated, filtering while hot, and then allowing to stand for approximately 1 min to cool to near room temperature. Experimental details are provided below.

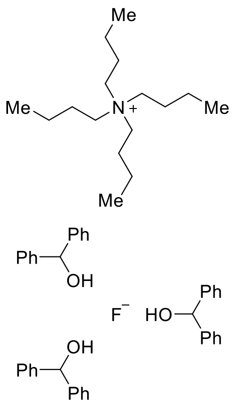

TBAF(Ph$_2$HCOH)$_3$, **2k**

Tetra-*N*-butylammonium fluoride tris(diphenylmethanol) (TBAF(Ph$_2$HCOH)$_3$ 2k): The title compound was prepared according to the general procedure with 4.0 mmol of TBAF(H$_2$O)$_3$ and 16.0 mmol of diphenylmethanol. The complex was thus obtained as a chalky off-white solid (2.96 g, 91% yield). **^{1}H NMR** (400 MHz, CDCl$_3$) δ 7.39–7.36 (m, 12H), 7.30–7.27 (m, 12H), 7.23–7.19 (m, 6H), 5.81 (s, 3H), 3.26–3.16 (m, 8H), 1.58–1.46 (m, 8H), 1.38–1.31 (m, 8H), 0.95 (t, *J* = 7.6 Hz, 12H); **^{13}C NMR** (100 MHz, CDCl$_3$) δ 128.12, 128.07, 126.74, 126.71, 75.21, 58.11, 23.94, 19.64, 13.65; **IR** (solid) ν 3024, 2961, 2873, 2693, 1490, 1453, 1181, 1052, 1027, 914, 884, 755, 737, 700 cm^{-1}. **X-ray** (single-crystal) Colorless needles of X-ray diffraction quality were obtained by carefully layering hexanes onto a saturated solution of **2k** in THF, which had previously been prepared by dissolving **2k** in refluxing THF (approximately 66 °C) until visibly saturated, filtering while hot, and then allowing to stand for approximately 1 min to cool to near room temperature. Experimental details are provided below.

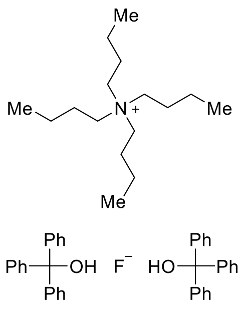

TBAF(Ph$_3$COH)$_2$, **2l**

Tetra-*N*-butylammonium fluoride bis(triphenylmethanol) (TBAF(Ph$_3$COH)$_2$, 2l): The title compound was prepared according to the general procedure with 2.0 mmol of TBAF(H$_2$O)$_3$ and 4.0 mmol of triphenylmethanol. After heating at reflux for 2 h, a viscous yellow oil was observed at the bottom of the reaction vessel, which solidified when allowed to stand at room temperature for 2 h. The reaction mixture was filtered, washed quickly with acetone and hexanes (×3), and dried under high vacuum. The complex was thus obtained as a crystalline yellow solid (954 mg, 61% yield). **^{1}H NMR** (400 MHz, CDCl$_3$) δ 7.27–7.24 (m, 30H), 3.31–3.27 (m, 8H), 1.62 (qn, *J* = 7.2 Hz, 8H), 1.40 (sx, *J* = 7.2 Hz, 8H), 0.97 (t, *J* = 7.2 Hz, 12H); **^{13}C NMR** (100 MHz, CDCl$_3$) δ 147.00, 127.86, 127.66, 126.91, 81.61, 58.54, 23.88, 19.58, 13.56; **IR** (solid) ν 3059, 2960, 2932, 2874, 2451, 1488, 1444, 1380, 1162, 1050, 1031, 884, 757, 698, 638 cm^{-1}. **X-ray** (single-crystal) A distinct sample of TBAF(Ph$_3$COH)$_n$ was synthesized using the general procedure with a 4:1 ratio of TBAF(H$_2$O)$_3$ and triphenylmethanol. After being filtered, washed with hexanes (×3), and dried under high vacuum, ^{1}H NMR analysis of this material revealed a ratio of 3.8:1. This material was used to grow single crystals of the 2:1 complex. Colorless needles of X-ray diffraction quality were obtained by carefully layering hexanes onto a saturated solution of TBAF(Ph$_3$COH)$_n$ in EtOAc, which had previously been prepared by dissolving TBAF(Ph$_3$COH)$_n$ in refluxing EtOAc (approximately 77 °C) until visibly saturated, filtering while hot, and then allowing to stand for 1 min to cool to near room temperature. The crystal was found to be an *n*-hexane solvate.

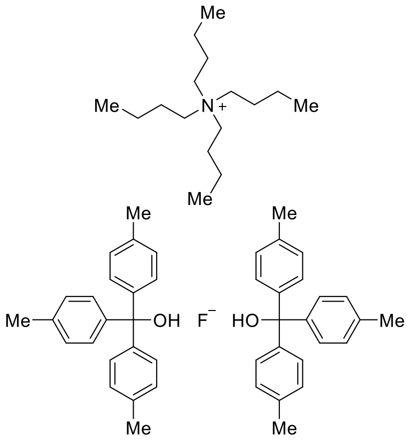

TBAF(p-Tol$_3$COH)$_2$, **2m**

Tetra-*N*-butylammonium fluoride bis(tri-*p*-tolylmethanol) (TBAF(*p*-Tol$_3$COH)$_2$, 2m): The title compound was prepared according to the general procedure with 4.0 mmol of TBAF(H_2O)$_3$ and 16.0 mmol of tri-*p*-tolylmethanol. During the course of the reaction, the solution became homogenous. After 2 h of heating at reflux, the flask was removed from the oil bath and allowed to stand at room temperature for 4 h, during which time a pale yellow precipitate was observed. Following the standard work-up, **2m** was obtained as a crystalline pale-yellow solid (2.62 g, 76% yield). **^{1}H NMR** (400 MHz, $CDCl_3$) δ 7.15 (d, *J* = 8.0 Hz, 12H), 7.09 (d, *J* = 8.0 Hz, 12H), 3.39–3.34 (m, 8H), 2.33 (s, 18H), 1.67 (qn, *J* = 7.2 Hz, 8H), 1.45 (sx, *J* = 7.2 Hz, 8H), 1.01 (t, *J* = 7.2 Hz, 12H); **^{13}C NMR** (100 MHz, $CDCl_3$) δ 144.28, 136.66, 128.49, 127.74, 81.14, 58.87, 24.09, 20.98, 19.75, 13.66; **IR** (solid) ν 2962, 2935, 2874, 2482, 1508, 1488, 1456, 1380, 1182, 1155, 1063, 916, 886, 812, 781 cm^{-1}; **X-ray** (single-crystal) Colorless needles of X-ray quality were obtained by vapor diffusion of hexanes into a saturated solution of **2m** in Et_2O, which had previously been prepared by dissolving **2m** in refluxing Et_2O (approximately 35 °C) until visibly saturated, filtering while hot, and then allowing to stand for approximately 1 min to cool to near room temperature. Experimental details are provided below.

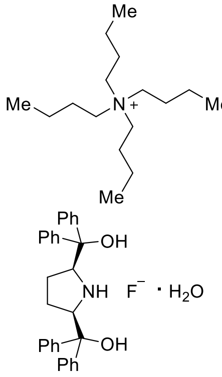

TBAF(pyrrolidine-2,5-diylbis (diphenylmethanol)·(H_2O), **2n**

Tetra-*N*-butylammonium fluoride (pyrrolidine-2,5-diyl(diphenylmethanol) hydrate (TBAF(pyrrolidine-2,5-diyl(diphenylmethanol))•(H_2O), (2n): The title compound was prepared according to the general procedure with 1.0 mmol of TBAF(H_2O)$_3$ and 1.0 mmol of **1n**. The complex was thus obtained as a white solid (608 mg, 85% yield). **^{1}H NMR** (400 MHz, $CDCl_3$) δ 7.51–7.47 (m, 4H), 7.44–7.39 (m, 4H), 7.22 (t, *J* = 7.7 Hz, 8H), 7.14–7.08 (m, 4H), 4.36 (ddd, J_1 = 7.2 Hz, J_2 = 5.3 Hz, J_3 = 2.0 Hz, 2H), 3.33–3.25 (m, 8H), 1.76–1.67 (m, 2H), 1.67–1.57 (m, 8H), 1.49–1.35 (m, 10H), 0.97 (t, *J* = 7.3 Hz, 12H); **^{13}C NMR** (100 MHz, $CDCl_3$) δ 147.05, 145.08, 128.15, 127.78, 126.53, 126.27, 125.75, 125.33, 77.26, 64.00, 58.57, 25.32, 23.89, 19.57, 13.54; **IR** (solid) ν 3289, 3086, 3058, 3022, 2959, 2933, 2874, 2581, 2361, 2341, 1653, 1598, 1489, 1467, 1448, 1379, 1350, 1317, 1263, 1181, 1172, 1149, 1106, 1065, 1052, 1033, 998, 978, 940, 885, 840, 800, 745, 697, 666, 636 cm^{-1}; **X-ray** (single-crystal) Colorless block crystals of X-ray quality were obtained by vapor diffusion of hexanes into a saturated solution of **2n** in EtOAc, which had previously been prepared by dissolving **2n** in refluxing EtOAc (approximately 77 °C) until visibly saturated, filtering while hot, and then allowing to stand for approximately 1 min to cool to near room temperature. Vapor diffusion of hexanes into a saturated solution of **2n** in EtOAc, which had previously been prepared by dissolving **2n** in EtOAc at room temperature and filtering to remove residual solid, also gave crystals of suitable quality. Experimental details are provided below.

Representative Procedure for Nucleophilic Fluorination (Preparative Scale):

To a 14 mL screw-top vial, were added **3b** (53.0 mg, 0.2 mmol), MeCN (0.8 mL), and TBAF(*t*-BuOH)$_4$ (0.8 mmol, 223.2 mg). The vial was capped and transferred to an oil bath, which had been pre-heated to 70 °C. After 1 h, the vial was removed from the oil bath and allowed to cool to room temperature. Et_2O (4 mL) and H_2O (4 mL) were added to quench the reaction. A sample of the organic phases was removed, and the solvent was removed *in vacuo*. The resulting residue was dissolved in $CDCl_3$ for analysis by ^{1}H NMR spectroscopy. The NMR sample was combined with the reaction mixture and the layers were separated. The aqueous layer was further extracted with Et_2O (2 × 5 mL). The combined organic layers were dried over Na_2SO_4, filtered, and concentrated *in vacuo*. Subsequent purification was carried out using silica gel column chromatography, using a gradient solvent system of pure hexanes → 10:1 hexanes:EtOAc. (Hexanes was used as an eluent until the olefin, **5**, eluted, and 10:1 hexanes:EtOAc was used to elute the fluoride, **4**.) The olefin byproduct and fluorinated product were obtained as pure compounds; the spectral data were in accordance with literature precedents.[20,21]

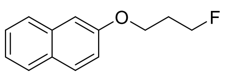

4

2-(3-Fluoropropoxy)naphthalene (4): The title compound was prepared according to the general procedure and isolated via silica gel column chromatography (hexanes) (63 mg, 63% yield). **^{1}H NMR** (400 MHz, $CDCl_3$) δ 7.78–7.71 (m, 3H), 7.44 (ddd, J_1 = 8.0 Hz, J_2 = 6.8 Hz, J_3 = 1.2 Hz, 1H), 7.34 (ddd, J_1 = 8.0 Hz, J_2 = 6.8 Hz, J_3 = 1.2 Hz, 1H), 7.15–7.13 (m, 2H), 4.70 (dt, $J_{H–F}$ = 47.2, $J_{H–H}$ = 6.0 Hz, 2H), 4.23 (t, J = 6.0 Hz, 2H), 2.25 (dqn, $J_{H–F}$ = 26.0 Hz, $J_{H–H}$ = 6.0 Hz, 2H); **^{13}C NMR** (100 MHz, $CDCl_3$) δ 156.65, 134.49, 129.39, 128.97, 127.60, 126.72, 126.35, 123.63, 118.77, 106.62, 80.77 (d, $J_{C–F}$ = 163.5 Hz), 63.46 (d, $J_{C–F}$ = 5.2 Hz), 30.35 (d, $J_{C–F}$ = 19.9 Hz); **^{19}F NMR** (376.5 MHz, $CDCl_3$) δ –222.11; **IR** (film, $CDCl_3$) ν 3058, 2969, 1629, 1601, 1510, 1465, 1390, 1258, 1216, 1181, 1097, 1052, 838, 747cm^{-1}; **HRMS** (EI/FI) *m/z* Calcd for $C_{13}H_{13}FO$ $[M]^+$ 204.0950, found 205.0957.

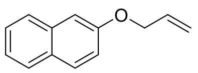

5

2-(Allyloxy)naphthalene (5): The title compound was prepared according to the general procedure and isolated via silica gel column chromatography (hexanes) (27 mg, 29% yield). **^{1}H NMR** (400 MHz, $CDCl_3$) δ 7.78–7.71 (m, 3H), 7.43 (td, J_1 = 8.0 Hz, J_2 = 1.2 Hz, 1H), 7.33 (td, J_1 = 8.0 Hz, J_2 = 1.2 Hz, 1H), 7.18 (dd, J_1 = 8.8 Hz, J_2 = 2.8 Hz, 1H), 7.15–7.14 (m, 1H), 6.14 (ddt, J_1 = 17.4 Hz, J_2 = 10.6 Hz, J_3 = 5.6 Hz, 1H), 5.51–5.45 (m, 1H), 5.35–5.31 (m, 1H), 4.66 (dt, J = 5.6 Hz, J_2 = 1.2 Hz, 2H); **^{13}C NMR** (100 MHz, $CDCl_3$) δ 156.51, 134.48, 133.17, 129.40, 129.00, 127.63, 126.74, 126.33, 123.64, 118.96, 117.79, 106.98, 68.81; **IR** (film, $CDCl_3$) ν 3058, 1630, 1600, 1258, 1217, 1182, 1017, 837, 747 cm^{-1}; **HRMS** (EI/FI) *m/z* Calcd for $C_{13}H_{12}O$ $[M]^+$ 184.0888, found 184.0891.

Crystal Structures

Table S1 gives as summary of all measured structures.

Table S1 Overview of crystal structures of complexes **2a–2n** (continued on the following pages).

**Nr.**	**2a**	**2b**	**2c**	**2d**	**2e**	**2f**	**2g**
**Abbreviation**	TBAF(1-Ada-OH)$_4$	TBAF(PERT)	TBAF(TME)$_2$	TBAF(NPG)$_2$	TBAF((+)-DIPT)$_2$	TBAF(1,2:5,6-Di-*O*-isopropylidene-D-mannitol)$_2$	TBAF(pin)$_2$
**Formula**	$C_{56}H_{100}F_1N_1O_4$	$C_{22}H_{46}Cl_2F_1N_1O_4$	$C_{26}H_{60}F_1N_1O_6$	$C_{26}H_{60}F_1N_1O_4$	$C_{38.39}H_{77.58}F_1N_1O_{12}$	$C_{40}H_{80}F_1N_1O_{12}$	$C_{28}H_{64}F_1N_1O_4$
**Molecular Weight**	870.41	482.55	501.76	469.76	764.3	437.09	497.82
**Crystal System**	tetragonal	monoclinic	orthorhombic	monoclinic	orthorhombic	monoclinic	orthorhombic
**T [K]**	150	150	150	150	150	150	150
**Space Group**	P -4	P 21/c	P b c n	C 1 2/c 1	P 21 21 2	C 2	P b c a
**a [Å]**	15.6111(1)	9.9446(3)	9.9133(1)	22.5166(1)	18.9780(2)	25.1923(1)	18.6017(3)
**b [Å]**	15.6111(1)	18.0891(7)	17.3454(3)	14.2863(1)	24.2645(2)	9.6565(1)	18.6158(3)
**c [Å]**	10.5050(1)	15.7361(6)	18.3631(3)	19.8783(1)	10.3218(1)	10.6715(1)	19.1084(3)
**α [°]**	90	90	90	90	90	90	90
**β [°]**	90	103.523(4)	90	103.6255(5)	90	92.246(1)	90
**γ [°]**	90	90	90	90	90	90	90
**V [Å^3]**	2560.14(3)	2752.27(18)	3157.54(8)	6214.47(6)	4753.10(8)	2594.1(1)	6616.96(18)
**Z**	4	4	8	8	4	2	8
**D_{calc} [g cm^{-3}]**	1.129	1.164	1.055	1.004	1.068	1.119	0.999
**F(0 0 0)**	968	1056	1120	2112	1679.681	960	2240
**μ [mm^{-1}]**	0.546	2.380	0.076	0.549	0.080	0.689	0.068
**Crystal Size [mm]**	0.20 * 0.21 * 0.23	0.04 * 0.15 * 0.18	0.10 * 0.20 * 0.35	0.18 * 0.20 * 0.28	0.10 * 0.10 * 0.43	0.15 * 0.15 * 0.20	0.08 * 0.10 * 0.49
**Colour, Shape**	Clear colourless, block	Clear colourless, plate	Clear colourless, plate	Clear colourless, block	Clear colourless, prism	Clear colourless, block	Clear colourless, prism
**R_{int}**	5.82	6.93	6.23	5.82	6.75	3.27	5.58
**$θ_{min}$, $θ_{max}$ [deg]**	2.831 - 75.425	3.784 - 72.237	5.200 - 26.942	3.695 - 75.176	5.098 - 26.381	3.511 - 76.028	5.115 - 26.020
**Total Reflections (before merge)**	5329	19286	46870	66092	65091	49863	96568
**Data (I>-3.0/sigma(I)) [Reflections, Restraints, Parameters]**	3520, 1255, 406	4548, 32, 293	3597, 0, 155	5509, 0, 289	10798, 201, 525	5353, 623, 321	6466, 0, 308
**S (=GooF)**	1.011	0.986	0.962	0.994	0.981	1.004	0.986
**Min. and Max. Residual Dens., [e/Å3]**	-0.26	-0.47	-0.25	-0.24	-0.25	-0.20	-0.14
	0.29	0.60	0.34	0.49	0.30	0.26	0.20
**Threshold Expression**	I>2sigma(I)	I>2sigma(I)	I>2sigma(I)	I>2sigma(I)	I>2sigma(I)	I>2sigma(I)	I>2sigma(I)
**R_1**	0.0574	0.0572	0.0431	0.0556	0.0448	0.0326	0.0427
**wR_2**	0.1551	0.1408	0.0972	0.1490	0.0884	0.0889	0.0979

Nr.	2h	2i	2j	2k	2l	2m	2n
**Abbreviation**	TBAF(*R*-BINOL)$_2$	TBAF(1,3,3-trimethyl-1,3-dihydroisobenzofuran-1-ol)$_3$·H_2O	TBAF(9-phenyl-9-fluorenol)$_3$	TBAF(Ph_2HCOH)$_3$	TBAF(Ph_3COH)$_2$	TBAF(*p*-Tol$_3$COH)2	TBAF(pyrrolidine-2,5-diyl(diphenylmethanol))·H_2O
**Formula**	$C_{56}H_{64}F_1N_1O_4$	$C_{49}H_{80}F_1N_1O_7$	$C_{73}H_{73}F_1N_1O_3$	$C_{55}H_{72}F_1N_1O_3$	$C_{54}H_{68}F_1N_1O_2$	$C_{60}H_{80}F_1N_1O_2$	$C_{38}H_{48}F_{0.5}N_{1.5}O_{2.5}$
**Molecular Weight**	834.13	814.17	1036.43	814.18	782.14	866.3	575.31
**Crystal System**	orthorhombic	monoclinic	triclinic	triclinic	monoclinic	triclinic	monoclinic
**T [K]**	150	150	150	150	150	150	150
**Space Group**	P 21 21 21	P c	P -1	P -1	P 21/n	P -1	C 2/c
**a [Å]**	10.68690(10)	8.75510(1)	14.5683(1)	9.4953(1)	11.2887(1)	13.4717(3)	27.7157(6)
**b [Å]**	12.65280(10)	12.74630(1)	19.9837(2)	12.1501(2)	17.6025(1)	17.5643(4)	9.7374(2)
**c [Å]**	34.7970(3)	22.1115(2)	20.7534(2)	21.4618(4)	24.9660(2)	24.8506(5)	25.2072(5)
**α [°]**	90	90	91.2454(7)	81.8444(6)	90	78.8655(19)	90
**β [°]**	90	92.0698(6)	94.4191(8)	89.1604(6)	98.4318(7)	80.5422(17)	105.664(2)
**γ [°]**	90	90	103.9009(9)	78.5486(7)	90	69.645(2)	90
**V [Å^3]**	4704.78(7)	2465.93(4)	5842.46(9)	2401.96(7)	4907.35(7)	5379.0(2)	6550.2(2)
**Z**	4	2	2	2	4	2	8
**D_{calc} [g cm^{-3}]**	1.178	1.096	1.178	1.126	1.059	1.070	1.167
**F(0 0 0)**	1792	892	2224	884	1696	1888	2488
**μ [mm^{-1}]**	0.592	0.589	0.561	0.070	0.504	0.500	0.576
**Crystal Size [mm]**	0.18 * 0.20 * 0.20	0.16 * 0.17 * 0.19	0.21 * 0.24 * 0.26	0.14 * 0.18 * 0.65	0.22 * 0.24 * 0.30	0.18 * 0.20 * 0.21	0.20 * 0.20 * 0.24
**Colour, Shape**	Clear colourless, block	Clear colourless, block	Clear colourless, block	Clear colourless, block	Clear colourless, block	Clear colourless, block	Clear colourless, block
**R_{int}**	2.74	3.00	4.29	6.11	6.14	5.02	4.99
**θ_{min}, θ_{max} [deg]**	3.717 - 76.149	3.467 - 75.196	3.060 - 75.122	5.114 - 25.087	3.083 - 76.917	3.038 - 74.851	3.312 - 74.463
**Total Reflections (before merge)**	52468	53899	227665	40263	117478	110583	26899
**Data (I>-3.0/sigma(I)) [Reflections, Restraints, Parameters]**	9732, 0, 560	9828, 2, 544	20878, 0, 1406	8998, 256, 597	10304, 1269, 836	22370, 0, 1154	6762, 950, 443
**S (=GooF)**	0.964	0.998	0.986	0.997	0.982	0.991	0.994
**Min. and Max. Residual Dens., [e/Å3]**	-0.14	-0.16	-0.30	-0.35	-0.60	-0.21	-0.17
	0.15	0.33	0.52	0.53	0.69	0.33	0.94
**Threshold Expression**	I>2sigma(I)	I>2sigma(I)	I>2sigma(I)	I>2sigma(I)	I>2sigma(I)	I>2sigma(I)	I>2sigma(I)
**R_1**	0.0263	0.0296	0.0408	0.0480	0.0553	0.0439	0.0469
**wR_2**	0.0662	0.0793	0.0976	0.1166	0.1394	0.1141	0.1322

Reaction Kinetics

Rate profile measurements with [tetrabutylammonium fluoride–alcohol] complexes:

To a 14 mL screw-top vial, were added **3b** (53.0 mg, 0.2 mmol), MeCN (0.8 mL) and the [tetrabutylammonium fluoride–alcohol] complex (0.4 mmol). The vial was capped with a rubber septum, then stirred at 70 °C for 2 h. At the indicated time points, a small aliquot (< 80 µL) was taken with a syringe and needle. Each aliquot was filtered through a short plug of silica gel to quench the reaction, and the silica gel was washed with EtOAc (3 × 2 mL). The combined rinsings were concentrated *in vacuo*, and the resulting residue was analyzed by ^{1}H NMR. The values in the kinetics tables below were determined by dividing the integral corresponding to each of the methylene/allylic proton signals depicted in Figure S1 by the sum of all integrals corresponding to methylene/allylic proton signals α to oxygen. The end-point % compositions in the summary tables were determined by dividing the integral corresponding to each of the methylene/allylic proton signals depicted in Figure S1 by the sum total of integrals corresponding to these same peaks. The latter calculation inherently gives values that add to 100%, while the former does not. The former provides better standardization from one time point to the next. The procedure was repeated three times for each complex. The resulting data were plotted, and linear regression of the first four or five time points established the initial rate. The results are summarized in Table S2. Details for individual complexes are shown in Tables S3–S9 and Figures S4–S18.

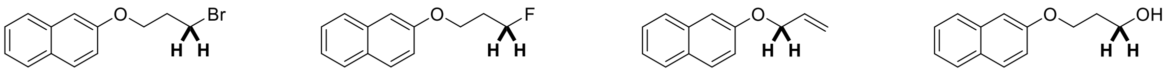

3b 3.66 (t, J = 6.8 Hz, 2H) | **4** 4.70 (dt, $J_{H\text{-}F}$ = 47.2, $J_{H\text{-}H}$ = 6.0 Hz, 2H) | **5** 4.66 (dt, J_1 = 5.6, J_2 = 1.2 Hz, 2H) | 3.92 (t, J = 6.8 Hz, 2H)

Figure S1: Summary of key peaks for integration. The chemical shifts shown here are based on pure, isolated samples of these compounds. The corresponding alkoxylated products were generally not observed in measurable amounts. In the reaction mixture, the signal for the alcohol product can appear as a broad triplet or broad quartet, depending on the concentration and pH of the solution.

Table S2: Summary of kinetic data and reactivity studies.[a]

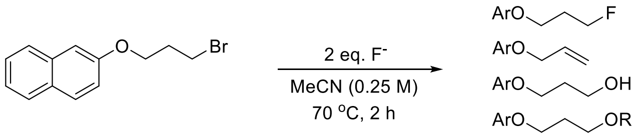

		Reaction Composition after 2 h (%, ^{1}H NMR)				
Entry	Fluoride Complex	Fluoride	Olefin	Alcohol	Ether	Bromide
1	TBAF(p-Tol$_3$COH)$_2$	67	33	0	0	0
2	TBAF(t-BuOH)$_4$	66(63)	34(29)	0	0	0
3	TBAF(9-phenyl-9-fluorenol)$_3$	77	22	0	0	1
4	TBAF(H$_2$O)$_3$	59	33	8	0	0
5	TBAF(Pin)$_2$	67	23	0	0	10
6	TBAF(NPG)$_2$	45	14	0	0	41
7	TBAF(TME)$_2$	32	8	0	0	60
8	TBAF(anhydrous)	22	78	0	0	0

[a] The % composition was determined by ^{1}H NMR analysis of the crude reaction mixture. Values represent average of three independent trials. Isolated yields from a fourth independent run (0.5 mmol scale) shown in parentheses. [b] See Ref. (16): Reaction conditions: CD$_3$CN (0.25 M), 25 °C, 1 h. Anhydrous TBAF generated from tetrabutylammonium cyanide and hexafluorobenzene using the procedure described by DiMagno et al.[22]

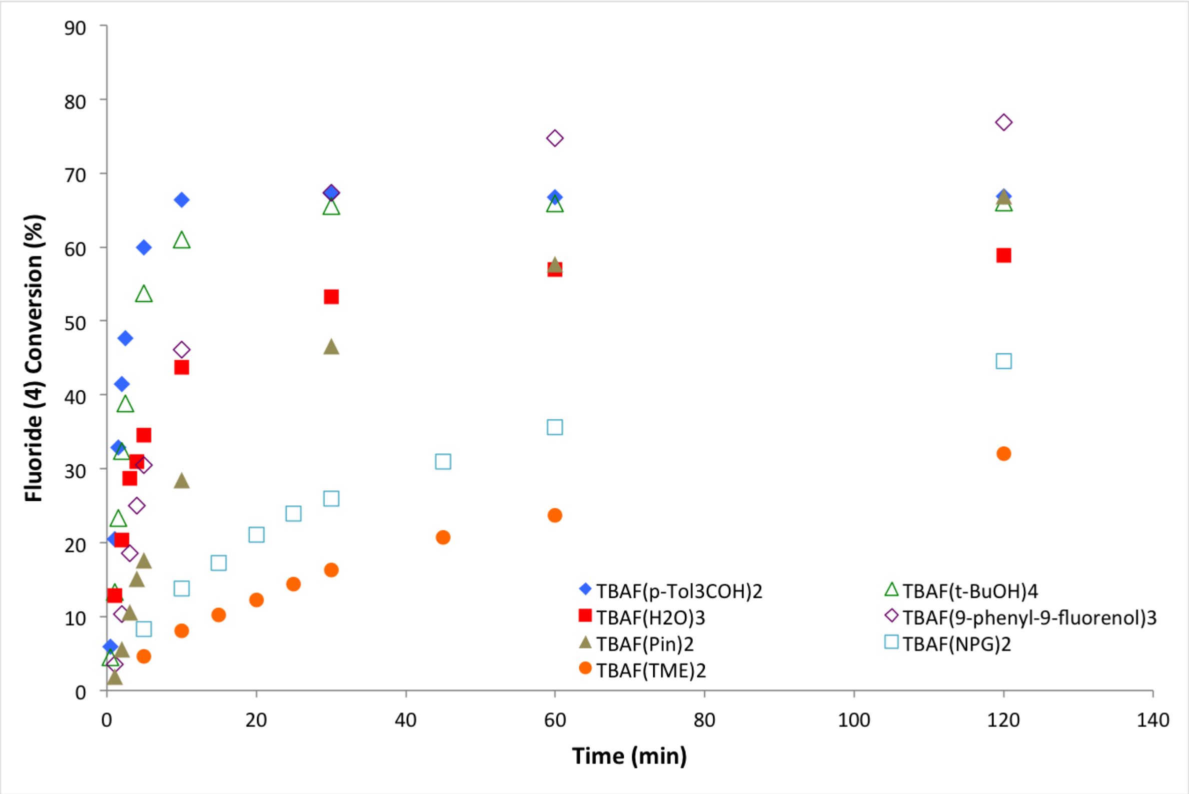

Figure S2: Summary of rate profiles for fluoride (**4**) product formation for all [fluoride–alcohol] complexes examined.

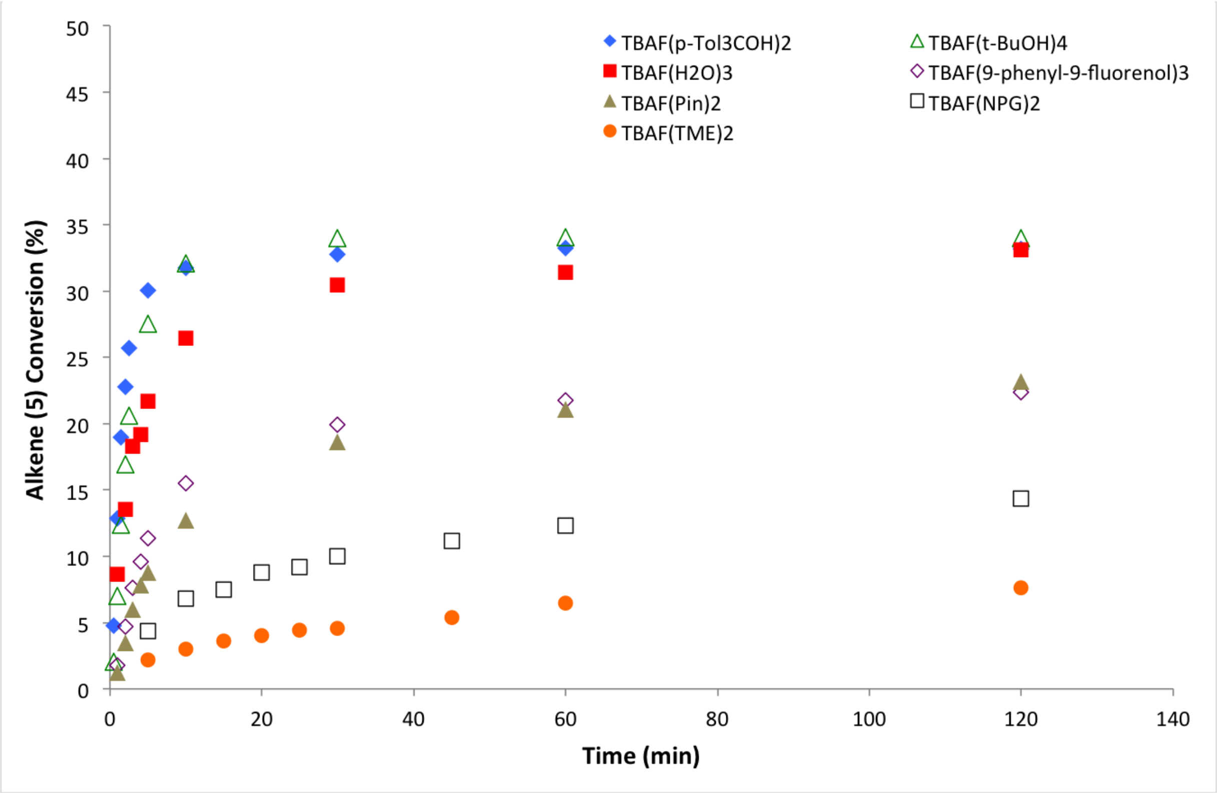

Figure S3: Summary of rate profiles for olefin (**5**) product formation for all [fluoride–alcohol] complexes examined.

Table S3: Kinetic measurements using TBAF(NPG)$_2$ (**2d**).[a]

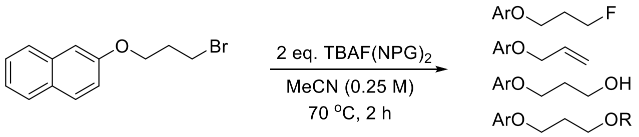

		Composition (%), Trial 1					*Composition (%), Trial 2*					*Composition (%), Trial 3*					Fluoride (%)		Alkene (%)		Alcohol (%)		Ether (%)		Bromide (%)	
Entry	t (sec)	Fluoride	Alkene	Alcohol	Ether	Bromide	Fluoride	Alkene	Alcohol	Ether	Bromide	Fluoride	Alkene	Alcohol	Ether	Bromide	*Ave.*	*Std. Dev.*	*Ave.*	*Std. Dev.*	*Ave.*	*Std. Dev.*	*Ave.*	*Std. Dev.*	*Ave.*	*Std. Dev.*
1	300	9.3	4.8	0.0	0.0	87.5	10.4	5.3	0.0	0.0	83.4	7.6	4.3	0.0	0.0	88.7	9.1	1.4	4.8	0.5	0.0	0.0	0.0	0.0	86.5	2.8
2	600	13.0	6.1	0.0	0.0	87.2	16.2	7.7	0.0	0.0	81.7	13.4	6.5	0.0	0.0	80.4	14.2	1.7	6.8	0.8	0.0	0.0	0.0	0.0	83.1	3.6
3	900	18.1	7.8	0.0	0.0	76.1	19.5	8.4	0.0	0.0	73.3	18.7	8.6	0.0	0.0	80.2	18.8	0.7	8.3	0.4	0.0	0.0	0.0	0.0	76.5	3.5
4	1200	15.6	6.4	0.0	0.0	58.7	23.2	9.7	0.0	0.0	66.5	21.6	9.3	0.0	0.0	74.6	20.1	4.0	8.5	1.8	0.0	0.0	0.0	0.0	66.6	8.0
5	1500	25.6	9.8	0.0	0.0	72.0	24.6	10.0	0.0	0.0	69.1	24.5	10.3	0.0	0.0	68.5	24.9	0.6	10.0	0.2	0.0	0.0	0.0	0.0	69.8	1.9
6	1800	26.2	10.1	0.0	0.0	63.7	26.9	10.5	0.0	0.0	62.8	27.7	11.2	0.0	0.0	68.0	26.9	0.7	10.6	0.6	0.0	0.0	0.0	0.0	64.8	2.8
7	2700	32.5	11.1	0.0	0.0	57.0	32.0	11.6	0.0	0.0	54.5	32.5	12.3	0.0	0.0	58.7	32.3	0.3	11.7	0.6	0.0	0.0	0.0	0.0	56.7	2.1
8	3600	37.0	12.8	0.0	0.0	55.0	36.7	12.8	0.0	0.0	52.9	36.9	13.4	0.0	0.0	50.4	36.9	0.2	13.0	0.4	0.0	0.0	0.0	0.0	52.8	2.3
9	7200	50.2	15.7	0.0	0.0	50.1	45.0	14.6	0.0	0.0	38.6	46.7	16.0	0.0	0.0	41.1	47.3	2.6	15.5	0.7	0.0	0.0	0.0	0.0	43.3	6.0

		Concentration (M), Trial 1					*Concentration (M), Trial 2*					*Concentration (M), Trial 3*					Fluoride (M)		Alkene (M)		Alcohol (M)		Ether (M)		Bromide (M)	
Entry	t (sec)	Fluoride	Alkene	Alcohol	Ether	Bromide	Fluoride	Alkene	Alcohol	Ether	Bromide	Fluoride	Alkene	Alcohol	Ether	Bromide	*Ave.*	*Std. Dev.*	*Ave.*	*Std. Dev.*	*Ave.*	*Std. Dev.*	*Ave.*	*Std. Dev.*	*Ave.*	*Std. Dev.*
1	300	0.023	0.012	0.000	0.000	0.219	0.026	0.013	0.000	0.000	0.208	0.019	0.011	0.000	0.000	0.222	0.023	0.003	0.012	0.001	0.000	0.000	0.000	0.000	0.216	0.007
2	600	0.033	0.015	0.000	0.000	0.218	0.040	0.019	0.000	0.000	0.204	0.033	0.016	0.000	0.000	0.201	0.036	0.004	0.017	0.002	0.000	0.000	0.000	0.000	0.208	0.009
3	900	0.045	0.020	0.000	0.000	0.190	0.049	0.021	0.000	0.000	0.183	0.047	0.021	0.000	0.000	0.201	0.047	0.002	0.021	0.001	0.000	0.000	0.000	0.000	0.191	0.009
4	1200	0.039	0.016	0.000	0.000	0.147	0.058	0.024	0.000	0.000	0.166	0.054	0.023	0.000	0.000	0.186	0.050	0.010	0.021	0.005	0.000	0.000	0.000	0.000	0.166	0.020
5	1500	0.064	0.025	0.000	0.000	0.180	0.061	0.025	0.000	0.000	0.173	0.061	0.026	0.000	0.000	0.171	0.062	0.002	0.025	0.001	0.000	0.000	0.000	0.000	0.175	0.005
6	1800	0.066	0.025	0.000	0.000	0.159	0.067	0.026	0.000	0.000	0.157	0.069	0.028	0.000	0.000	0.170	0.067	0.002	0.027	0.001	0.000	0.000	0.000	0.000	0.162	0.007
7	2700	0.081	0.028	0.000	0.000	0.142	0.080	0.029	0.000	0.000	0.136	0.081	0.031	0.000	0.000	0.147	0.081	0.001	0.029	0.001	0.000	0.000	0.000	0.000	0.142	0.005
8	3600	0.092	0.032	0.000	0.000	0.138	0.092	0.032	0.000	0.000	0.132	0.092	0.034	0.000	0.000	0.126	0.092	0.000	0.032	0.001	0.000	0.000	0.000	0.000	0.132	0.006
9	7200	0.125	0.039	0.000	0.000	0.125	0.113	0.037	0.000	0.000	0.097	0.117	0.040	0.000	0.000	0.103	0.118	0.007	0.039	0.002	0.000	0.000	0.000	0.000	0.108	0.015

[a] The % composition was determined by ^{1}H NMR analysis of the crude reaction mixture.

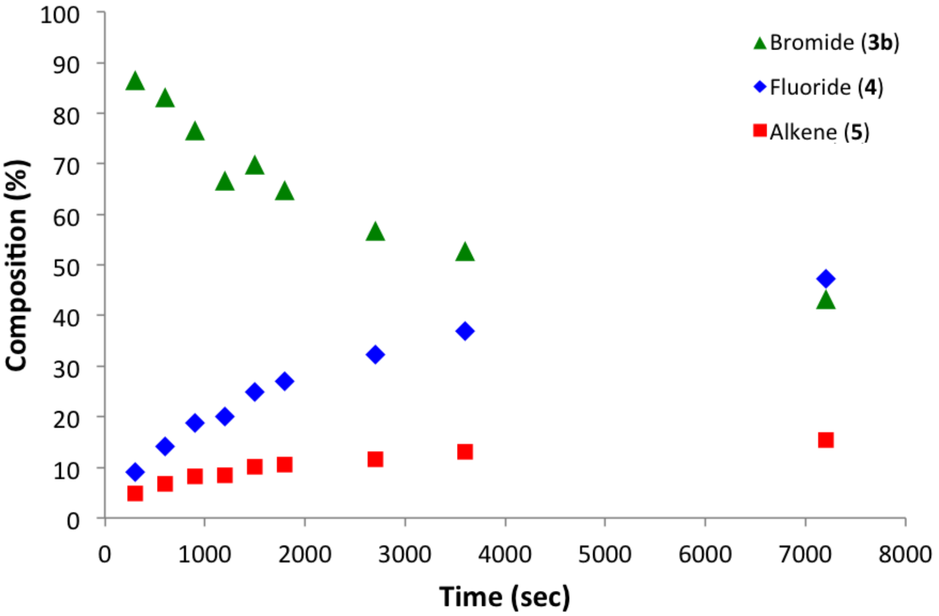

Figure S4: Rate profile with TBAF(NPG)$_2$ (**2d**) Each data point represents the average value of three independent trials.

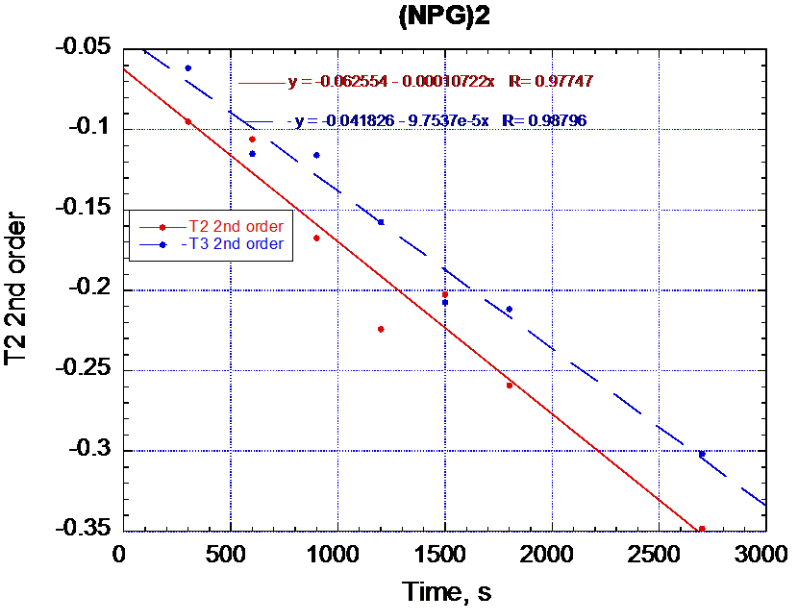

Figure S5: 2nd order plot for the reaction with TBAF(NPG)$_2$.

Table S4: Kinetic measurements using TBAF(TME)$_2$ (**2c**).[a]

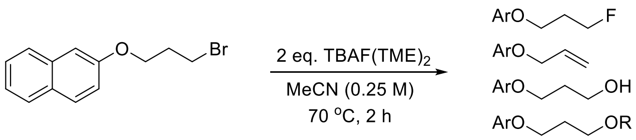

		Composition (%), Trial 1					*Composition (%), Trial 2*					*Composition (%), Trial 3*					Fluoride (%)		Alkene (%)		Alcohol (%)		Ether (%)		Bromide (%)	
Entry	t (sec)	Fluoride	Alkene	Alcohol	Ether	Bromide	Fluoride	Alkene	Alcohol	Ether	Bromide	Fluoride	Alkene	Alcohol	Ether	Bromide	*Ave.*	*Std. Dev.*	*Ave.*	*Std. Dev.*	*Ave.*	*Std. Dev.*	*Ave.*	*Std. Dev.*	*Ave.*	*Std. Dev.*
1	300	3.9	1.7	0.0	0.0	99.0	3.8	2.1	0.0	0.0	97.5	2.3	1.3	0.0	0.0	99.4	3.4	0.9	1.7	0.4	0.0	0.0	0.0	0.0	98.6	1.0
2	600	6.8	2.5	0.0	0.0	96.0	4.5	1.8	0.0	0.0	97.9	4.9	2.0	0.0	0.0	96.9	5.4	1.2	2.1	0.4	0.0	0.0	0.0	0.0	97.0	1.0
3	900	8.6	2.9	0.0	0.0	93.4	8.3	2.8	0.0	0.0	92.6	6.5	2.0	0.0	0.0	92.3	7.8	1.2	2.6	0.5	0.0	0.0	0.0	0.0	92.8	0.6
4	1200	10.8	3.2	0.0	0.0	88.7	11.2	3.5	0.0	0.0	89.5	9.1	2.7	0.0	0.0	90.9	10.4	1.1	3.1	0.4	0.0	0.0	0.0	0.0	89.7	1.1
5	1500	12.9	3.5	0.0	0.0	87.5	13.5	4.0	0.0	0.0	88.2	12.1	3.4	0.0	0.0	85.7	12.8	0.7	3.6	0.3	0.0	0.0	0.0	0.0	87.1	1.3
6	1800	16.2	4.5	0.0	0.0	83.6	15.4	4.6	0.0	0.0	85.8	13.9	3.9	0.0	0.0	82.8	15.2	1.2	4.3	0.4	0.0	0.0	0.0	0.0	84.1	1.5
7	2700	20.3	4.9	0.0	0.0	81.3	20.7	5.3	0.0	0.0	82.4	18.8	4.9	0.0	0.0	79.3	19.9	1.0	5.0	0.2	0.0	0.0	0.0	0.0	81.0	1.6
8	3600	24.8	5.9	0.0	0.0	76.5	23.7	6.1	0.0	0.0	76.4	31.9	7.3	0.0	0.0	69.6	26.8	4.4	6.4	0.8	0.0	0.0	0.0	0.0	74.2	4.0
9	7200	36.7	8.2	0.0	0.0	69.2	36.2	8.5	0.0	0.0	67.6	21.9	5.3	0.0	0.0	79.6	31.6	8.4	7.4	1.8	0.0	0.0	0.0	0.0	72.2	6.5

		Concentration (M), Trial 1					*Concentration (M), Trial 2*					*Concentration (M), Trial 3*					Fluoride (M)		Alkene (M)		Alcohol (M)		Ether (M)		Bromide (M)	
Entry	t (sec)	Fluoride	Alkene	Alcohol	Ether	Bromide	Fluoride	Alkene	Alcohol	Ether	Bromide	Fluoride	Alkene	Alcohol	Ether	Bromide	*Ave.*	*Std. Dev.*	*Ave.*	*Std. Dev.*	*Ave.*	*Std. Dev.*	*Ave.*	*Std. Dev.*	*Ave.*	*Std. Dev.*
1	18000	0.010	0.004	0.000	0.000	0.247	0.010	0.005	0.000	0.000	0.244	0.006	0.003	0.000	0.000	0.249	0.008	0.002	0.004	0.001	0.000	0.000	0.000	0.000	0.247	0.003
2	36000	0.017	0.006	0.000	0.000	0.240	0.011	0.004	0.000	0.000	0.245	0.012	0.005	0.000	0.000	0.242	0.013	0.003	0.005	0.001	0.000	0.000	0.000	0.000	0.242	0.002
3	54000	0.022	0.007	0.000	0.000	0.234	0.021	0.007	0.000	0.000	0.231	0.016	0.005	0.000	0.000	0.231	0.019	0.003	0.006	0.001	0.000	0.000	0.000	0.000	0.232	0.001
4	72000	0.027	0.008	0.000	0.000	0.222	0.028	0.009	0.000	0.000	0.224	0.023	0.007	0.000	0.000	0.227	0.026	0.003	0.008	0.001	0.000	0.000	0.000	0.000	0.224	0.003
5	90000	0.032	0.009	0.000	0.000	0.219	0.034	0.010	0.000	0.000	0.220	0.030	0.009	0.000	0.000	0.214	0.032	0.002	0.009	0.001	0.000	0.000	0.000	0.000	0.218	0.003
6	108000	0.041	0.011	0.000	0.000	0.209	0.038	0.011	0.000	0.000	0.215	0.035	0.010	0.000	0.000	0.207	0.038	0.003	0.011	0.001	0.000	0.000	0.000	0.000	0.210	0.004
7	162000	0.051	0.012	0.000	0.000	0.203	0.052	0.013	0.000	0.000	0.206	0.047	0.012	0.000	0.000	0.198	0.050	0.002	0.013	0.001	0.000	0.000	0.000	0.000	0.202	0.004
8	216000	0.062	0.015	0.000	0.000	0.191	0.059	0.015	0.000	0.000	0.191	0.080	0.018	0.000	0.000	0.174	0.067	0.011	0.016	0.002	0.000	0.000	0.000	0.000	0.185	0.010
9	432000	0.092	0.020	0.000	0.000	0.173	0.090	0.021	0.000	0.000	0.169	0.055	0.013	0.000	0.000	0.199	0.079	0.021	0.018	0.004	0.000	0.000	0.000	0.000	0.180	0.016

[a] The % composition was determined by ^1H NMR analysis of the crude reaction mixture.

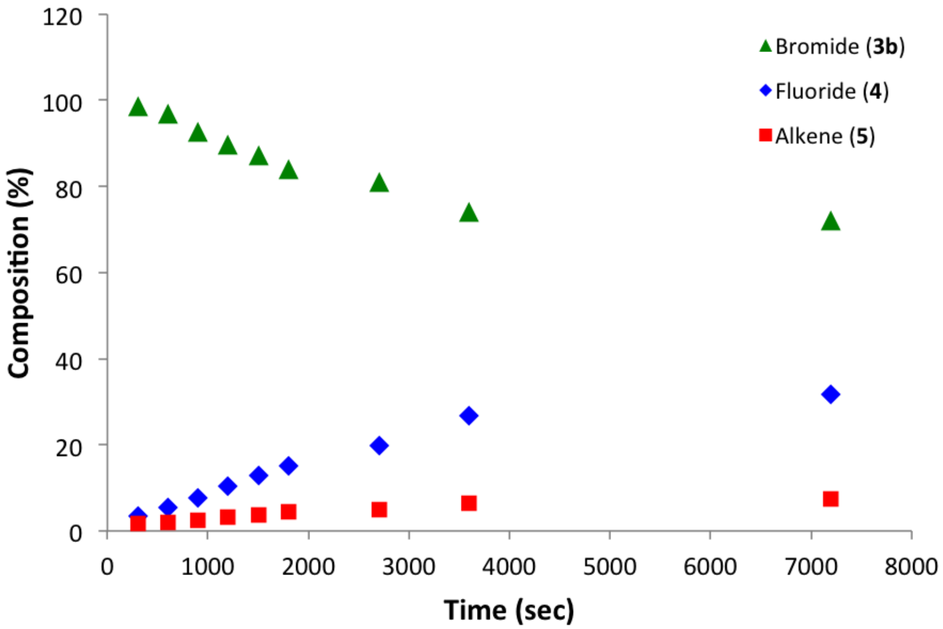

Figure S6: Rate profile with TBAF(TME)$_2$ (**2c**). Each data point represents the average value of three independent trials.

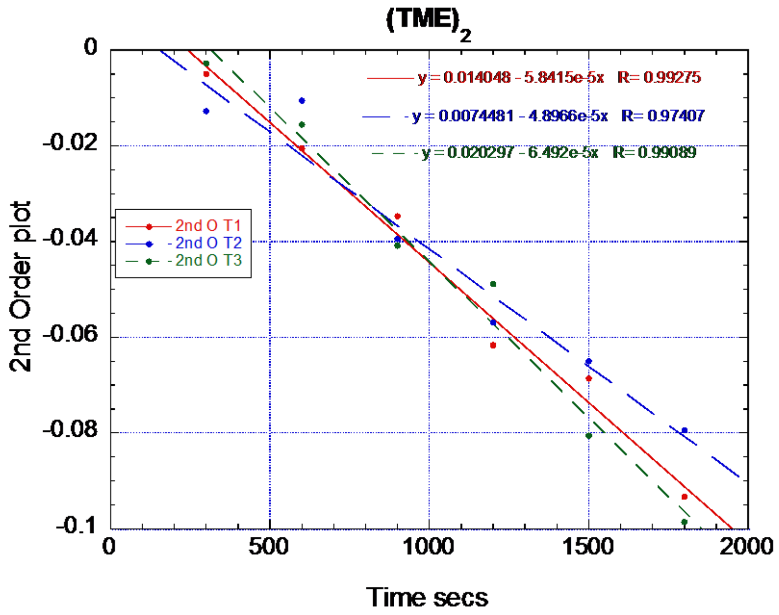

Figure S7: 2nd order plot for the reaction with TBAF(TME)$_2$.

Table S5: Kinetic measurements using TBAF(Pin)$_2$ (**2g**)[a]

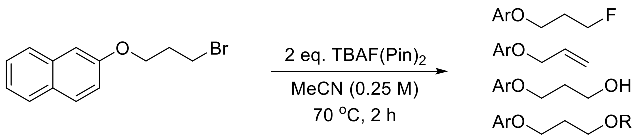

		Composition (%), Trial 1					*Composition (%), Trial 2*					*Composition (%), Trial 3*				
Entry	t (sec)	Fluoride	Alkene	Alcohol	Ether	Bromide	Fluoride	Alkene	Alcohol	Ether	Bromide	Fluoride	Alkene	Alcohol	Ether	Bromide
1	60	2.4	1.7	0.0	0.0	92.5	0.8	1.0	0.0	0.0	92.9	2.2	1.8	0.0	0.0	91.2
2	120	5.2	3.1	0.0	0.0	86.1	6.4	3.9	0.0	0.0	85.0	6.7	4.0	0.0	0.0	84.2
3	180	10.1	5.7	0.0	0.0	79.9	11.2	6.2	0.0	0.0	77.9	12.5	6.8	0.0	0.0	77.1
4	240	16.1	8.2	0.0	0.0	74.8	14.6	7.8	0.0	0.0	73.8	15.9	8.5	0.0	0.0	70.9
5	300	17.5	8.9	0.0	0.0	69.7	17.6	8.7	0.0	0.0	69.9	18.2	9.3	0.0	0.0	67.5
6	600	26.6	12.1	0.0	0.0	57.4	27.1	12.2	0.0	0.0	56.2	28.9	13.0	0.0	0.0	53.6
7	1800	43.8	17.3	0.0	0.0	33.6	43.2	17.0	0.0	0.0	33.2	47.4	18.8	0.0	0.0	29.8
8	3600	54.8	20.5	0.0	0.0	19.4	54.2	19.8	0.0	0.0	21.9	56.3	21.1	1.1	0.0	17.7
9	7200	58.8	22.7	0.0	0.0	9.3	62.7	21.7	0.0	0.0	10.8	65.0	23.1	0.0	0.0	7.8

		Fluoride (%)		Alkene (%)		Alcohol (%)		Ether (%)		Bromide (%)	
Entry	t (sec)	*Ave.*	*Std. Dev.*	*Ave.*	*Std. Dev.*	*Ave.*	*Std. Dev.*	*Ave.*	*Std. Dev.*	*Ave.*	*Std. Dev.*
1	60	1.8	0.9	1.5	0.4	0.0	0.0	0.0	0.0	92.2	0.9
2	120	6.1	0.8	3.7	0.5	0.0	0.0	0.0	0.0	85.1	1.0
3	180	11.3	1.2	6.2	0.6	0.0	0.0	0.0	0.0	78.3	1.4
4	240	15.5	0.8	8.2	0.3	0.0	0.0	0.0	0.0	73.2	2.0
5	300	17.7	0.3	9.0	0.3	0.0	0.0	0.0	0.0	69.1	1.3
6	600	27.5	1.2	12.5	0.5	0.0	0.0	0.0	0.0	55.7	1.9
7	1800	44.8	2.3	17.7	1.0	0.0	0.0	0.0	0.0	32.2	2.1
8	3600	55.1	1.1	20.5	0.7	0.4	0.7	0.0	0.0	19.6	2.1
9	7200	62.1	3.1	22.5	0.7	0.0	0.0	0.0	0.0	9.3	1.5

		Concentration (M), Trial 1					*Concentration (M), Trial 2*					*Concentration (M), Trial 3*				
Entry	t (sec)	Fluoride	Alkene	Alcohol	Ether	Bromide	Fluoride	Alkene	Alcohol	Ether	Bromide	Fluoride	Alkene	Alcohol	Ether	Bromide
1	3600	0.006	0.004	0.000	0.000	0.231	0.002	0.002	0.000	0.000	0.232	0.005	0.004	0.000	0.000	0.228
2	7200	0.013	0.008	0.000	0.000	0.215	0.016	0.010	0.000	0.000	0.213	0.017	0.010	0.000	0.000	0.211
3	10800	0.025	0.014	0.000	0.000	0.200	0.028	0.016	0.000	0.000	0.195	0.031	0.017	0.000	0.000	0.193
4	14400	0.040	0.020	0.000	0.000	0.187	0.036	0.020	0.000	0.000	0.185	0.040	0.021	0.000	0.000	0.177
5	18000	0.044	0.022	0.000	0.000	0.174	0.044	0.022	0.000	0.000	0.175	0.045	0.023	0.000	0.000	0.169
6	36000	0.066	0.030	0.000	0.000	0.143	0.068	0.031	0.000	0.000	0.140	0.072	0.033	0.000	0.000	0.134
7	108000	0.109	0.043	0.000	0.000	0.084	0.108	0.042	0.000	0.000	0.083	0.119	0.047	0.000	0.000	0.075
8	216000	0.137	0.051	0.000	0.000	0.048	0.136	0.050	0.000	0.000	0.055	0.141	0.053	0.003	0.000	0.044
9	432000	0.147	0.057	0.000	0.000	0.023	0.157	0.054	0.000	0.000	0.027	0.162	0.058	0.000	0.000	0.020

		Fluoride (M)		Alkene (M)		Alcohol (M)		Ether (M)		Bromide (M)	
Entry	t (sec)	*Ave.*	*Std. Dev.*	*Ave.*	*Std. Dev.*	*Ave.*	*Std. Dev.*	*Ave.*	*Std. Dev.*	*Ave.*	*Std. Dev.*
1	3600	0.004	0.002	0.004	0.001	0.000	0.000	0.000	0.000	0.230	0.002
2	7200	0.015	0.002	0.009	0.001	0.000	0.000	0.000	0.000	0.213	0.002
3	10800	0.028	0.003	0.016	0.001	0.000	0.000	0.000	0.000	0.196	0.004
4	14400	0.039	0.002	0.020	0.001	0.000	0.000	0.000	0.000	0.183	0.005
5	18000	0.044	0.001	0.022	0.001	0.000	0.000	0.000	0.000	0.173	0.003
6	36000	0.069	0.003	0.031	0.001	0.000	0.000	0.000	0.000	0.139	0.005
7	108000	0.112	0.006	0.044	0.002	0.000	0.000	0.000	0.000	0.081	0.005
8	216000	0.138	0.003	0.051	0.002	0.001	0.002	0.000	0.000	0.049	0.005
9	432000	0.155	0.008	0.056	0.002	0.000	0.000	0.000	0.000	0.023	0.004

[a] The % composition was determined by ^{1}H NMR analysis of the crude reaction mixture.

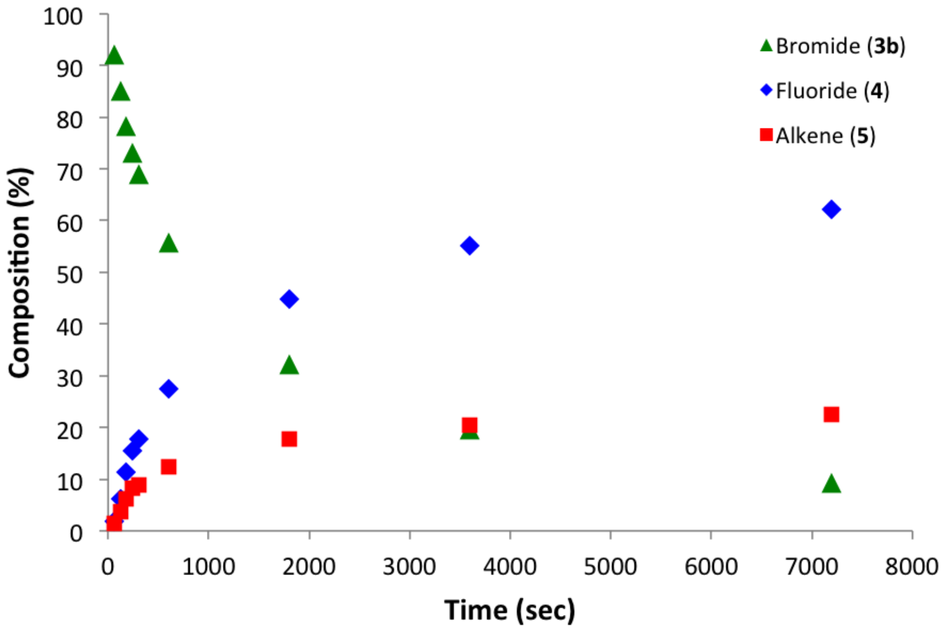

Figure S8: Rate profile with TBAF(Pin)$_2$ (**2g**). Each data point represents the average value of three independent trials.

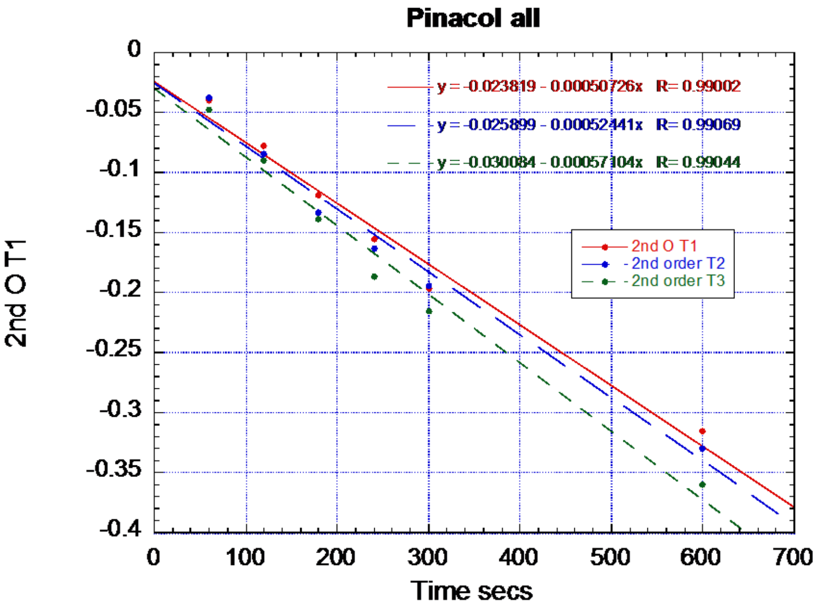

Figure S9: 2nd order plot for the reaction with TBAF(Pin)$_2$.

Table S6: Kinetic measurements using TBAF(*t*-BuOH)$_4$.[a]

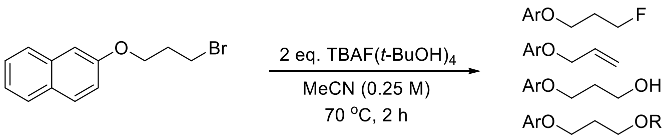

		Composition (%), Trial 1					*Composition (%), Trial 2*					*Composition (%), Trial 3*					Fluoride (%)		Alkene (%)		Alcohol (%)		Ether (%)		Bromide (%)	
Entry	t (sec)	Fluoride	Alkene	Alcohol	Ether	Bromide	Fluoride	Alkene	Alcohol	Ether	Bromide	Fluoride	Alkene	Alcohol	Ether	Bromide	*Ave.*	*Std. Dev.*	*Ave.*	*Std. Dev.*	*Ave.*	*Std. Dev.*	*Ave.*	*Std. Dev.*	*Ave.*	*Std. Dev.*
1	30	1.9	1.3	0.0	0.0	92.8	1.8	1.4	0.0	0.0	92.6	1.9	1.3	0.0	0.0	92.2	1.8	0.0	1.3	0.1	0.0	0.0	0.0	0.0	92.5	0.3
2	60	10.2	5.7	0.0	0.0	80.2	11.7	6.5	0.0	0.0	78.2	12.7	7.1	0.0	0.0	76.4	11.5	1.3	6.5	0.7	0.0	0.0	0.0	0.0	78.2	1.9
3	90	21.0	11.7	0.0	0.0	63.3	20.1	10.7	0.0	0.0	65.7	24.0	13.2	0.0	0.0	60.3	21.7	2.0	11.8	1.3	0.0	0.0	0.0	0.0	63.1	2.7
4	120	30.1	16.6	0.0	0.0	49.9	29.6	15.8	0.0	0.0	50.6	30.9	16.4	0.0	0.0	47.8	30.2	0.6	16.3	0.4	0.0	0.0	0.0	0.0	49.4	1.4
5	150	37.2	20.0	0.0	0.0	40.2	36.1	18.6	0.0	0.0	42.0	37.8	20.5	0.0	0.0	37.5	37.0	0.9	19.7	1.0	0.0	0.0	0.0	0.0	39.9	2.3
6	300	52.3	27.5	0.0	0.0	17.5	51.4	26.0	0.0	0.0	20.2	53.0	27.7	0.0	0.0	16.5	52.2	0.8	27.0	0.9	0.0	0.0	0.0	0.0	18.1	1.9
7	600	59.5	31.1	0.0	0.0	6.7	58.9	29.7	0.0	0.0	8.0	61.1	31.4	0.0	0.0	5.5	59.8	1.1	30.8	0.9	0.0	0.0	0.0	0.0	6.7	1.3
8	1800	63.0	33.1	0.0	0.0	0.6	64.1	32.2	0.0	0.0	0.9	63.3	33.4	0.0	0.0	0.7	63.5	0.6	32.9	0.6	0.0	0.0	0.0	0.0	0.7	0.2
9	3600	63.4	32.8	0.0	0.0	1.0	64.7	32.2	0.0	0.0	0.5	63.2	33.0	0.0	0.0	1.2	63.8	0.8	32.7	0.4	0.0	0.0	0.0	0.0	0.9	0.3
10	7200	63.5	33.2	0.0	0.0	0.6	64.0	32.9	0.0	0.0	0.7	63.7	33.0	0.0	0.0	0.6	63.8	0.3	33.0	0.1	0.0	0.0	0.0	0.0	0.6	0.1

		Concentration (M), Trial 1					*Concentration (M), Trial 2*					*Concentration (M), Trial 3*					Fluoride (M)		Alkene (M)		Alcohol (M)		Ether (M)		Bromide (M)	
Entry	t (sec)	Fluoride	Alkene	Alcohol	Ether	Bromide	Fluoride	Alkene	Alcohol	Ether	Bromide	Fluoride	Alkene	Alcohol	Ether	Bromide	*Ave.*	*Std. Dev.*	*Ave.*	*Std. Dev.*	*Ave.*	*Std. Dev.*	*Ave.*	*Std. Dev.*	*Ave.*	*Std. Dev.*
1	30	0.005	0.003	0.000	0.000	0.232	0.005	0.004	0.000	0.000	0.231	0.005	0.003	0.000	0.000	0.230	0.005	0.000	0.003	0.000	0.000	0.000	0.000	0.000	0.231	0.001
2	60	0.025	0.014	0.000	0.000	0.201	0.029	0.016	0.000	0.000	0.195	0.032	0.018	0.000	0.000	0.191	0.029	0.003	0.016	0.002	0.000	0.000	0.000	0.000	0.196	0.005
3	90	0.052	0.029	0.000	0.000	0.158	0.050	0.027	0.000	0.000	0.164	0.060	0.033	0.000	0.000	0.151	0.054	0.005	0.030	0.003	0.000	0.000	0.000	0.000	0.158	0.007
4	120	0.075	0.041	0.000	0.000	0.125	0.074	0.039	0.000	0.000	0.126	0.077	0.041	0.000	0.000	0.119	0.075	0.002	0.041	0.001	0.000	0.000	0.000	0.000	0.124	0.004
5	150	0.093	0.050	0.000	0.000	0.100	0.090	0.046	0.000	0.000	0.105	0.095	0.051	0.000	0.000	0.094	0.093	0.002	0.049	0.002	0.000	0.000	0.000	0.000	0.100	0.006
6	300	0.131	0.069	0.000	0.000	0.044	0.129	0.065	0.000	0.000	0.051	0.132	0.069	0.000	0.000	0.041	0.131	0.002	0.068	0.002	0.000	0.000	0.000	0.000	0.045	0.005
7	600	0.149	0.078	0.000	0.000	0.017	0.147	0.074	0.000	0.000	0.020	0.153	0.079	0.000	0.000	0.014	0.150	0.003	0.077	0.002	0.000	0.000	0.000	0.000	0.017	0.003
8	1800	0.157	0.083	0.000	0.000	0.001	0.160	0.080	0.000	0.000	0.002	0.158	0.083	0.000	0.000	0.002	0.159	0.001	0.082	0.002	0.000	0.000	0.000	0.000	0.002	0.000
9	3600	0.159	0.082	0.000	0.000	0.002	0.162	0.080	0.000	0.000	0.001	0.158	0.082	0.000	0.000	0.003	0.159	0.002	0.082	0.001	0.000	0.000	0.000	0.000	0.002	0.001
10	7200	0.159	0.083	0.000	0.000	0.002	0.160	0.082	0.000	0.000	0.002	0.159	0.082	0.000	0.000	0.001	0.159	0.001	0.083	0.000	0.000	0.000	0.000	0.000	0.002	0.000

[a] The % composition was determined by ^{1}H NMR analysis of the crude reaction mixture.

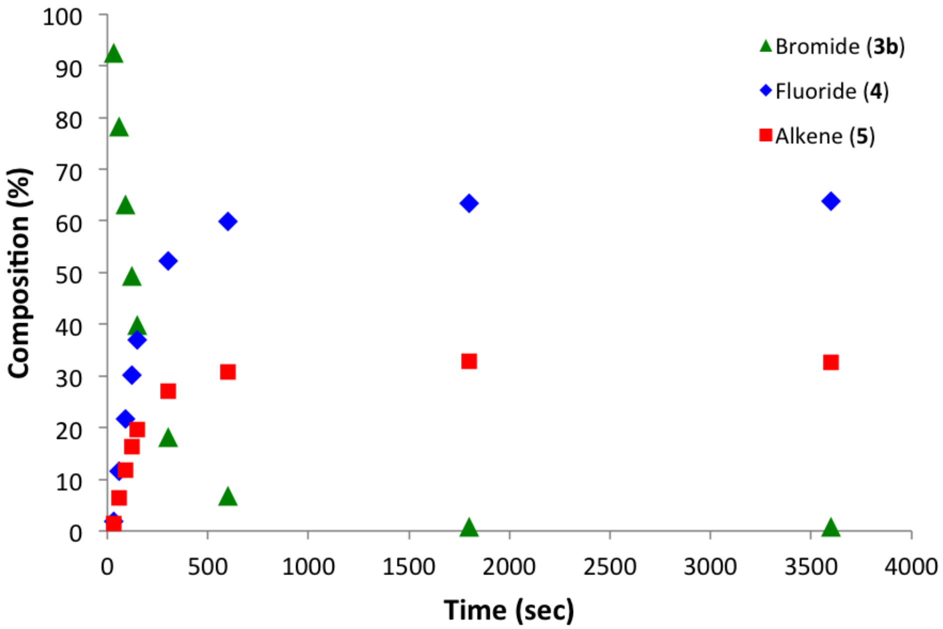

Figure S10: Rate profile with TBAF(t-BuOH)$_4$. Each data point represents the average value of three independent trials.

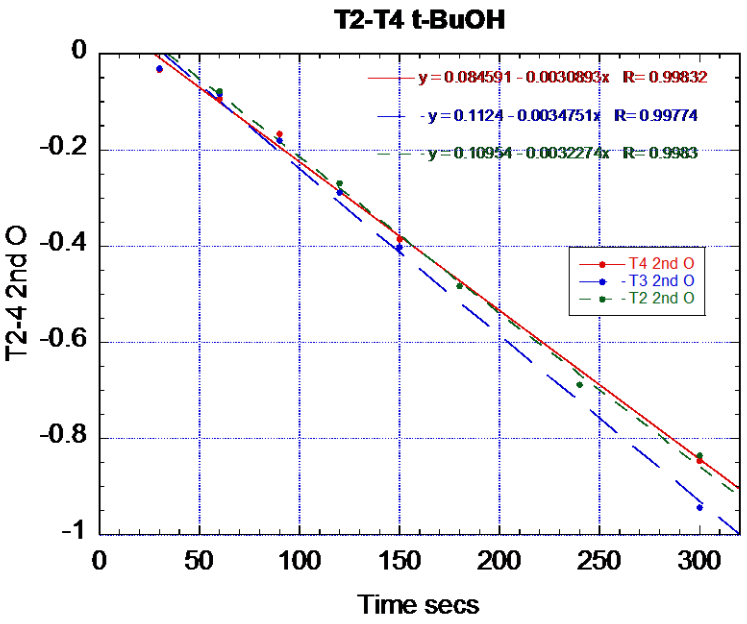

Figure S11: 2nd order plot for the reaction with TBAF(t-BuOH)$_4$.

Table S7: Kinetic measurements using TBAF$(H_2O)_3$.[a]

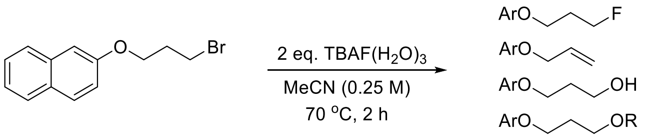

		Composition (%), Trial 1					*Composition (%), Trial 2*					*Composition (%), Trial 3*				
Entry	t (sec)	Fluoride	Alkene	Alcohol	Ether	Bromide	Fluoride	Alkene	Alcohol	Ether	Bromide	Fluoride	Alkene	Alcohol	Ether	Bromide
1	60	12.2	8.7	1.9	0.0	74.8	10.2	7.7	1.6	0.0	75.6	9.1	7.0	1.4	0.0	77.3
2	120	17.9	12.4	2.5	0.0	64.7	21.0	14.5	3.0	0.0	56.8	18.1	13.1	2.1	0.0	61.7
3	180	24.9	16.3	3.4	0.0	52.7	27.3	17.9	4.0	0.0	46.3	26.2	17.8	3.0	0.0	48.9
4	240	29.1	18.4	3.7	0.0	47.0	31.4	20.6	4.4	0.0	39.6	31.5	20.6	3.8	0.0	41.0
5	300	32.6	20.4	4.1	0.0	40.4	33.9	21.8	5.1	0.0	35.0	34.9	22.7	3.9	0.0	34.5
6	600	42.9	25.7	5.2	0.0	23.1	42.2	25.9	6.3	0.0	21.9	42.5	26.4	5.1	0.0	22.9
7	1800	52.9	29.8	6.5	0.0	7.8	51.0	29.4	8.4	0.0	8.0	51.6	30.1	6.9	0.0	9.1
8	3600	55.8	31.0	7.0	0.0	2.4	54.1	30.6	9.1	0.0	2.2	55.1	32.2	6.8	0.0	2.8
9	7200	56.9	31.7	7.5	0.0	0.8	55.3	31.5	8.6	0.0	0.3	56.6	32.5	7.4	0.0	0.8

		Fluoride (%)		Alkene (%)		Alcohol (%)		Ether (%)		Bromide (%)	
Entry	t (sec)	*Ave.*	*Std. Dev.*	*Ave.*	*Std. Dev.*	*Ave.*	*Std. Dev.*	*Ave.*	*Std. Dev.*	*Ave.*	*Std. Dev.*
1	60	10.5	1.6	7.8	0.9	1.6	0.2	0.0	0.0	75.9	1.3
2	120	19.0	1.7	13.4	1.1	2.5	0.5	0.0	0.0	61.1	4.0
3	180	26.1	1.2	17.4	0.9	3.4	0.5	0.0	0.0	49.3	3.2
4	240	30.7	1.3	19.9	1.3	4.0	0.4	0.0	0.0	42.5	3.9
5	300	33.8	1.2	21.6	1.2	4.3	0.6	0.0	0.0	36.7	3.3
6	600	42.5	0.3	26.0	0.4	5.5	0.7	0.0	0.0	22.6	0.6
7	1800	51.8	1.0	29.8	0.4	7.3	1.0	0.0	0.0	8.3	0.7
8	3600	55.0	0.9	31.3	0.8	7.6	1.3	0.0	0.0	2.5	0.3
9	7200	56.3	0.8	31.9	0.5	7.9	0.7	0.0	0.0	0.6	0.3

		Concentration (M), Trial 1					*Concentration (M), Trial 2*					*Concentration (M), Trial 3*				
Entry	t (sec)	Fluoride	Alkene	Alcohol	Ether	Bromide	Fluoride	Alkene	Alcohol	Ether	Bromide	Fluoride	Alkene	Alcohol	Ether	Bromide
1	60	0.031	0.022	0.005	0.000	0.187	0.026	0.019	0.004	0.000	0.189	0.023	0.017	0.004	0.000	0.193
2	120	0.045	0.031	0.006	0.000	0.162	0.052	0.036	0.008	0.000	0.142	0.045	0.033	0.005	0.000	0.154
3	180	0.062	0.041	0.008	0.000	0.132	0.068	0.045	0.010	0.000	0.116	0.065	0.045	0.007	0.000	0.122
4	240	0.073	0.046	0.009	0.000	0.117	0.078	0.052	0.011	0.000	0.099	0.079	0.051	0.010	0.000	0.103
5	300	0.081	0.051	0.010	0.000	0.101	0.085	0.054	0.013	0.000	0.088	0.087	0.057	0.010	0.000	0.086
6	600	0.107	0.064	0.013	0.000	0.058	0.105	0.065	0.016	0.000	0.055	0.106	0.066	0.013	0.000	0.057
7	1800	0.132	0.075	0.016	0.000	0.020	0.127	0.073	0.021	0.000	0.020	0.129	0.075	0.017	0.000	0.023
8	3600	0.140	0.077	0.017	0.000	0.006	0.135	0.077	0.023	0.000	0.005	0.138	0.081	0.017	0.000	0.007
9	7200	0.142	0.079	0.019	0.000	0.002	0.138	0.079	0.022	0.000	0.001	0.141	0.081	0.019	0.000	0.002

		Fluoride (M)		Alkene (M)		Alcohol (M)		Ether (M)		Bromide (M)	
Entry	t (sec)	*Ave.*	*Std. Dev.*	*Ave.*	*Std. Dev.*	*Ave.*	*Std. Dev.*	*Ave.*	*Std. Dev.*	*Ave.*	*Std. Dev.*
1	60	0.026	0.004	0.020	0.002	0.004	0.001	0.000	0.000	0.190	0.003
2	120	0.047	0.004	0.033	0.003	0.006	0.001	0.000	0.000	0.153	0.010
3	180	0.065	0.003	0.043	0.002	0.009	0.001	0.000	0.000	0.123	0.008
4	240	0.077	0.003	0.050	0.003	0.010	0.001	0.000	0.000	0.106	0.010
5	300	0.084	0.003	0.054	0.003	0.011	0.002	0.000	0.000	0.092	0.008
6	600	0.106	0.001	0.065	0.001	0.014	0.002	0.000	0.000	0.057	0.002
7	1800	0.130	0.003	0.074	0.001	0.018	0.003	0.000	0.000	0.021	0.002
8	3600	0.138	0.002	0.078	0.002	0.019	0.003	0.000	0.000	0.006	0.001
9	7200	0.141	0.002	0.080	0.001	0.020	0.002	0.000	0.000	0.002	0.001

[a] The % composition was determined by ^{1}H NMR analysis of the crude reaction mixture.

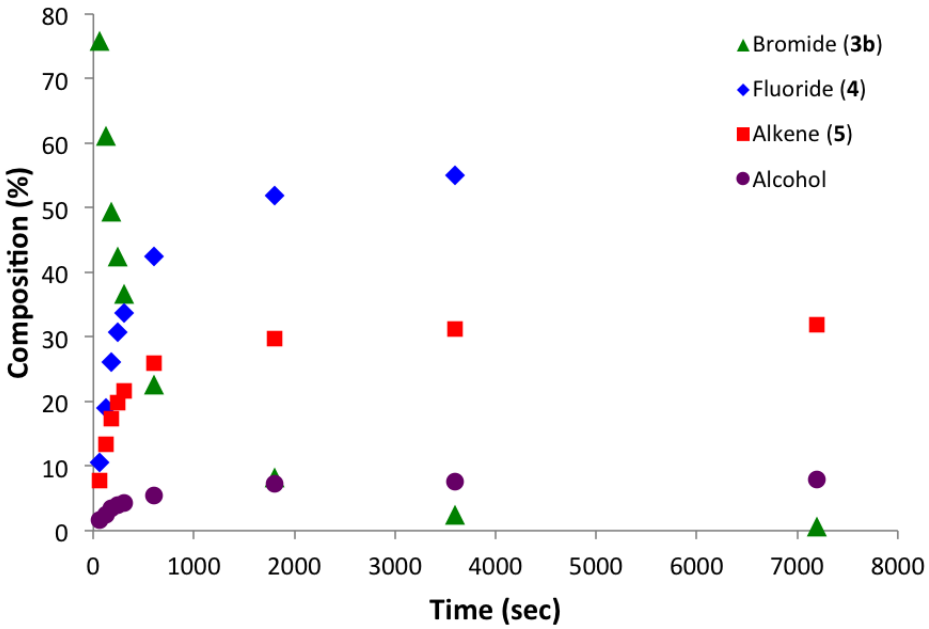

Figure S12: Rate profile with TBAF$(H_2O)_3$. Each data point represents the average value of three independent trials.

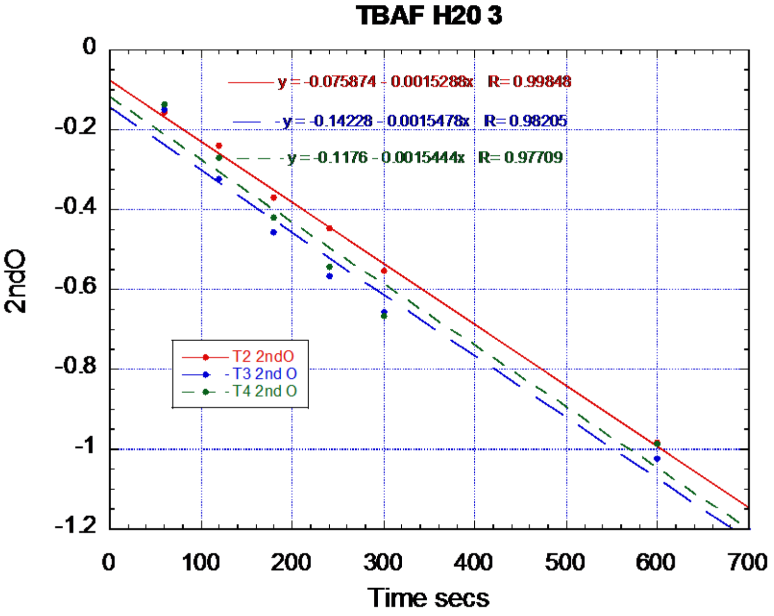

Figure S13: 2nd order plot for the reaction with TBAF(H_2O)$_3$.

Table S8: Kinetic measurements using TBAF(9-phenyl-9-fluorenol)$_3$ (**2j**).[a]

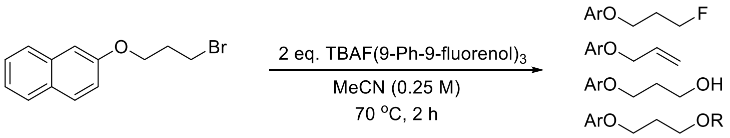

		Composition (%), Trial 1					*Composition (%), Trial 2*					*Composition (%), Trial 3*					Fluoride (%)		Alkene (%)		Alcohol (%)		Ether (%)		Bromide (%)	
Entry	t (sec)	Fluoride	Alkene	Alcohol	Ether	Bromide	Fluoride	Alkene	Alcohol	Ether	Bromide	Fluoride	Alkene	Alcohol	Ether	Bromide	*Ave.*	*Std. Dev.*	*Ave.*	*Std. Dev.*	*Ave.*	*Std. Dev.*	*Ave.*	*Std. Dev.*	*Ave.*	*Std. Dev.*
1	60	2.9	0.2	0.0	0.0	92.9	2.3	1.6	0.0	0.0	92.0	3.8	1.1	0.0	0.0	94.0	3.0	0.8	1.0	0.7	0.0	0.0	0.0	0.0	93.0	1.0
2	120	7.3	3.3	0.0	0.0	84.6	10.7	4.9	0.0	0.0	77.3	8.0	3.9	0.0	0.0	74.9	8.7	1.8	4.0	0.8	0.0	0.0	0.0	0.0	78.9	5.1
3	180	11.8	4.8	0.0	0.0	74.1	19.4	8.1	0.0	0.0	60.3	13.9	5.7	0.0	0.0	70.2	15.0	3.9	6.2	1.7	0.0	0.0	0.0	0.0	68.2	7.1
4	240	17.6	6.3	0.0	0.0	68.1	25.7	10.0	0.0	0.0	57.5	22.5	8.9	0.0	0.0	54.1	21.9	4.1	8.4	1.9	0.0	0.0	0.0	0.0	59.9	7.3
5	300	22.8	7.8	0.0	0.0	61.5	29.5	10.9	0.0	0.0	50.9	29.2	10.6	0.0	0.0	51.7	27.2	3.8	9.7	1.7	0.0	0.0	0.0	0.0	54.7	5.9
6	600	36.4	10.9	0.0	0.0	44.3	43.8	14.5	0.0	0.0	31.2	43.8	14.3	0.0	0.0	31.8	41.4	4.3	13.3	2.0	0.0	0.0	0.0	0.0	35.8	7.4
7	1800	55.5	15.5	0.0	0.0	15.2	59.9	18.8	0.0	0.0	8.2	61.2	17.9	0.0	0.0	10.1	58.9	3.0	17.4	1.7	0.0	0.0	0.0	0.0	11.2	3.6
8	3600	65.9	17.5	0.0	0.0	6.3	67.7	19.9	0.0	0.0	2.4	67.0	19.2	0.0	0.0	1.7	66.8	0.9	18.9	1.2	0.0	0.0	0.0	0.0	3.4	2.5
9	7200	69.4	17.5	0.0	0.0	1.0	65.7	19.9	0.0	0.0	1.7	64.8	18.9	0.0	0.0	1.6	66.6	2.4	18.7	1.2	0.0	0.0	0.0	0.0	1.4	0.4

		Concentration (M), Trial 1					*Concentration (M), Trial 2*					*Concentration (M), Trial 3*					Fluoride (M)		Alkene (M)		Alcohol (M)		Ether (M)		Bromide (M)	
Entry	t (sec)	Fluoride	Alkene	Alcohol	Ether	Bromide	Fluoride	Alkene	Alcohol	Ether	Bromide	Fluoride	Alkene	Alcohol	Ether	Bromide	*Ave.*	*Std. Dev.*	*Ave.*	*Std. Dev.*	*Ave.*	*Std. Dev.*	*Ave.*	*Std. Dev.*	*Ave.*	*Std. Dev.*
1	60	0.007	0.000	0.000	0.000	0.232	0.006	0.004	0.000	0.000	0.230	0.010	0.003	0.000	0.000	0.235	0.008	0.002	0.002	0.002	0.000	0.000	0.000	0.000	0.232	0.003
2	120	0.018	0.008	0.000	0.000	0.212	0.027	0.012	0.000	0.000	0.193	0.020	0.010	0.000	0.000	0.187	0.022	0.004	0.010	0.002	0.000	0.000	0.000	0.000	0.197	0.013
3	180	0.030	0.012	0.000	0.000	0.185	0.048	0.020	0.000	0.000	0.151	0.035	0.014	0.000	0.000	0.176	0.038	0.010	0.015	0.004	0.000	0.000	0.000	0.000	0.171	0.018
4	240	0.044	0.016	0.000	0.000	0.170	0.064	0.025	0.000	0.000	0.144	0.056	0.022	0.000	0.000	0.135	0.055	0.010	0.021	0.005	0.000	0.000	0.000	0.000	0.150	0.018
5	300	0.057	0.020	0.000	0.000	0.154	0.074	0.027	0.000	0.000	0.127	0.073	0.026	0.000	0.000	0.129	0.068	0.010	0.024	0.004	0.000	0.000	0.000	0.000	0.137	0.015
6	600	0.091	0.027	0.000	0.000	0.111	0.110	0.036	0.000	0.000	0.078	0.110	0.036	0.000	0.000	0.080	0.103	0.011	0.033	0.005	0.000	0.000	0.000	0.000	0.089	0.018
7	1800	0.139	0.039	0.000	0.000	0.038	0.150	0.047	0.000	0.000	0.020	0.153	0.045	0.000	0.000	0.025	0.147	0.007	0.044	0.004	0.000	0.000	0.000	0.000	0.028	0.009
8	3600	0.165	0.044	0.000	0.000	0.016	0.169	0.050	0.000	0.000	0.006	0.167	0.048	0.000	0.000	0.004	0.167	0.002	0.047	0.003	0.000	0.000	0.000	0.000	0.009	0.006
9	7200	0.173	0.044	0.000	0.000	0.003	0.164	0.050	0.000	0.000	0.004	0.162	0.047	0.000	0.000	0.004	0.167	0.006	0.047	0.003	0.000	0.000	0.000	0.000	0.004	0.001

[a] The % composition was determined by ^{1}H NMR analysis of the crude reaction mixture.

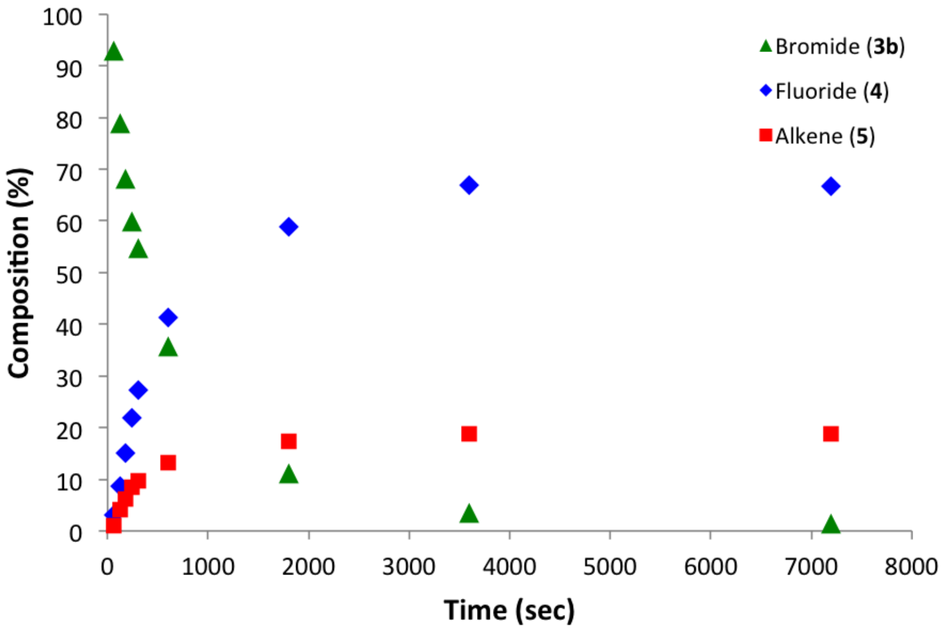

Figure S14: Rate profile with TBAF(9-phenyl-9-fluorenol)$_3$ (**2j**). Each data point represents the average value of three independent trials.

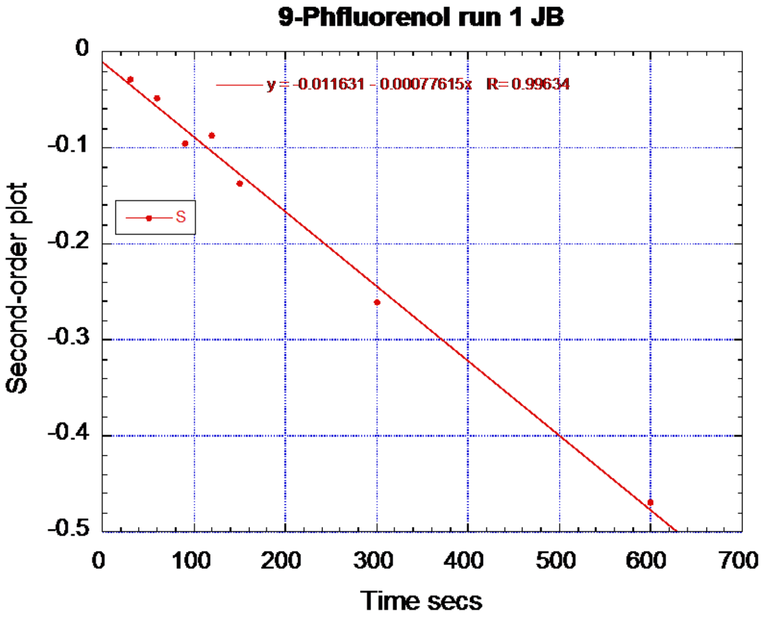

Figure S15: 2nd order plot for the first run of the reaction with TBAF(9-Ph-9-fluorenol)$_3$.

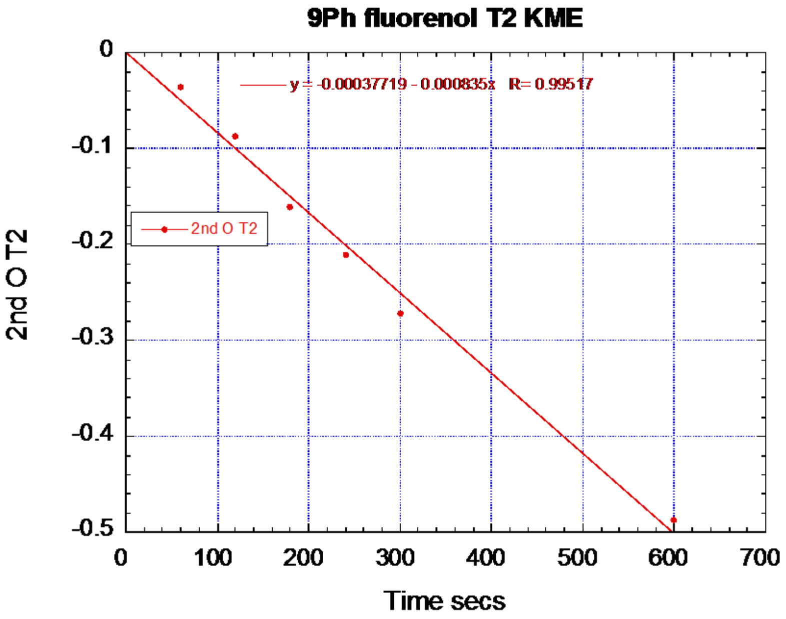

Figure S16: 2nd order plot for the second run of the reaction with TBAF(9-Ph-9-fluorenol)$_3$.

Table S9: Kinetic measurements using TBAF(p-Tol$_3$COH)$_2$ (**2m**)[a]

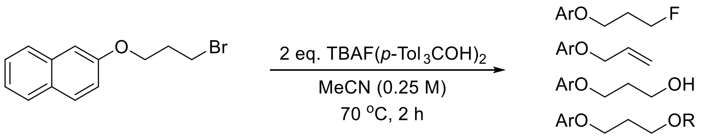

		Composition (%), Trial 1					*Composition (%), Trial 2*					*Composition (%), Trial 3*					Fluoride (%)		Alkene (%)		Alcohol (%)		Ether (%)		Bromide (%)	
Entry	t (sec)	Fluoride	Alkene	Alcohol	Ether	Bromide	Fluoride	Alkene	Alcohol	Ether	Bromide	Fluoride	Alkene	Alcohol	Ether	Bromide	*Ave.*	*Std. Dev.*	*Ave.*	*Std. Dev.*	*Ave.*	*Std. Dev.*	*Ave.*	*Std. Dev.*	*Ave.*	*Std. Dev.*
1	30	7.0	5.0	0.0	0.0	81.7	4.3	4.0	0.0	0.0	90.1	5.2	4.1	0.0	0.0	81.0	5.5	1.4	4.4	0.5	0.0	0.0	0.0	0.0	84.3	5.1
2	60	17.6	11.2	0.0	0.0	63.0	19.7	12.2	0.0	0.0	66.9	19.0	12.3	0.0	0.0	59.8	18.8	1.1	11.9	0.6	0.0	0.0	0.0	0.0	63.3	3.5
3	90	29.4	16.9	0.0	0.0	46.8	30.4	17.5	0.0	0.0	47.7	31.2	18.1	0.0	0.0	42.1	30.3	0.9	17.5	0.6	0.0	0.0	0.0	0.0	45.5	3.0
4	120	37.6	20.6	0.0	0.0	35.4	39.0	21.7	0.0	0.0	35.6	40.4	22.3	0.0	0.0	30.8	39.0	1.4	21.5	0.8	0.0	0.0	0.0	0.0	33.9	2.7
5	150	42.5	22.4	0.0	0.0	25.6	43.4	23.2	0.0	0.0	26.3	45.2	24.3	0.0	0.0	22.6	43.7	1.4	23.3	0.9	0.0	0.0	0.0	0.0	24.8	2.0
6	300	55.2	27.3	0.0	0.0	8.8	57.0	29.1	0.0	0.0	10.4	56.8	29.0	0.0	0.0	6.8	56.3	1.0	28.5	1.0	0.0	0.0	0.0	0.0	8.7	1.8
7	600	62.9	30.7	0.0	0.0	1.8	61.9	30.4	0.0	0.0	1.7	61.2	30.6	0.0	0.0	0.8	62.0	0.8	30.6	0.2	0.0	0.0	0.0	0.0	1.4	0.5
8	1800	62.8	31.0	0.0	0.0	0.2	63.0	30.7	0.0	0.0	0.0	60.6	30.3	0.0	0.0	0.0	62.1	1.3	30.7	0.4	0.0	0.0	0.0	0.0	0.1	0.1
9	3600	59.6	29.0	0.0	0.0	0.0	61.9	30.3	0.0	0.0	0.0	62.6	31.1	0.0	0.0	0.0	61.4	1.6	30.1	1.0	0.0	0.0	0.0	0.0	0.0	0.0

		Concentration (M), Trial 1					*Concentration (M), Trial 2*					*Concentration (M), Trial 3*					Fluoride (M)		Alkene (M)		Alcohol (M)		Ether (M)		Bromide (M)	
Entry	t (sec)	Fluoride	Alkene	Alcohol	Ether	Bromide	Fluoride	Alkene	Alcohol	Ether	Bromide	Fluoride	Alkene	Alcohol	Ether	Bromide	*Ave.*	*Std. Dev.*	*Ave.*	*Std. Dev.*	*Ave.*	*Std. Dev.*	*Ave.*	*Std. Dev.*	*Ave.*	*Std. Dev.*
1	30	0.017	0.012	0.000	0.000	0.204	0.011	0.010	0.000	0.000	0.225	0.013	0.010	0.000	0.000	0.203	0.014	0.003	0.011	0.001	0.000	0.000	0.000	0.000	0.211	0.013
2	60	0.044	0.028	0.000	0.000	0.158	0.049	0.031	0.000	0.000	0.167	0.048	0.031	0.000	0.000	0.150	0.047	0.003	0.030	0.002	0.000	0.000	0.000	0.000	0.158	0.009
3	90	0.074	0.042	0.000	0.000	0.117	0.076	0.044	0.000	0.000	0.119	0.078	0.045	0.000	0.000	0.105	0.076	0.002	0.044	0.001	0.000	0.000	0.000	0.000	0.114	0.008
4	120	0.094	0.052	0.000	0.000	0.089	0.098	0.054	0.000	0.000	0.089	0.101	0.056	0.000	0.000	0.077	0.098	0.004	0.054	0.002	0.000	0.000	0.000	0.000	0.085	0.007
5	150	0.106	0.056	0.000	0.000	0.064	0.108	0.058	0.000	0.000	0.066	0.113	0.061	0.000	0.000	0.056	0.109	0.003	0.058	0.002	0.000	0.000	0.000	0.000	0.062	0.005
6	300	0.138	0.068	0.000	0.000	0.022	0.142	0.073	0.000	0.000	0.026	0.142	0.073	0.000	0.000	0.017	0.141	0.002	0.071	0.002	0.000	0.000	0.000	0.000	0.022	0.004
7	600	0.157	0.077	0.000	0.000	0.004	0.155	0.076	0.000	0.000	0.004	0.153	0.076	0.000	0.000	0.002	0.155	0.002	0.076	0.000	0.000	0.000	0.000	0.000	0.004	0.001
8	1800	0.157	0.077	0.000	0.000	0.001	0.157	0.077	0.000	0.000	0.000	0.151	0.076	0.000	0.000	0.000	0.155	0.003	0.077	0.001	0.000	0.000	0.000	0.000	0.000	0.000
9	3600	0.149	0.073	0.000	0.000	0.000	0.155	0.076	0.000	0.000	0.000	0.156	0.078	0.000	0.000	0.000	0.153	0.004	0.075	0.003	0.000	0.000	0.000	0.000	0.000	0.000

[a] The % composition was determined by ^{1}H NMR analysis of the crude reaction mixture.

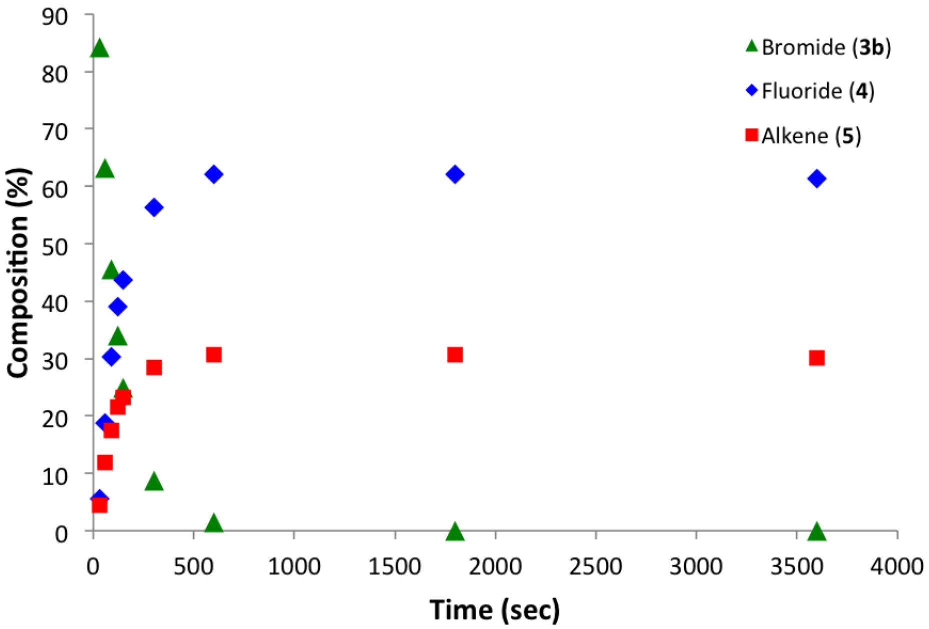

Figure S17: Rate profile with TBAF(p-$Tol_3COH)_2$ (**2m**). Each data point represents the average value of three independent trials.

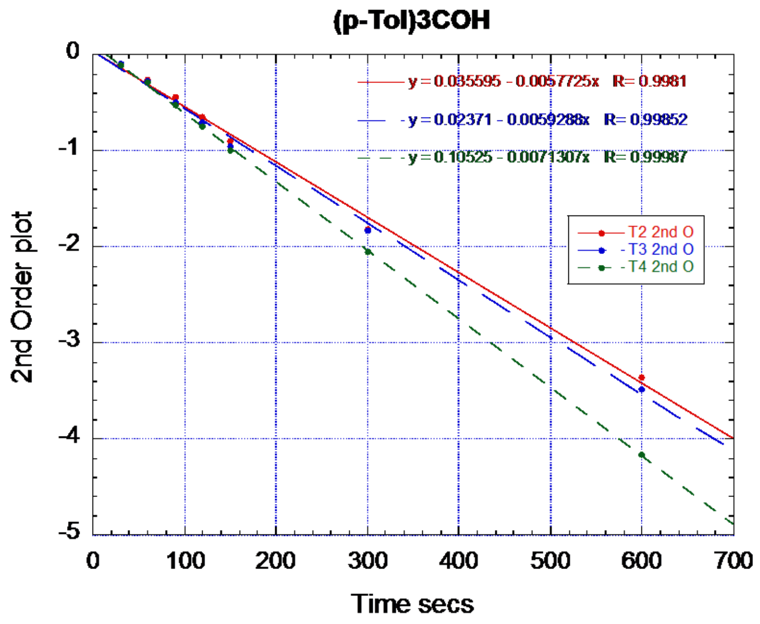

Figure S18: 2nd order plot for the reaction with TBAF(p-$Tol_3COH)_2$.

COUNTERION STUDIES

Rate profile measurements with [tetraalkylammonium fluoride–(pinacol)$_2$] and with [tetraalkylammonium fluoride–(neopentylglycol)$_2$] complexes:

To a 14 mL screw-top vial, were added **3b** (53.0 mg, 0.2 mmol), MeCN (0.8 mL) and [tetraalkylammonium fluoride–alcohol] complex (0.4 mmol). The vial was capped with a rubber septum, then stirred at 70 °C for 2 h. At the indicated time points, a small aliquot (< 80 μL) was taken with a syringe and needle. Each aliquot was filtered through a short plug of silica gel to quench the reaction, and the silica gel was washed with EtOAc (3 × 2 mL). The combined rinsings were concentrated *in vacuo*, and the resulting residue was analyzed by ^{1}H NMR. The conversion at each time point was determined by integration of the methylene/allylic proton signals depicted in Figure S1. The results are summarized in Table S10. Details for each individual complex are shown in Tables S11–S14 and Figures S21–S24.

Table S10: Summary of kinetic data and reactivity studies.[a]

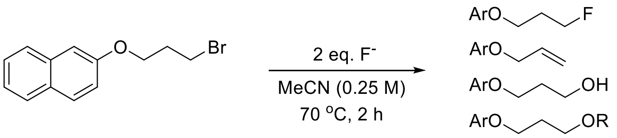

		Reaction Composition after 2 h (%, ^{1}H NMR)				
Entry	Fluoride Complex	Fluoride	Olefin	Alcohol	Ether	Bromide
1	TMAF(Pin)$_2$	68	38	1	0	3
2	TEAF(Pin)$_2$	68	25	0	0	7
3	TBAF(Pin)$_2$	67	23	0	0	10
4	TMAF(NPG)$_2$	47	16	0	0	37
5	TEAF(NPG)$_2$	54	18	0	0	27
6	TBAF(NPG)$_2$	45	14	0	0	41

[a] The % composition was determined by ^{1}H NMR analysis of the crude reaction mixture. Values for TBAF(Pin)$_2$ and TBAF(NPG)$_2$ represent average of three independent trials (reproduced from above). Other values are the result of single trials.

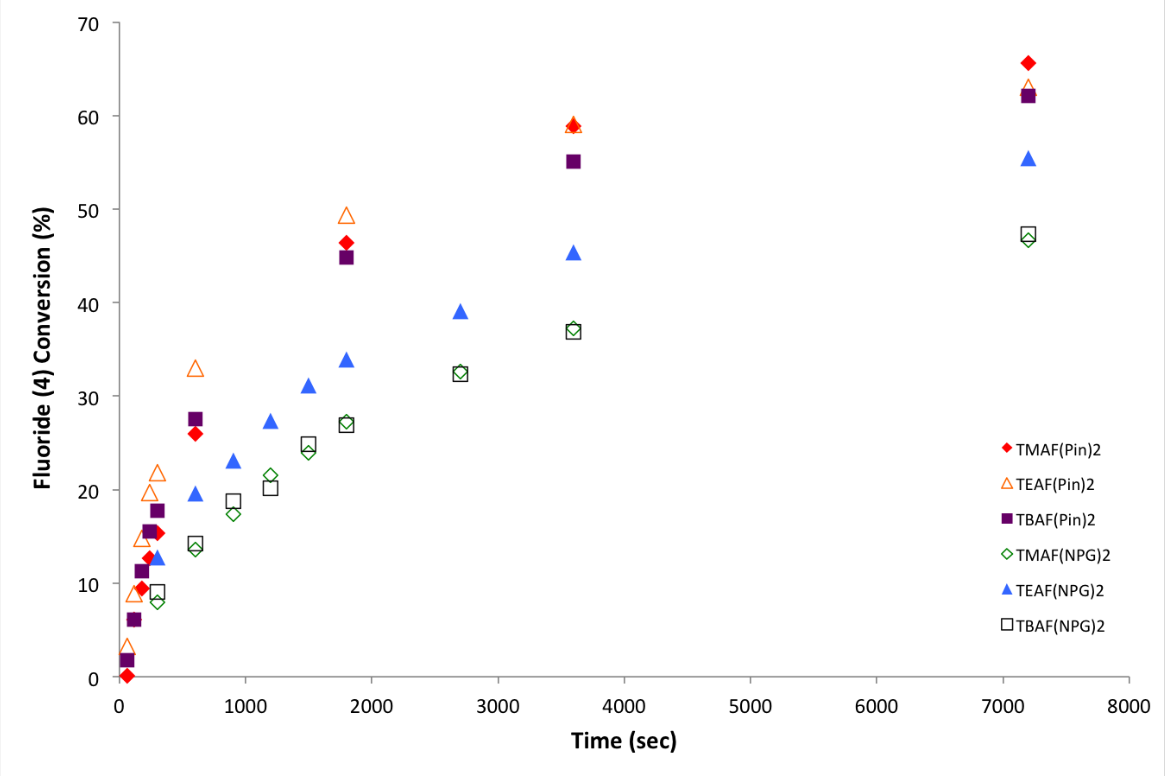

Figure S19: Summary of rate profiles for fluoride product (**4**) formation for [fluoride–alcohol] complexes examined. Values for TBAF(Pin)$_2$ and TBAF(NPG)$_2$ represent average of three independent trials (reproduced from above). Other values represent single trials.

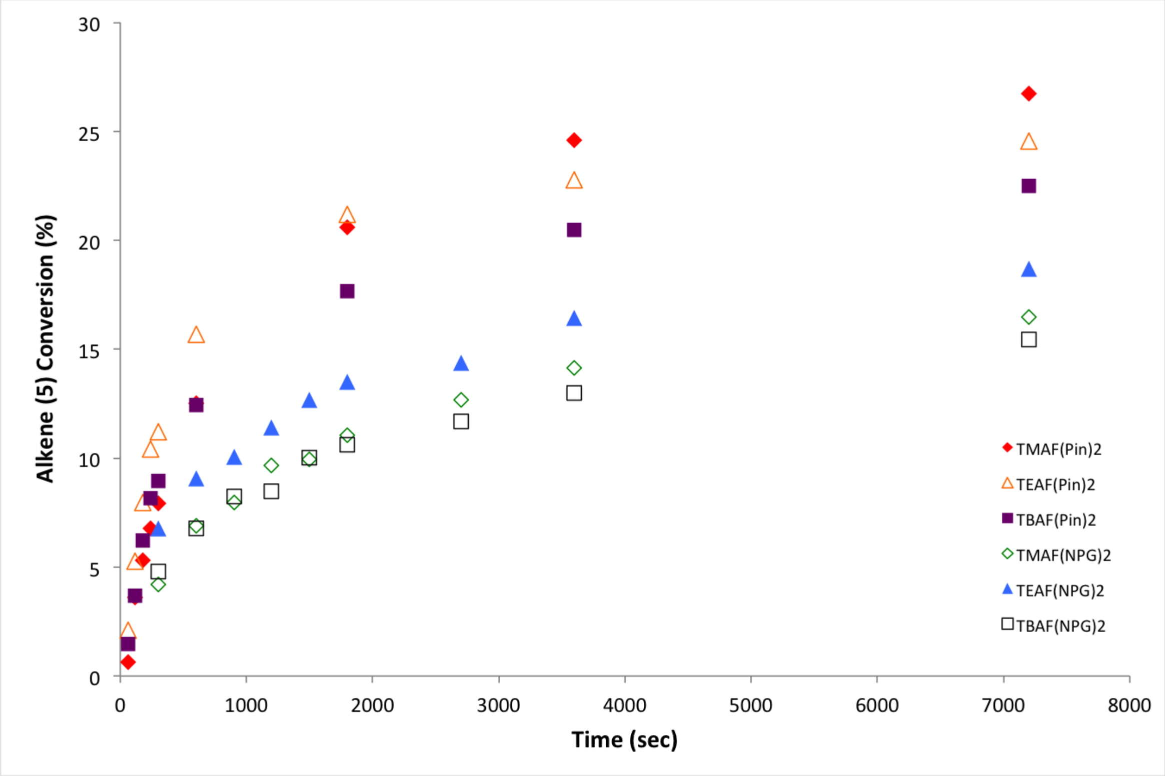

Figure S20: Summary of rate profiles for olefin product (**5**) formation for [fluoride–alcohol] complexes examined. Values for TBAF(Pin)$_2$ and TBAF(NPG)$_2$ represent average of three independent trials (reproduced from above). Other values represent single trials.

Table S11: Kinetic measurements using TMAF(Pin)$_2$.[a]

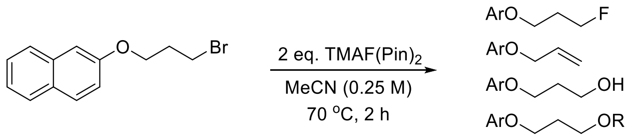

		Composition (%), Trial 1				
Entry	t (sec)	Fluoride	Alkene	Alcohol	Ether	Bromide
1	60	0.1	0.7	0.0	0.0	93.7
2	120	6.1	3.6	0.0	0.0	86.6
3	180	9.5	5.3	0.0	0.0	80.8
4	240	12.6	6.8	0.0	0.0	75.9
5	300	15.4	7.9	0.0	0.0	72.4
6	600	25.9	12.5	0.0	0.0	57.0
7	1800	46.4	20.6	1.2	0.0	29.0
8	3600	58.9	24.6	1.5	0.0	13.1
9	7200	65.6	26.7	1.4	0.0	2.1

		Concentration (M), Trial 1				
Entry	t (sec)	Fluoride	Alkene	Alcohol	Ether	Bromide
1	60	0.000	0.002	0.000	0.000	0.234
2	120	0.015	0.009	0.000	0.000	0.217
3	180	0.024	0.013	0.000	0.000	0.202
4	240	0.032	0.017	0.000	0.000	0.190
5	300	0.038	0.020	0.000	0.000	0.181
6	600	0.065	0.031	0.000	0.000	0.143
7	1800	0.116	0.052	0.003	0.000	0.072
8	3600	0.147	0.061	0.004	0.000	0.033
9	7200	0.164	0.067	0.003	0.000	0.005

[a] The % composition was determined by ^{1}H NMR analysis of the crude reaction mixture.

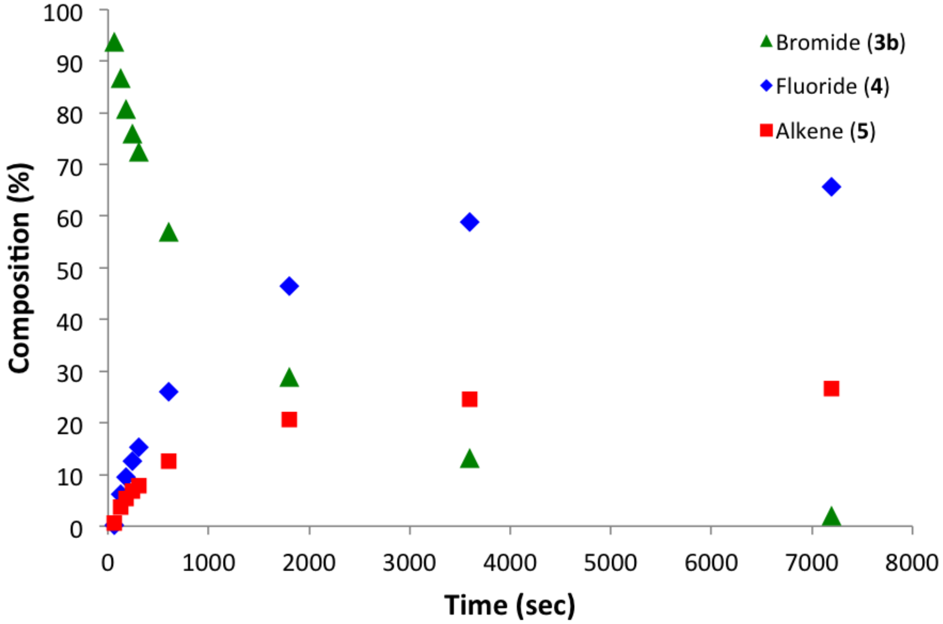

Figure S21: Rate profile with TMAF(Pin)$_2$.

Table S12: Kinetic measurements using TEAF(Pin)$_2$.[a]

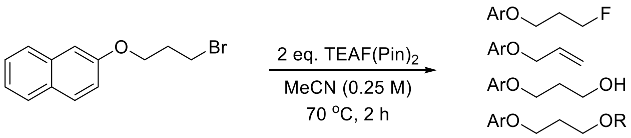

		Composition (%), Trial 1				
Entry	t (sec)	Fluoride	Alkene	Alcohol	Ether	Bromide
1	60	3.3	2.1	0.0	0.0	88.0
2	120	8.8	5.3	0.0	0.0	78.2
3	180	14.8	8.0	0.0	0.0	71.4
4	240	19.7	10.4	0.0	0.0	64.5
5	300	21.8	11.2	0.0	0.0	60.8
6	600	33.0	15.7	0.0	0.0	46.7
7	1800	49.4	21.2	0.0	0.0	24.0
8	3600	59.1	22.8	0.0	0.0	12.0
9	7200	63.0	24.5	0.0	0.0	4.6

		Concentration (M), Trial 1				
Entry	t (sec)	Fluoride	Alkene	Alcohol	Ether	Bromide
1	60	0.008	0.005	0.000	0.000	0.220
2	120	0.022	0.013	0.000	0.000	0.195
3	180	0.037	0.020	0.000	0.000	0.178
4	240	0.049	0.026	0.000	0.000	0.161
5	300	0.055	0.028	0.000	0.000	0.152
6	600	0.083	0.039	0.000	0.000	0.117
7	1800	0.123	0.053	0.000	0.000	0.060
8	3600	0.148	0.057	0.000	0.000	0.030
9	7200	0.158	0.061	0.000	0.000	0.012

[a] The % composition was determined by ^{1}H NMR analysis of the crude reaction mixture.

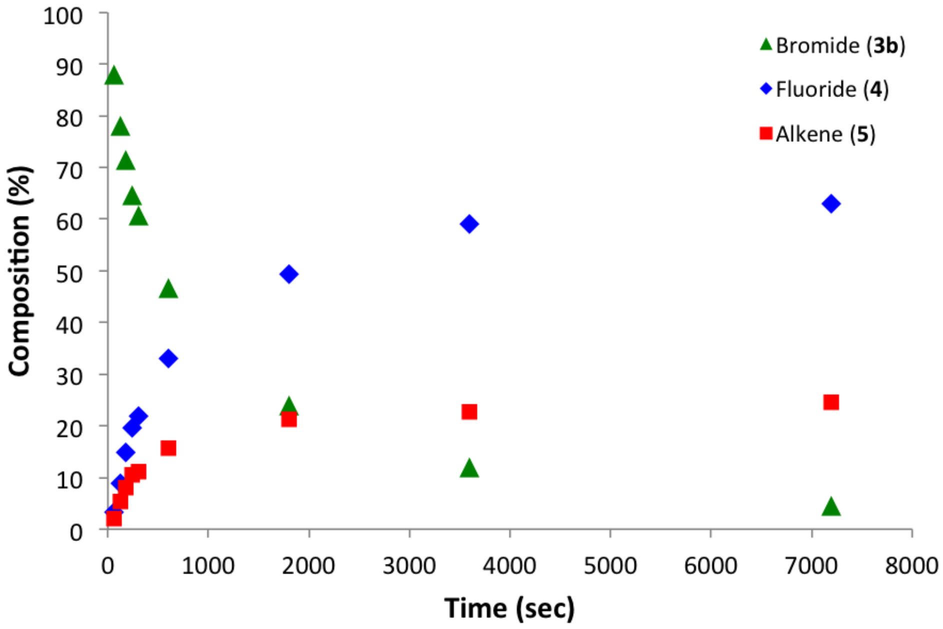

Figure S22: Rate profile with TEAF(Pin)$_2$.

Table S13: Kinetic measurements using TMAF(NPG)$_2$.[a]

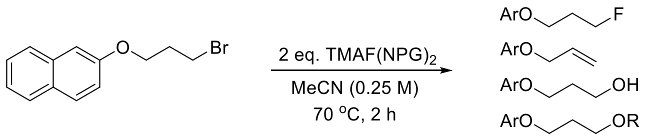

		Composition (%), Trial 1				
Entry	t (sec)	Fluoride	Alkene	Alcohol	Ether	Bromide
1	300	8.0	4.2	0.0	0.0	91.1
2	600	13.6	6.9	0.0	0.0	82.4
3	900	17.4	8.0	0.0	0.0	74.1
4	1200	21.5	9.6	0.0	0.0	69.4
5	1500	24.0	10.0	0.0	0.0	64.8
6	1800	27.3	11.1	0.0	0.0	63.9
7	2700	32.6	12.7	0.0	0.0	58.5
8	3600	37.3	14.2	0.0	0.0	50.6
9	7200	46.7	16.5	0.0	0.0	40.2

		Concentration (M), Trial 1				
Entry	t (sec)	Fluoride	Alkene	Alcohol	Ether	Bromide
1	18000	0.020	0.011	0.000	0.000	0.228
2	36000	0.034	0.017	0.000	0.000	0.206
3	54000	0.043	0.020	0.000	0.000	0.185
4	72000	0.054	0.024	0.000	0.000	0.174
5	90000	0.060	0.025	0.000	0.000	0.162
6	108000	0.068	0.028	0.000	0.000	0.160
7	162000	0.081	0.032	0.000	0.000	0.146
8	216000	0.093	0.035	0.000	0.000	0.126
9	432000	0.117	0.041	0.000	0.000	0.101

[a] The % composition was determined by ^{1}H NMR analysis of the crude reaction mixture.

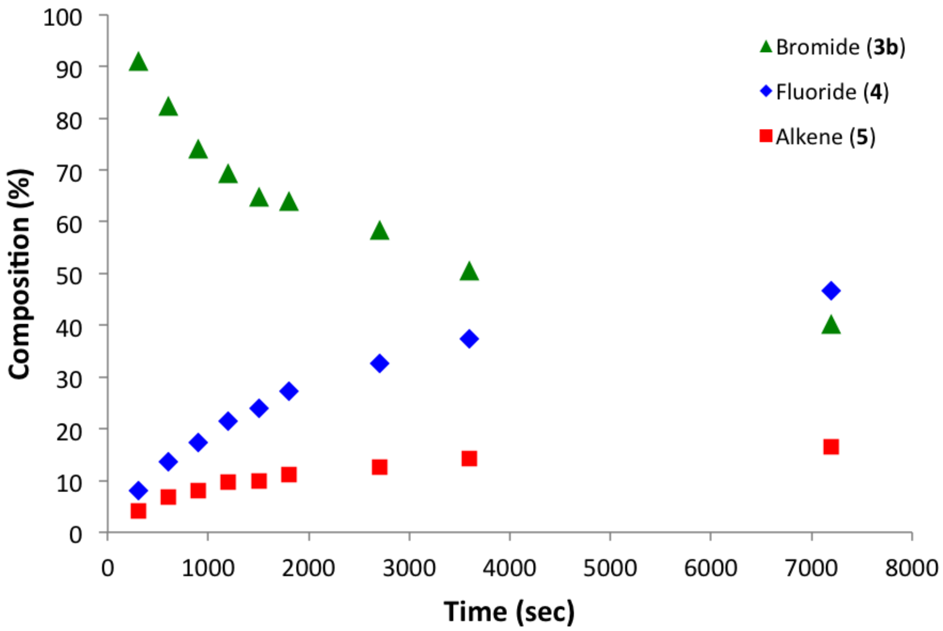

Figure S23: Rate profile with TMAF(NPG)$_2$.

Table S14: Kinetic measurements using TEAF(NPG)$_2$.[a]

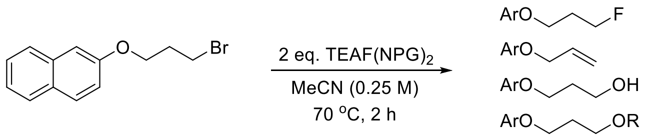

		Composition (%), Trial 1				
Entry	t (min)	Fluoride	Alkene	Alcohol	Ether	Bromide
1	300	12.7	6.8	0.0	0.0	82.9
2	600	19.6	9.1	0.0	0.0	74.0
3	900	23.1	10.1	0.0	0.0	66.3
4	1200	27.4	11.4	0.0	0.0	64.1
5	1500	31.2	12.7	0.0	0.0	57.7
6	1800	33.9	13.5	0.0	0.0	52.9
7	2700	39.1	14.4	0.0	0.0	43.6
8	3600	45.4	16.4	0.0	0.0	43.3
9	7200	55.4	18.7	0.0	0.0	26.2

		Concentration (M), Trial 1				
Entry	t (min)	Fluoride	Alkene	Alcohol	Ether	Bromide
1	300	0.032	0.017	0.000	0.000	0.207
2	600	0.049	0.023	0.000	0.000	0.185
3	900	0.058	0.025	0.000	0.000	0.166
4	1200	0.068	0.028	0.000	0.000	0.160
5	1500	0.078	0.032	0.000	0.000	0.144
6	1800	0.085	0.034	0.000	0.000	0.132
7	2700	0.098	0.036	0.000	0.000	0.109
8	3600	0.114	0.041	0.000	0.000	0.108
9	7200	0.139	0.047	0.000	0.000	0.065

[a] The % composition was determined by ^{1}H NMR analysis of the crude reaction mixture.

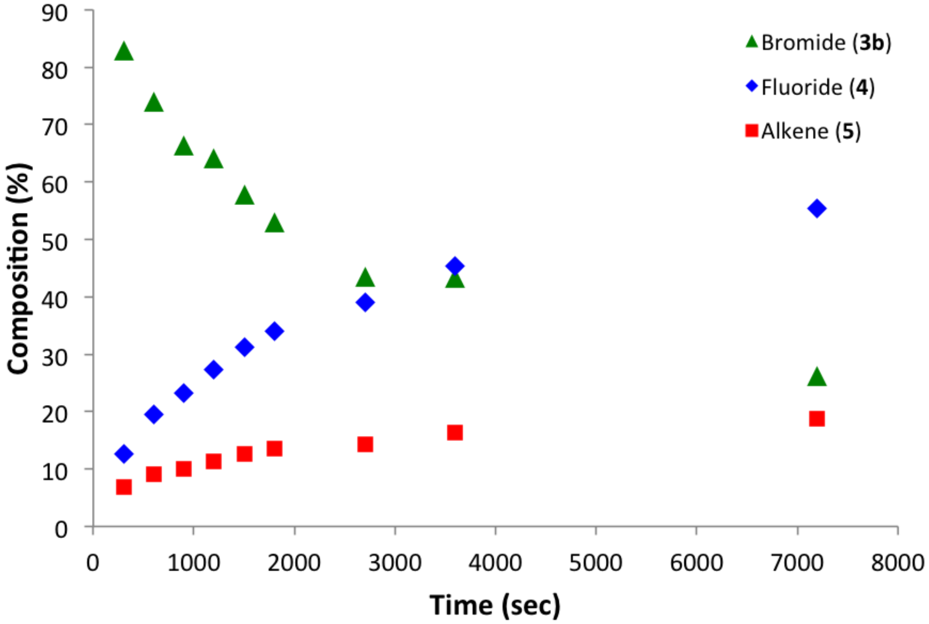

Figure S24: Rate profile with TEAF(NPG)$_2$.

REACTIVITY IN TOLUENE

Rate profile measurements with [tetrabutylammonium fluoride–(pinacol)$_2$], [tetrabutylammonium fluoride-(*p*-Tol$_3$COH)$_2$] and [tetrabutylammonium fluoride–trihydrate] complexes:

To a 14 mL screw-top vial, were added **3b** (53.0 mg, 0.2 mmol), PhMe (0.8 mL), and the [tetrabutylammonium fluoride–alcohol] complex (0.4 mmol). The vial was capped with a rubber septum, then stirred at 70 °C for 2 h. At the indicated time points, a small aliquot (< 80 μL) was taken with a syringe and needle. Each aliquot was filtered through a short plug of silica gel to quench the reaction, and the silica gel was washed with EtOAc (3 × 2 mL). The combined rinsings were concentrated *in vacuo*, and the resulting residue was analyzed by ^{1}H NMR. The conversion at each time point was determined by integration of the methylene/allylic proton signals depicted in Figure S1. The resulting data were plotted, and linear regression of the first three to five time points established the initial rate. The results are summarized in Table S15. Details for each individual complex are shown in Tables S16–S18 and Figures S27–S30.

Table S15: Summary of kinetic data and reactivity studies.[a]

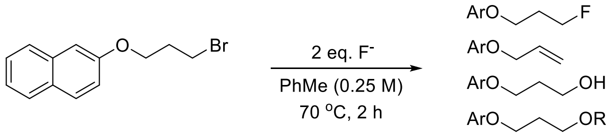

			Reaction Composition after 2 h (%, ^{1}H NMR)				
Entry	Fluoride Complex	Solvent	Fluoride	Olefin	Alcohol	Ether	Bromide
1	TBAF(*p*-Tol$_3$COH)$_2$	PhMe	78	20	2	0	0
2	TBAF(H$_2$O)$_3$	PhMe	59	16	25	0	0
3	TBAF(Pin)$_2$	PhMe	52	8	0	0	40
4	TBAF(*p*-Tol$_3$COH)$_2$	MeCN	67	33	0	0	0
5	TBAF(H$_2$O)$_3$	MeCN	59	33	8	0	0
6	TBAF(Pin)$_2$	MeCN	67	23	0	0	10

[a] The % composition was determined by ^{1}H NMR analysis of the crude reaction mixture. Values in MeCN (reproduced from above) represent average of three independent trials.

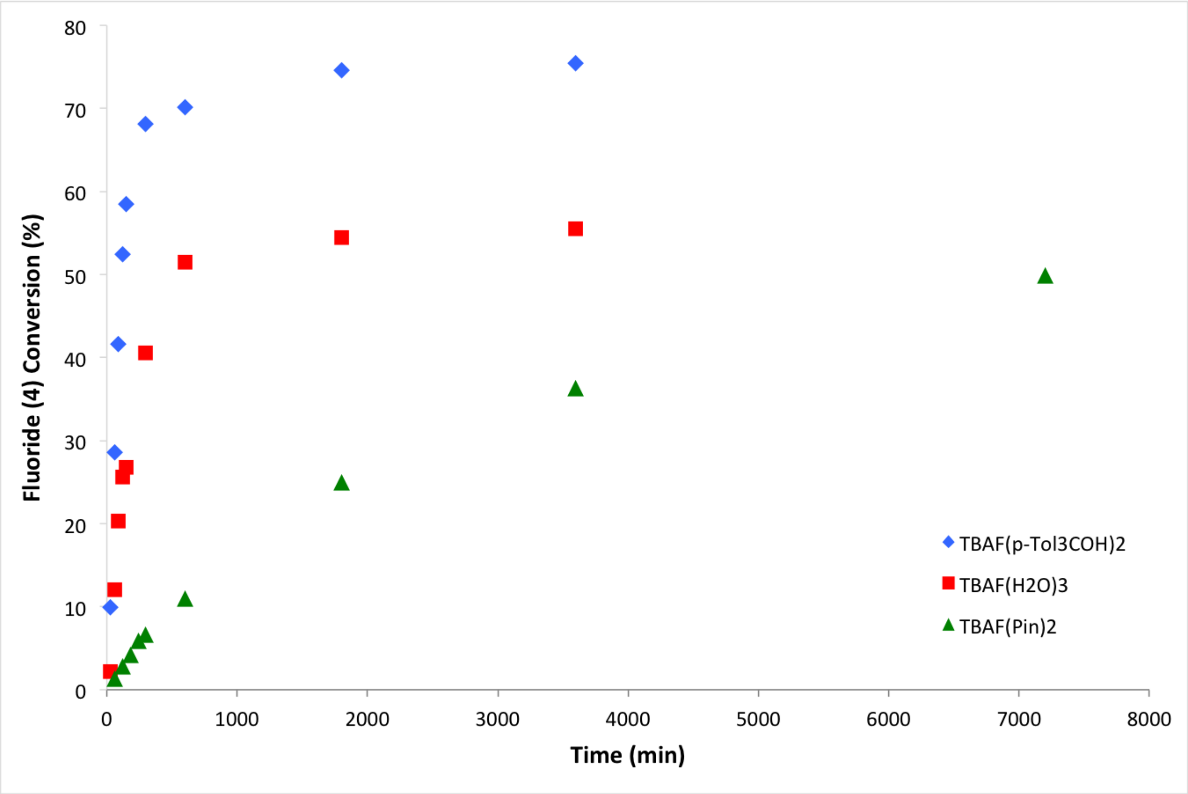

Figure S25: Summary of rate profiles for fluoride product (**4**) formation for [fluoride–alcohol] complexes examined.

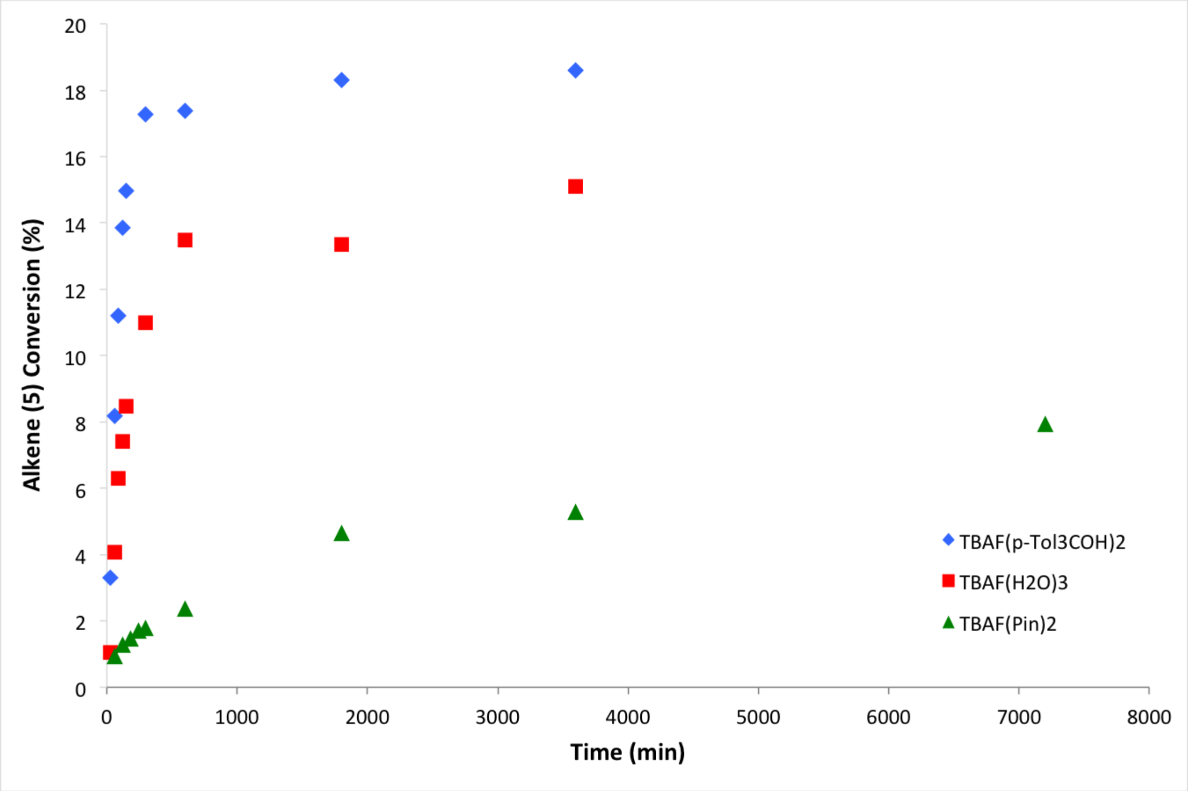

Figure S26: Summary of rate profiles for olefin product (**5**) formation for [fluoride–alcohol] complexes examined.

Table S16: Kinetic measurements using TBAF(*p*-Tol$_3$COH)$_2$ in PhMe.[a]

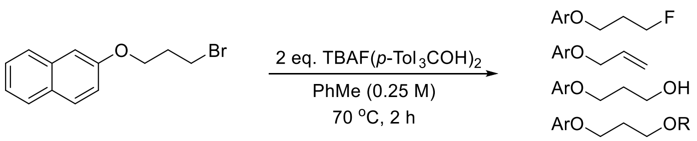

		Composition (%), Trial 1				
Entry	t (sec)	Fluoride	Alkene	Alcohol	Ether	Bromide
1	30	9.9	3.3	0.0	0.0	79.0
2	60	28.5	8.2	0.0	0.0	52.2
3	90	41.6	11.2	0.0	0.0	35.1
4	120	52.4	13.8	0.0	0.0	26.5
5	150	58.4	15.0	0.0	0.0	18.9
6	300	68.1	17.3	0.0	0.0	6.7
7	600	70.1	17.4	0.0	0.0	0.7
8	1800	74.6	18.3	0.0	0.0	0.0
9	3600	75.5	18.6	0.0	0.0	0.0

		Concentration (M), Trial 1				
Entry	t (sec)	Fluoride	Alkene	Alcohol	Ether	Bromide
1	30	0.025	0.008	0.000	0.000	0.198
2	60	0.071	0.020	0.000	0.000	0.130
3	90	0.104	0.028	0.000	0.000	0.088
4	120	0.131	0.035	0.000	0.000	0.066
5	150	0.146	0.037	0.000	0.000	0.047
6	300	0.170	0.043	0.000	0.000	0.017
7	600	0.175	0.043	0.000	0.000	0.002
8	1800	0.187	0.046	0.000	0.000	0.000
9	3600	0.189	0.047	0.000	0.000	0.000

[a] The % composition was determined by ^{1}H NMR analysis of the crude reaction mixture.

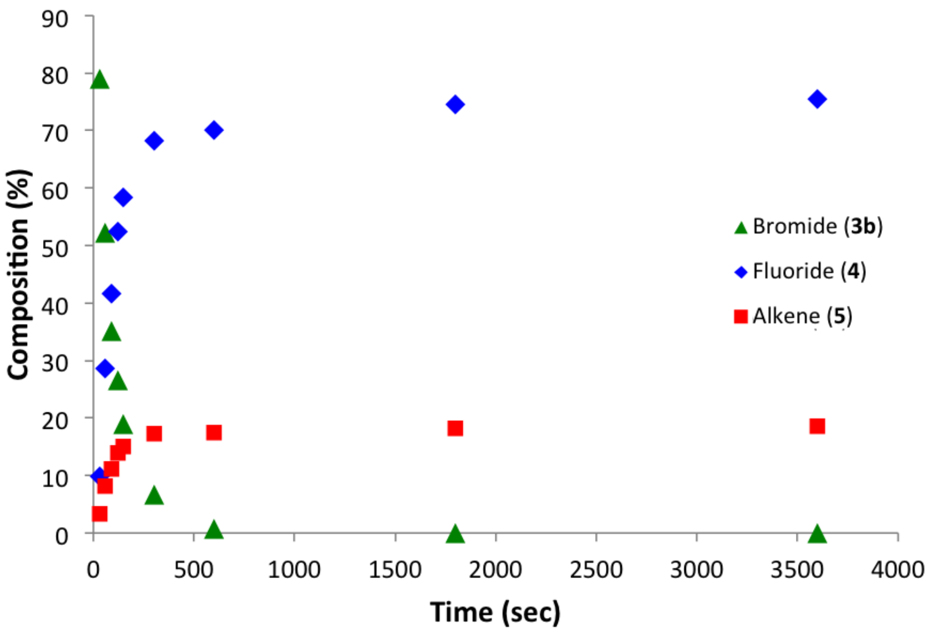

Figure S27: Rate profile with TBAF(*p*-Tol$_3$COH)$_2$ in PhMe.

Table S17: Kinetic measurements using TBAF$(H_2O)_3$ in PhMe.[a]

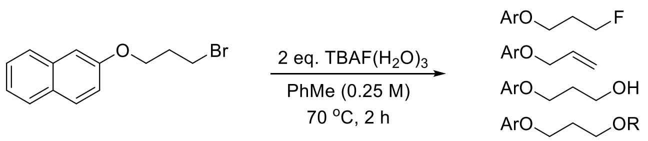

		Composition (%), Trial 1				
Entry	t (sec)	Fluoride	Alkene	Alcohol	Ether	Bromide
1	30	2.2	1.1	1.1	0.0	90.5
2	60	12.0	4.1	3.6	0.0	75.1
3	90	20.3	6.3	5.2	0.0	62.2
4	120	25.6	7.4	8.3	0.0	53.3
5	150	26.7	8.5	9.2	0.0	44.5
6	300	40.5	11.0	11.7	0.0	30.2
7	600	51.5	13.5	15.2	0.0	13.9
8	1800	54.4	13.3	24.4	0.0	1.4
9	3600	55.5	15.1	22.0	0.0	0.1

		Concentration (M), Trial 1				
Entry	t (sec)	Fluoride	Alkene	Alcohol	Ether	Bromide
1	30	0.006	0.003	0.003	0.000	0.226
2	60	0.030	0.010	0.009	0.000	0.188
3	90	0.051	0.016	0.013	0.000	0.155
4	120	0.064	0.019	0.021	0.000	0.133
5	150	0.067	0.021	0.023	0.000	0.111
6	300	0.101	0.027	0.029	0.000	0.075
7	600	0.129	0.034	0.038	0.000	0.035
8	1800	0.136	0.033	0.061	0.000	0.004
9	3600	0.139	0.038	0.055	0.000	0.000

[a] The % composition was determined by ^{1}H NMR analysis of the crude reaction mixture.

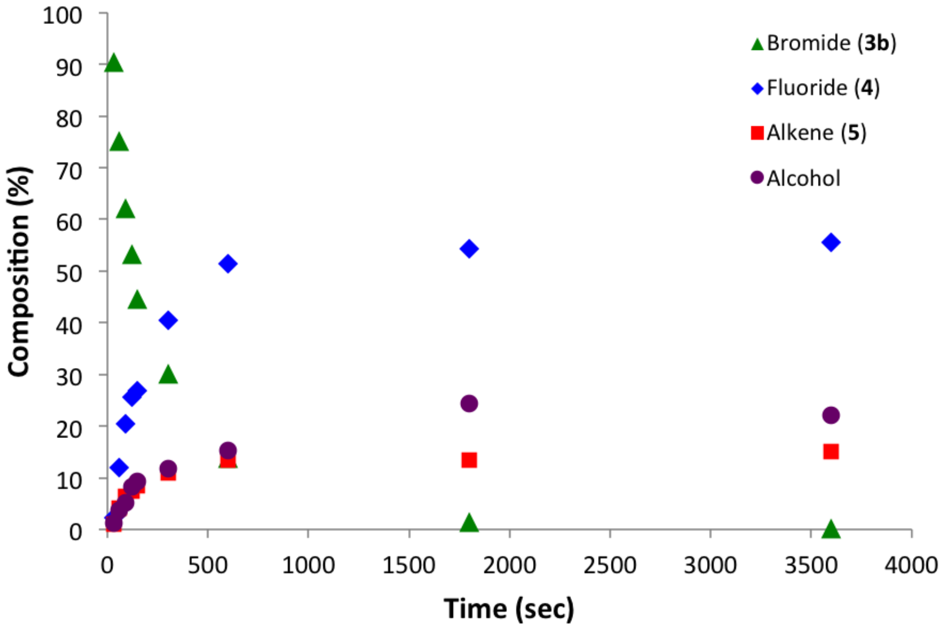

Figure S28: Rate profile with TBAF$(H_2O)_3$ in PhMe

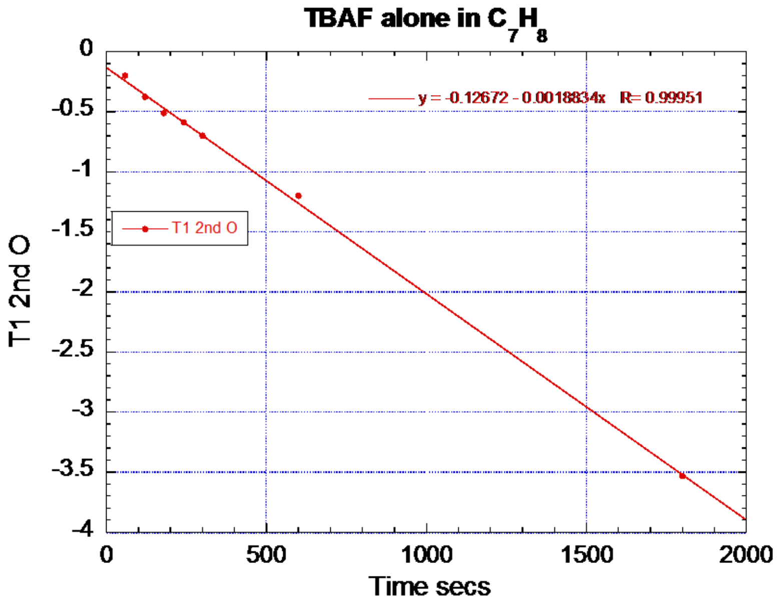

Figure S29: 2nd order plot for the reaction with TBAF(H_2O)$_3$ in PhMe.

Table S18: Kinetic measurements using TBAF(Pin)$_2$ in PhMe.[a]

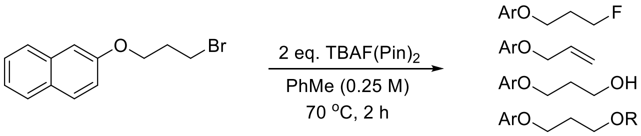

		Composition (%), Trial 1				
Entry	t (sec)	Fluoride	Alkene	Alcohol	Ether	Bromide
1	60	1.3	0.9	0.0	0.0	94.8
2	120	2.9	1.3	0.0	0.0	92.6
3	180	4.2	1.5	0.0	0.0	90.7
4	240	5.9	1.7	0.0	0.0	89.0
5	300	6.7	1.8	0.0	0.0	87.2
6	600	11.0	2.4	0.0	0.0	81.2
7	1800	25.0	4.7	0.0	0.0	65.6
8	3600	36.4	5.3	0.0	0.0	53.3
9	7200	49.9	7.9	0.0	0.0	37.0

		Concentration (M), Trial 1				
Entry	t (sec)	Fluoride	Alkene	Alcohol	Ether	Bromide
1	60	0.003	0.002	0.000	0.000	0.237
2	120	0.007	0.003	0.000	0.000	0.231
3	180	0.010	0.004	0.000	0.000	0.227
4	240	0.015	0.004	0.000	0.000	0.223
5	300	0.017	0.004	0.000	0.000	0.218
6	600	0.028	0.006	0.000	0.000	0.203
7	1800	0.062	0.012	0.000	0.000	0.164
8	3600	0.091	0.013	0.000	0.000	0.133
9	7200	0.125	0.020	0.000	0.000	0.093

[a] The % composition was determined by ^{1}H NMR analysis of the crude reaction mixture.

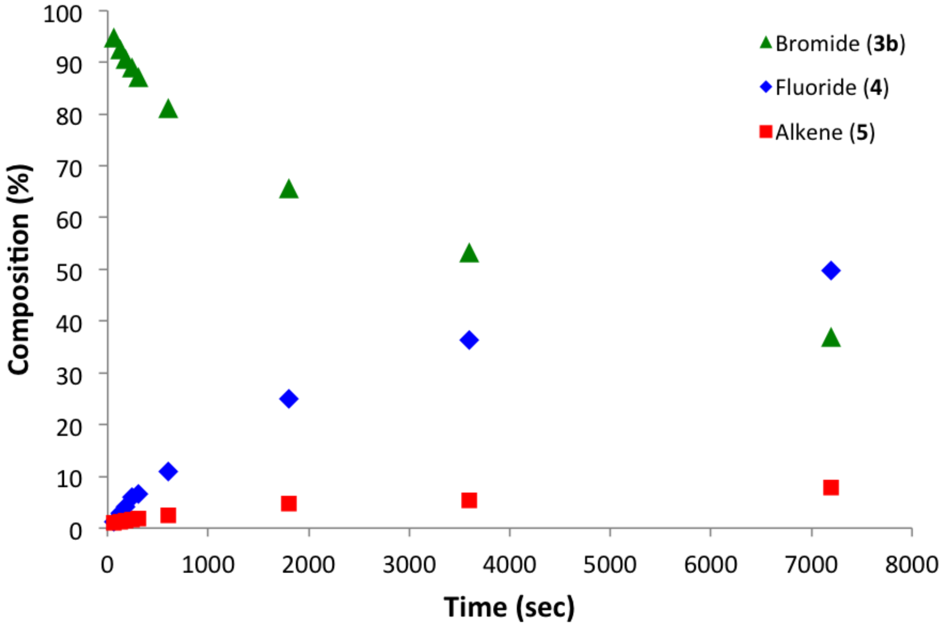

Figure S30: Rate profile with TBAF(Pin)$_2$ (**2g**) in PhMe.

CONCENTRATION STUDIES

Rate profile measurements with [tetrabutylammonium fluoride–(*p*-Tol$_3$COH)$_2$] complex at different concentrations and with added *p*-Tol$_3$COH:

To a 14 mL screw-top vial, were added **3b** (53.0 mg, 0.2 mmol), MeCN (0.8/3.2/8.0 mL) and [tetrabutylammonium–fluoride-(*p*-Tol$_3$COH)$_2$] complex **2m** (0.4 mmol). (Additional *p*-Tol$_3$COH (0.4 mmol) was added in the first reaction). The vial was capped with a rubber septum, then stirred at 70 °C for 2 h. At the indicated time points, a small aliquot (< 80 μL) was taken with a syringe and needle. Each aliquot was filtered through a short plug of silica gel to quench the reaction, and the silica gel was washed with EtOAc (3 × 2 mL). The combined rinsings were concentrated *in vacuo*, and the resulting residue was analyzed by ^{1}H NMR. The conversion at each time point was determined by integration of the methylene/allylic proton signals depicted in Figure S1. The results are summarized in Table S19. Details for each individual complex are shown in Tables S20–S22 and Figures S33–S37.

Table S19: Summary of kinetic data and reactivity studies.[a]

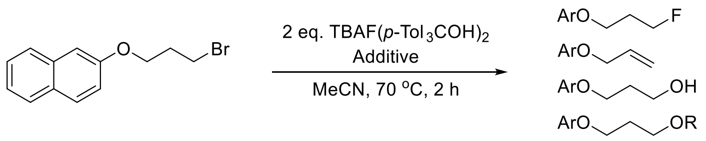

			Reaction Composition after 2 h (%, ^{1}H NMR)				
Entry	Conc. (M)	Additive	Fluoride	Olefin	Alcohol	Ether	Bromide
1	0.25	4 eq. *p*-Tol$_3$COH	79	21	0	0	0
2	0.25	---	67	33	0	0	0
3	0.0625	---	51	49	0	0	0
4	0.025	---	43	57	0	0	0

[a] The % composition was determined by ^{1}H NMR analysis of the crude reaction mixture. Values for 0.25 M (additive free) represent average of three independent trials (reproduced from above). Other values are the result of single trials.

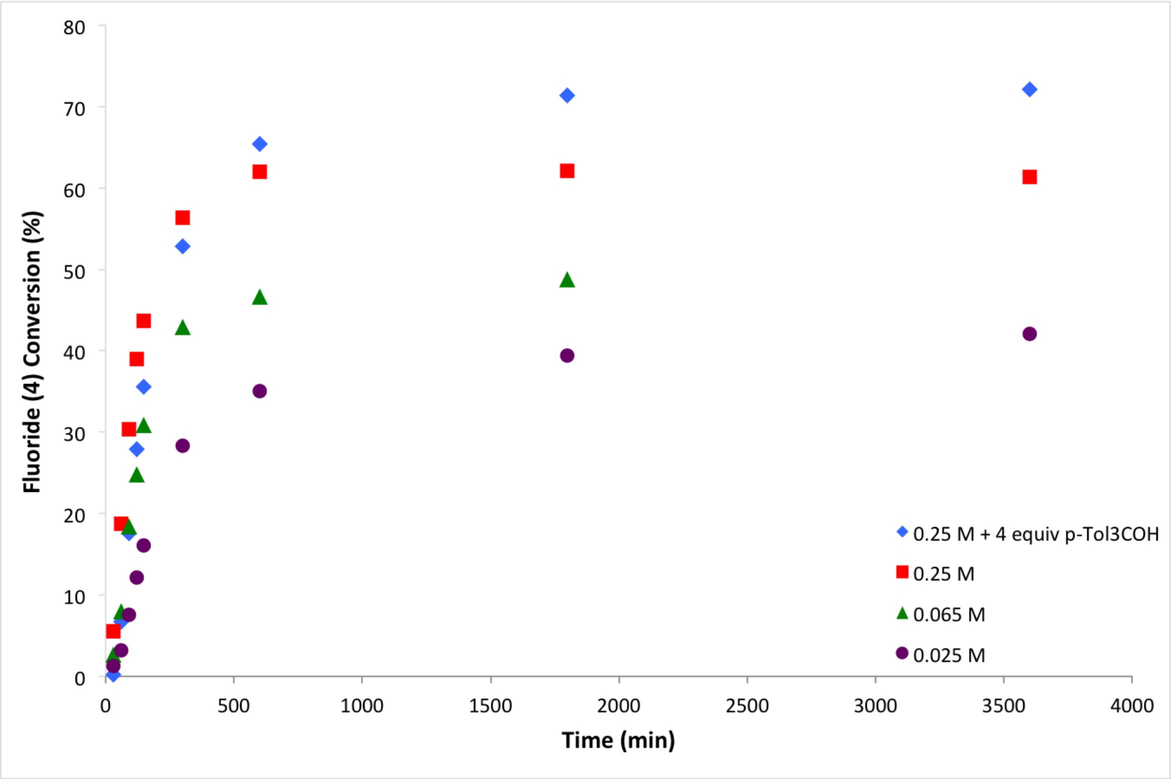

Figure S31: Summary of rate profiles for fluoride product (**4**) formation with TBAF(*p*-Tol$_3$COH)$_2$ in MeCN at different concentrations. Values for 0.25 M (additive free) represent average of three independent trials (reproduced from above). Other values represent single trials.

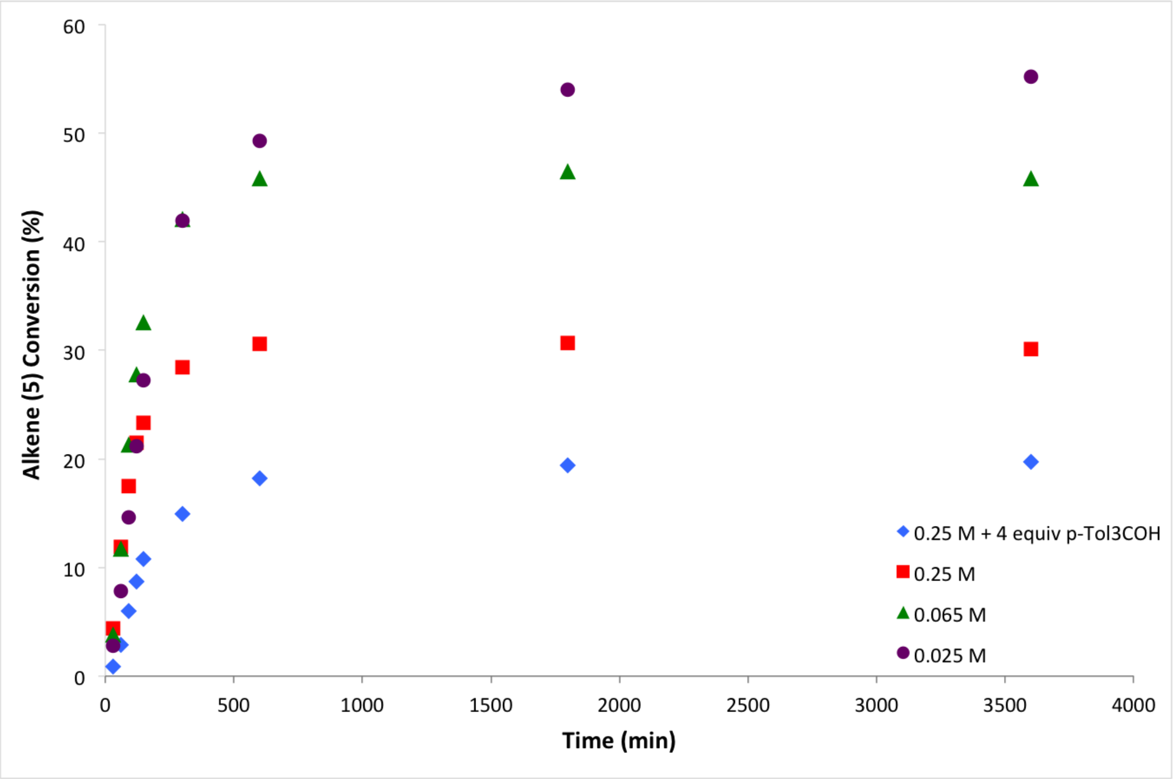

Figure S32: Summary of rate profiles for olefin product (**5**) formation with TBAF(*p*-Tol$_3$COH)$_2$ in MeCN at different concentrations. Values for 0.25 M (additive free) represent average of three independent trials (reproduced from above). Other values represent single trials.

Table S20: Kinetic measurements using TBAF(p-Tol$_3$COH)$_2$ in MeCN (0.25 M) with additional p-Tol$_3$COH (4 eq.).[a]

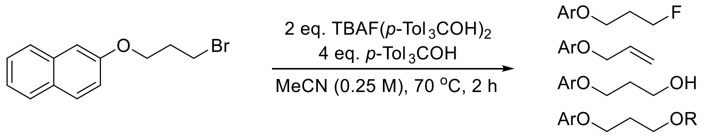

		Composition (%), Trial 1				
Entry	t (sec)	Fluoride	Alkene	Alcohol	Ether	Bromide
1	30	0.2	0.9	0.0	0.0	86.7
2	60	6.7	2.9	0.0	0.0	78.7
3	90	17.5	6.0	0.0	0.0	63.7
4	120	27.9	8.7	0.0	0.0	53.7
5	150	35.6	10.8	0.0	0.0	43.9
6	300	52.9	14.9	0.0	0.0	21.2
7	600	65.4	18.2	0.0	0.0	6.8
8	1800	71.3	19.4	0.0	0.0	0.0
9	3600	72.1	19.7	0.0	0.0	0.0

		Concentration (M), Trial 1				
Entry	t (sec)	Fluoride	Alkene	Alcohol	Ether	Bromide
1	30	0.000	0.002	0.000	0.000	0.217
2	60	0.017	0.007	0.000	0.000	0.197
3	90	0.044	0.015	0.000	0.000	0.159
4	120	0.070	0.022	0.000	0.000	0.134
5	150	0.089	0.027	0.000	0.000	0.110
6	300	0.132	0.037	0.000	0.000	0.053
7	600	0.163	0.046	0.000	0.000	0.017
8	1800	0.178	0.049	0.000	0.000	0.000
9	3600	0.180	0.049	0.000	0.000	0.000

[a] The % composition was determined by ^{1}H NMR analysis of the crude reaction mixture.

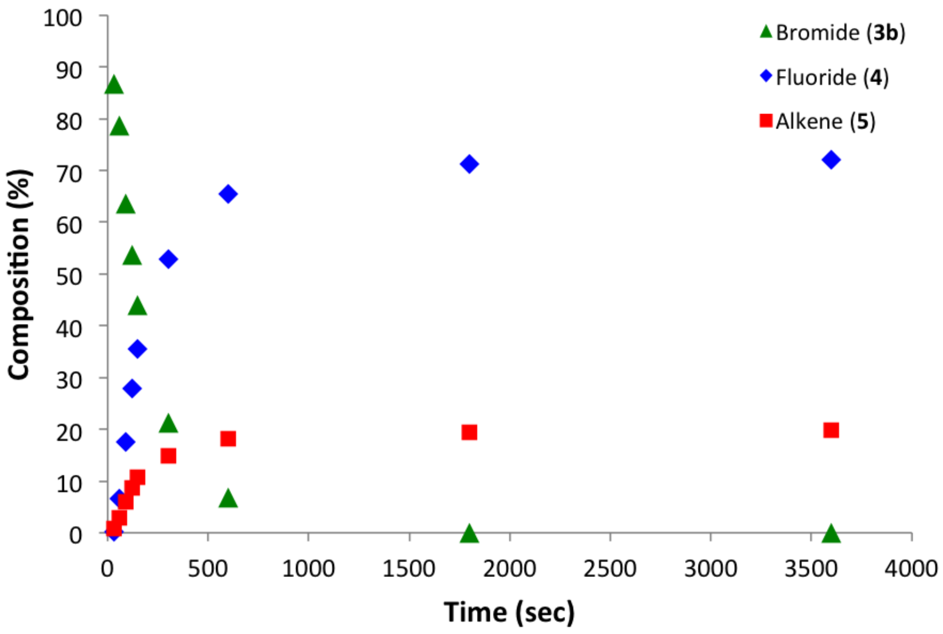

Figure S33: Rate profile with TBAF(p-Tol$_3$COH)$_2$ in MeCN (0.25 M) with additional p-Tol$_3$COH (4 eq.).

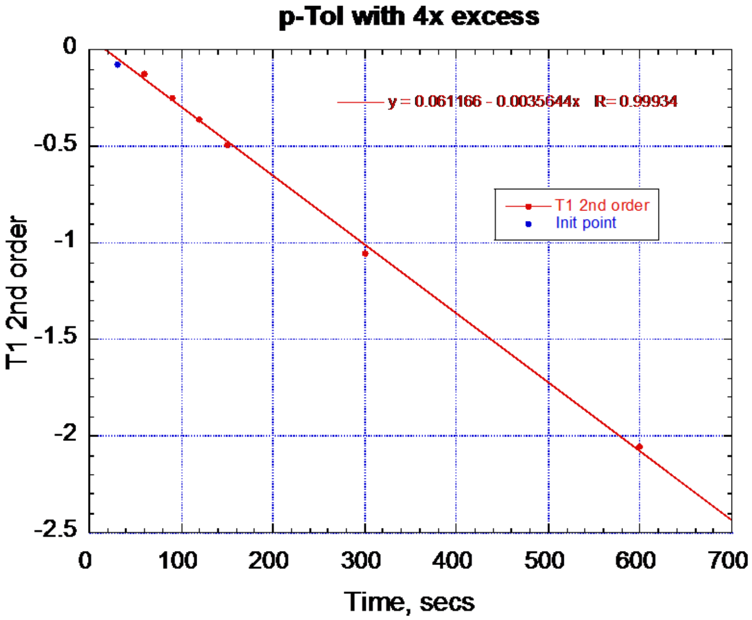

Figure S34: 2nd order plot for the reaction with TBAF(*p*-$Tol_3COH)_2$ in MeCN (0.25 M) with additional *p*-Tol_3COH (4 eq.).

Table S21: Kinetic measurements using TBAF(p-$Tol_3COH)_2$ in MeCN (0.0625 M).[a]

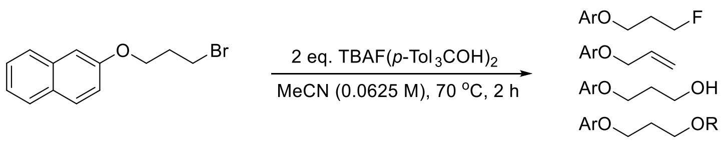

		Composition (%), Trial 1				
Entry	t (sec)	Fluoride	Alkene	Alcohol	Ether	Bromide
1	30	2.6	3.8	0.0	0.0	87.9
2	60	7.9	11.8	0.0	0.0	71.8
3	90	18.4	21.3	0.0	0.0	54.3
4	120	24.8	27.8	0.0	0.0	39.0
5	150	30.9	32.6	0.0	0.0	29.3
6	300	42.9	42.1	0.0	0.0	10.4
7	600	46.6	45.8	0.0	0.0	0.0
8	1800	48.8	46.5	0.0	0.0	0.0
9	3600	47.4	45.9	0.0	0.0	0.0

		Concentration (M), Trial 1				
Entry	t (sec)	Fluoride	Alkene	Alcohol	Ether	Bromide
1	30	0.0017	0.0024	0.0000	0.0000	0.0550
2	60	0.0050	0.0073	0.0000	0.0000	0.0449
3	90	0.0115	0.0133	0.0000	0.0000	0.0340
4	120	0.0155	0.0174	0.0000	0.0000	0.0244
5	150	0.0193	0.0204	0.0000	0.0000	0.0183
6	300	0.0268	0.0263	0.0000	0.0000	0.0065
7	600	0.0292	0.0286	0.0000	0.0000	0.0000
8	1800	0.0305	0.0290	0.0000	0.0000	0.0000
9	3600	0.0296	0.0287	0.0000	0.0000	0.0000

[a] The % composition was determined by ^{1}H NMR analysis of the crude reaction mixture.

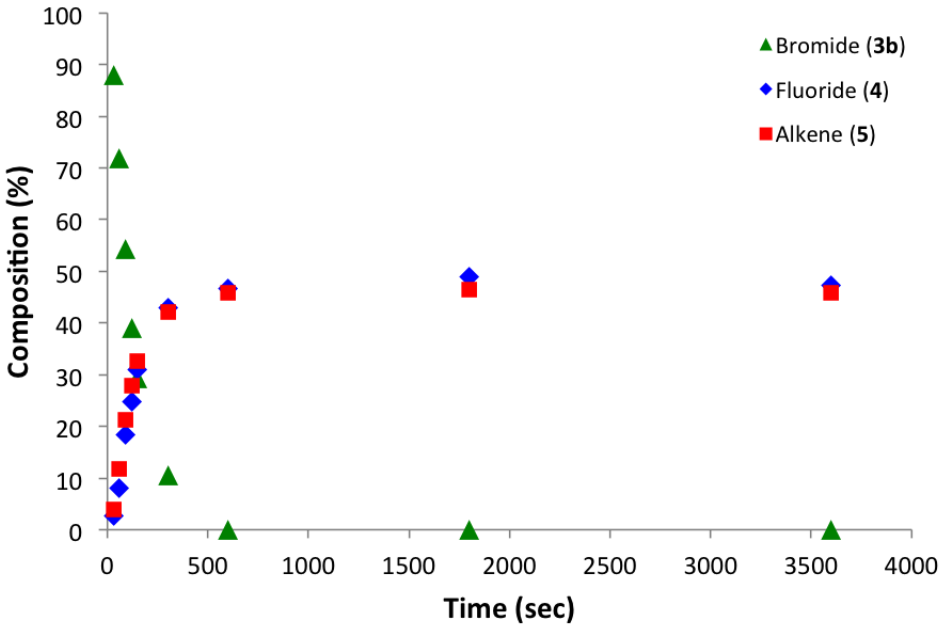

Figure S35: Rate profile with with TBAF(p-$Tol_3COH)_2$ in MeCN (0.0625 M).

Table S22: Kinetic measurements using TBAF(p-Tol$_3$COH)$_2$ in MeCN (0.025 M).[a]

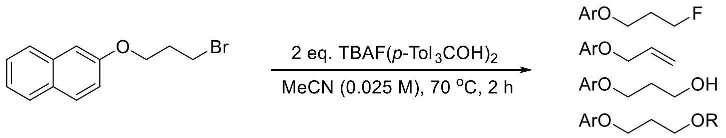

		Composition (%), Trial 1				
Entry	t (sec)	Fluoride	Alkene	Alcohol	Ether	Bromide
1	30	1.3	2.8	0.0	0.0	90.0
2	60	3.2	7.8	0.0	0.0	81.6
3	90	7.5	14.6	0.0	0.0	71.8
4	120	12.1	21.2	0.0	0.0	60.3
5	150	16.1	27.2	0.0	0.0	51.3
6	300	28.3	41.9	0.0	0.0	25.2
7	600	35.1	49.3	0.0	0.0	10.6
8	1800	39.4	54.0	0.0	0.0	0.7
9	3600	42.1	55.2	0.0	0.0	0.0

		Concentration (M), Trial 1				
Entry	t (sec)	Fluoride	Alkene	Alcohol	Ether	Bromide
1	30	0.0003	0.0007	0.0000	0.0000	0.0225
2	60	0.0008	0.0020	0.0000	0.0000	0.0204
3	90	0.0019	0.0036	0.0000	0.0000	0.0180
4	120	0.0030	0.0053	0.0000	0.0000	0.0151
5	150	0.0040	0.0068	0.0000	0.0000	0.0128
6	300	0.0071	0.0105	0.0000	0.0000	0.0063
7	600	0.0088	0.0123	0.0000	0.0000	0.0026
8	1800	0.0098	0.0135	0.0000	0.0000	0.0002
9	3600	0.0105	0.0138	0.0000	0.0000	0.0000

[a] The % composition was determined by ^{1}H NMR analysis of the crude reaction mixture.

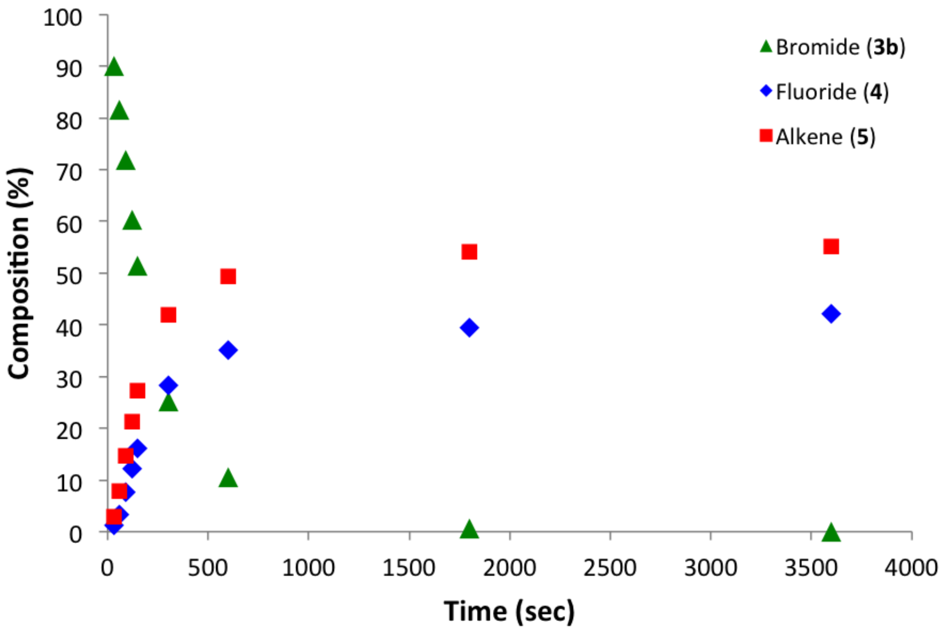

Figure S36: Rate profile with with TBAF(p-Tol$_3$COH)$_2$ in MeCN (0.025 M).

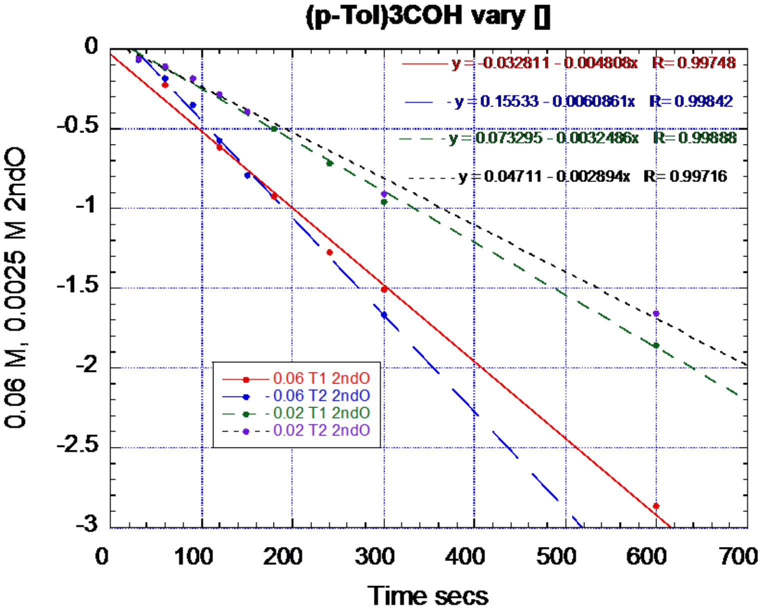

Figure S37: 2nd order plot for the reaction with TBAF(p-$Tol_3COH)_2$ in MeCN (0.0625 M, 0.025 M).

TEMPERATURE STUDIES

Rate profile measurements with [tetrabutylammonium fluoride–(*p*-Tol$_3$COH)$_2$] complex at different temperatures:

To a 14 mL screw-top vial, were added **3b** (53.0 mg, 0.2 mmol), MeCN (0.8 mL) and [tetrabutylammonium–fluoride-(*p*-Tol$_3$COH)$_2$] complex **2m** (0.4 mmol). The vial was capped with a rubber septum, then stirred at 50–70 °C for 2 h. At the indicated time points, a small aliquot (< 80 μL) was taken with a syringe and needle. Each aliquot was filtered through a short plug of silica gel to quench the reaction, and the silica gel was washed with EtOAc (3 × 2 mL). The combined rinsings were concentrated *in vacuo*, and the resulting residue was analyzed by ^{1}H NMR. The conversion at each time point was determined by integration of the methylene/allylic proton signals depicted in Figure S1. The results are summarized in Table S23. Details for each individual complex are shown in Tables S24–S25 and Figures S40–S41.

Table S23: Summary of kinetic data and reactivity studies.[a]

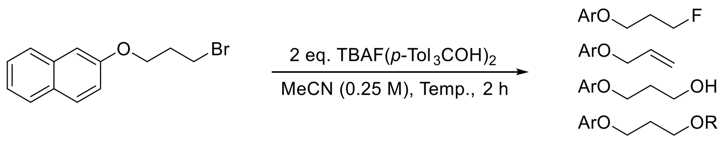

		Reaction Composition after 2 h (%, ^{1}H NMR)				
Entry	Temp. (°C)	Fluoride	Olefin	Alcohol	Ether	Bromide
1	50	72	28	0	0	0
2	60	69	31	0	0	0
3	70	67	33	0	0	0

[a] The % composition was determined by ^{1}H NMR analysis of the crude reaction mixture. Values for 70 °C represent average of three independent trials (reproduced from above). Other values are the result of single trials.

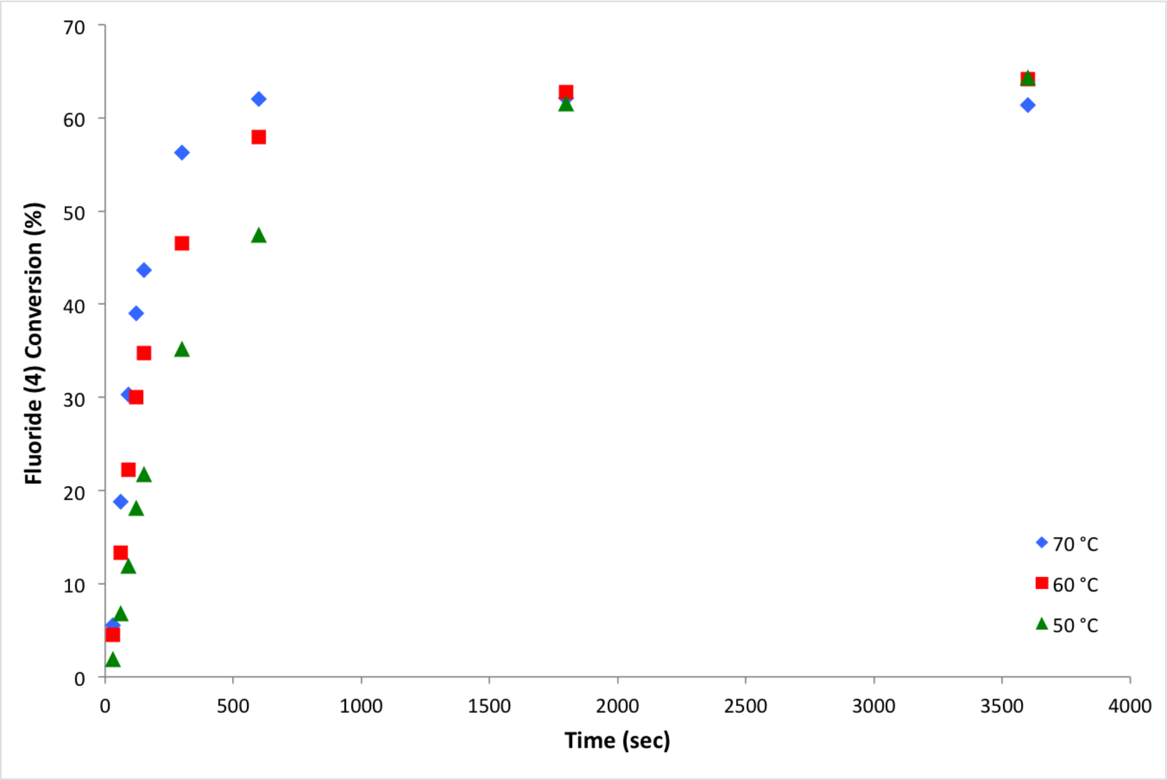

Figure S38: Summary of rate profiles for fluoride product (**4**) formation with TBAF(*p*-$Tol_3COH)_2$ in MeCN at different temperatures. Values for 70 °C represent average of three independent trials (reproduced from above). Other values represent single trials.

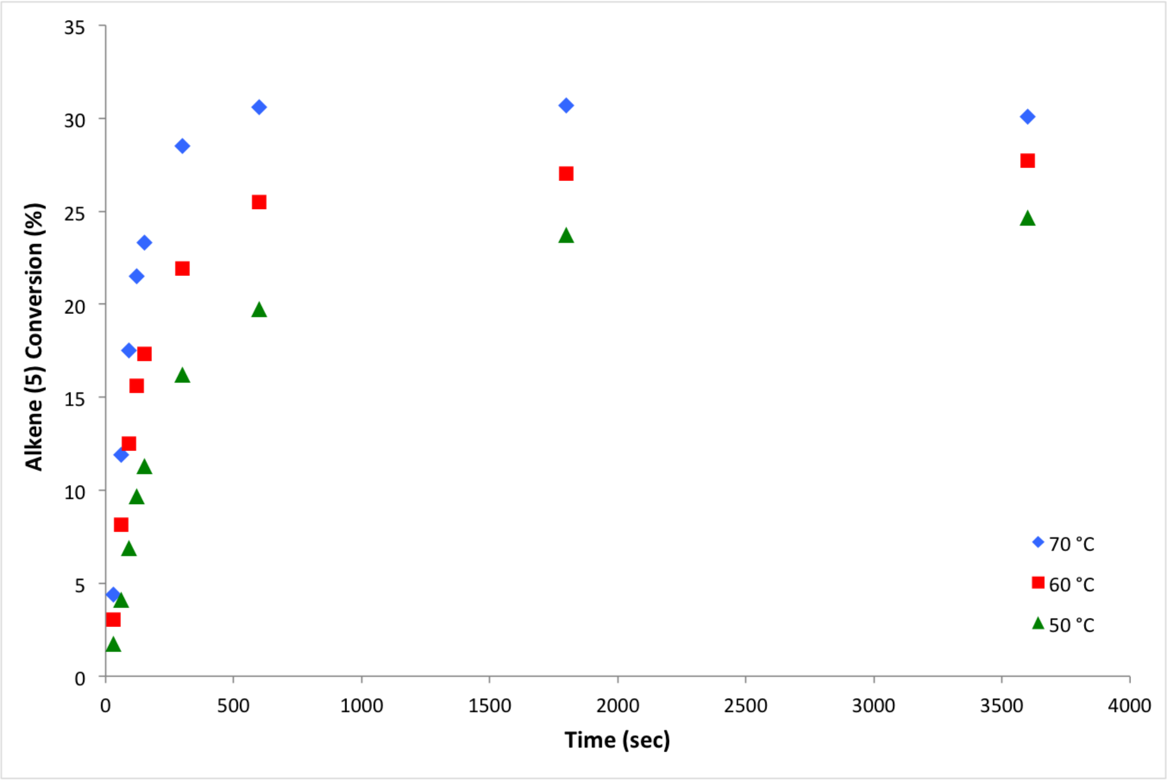

Figure S39: Summary of rate profiles for olefin product (**5**) formation with TBAF(*p*-$Tol_3COH)_2$ in MeCN at different temperatures. Values for 70 °C represent average of three independent trials (reproduced from above). Other values represent single trials.

Table S24: Kinetic measurements using TBAF(p-$Tol_3COH)_2$ in MeCN at 60 °C.[a]

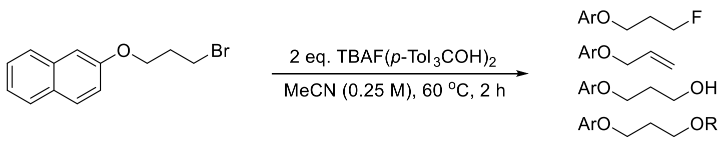

		Composition (%), Trial 1				
Entry	t (sec)	Fluoride	Alkene	Alcohol	Ether	Bromide
1	30	4.5	3.0	0.0	0.0	84.2
2	60	13.3	8.2	0.0	0.0	69.5
3	90	22.2	12.5	0.0	0.0	57.0
4	120	30.0	15.6	0.0	0.0	47.8
5	150	34.7	17.3	0.0	0.0	39.4
6	300	46.5	21.9	0.0	0.0	22.0
7	600	57.9	25.5	0.0	0.0	8.1
8	1800	62.7	27.0	0.0	0.0	0.3
9	3600	64.2	27.7	1.7	0.0	0.3

		Concentration (M), Trial 1				
Entry	t (sec)	Fluoride	Alkene	Alcohol	Ether	Bromide
1	30	0.011	0.008	0.000	0.000	0.210
2	60	0.033	0.020	0.000	0.000	0.174
3	90	0.056	0.031	0.000	0.000	0.142
4	120	0.075	0.039	0.000	0.000	0.120
5	150	0.087	0.043	0.000	0.000	0.099
6	300	0.116	0.055	0.000	0.000	0.055
7	600	0.145	0.064	0.000	0.000	0.020
8	1800	0.157	0.068	0.000	0.000	0.001
9	3600	0.160	0.069	0.004	0.000	0.001

[a] The % composition was determined by ^{1}H NMR analysis of the crude reaction mixture.

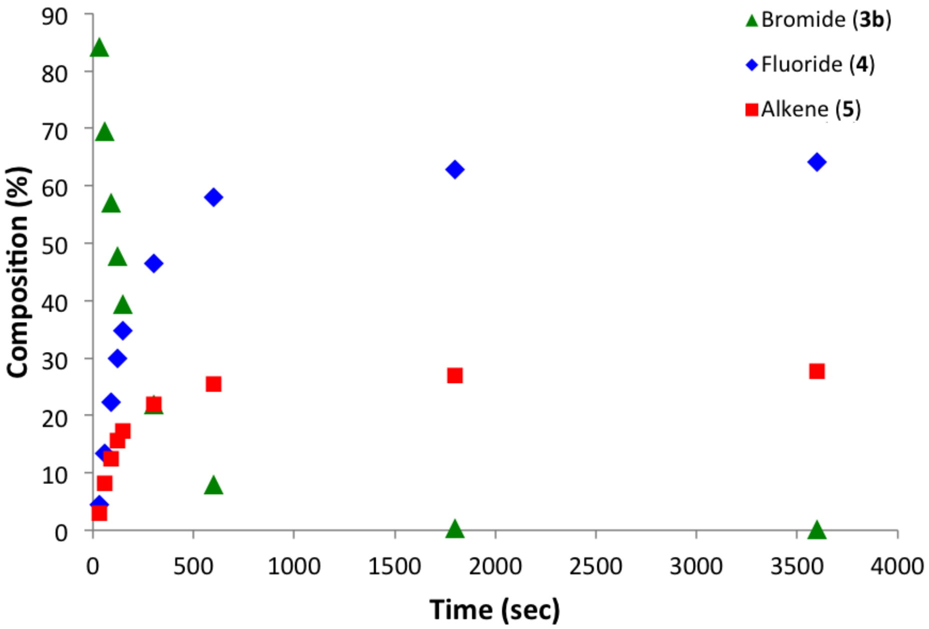

Figure S40: Rate profile with with TBAF(p-$Tol_3COH)_2$ in MeCN at 60 °C.

Table S25: Kinetic measurements using TBAF(*p*-$Tol_3COH)_2$ in MeCN at 50 °C.[a]

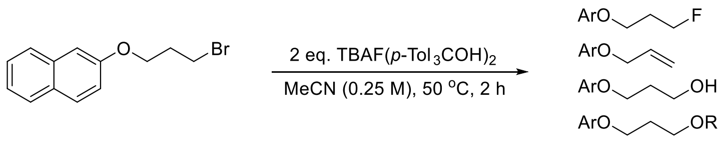

		Composition (%), Trial 1				
Entry	t (sec)	Fluoride	Alkene	Alcohol	Ether	Bromide
1	30	1.9	1.8	0.0	0.0	86.0
2	60	6.9	4.1	0.0	0.0	82.4
3	90	11.9	6.9	0.0	0.0	73.8
4	120	18.1	9.7	0.0	0.0	65.1
5	150	21.8	11.3	0.0	0.0	60.3
6	300	35.3	16.2	0.0	0.0	41.7
7	600	47.5	19.7	0.0	0.0	25.5
8	1800	61.6	23.7	0.0	0.0	6.4
9	3600	64.3	24.7	0.0	0.0	0.4

		Concentration (M), Trial 1				
Entry	t (sec)	Fluoride	Alkene	Alcohol	Ether	Bromide
1	30	0.005	0.004	0.000	0.000	0.215
2	60	0.017	0.010	0.000	0.000	0.206
3	90	0.030	0.017	0.000	0.000	0.185
4	120	0.045	0.024	0.000	0.000	0.163
5	150	0.054	0.028	0.000	0.000	0.151
6	300	0.088	0.041	0.000	0.000	0.104
7	600	0.119	0.049	0.000	0.000	0.064
8	1800	0.154	0.059	0.000	0.000	0.016
9	3600	0.161	0.062	0.000	0.000	0.001

[a] The % composition was determined by ^1H NMR analysis of the crude reaction mixture.

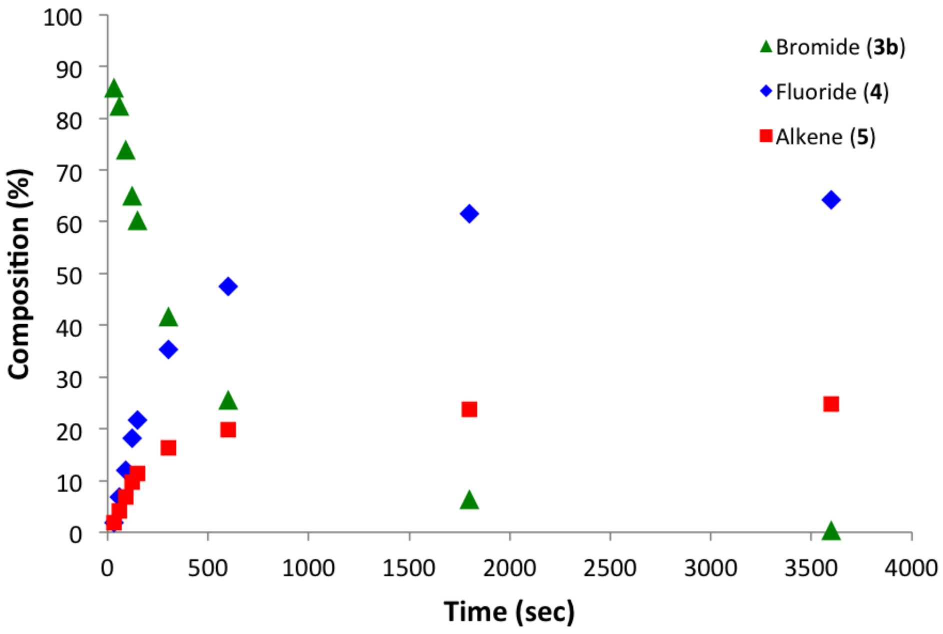

Figure S41: Rate profile with with TBAF(*p*-$Tol_3COH)_2$ in MeCN at 50 °C.

Alcohols as Additives

Prior to each experiment, CsF was first dried using the following procedure. CsF (approximately 200 mg) was added to a 100 mL two-necked round-bottom flask, with one neck outfitted with a rubber septum. The flask containing CsF was placed under high vacuum and heated with a heat gun (approximately 2 min) then allowed to cool to room temperature. This heat/cool cycle was repeated a total of three times. The flask was back-filled with Ar. While under a positive pressure of Ar, CsF (60.8 mg, 0.4 mmol) was quickly massed using a balance, while keeping the round bottom flask under a positive flow of Ar. The CsF was immediately added to a 14 mL screw-top vial containing **3b** (48.8 mg, 0.2 mmol) and MeCN (0.8 mL). The vial was capped and transferred to an oil bath, which had been pre-heated to 70 °C. After 24 h, the vial was removed from the oil bath and allowed to cool to room temperature. Et_2O (4 mL) and H_2O (4 mL) were added to quench the reaction. A sample of the organic phases was removed, and the solvent was removed *in vacuo*. The resulting residue was dissolved in $CDCl_3$ for analysis by ^1H NMR spectroscopy. The reaction composition was determined by integration of the methylene/allylic proton signals depicted in Figure S1. The results are summarized in Table S26.

Table S26: Reactivity data with CsF and alcohol additives.[a]

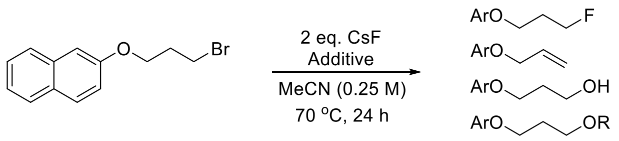

		Reaction Composition after 24 h (%, ^1H NMR)				
Entry	Additive	Fluoride	Olefin	Alcohol	Ether	Bromide
1	4 eq. *p*-Tol_3COH	79	17	0	0	4
2	4 eq. 9-phenyl-9-fluorenol	80	20	0	0	1
3	4 eq. *t*-BuOH	65	27	1	0	7
4	2 eq. Pin	58	11	1	0	31
5	2 eq. NPG	53	12	0	0	35
6	---	8	22	1	0	69

[a] The % composition was determined by ^1H NMR analysis of the crude reaction mixture.

References

(1) W. S. Jeon, E. Kim, Y. H. Ko, I. Hwang, J. W. Lee, S.-Y. Kim, H.-J. Kim and K. Kim, *Angew. Chem. Int. Ed.*, 2005, **44**, 87–91.

(2) J. Cosier and A. M. Glazer, *J. Appl. Cryst.*, 1986, **19**, 105–107.

(3) Z. Otwinowski and W. Minor, In *Methods in Enzymology*; C. W. Carter, Jr., R. M. Sweet, Eds.; Academic Press: New York, 1997; Vol. 276, pp 307–326.

(4) CrysAlisPro (Agilent Technologies, Oxford, 2011).

(5) A. Altomare, G. Cascarano, C. Giacovazzo, A. Guagliardi, M. C. Burla, G. Polidori and M. Camalli, *J. Appl. Crystallogr.*, 1994, **27**, 435.

(6) L. Palatinus and G. Chapuis, *J. Appl. Crystallogr.*, 2007, **40**, 786–790.

(7) P. W. Betteridge, J. R. Carruthers, R. I. Cooper, K. Prout and D. J. Watkin, *J. Appl. Crystallogr.*, 2003, **36**, 1487.

(8) P. Parois, R. I. Cooper and A. L. Thompson, *Chem. Cent.*, in press.

(9) A. L. Thompson and D. J. Watkin, *J. Appl. Cryst.*, 2011, **44**, 1017–1022.

(10) R. I. Cooper, A. L. Thompson and D. J. Watkin, *J. Appl. Crystallogr.*, 2010, **43**, 1100–1107.

(11) *(a)* A. L. Spek, *J. Appl. Cryst.*, 2003, **36**, 7–13; *(b)* P. van der Sluis and A. L. Spek, *Acta Cryst.*, 1990, **A46**, 194–201.

(12) R. I. Cooper, R. O. Gould, S. Parsons and D. J. Watkin, *J. Appl. Cryst.*, 2002, **35**, 168–174.

(13) A. L. Spek, *PLATON, A Multipurpose Crystallographic Tool*, Utrecht, the Netherlands, 1998.

(14) A. L. Spek, *J. Appl. Cryst.*, 2003, **36**, 7–13.

(15) G. M. Bennett and R. L. Wain, *J. Chem. Soc.*, 1936, 1114–1120.

(16) B. B. Elsner, H. E. Strauss and E. J. Forbes, *J. Chem. Soc.*, 1957, 578–582.

(17) G. Fraenkel, J. H. Duncan and J. Wang, *J. Am. Chem. Soc.*, 1999, **121**, 432–443.

(18) V. K. Aggarwal, F. Sandrinelli and J. P. H. Charmant, *Tetrahedron: Asymmetry*, 2002, **13**, 87–93.

(19) Z. Shi and C. He, *J. Am. Chem. Soc.*, 2004, **126**, 13596–13597.

(20) F. C. Gozzo, S. A. Fernandes, D. C. Rodrigues, M. N. Eberlin and A. J. Marsaioli, *J. Org. Chem.*, 2003, **68**, 5493–5499.

(21) D. W. Kim, H.-J. Jeong, S. T. Lim and M.-H. Sohn, *Angew. Chem. Int. Ed.*, 2008, **47**, 8404–8406.

(22) S. G. DiMagno and H. Sun, H. *J. Am. Chem. Soc.*, 2005, **127**, 2050–2051.

NMR Spectra

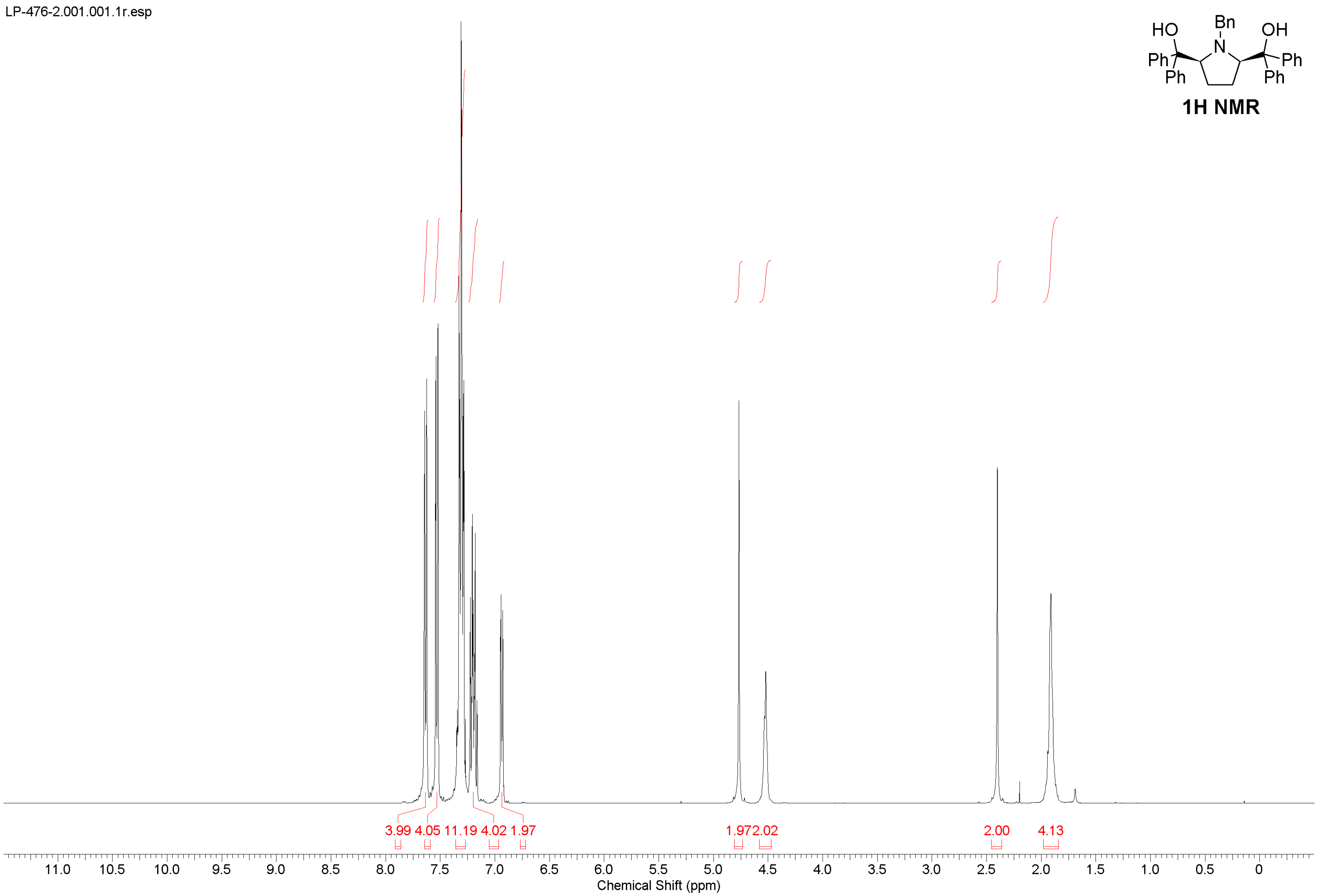
LP-476-2.001.001.1r.esp
HO
Bn
N
OH
Ph
Ph
Ph
Ph
1H NMR
3.99
4.05
11.19
4.02
1.97
1.97
2.02
2.00
4.13
11.0
10.5
10.0
9.5
9.0
8.5
8.0
7.5
7.0
6.5
6.0
5.5
5.0
4.5
4.0
3.5
3.0
2.5
2.0
1.5
1.0
0.5
0
Chemical Shift (ppm)

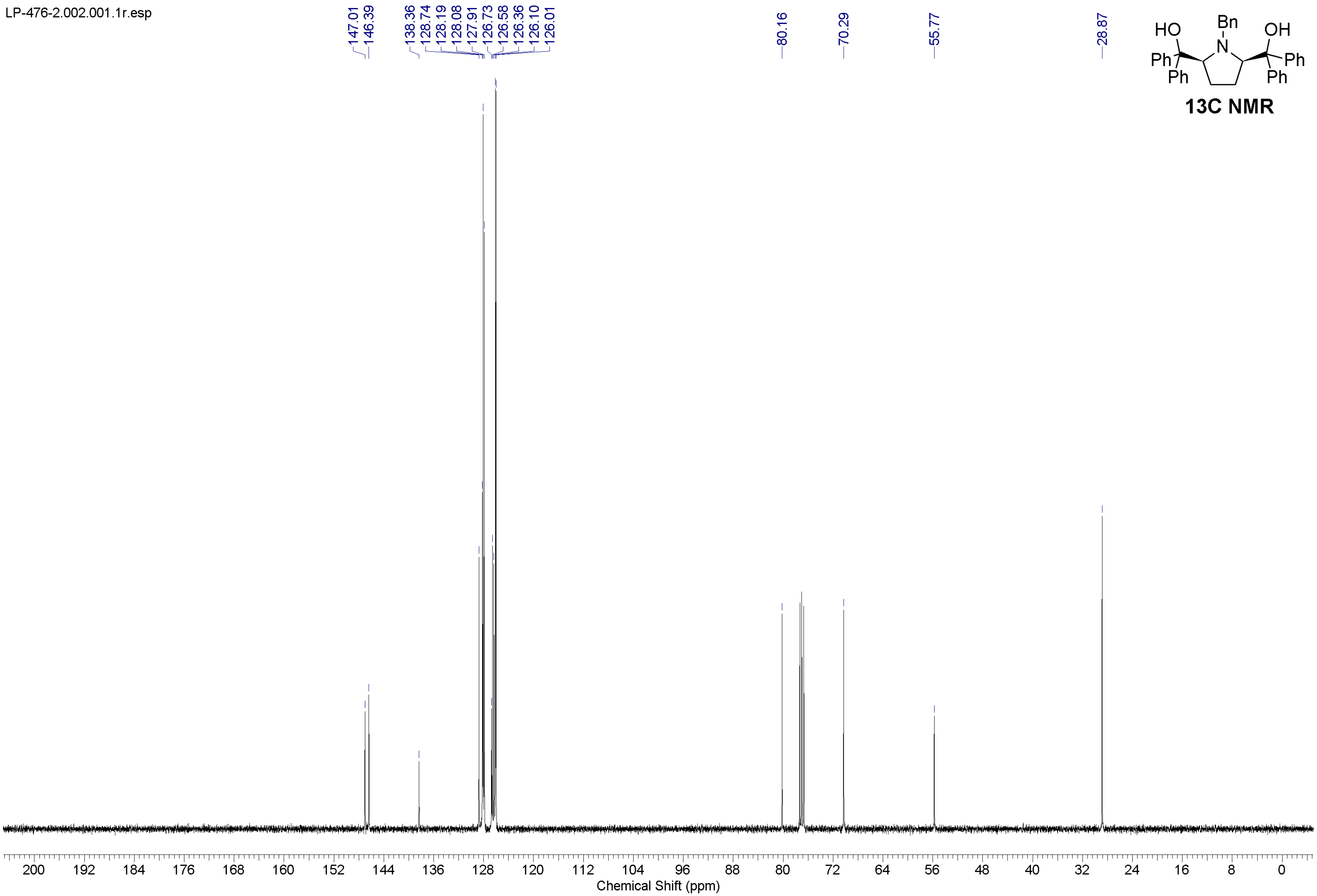
LP-476-2.002.001.1r.esp
147.01
146.39
138.36
128.74
128.19
128.08
127.91
126.73
126.58
126.36
126.10
126.01
80.16
70.29
55.77
28.87
HO
Bn
N
OH
Ph
Ph
Ph
Ph
13C NMR
200 192 184 176 168 160 152 144 136 128 120 112 104 96 88 80 72 64 56 48 40 32 24 16 8 0
Chemical Shift (ppm)

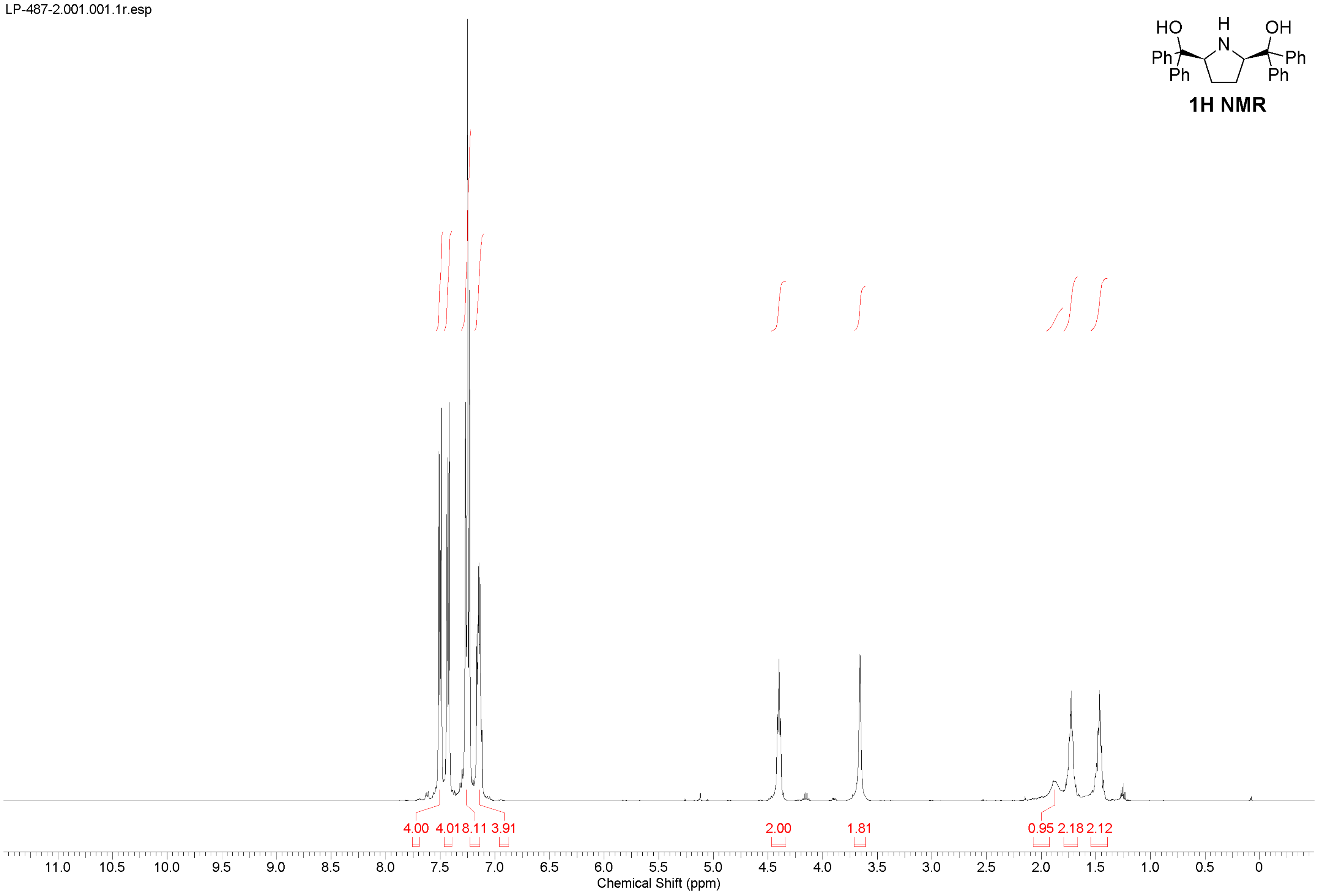
LP-487-2.001.001.1r.esp
HO
H
N
OH
Ph
Ph
Ph
Ph
1H NMR
4.00
4.01
8.11
3.91
2.00
1.81
0.95
2.18
2.12
11.0
10.5
10.0
9.5
9.0
8.5
8.0
7.5
7.0
6.5
6.0
5.5
5.0
4.5
4.0
3.5
3.0
2.5
2.0
1.5
1.0
0.5
0
Chemical Shift (ppm)

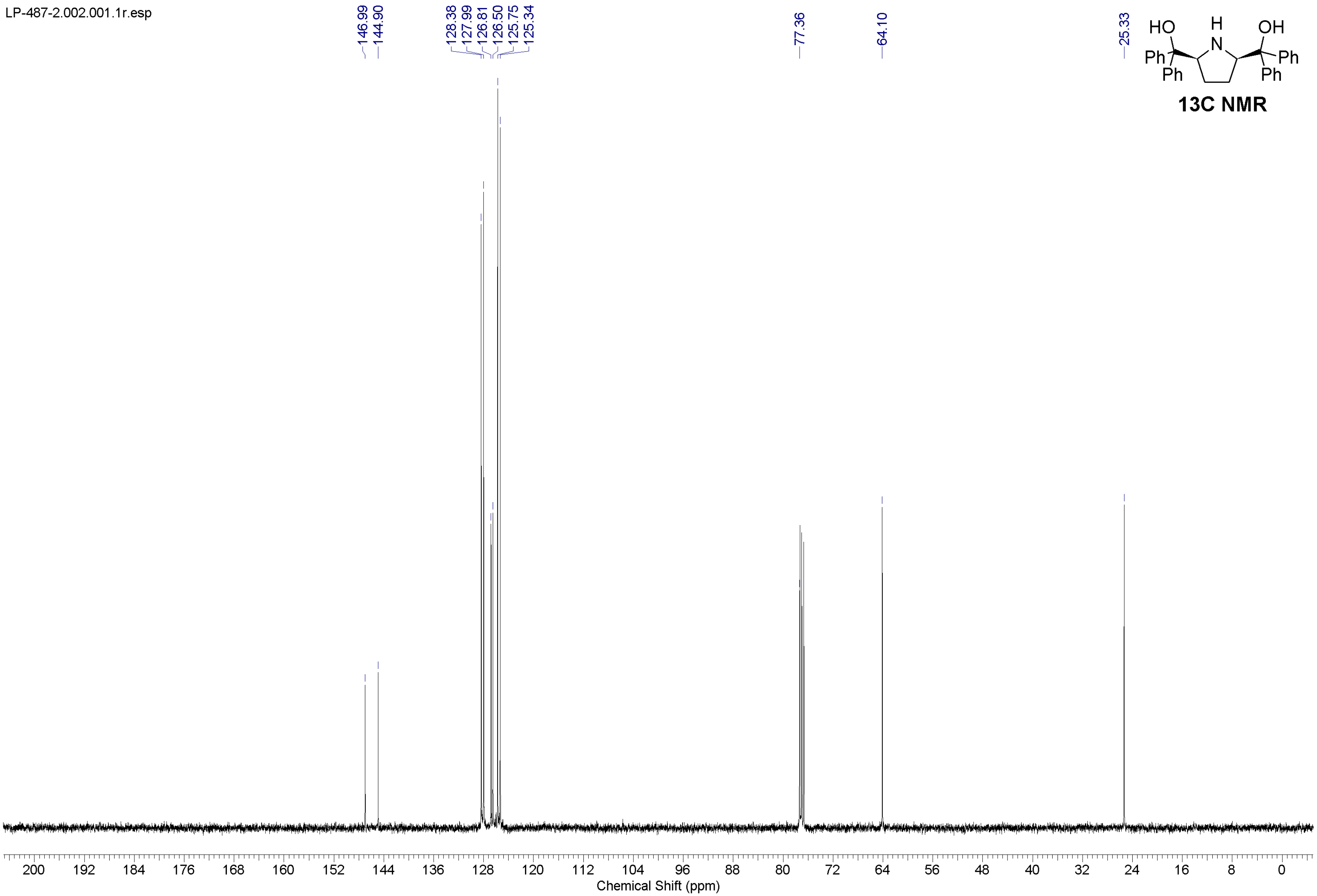

LP-487-2.002.001.1r.esp
146.99
144.90
128.38
127.99
126.81
126.50
125.75
125.34
77.36
64.10
25.33
HO
H
N
OH
Ph
Ph
Ph
Ph
13C NMR
200
192
184
176
168
160
152
144
136
128
120
112
104
96
88
80
72
64
56
48
40
32
24
16
8
0
Chemical Shift (ppm)

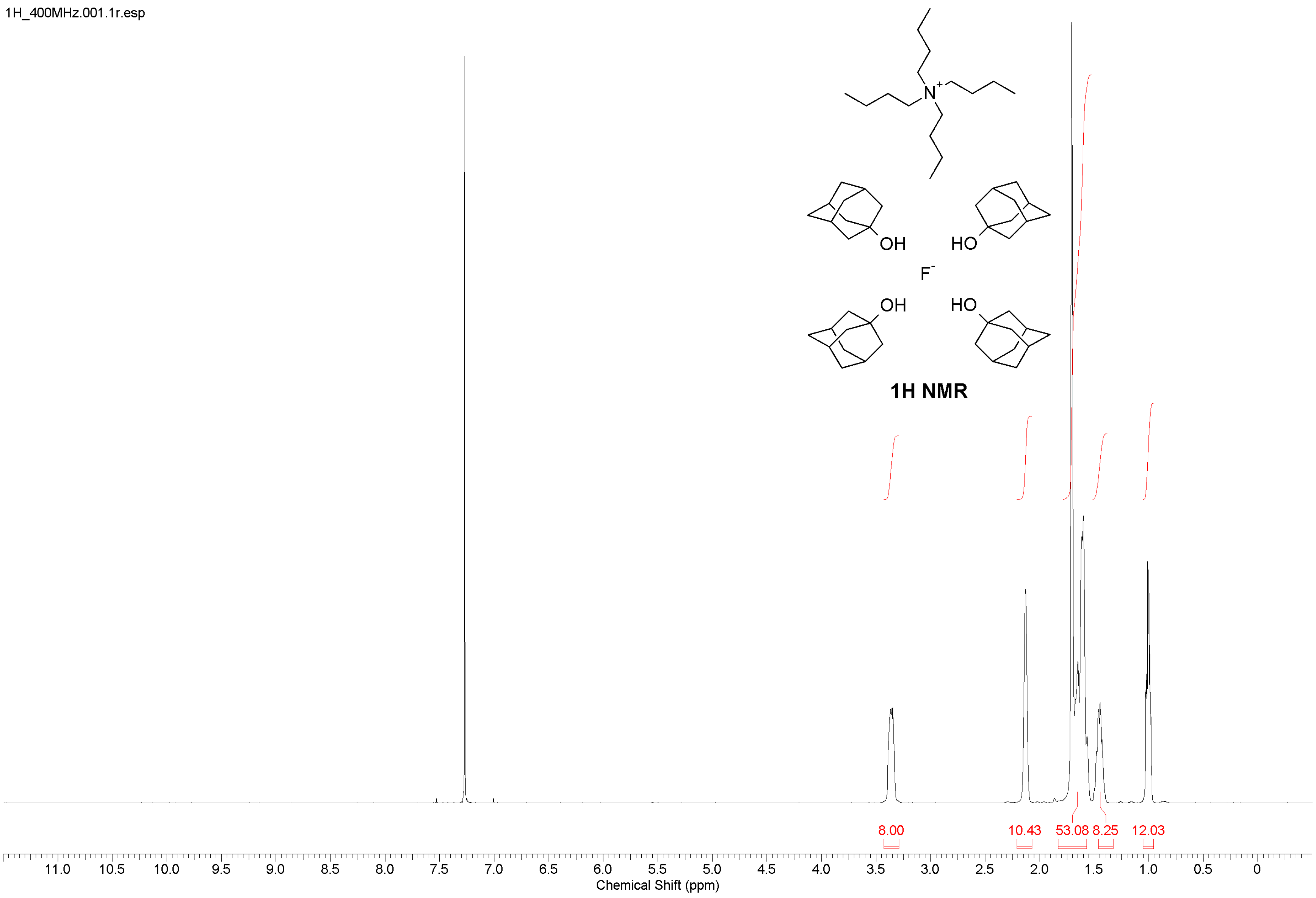

1H_400MHz.001.1r.esp
OH
HO
F-
OH
HO
1H NMR
8.00
10.43
53.08
8.25
12.03
11.0 10.5 10.0 9.5 9.0 8.5 8.0 7.5 7.0 6.5 6.0 5.5 5.0 4.5 4.0 3.5 3.0 2.5 2.0 1.5 1.0 0.5 0
Chemical Shift (ppm)

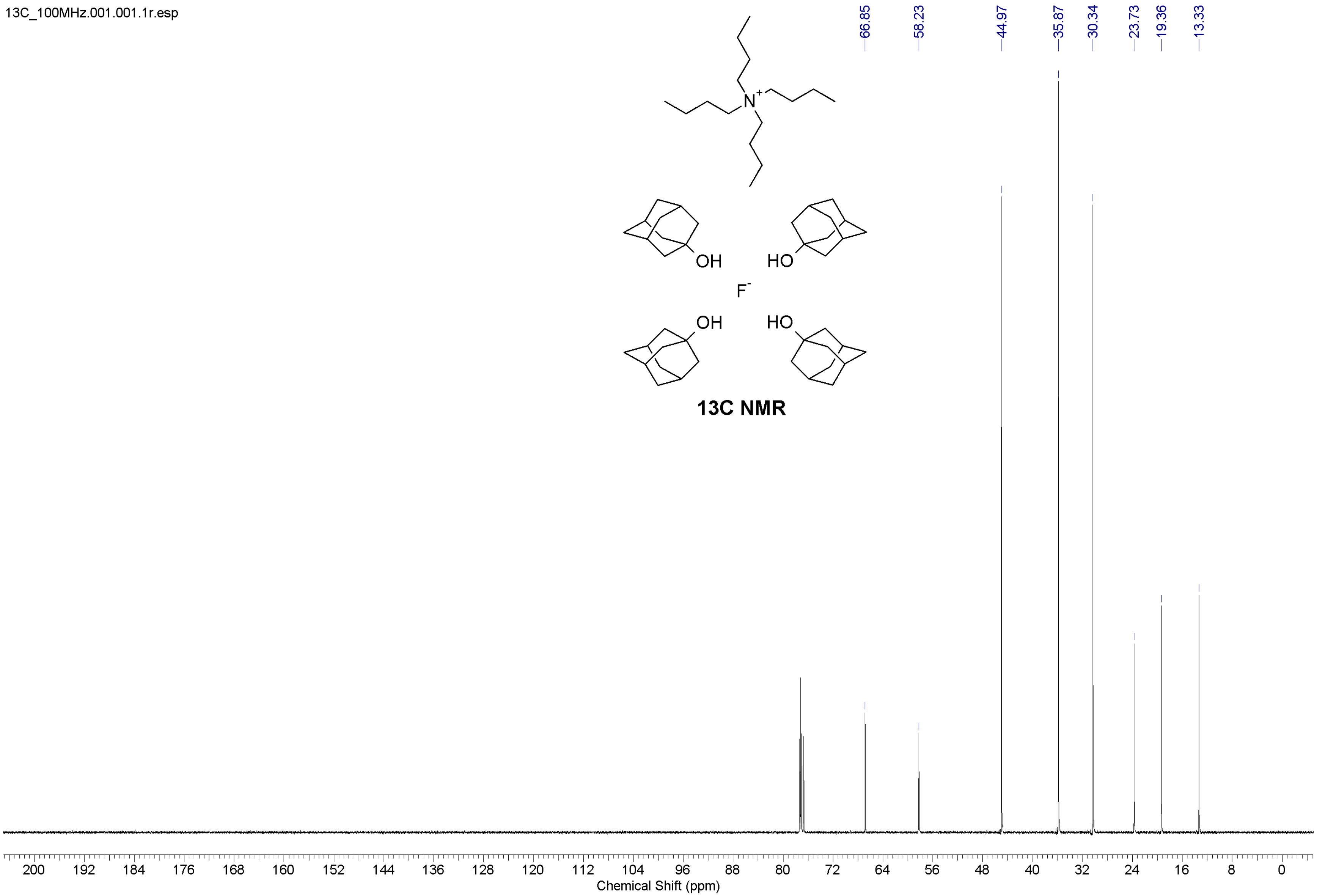
13C_100MHz.001.001.1r.esp
66.85
58.23
44.97
35.87
30.34
23.73
19.36
13.33
N+
OH
HO
F-
OH
HO
13C NMR
200 192 184 176 168 160 152 144 136 128 120 112 104 96 88 80 72 64 56 48 40 32 24 16 8 0
Chemical Shift (ppm)

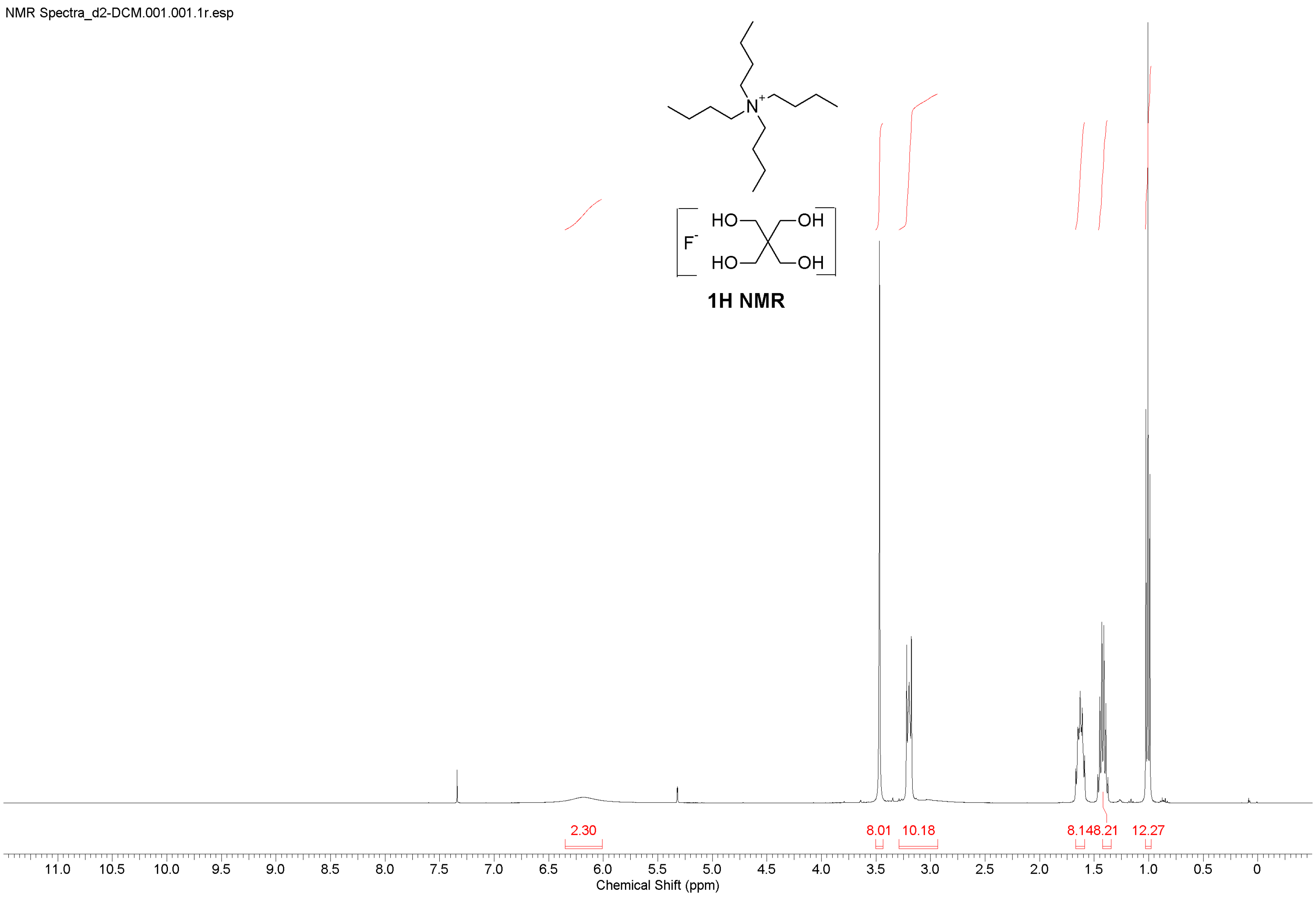
NMR Spectra_d2-DCM.001.001.1r.esp
N+
HO
OH
F-
HO
OH
1H NMR
2.30
8.01
10.18
8.14
8.21
12.27
11.0
10.5
10.0
9.5
9.0
8.5
8.0
7.5
7.0
6.5
6.0
5.5
5.0
4.5
4.0
3.5
3.0
2.5
2.0
1.5
1.0
0.5
0
Chemical Shift (ppm)

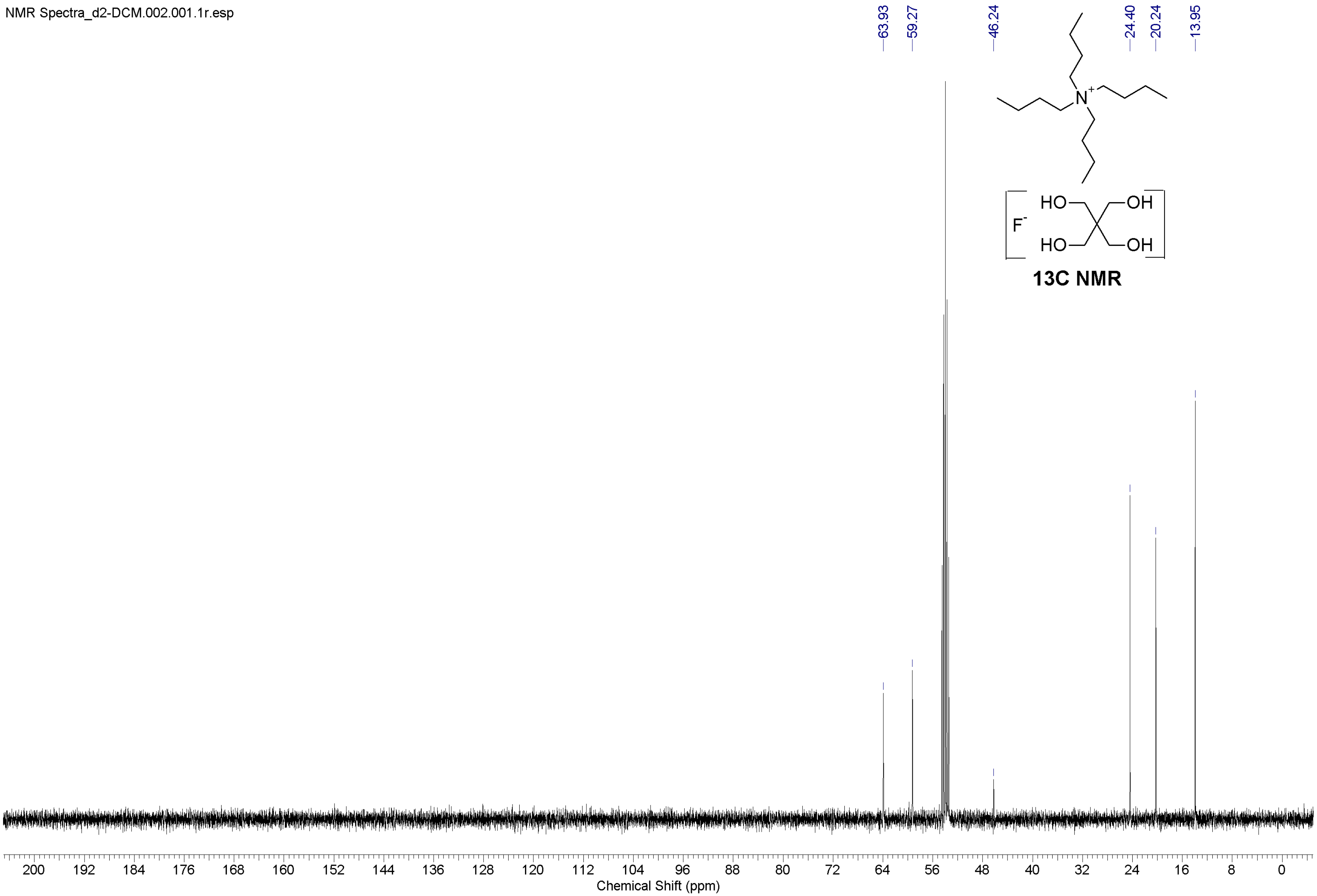
NMR Spectra_d2-DCM.002.001.1r.esp
63.93
59.27
46.24
24.40
20.24
13.95
N+
F-
HO
OH
HO
OH
13C NMR
200 192 184 176 168 160 152 144 136 128 120 112 104 96 88 80 72 64 56 48 40 32 24 16 8 0
Chemical Shift (ppm)

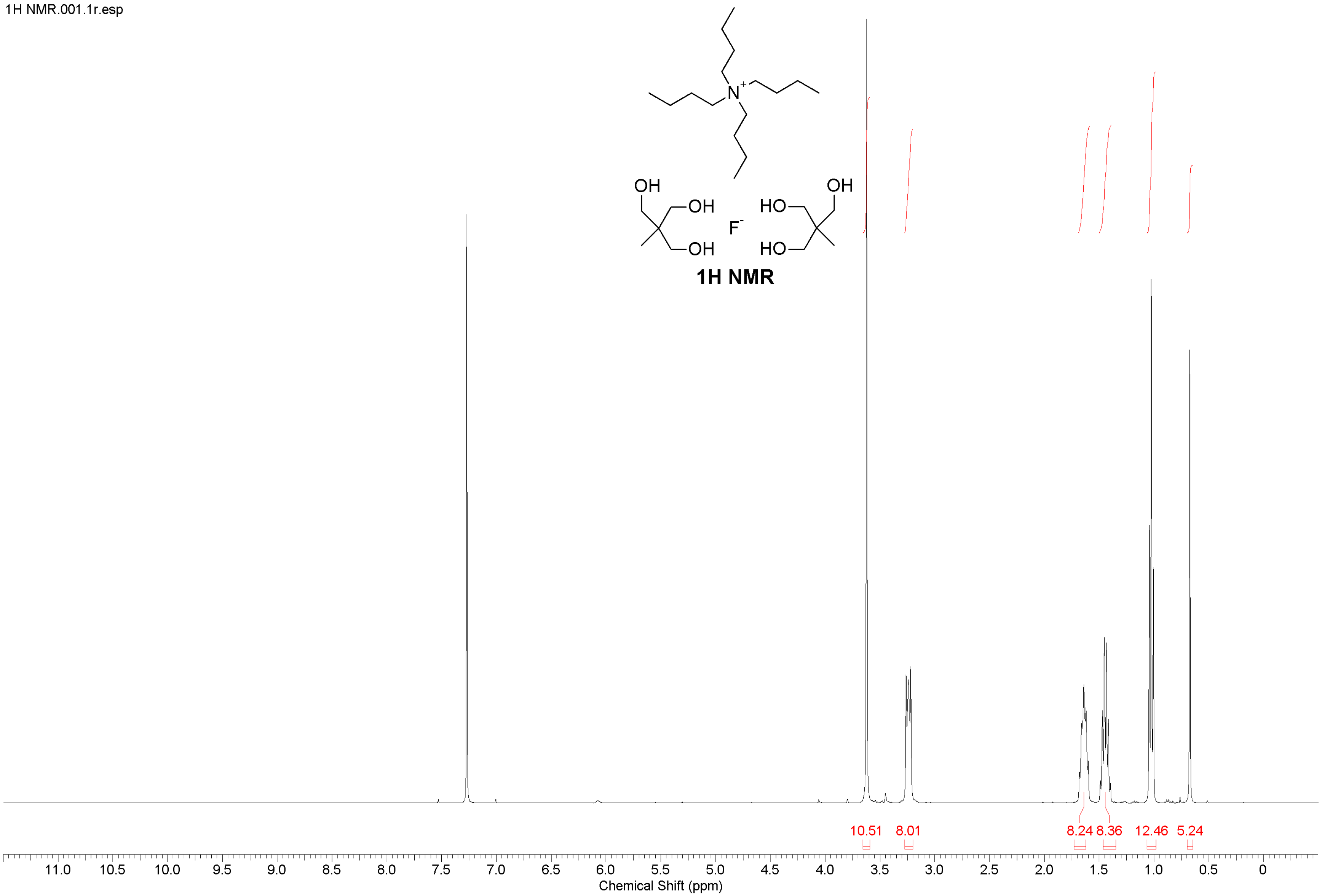

1H NMR.001.1r.esp
N+
OH
OH
OH
F-
HO
HO
OH
1H NMR
10.51
8.01
8.24
8.36
12.46
5.24
11.0 10.5 10.0 9.5 9.0 8.5 8.0 7.5 7.0 6.5 6.0 5.5 5.0 4.5 4.0 3.5 3.0 2.5 2.0 1.5 1.0 0.5 0
Chemical Shift (ppm)

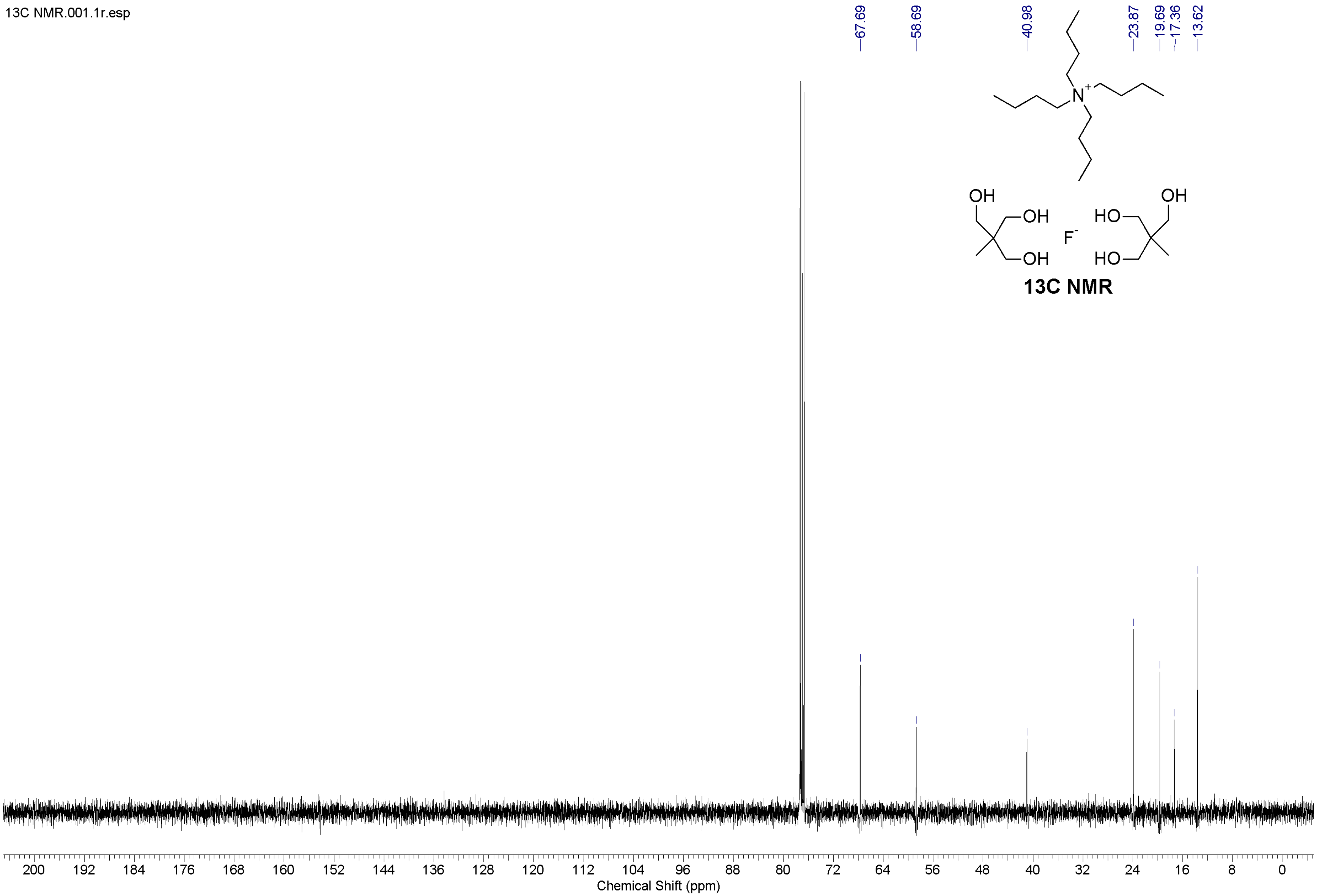
13C NMR.001.1r.esp
67.69
58.69
40.98
23.87
19.69
17.36
13.62
N+
OH
OH
OH
F-
OH
HO
HO
13C NMR
200 192 184 176 168 160 152 144 136 128 120 112 104 96 88 80 72 64 56 48 40 32 24 16 8 0
Chemical Shift (ppm)

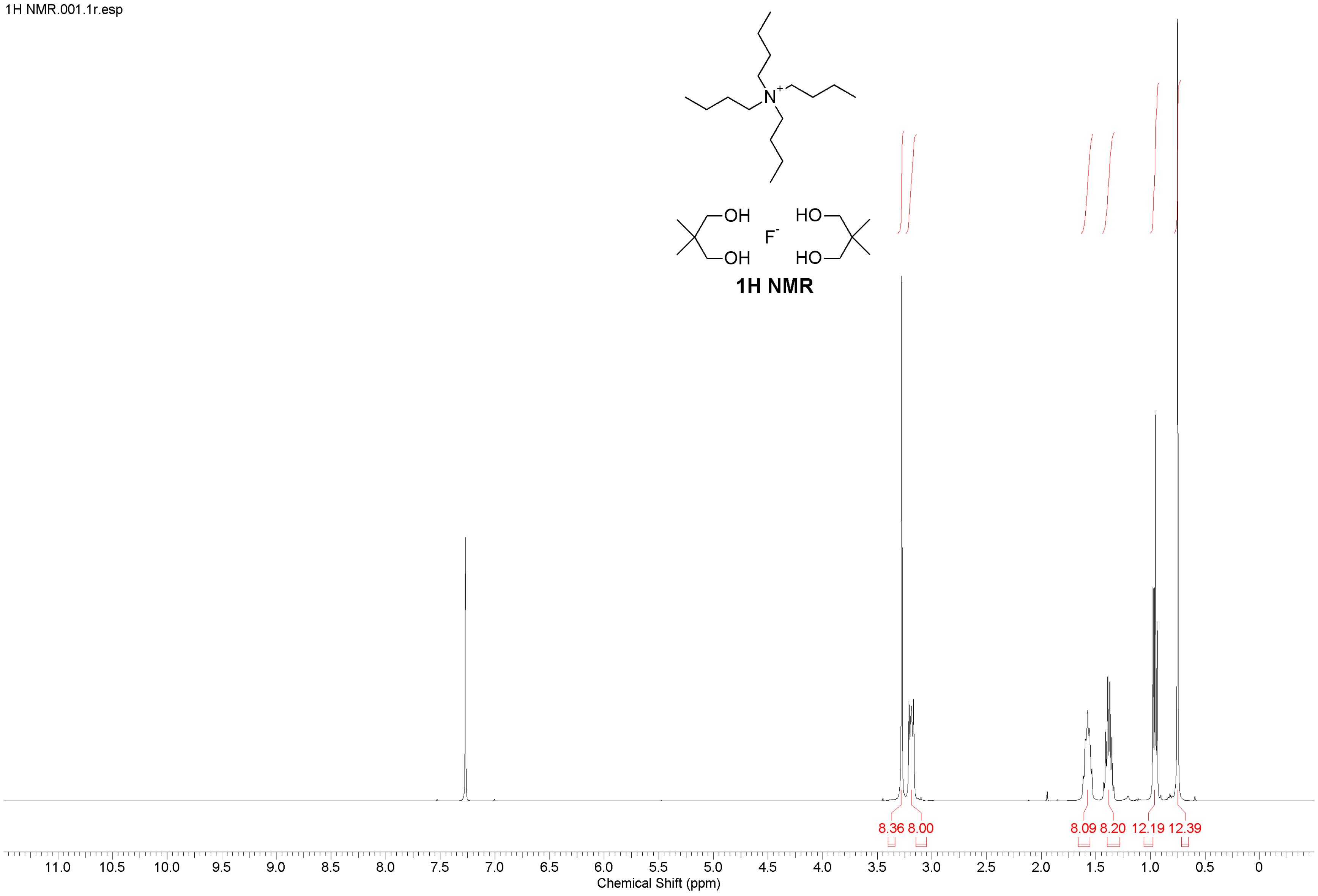
1H NMR.001.1r.esp
N+
OH
OH
F-
HO
HO
1H NMR
8.36
8.00
8.09
8.20
12.19
12.39
11.0 10.5 10.0 9.5 9.0 8.5 8.0 7.5 7.0 6.5 6.0 5.5 5.0 4.5 4.0 3.5 3.0 2.5 2.0 1.5 1.0 0.5 0
Chemical Shift (ppm)

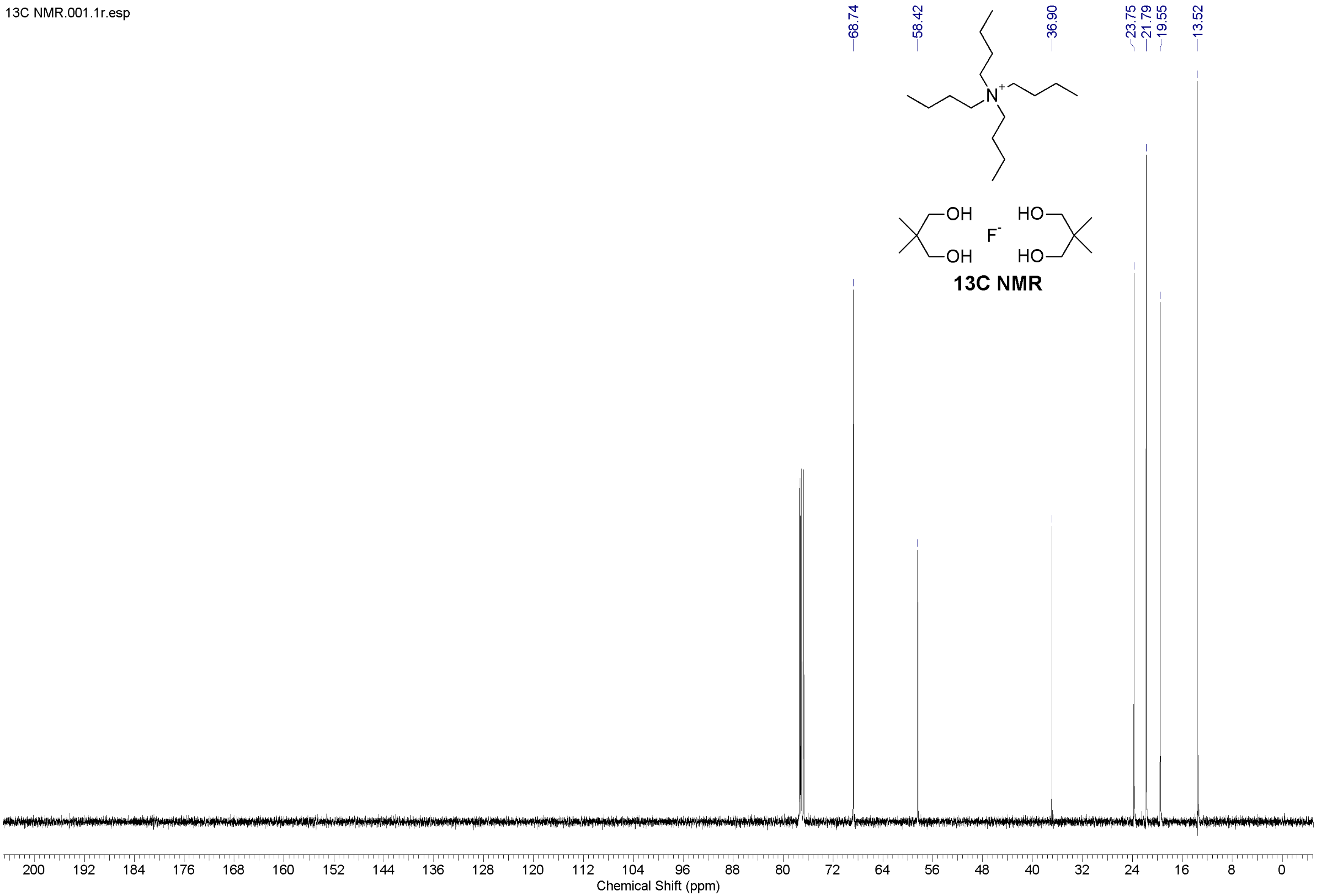
13C NMR.001.1r.esp
68.74
58.42
36.90
23.75
21.79
19.55
13.52
N+
OH
OH
HO
HO
F-
13C NMR
200 192 184 176 168 160 152 144 136 128 120 112 104 96 88 80 72 64 56 48 40 32 24 16 8 0
Chemical Shift (ppm)

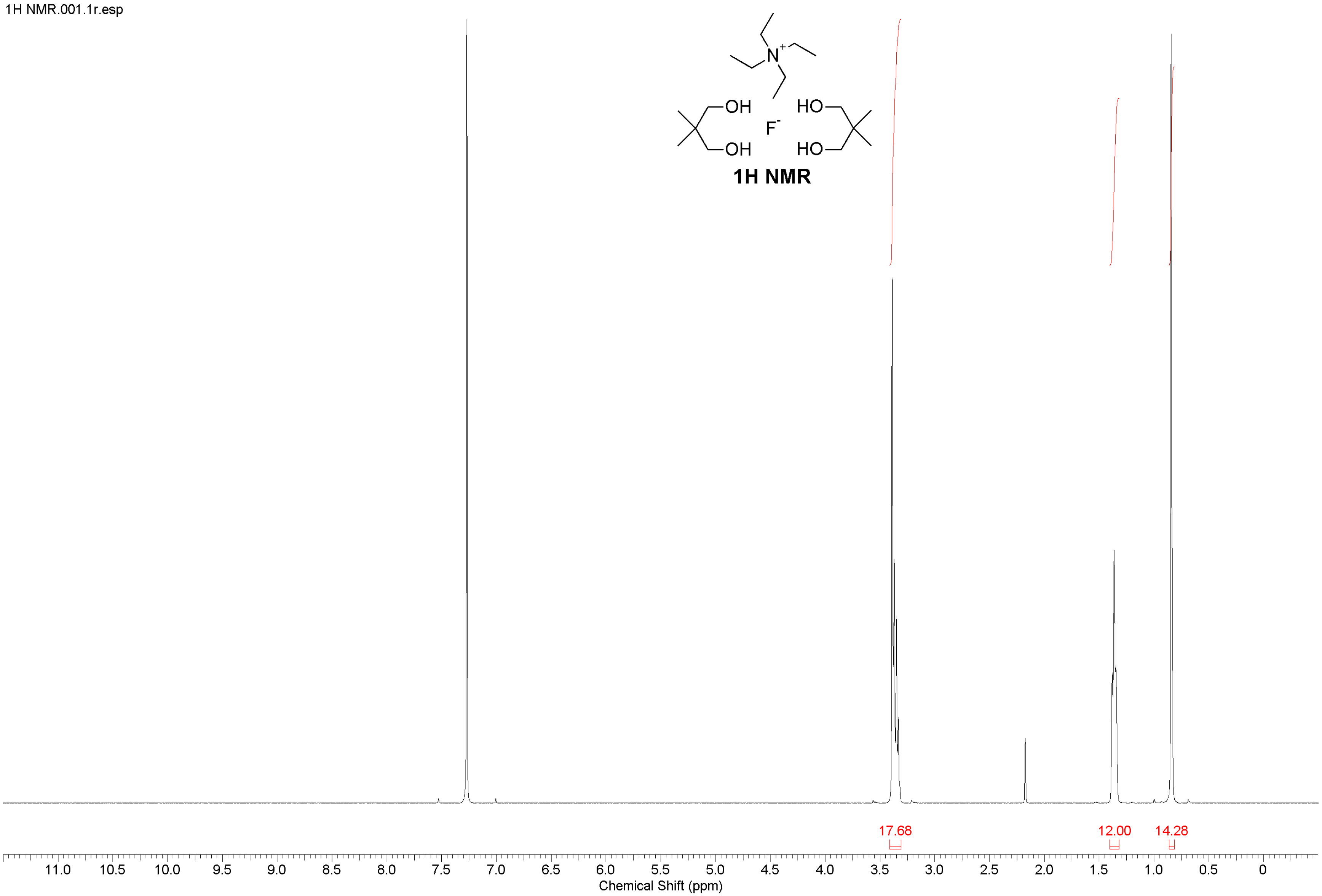
1H NMR.001.1r.esp
OH
OH
HO
HO
N+
F-
1H NMR
17.68
12.00
14.28
11.0
10.5
10.0
9.5
9.0
8.5
8.0
7.5
7.0
6.5
6.0
5.5
5.0
4.5
4.0
3.5
3.0
2.5
2.0
1.5
1.0
0.5
0
Chemical Shift (ppm)

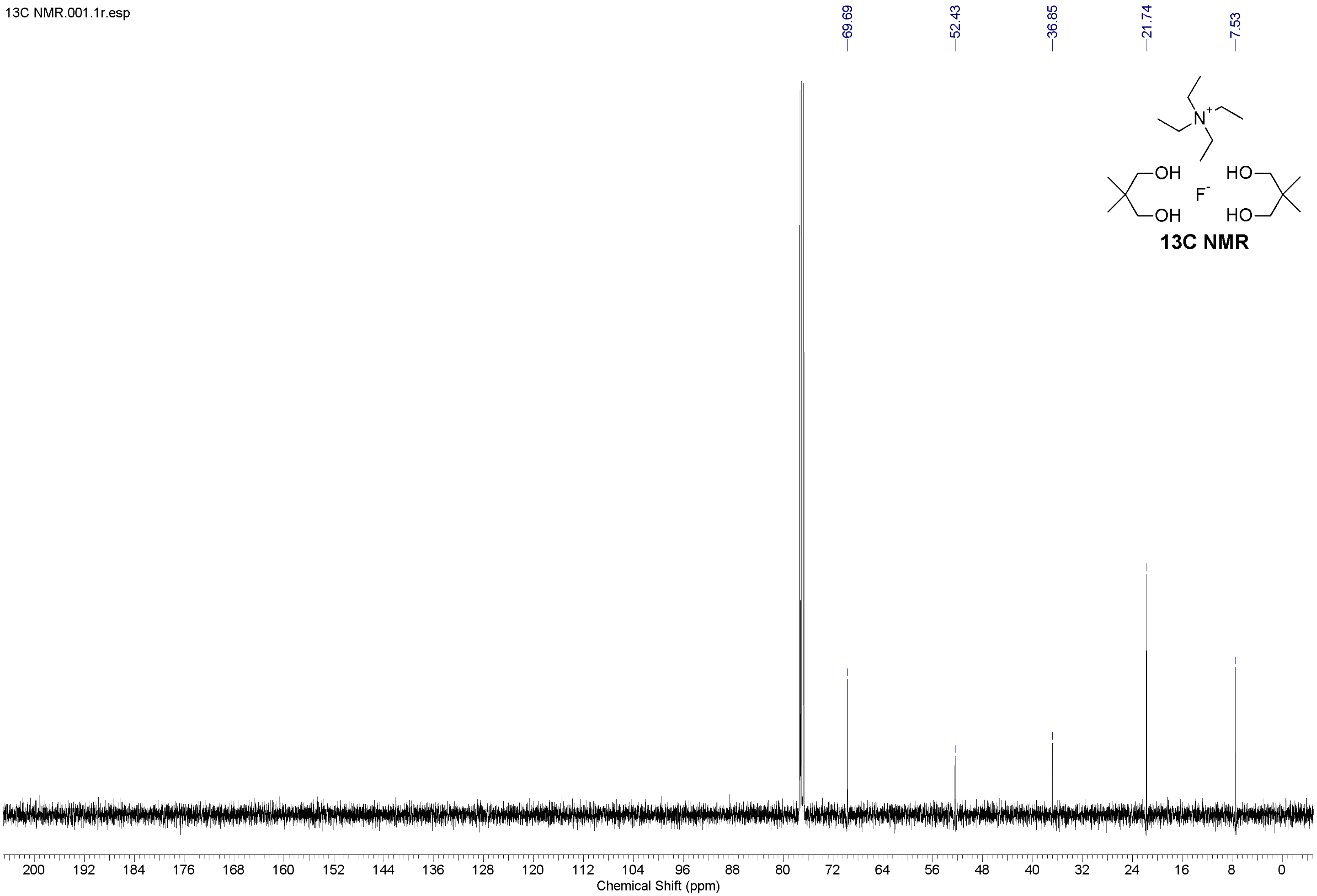
13C NMR.001.1r.esp
69.69
52.43
36.85
21.74
7.53
OH
OH
HO
HO
F
13C NMR
200
192
184
176
168
160
152
144
136
128
120
112
104
96
88
80
72
64
56
48
40
32
24
16
8
0
Chemical Shift (ppm)

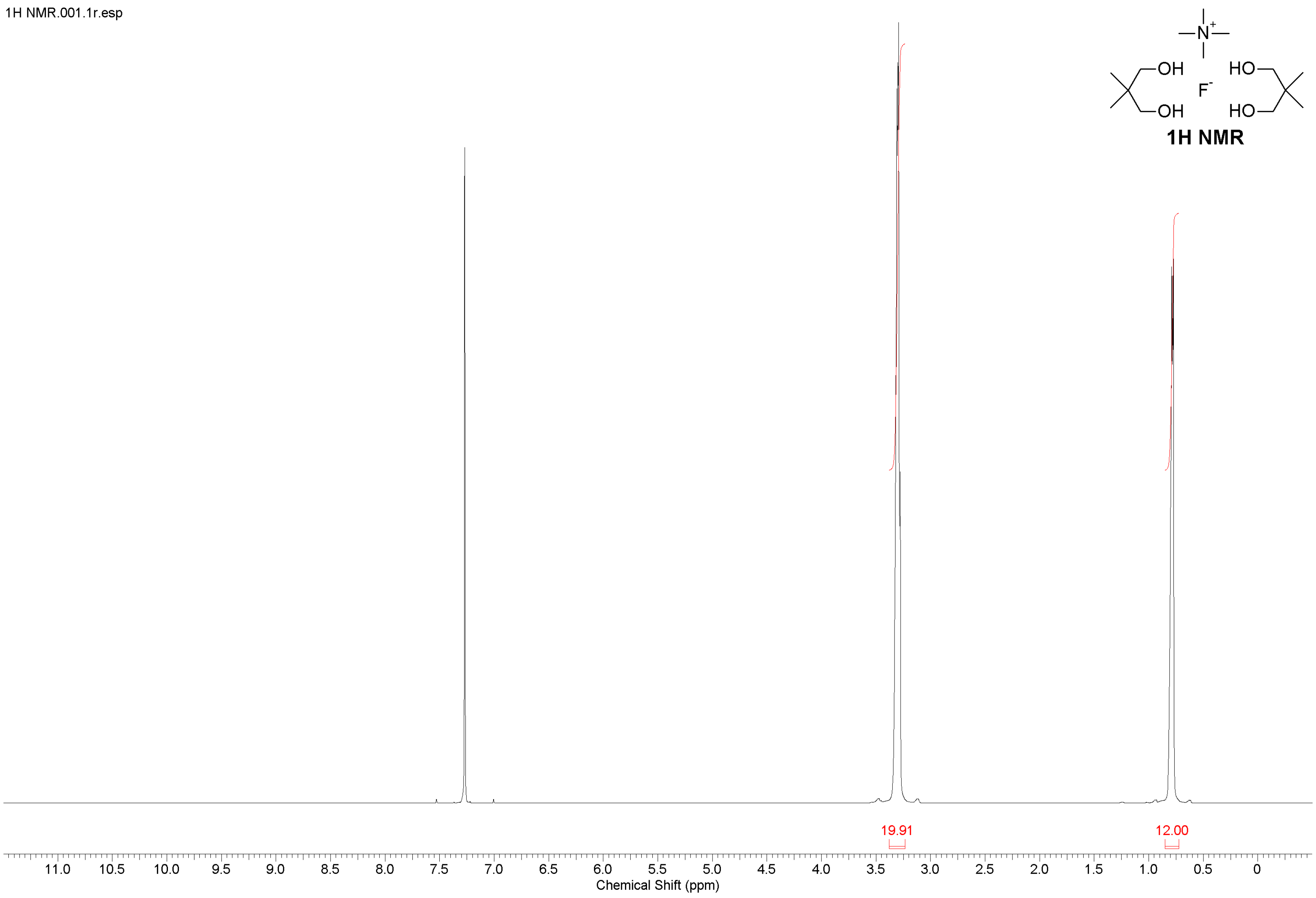
1H NMR.001.1r.esp
OH
OH
HO
HO
N+
F-
1H NMR
19.91
12.00
11.0 10.5 10.0 9.5 9.0 8.5 8.0 7.5 7.0 6.5 6.0 5.5 5.0 4.5 4.0 3.5 3.0 2.5 2.0 1.5 1.0 0.5 0
Chemical Shift (ppm)

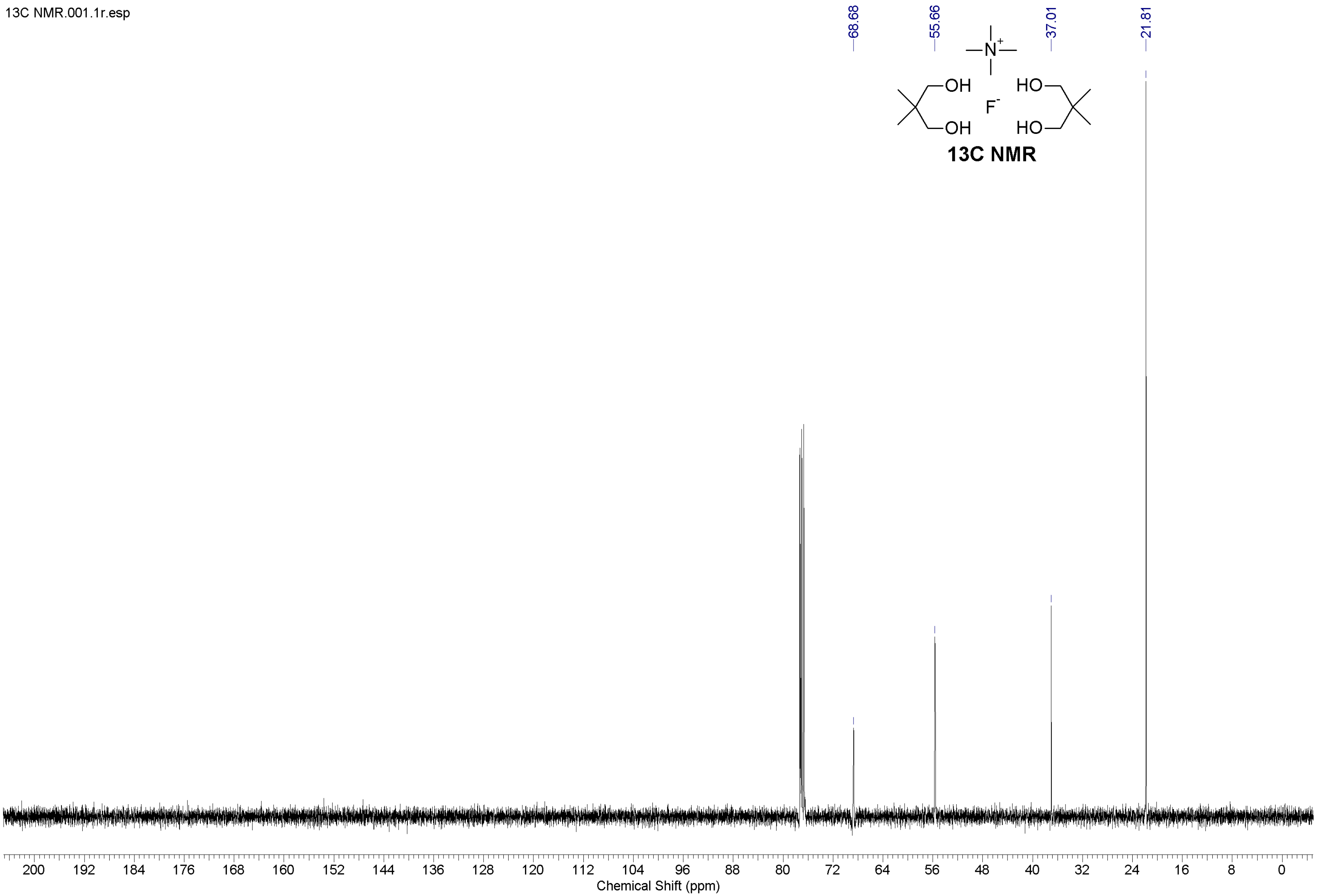

13C NMR.001.1r.esp
68.68
55.66
37.01
21.81
N+
OH
OH
HO
HO
F-
13C NMR
Chemical Shift (ppm)

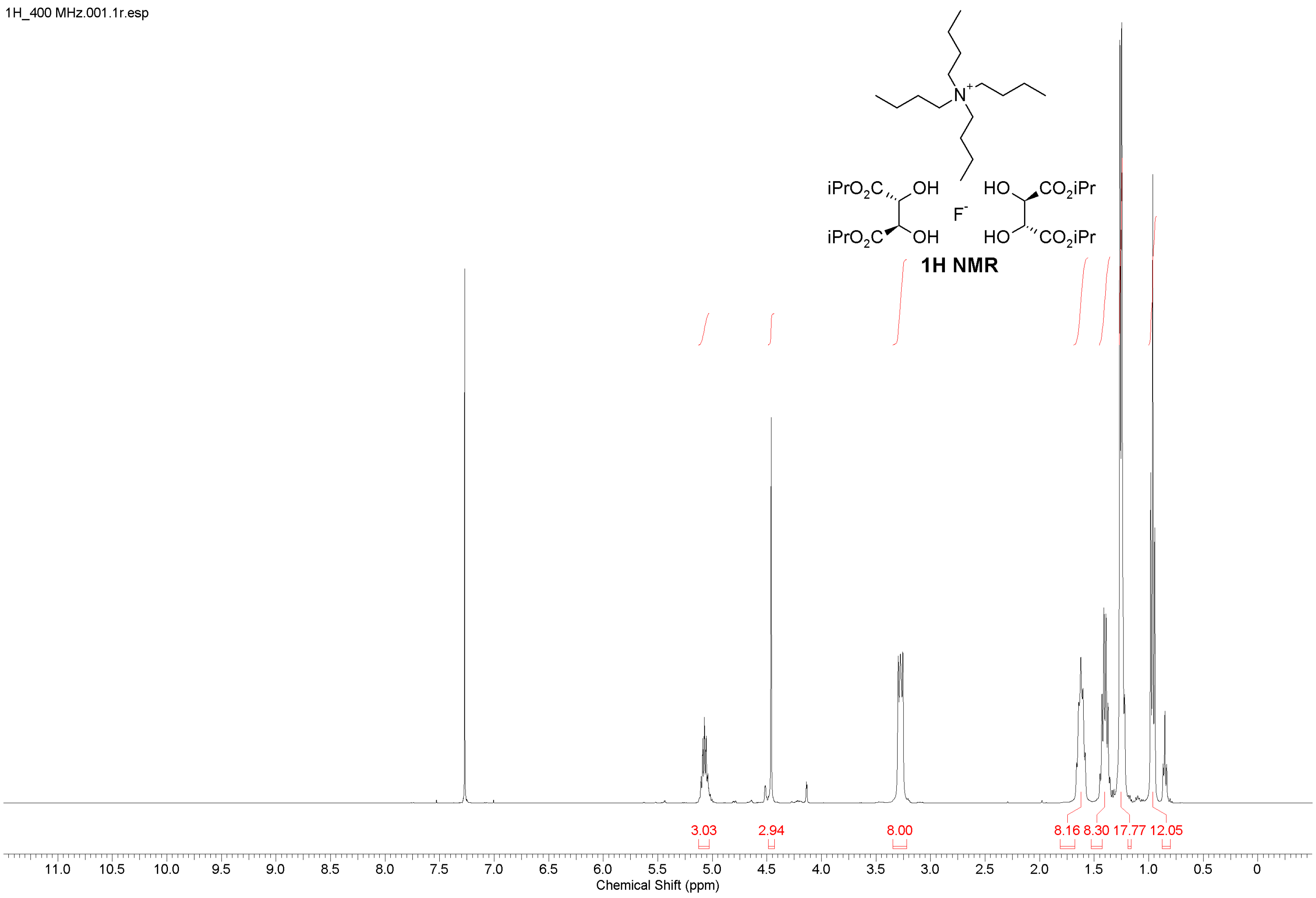

1H_400 MHz.001.1r.esp
iPrO2C
OH
HO
CO2iPr
F-
1H NMR
3.03
2.94
8.00
8.16
8.30
17.77
12.05
11.0
10.5
10.0
9.5
9.0
8.5
8.0
7.5
7.0
6.5
6.0
5.5
5.0
4.5
4.0
3.5
3.0
2.5
2.0
1.5
1.0
0.5
0
Chemical Shift (ppm)

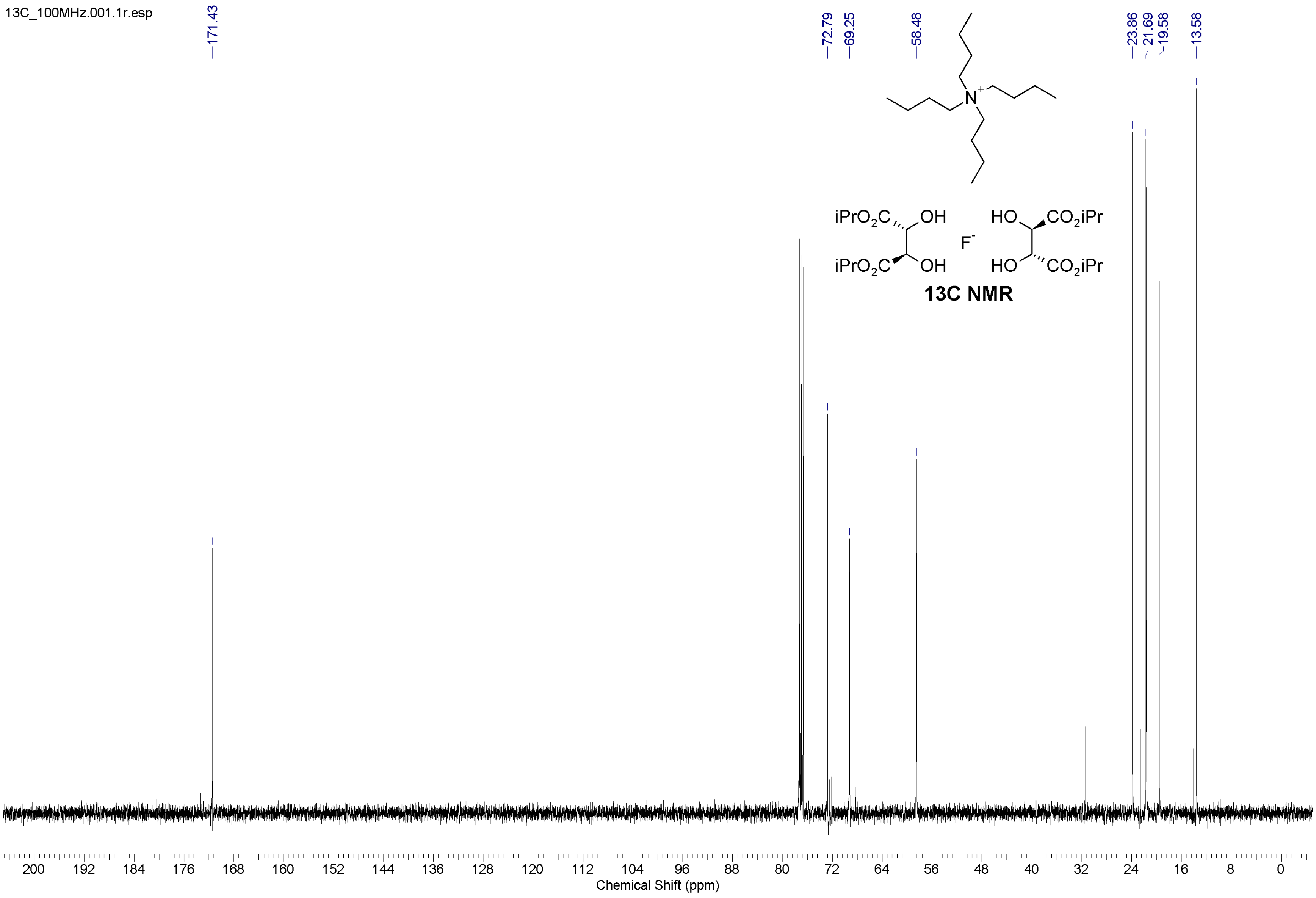
13C_100MHz.001.1r.esp
171.43
72.79
69.25
58.48
23.86
21.69
19.58
13.58
iPrO2C
OH
F-
HO
CO2iPr
13C NMR
200 192 184 176 168 160 152 144 136 128 120 112 104 96 88 80 72 64 56 48 40 32 24 16 8 0
Chemical Shift (ppm)

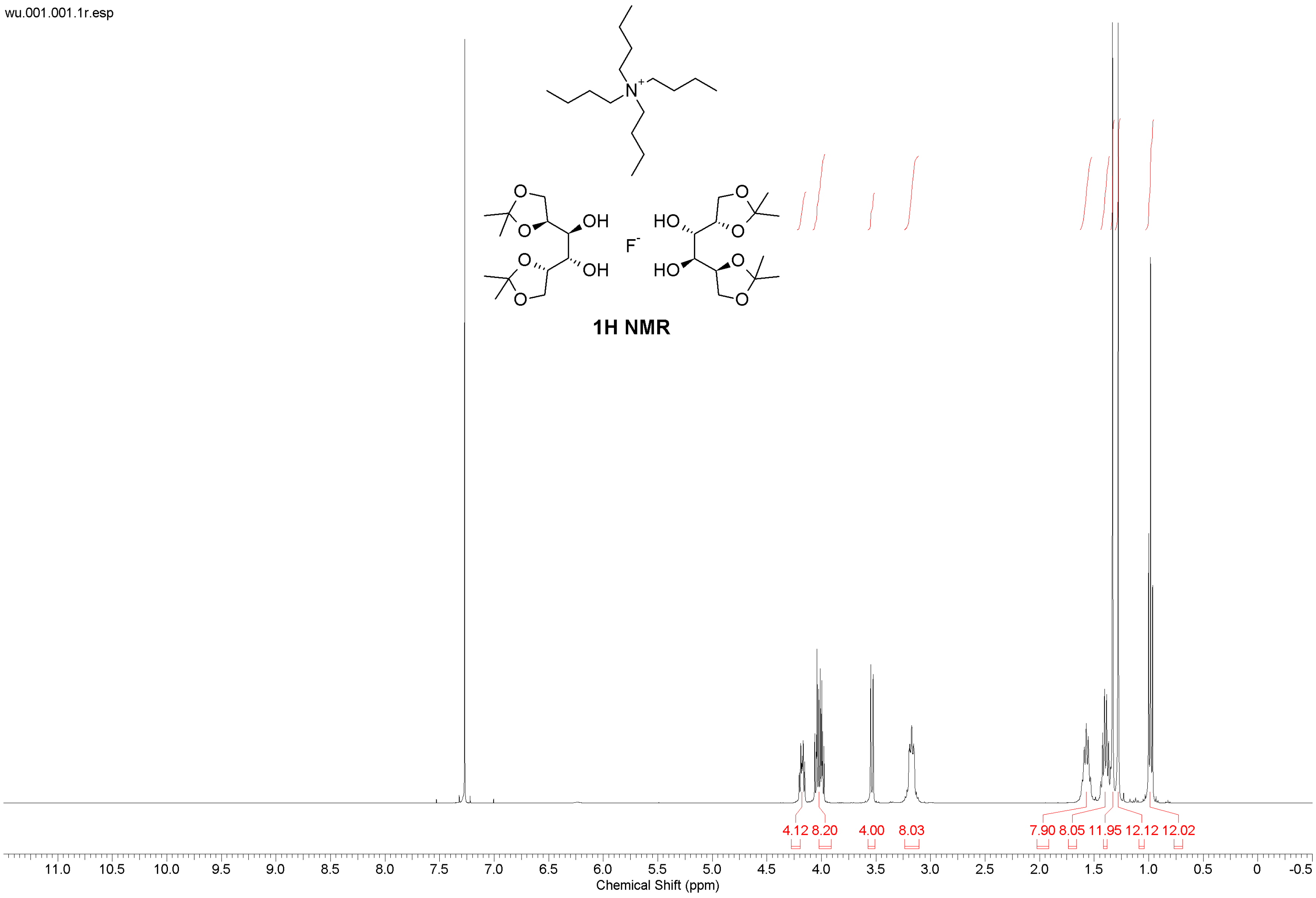
wu.001.001.1r.esp
1H NMR
4.12
8.20
4.00
8.03
7.90
8.05
11.95
12.12
12.02
Chemical Shift (ppm)

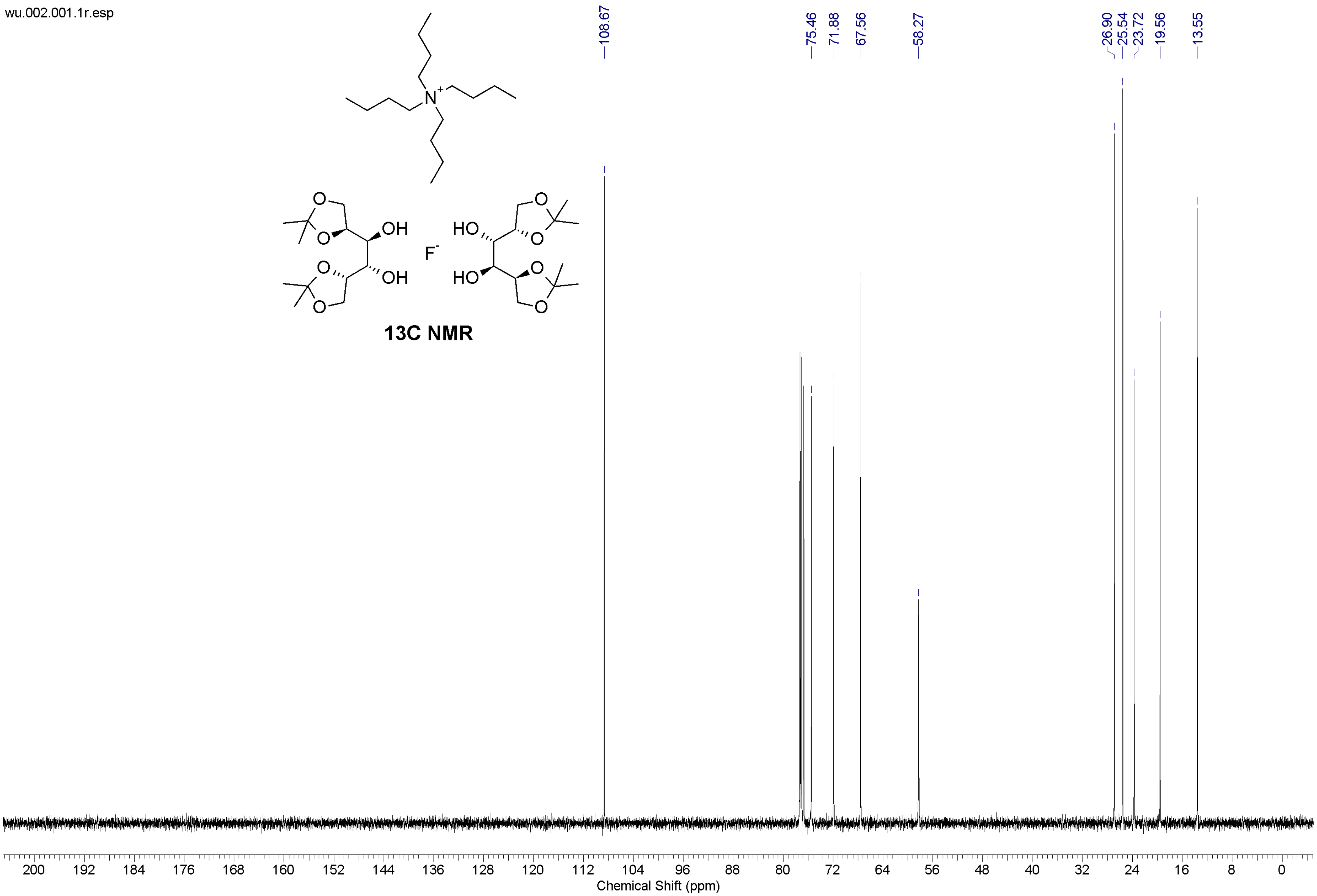
wu.002.001.1r.esp
13C NMR
108.67
75.46
71.88
67.56
58.27
26.90
25.54
23.72
19.56
13.55
Chemical Shift (ppm)

1H NMR.001.1r.esp

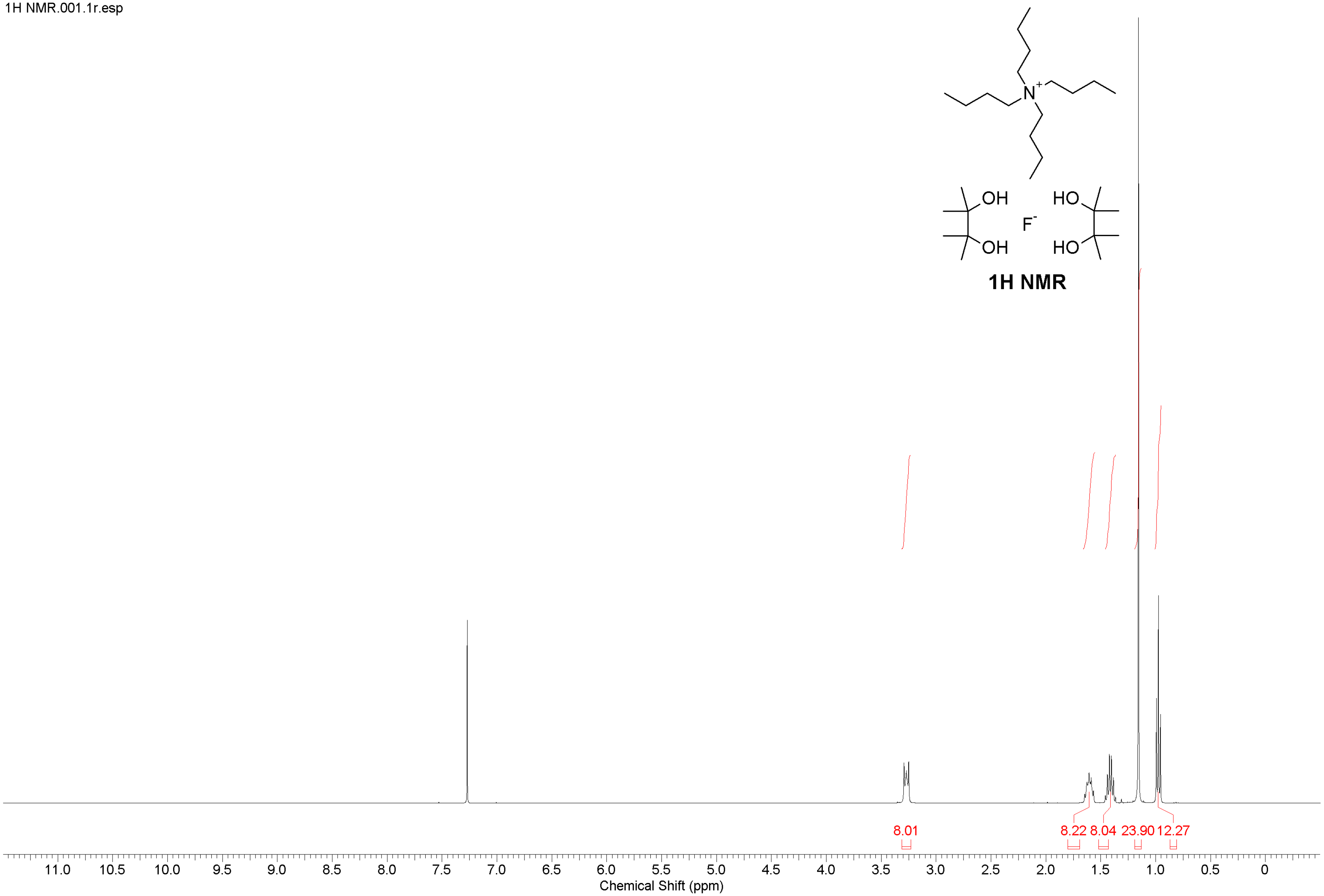

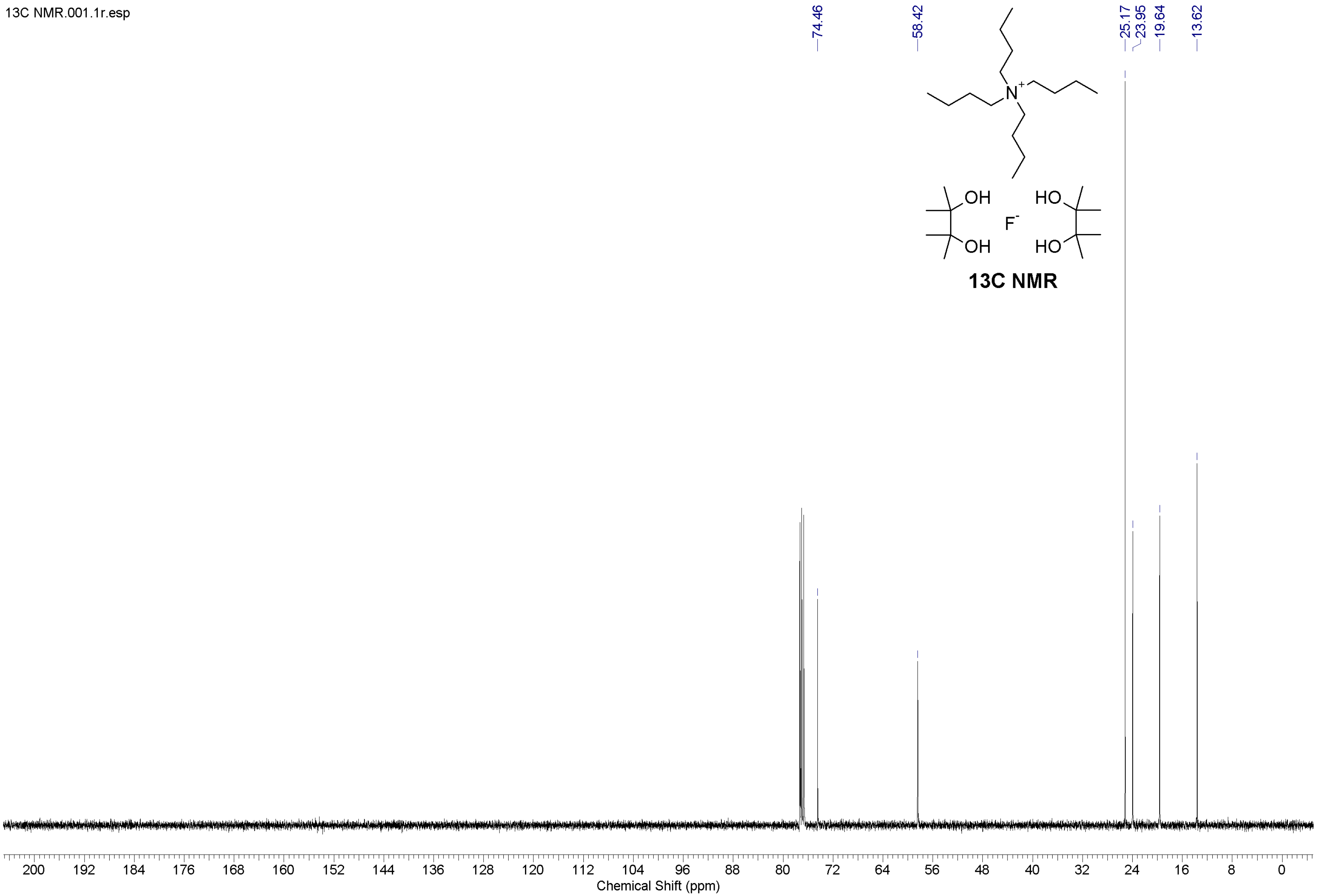
13C NMR.001.1r.esp
74.46
58.42
25.17
23.95
19.64
13.62
N+
OH
OH
HO
HO
F-
13C NMR
200
192
184
176
168
160
152
144
136
128
120
112
104
96
88
80
72
64
56
48
40
32
24
16
8
0
Chemical Shift (ppm)

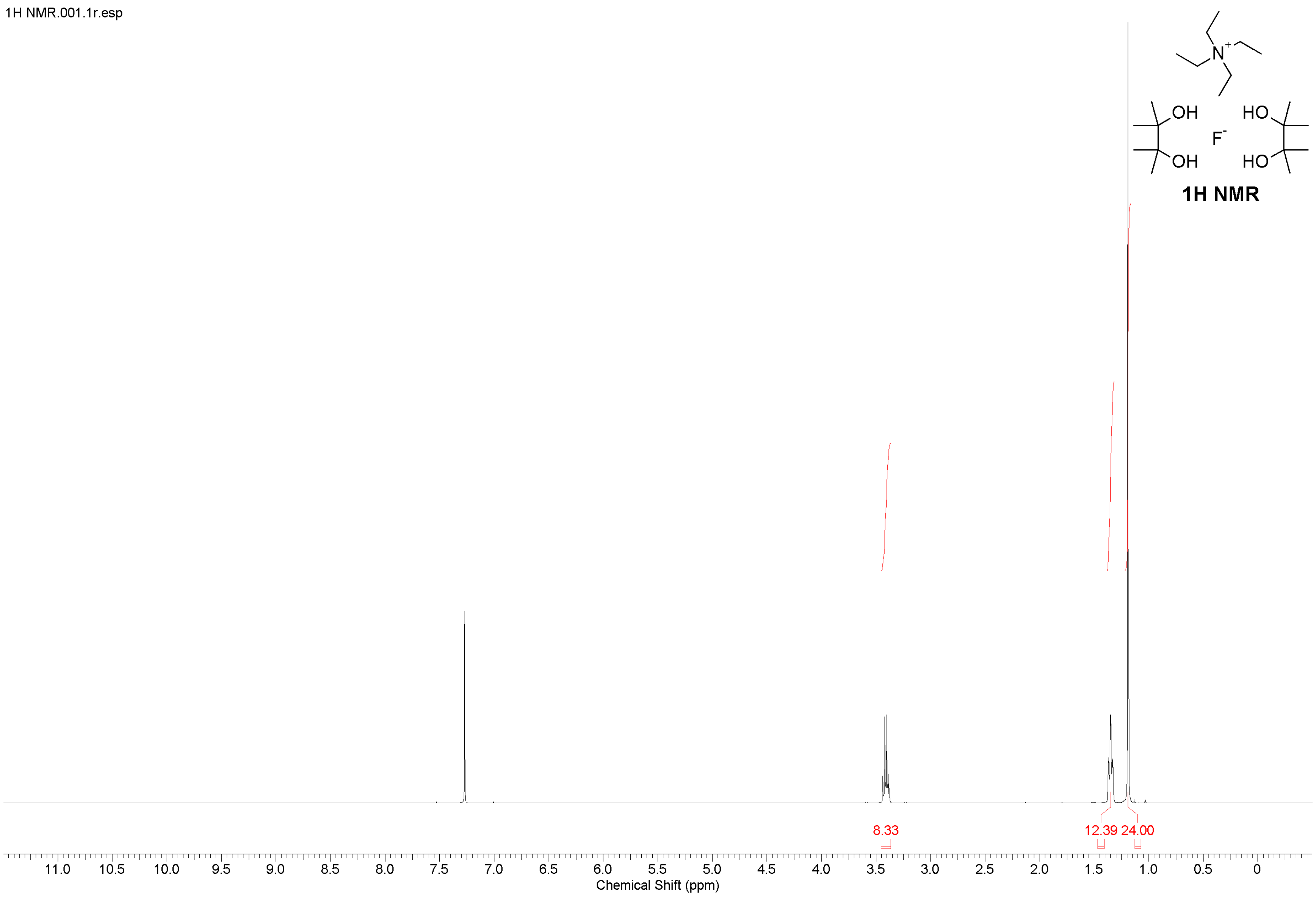
1H NMR.001.1r.esp
OH
OH
HO
HO
N+
F-
1H NMR
8.33
12.39
24.00
11.0 10.5 10.0 9.5 9.0 8.5 8.0 7.5 7.0 6.5 6.0 5.5 5.0 4.5 4.0 3.5 3.0 2.5 2.0 1.5 1.0 0.5 0
Chemical Shift (ppm)

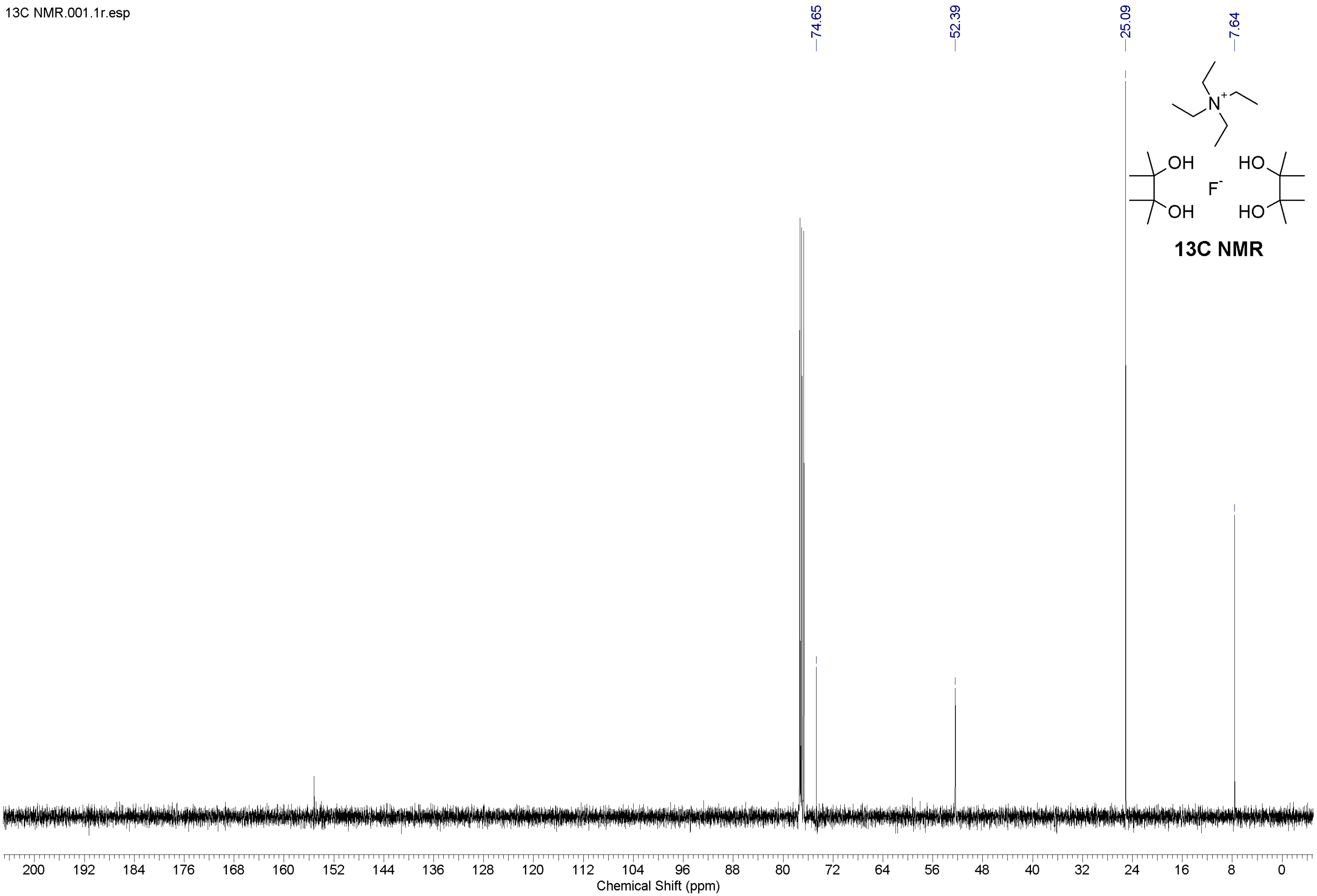
13C NMR.001.1r.esp
74.65
52.39
25.09
7.64
N+
OH
OH
HO
HO
F-
13C NMR
200
192
184
176
168
160
152
144
136
128
120
112
104
96
88
80
72
64
56
48
40
32
24
16
8
0
Chemical Shift (ppm)

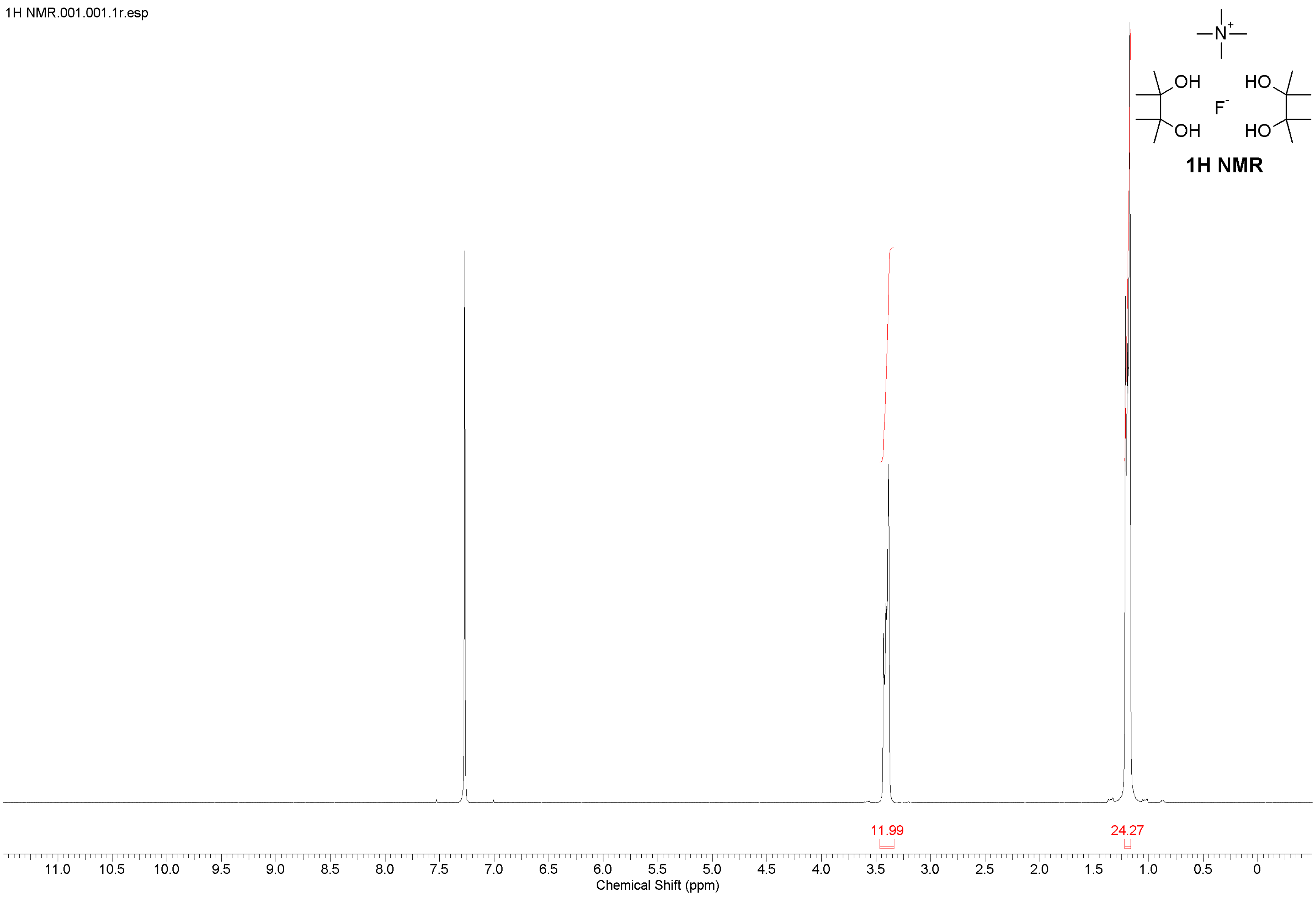
1H NMR.001.001.1r.esp
OH
OH
HO
HO
F-
N+
1H NMR
11.99
24.27
11.0
10.5
10.0
9.5
9.0
8.5
8.0
7.5
7.0
6.5
6.0
5.5
5.0
4.5
4.0
3.5
3.0
2.5
2.0
1.5
1.0
0.5
0
Chemical Shift (ppm)

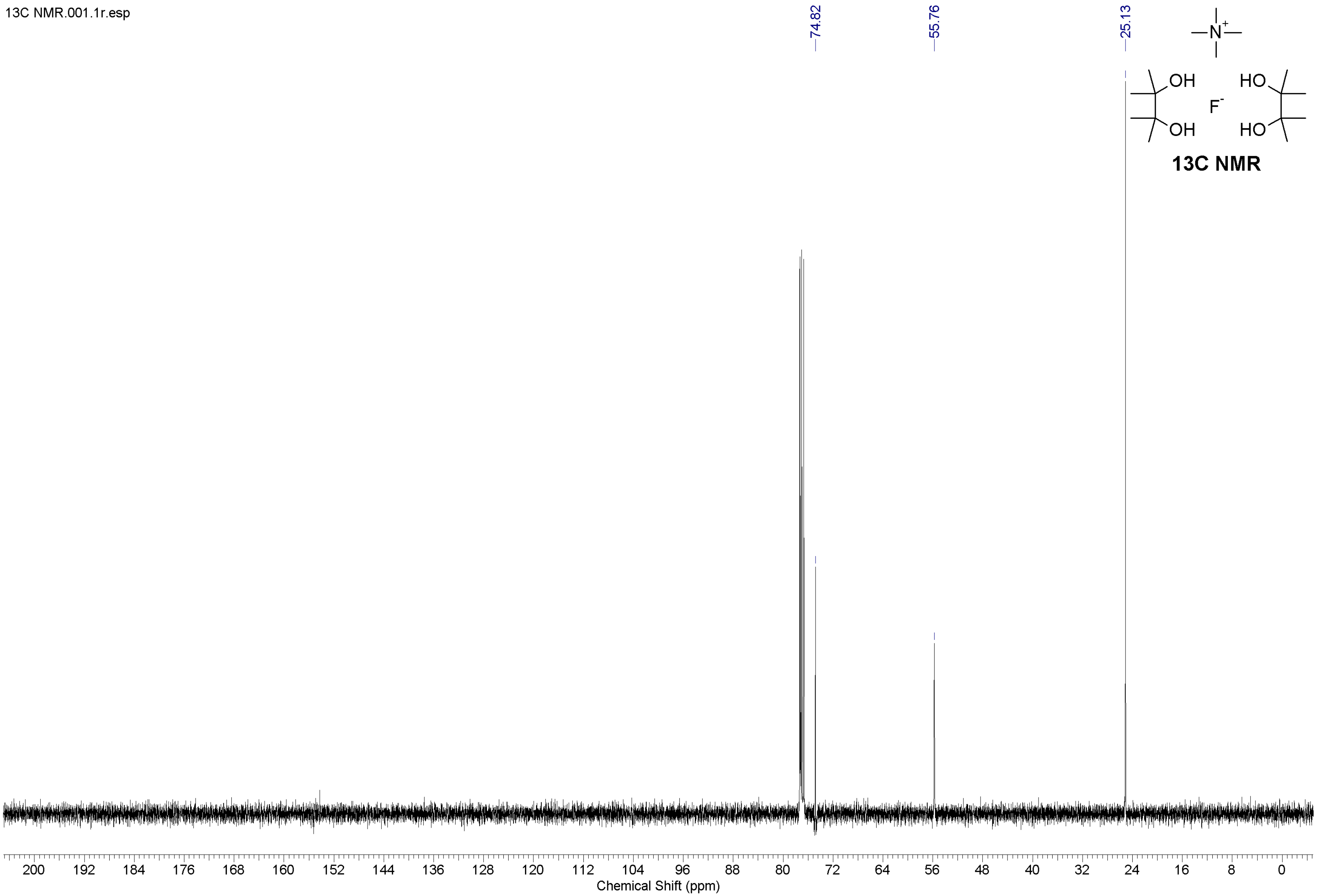

13C NMR.001.1r.esp
74.82
55.76
25.13
N+
OH
OH
HO
HO
F-
13C NMR
200
192
184
176
168
160
152
144
136
128
120
112
104
96
88
80
72
64
56
48
40
32
24
16
8
0
Chemical Shift (ppm)

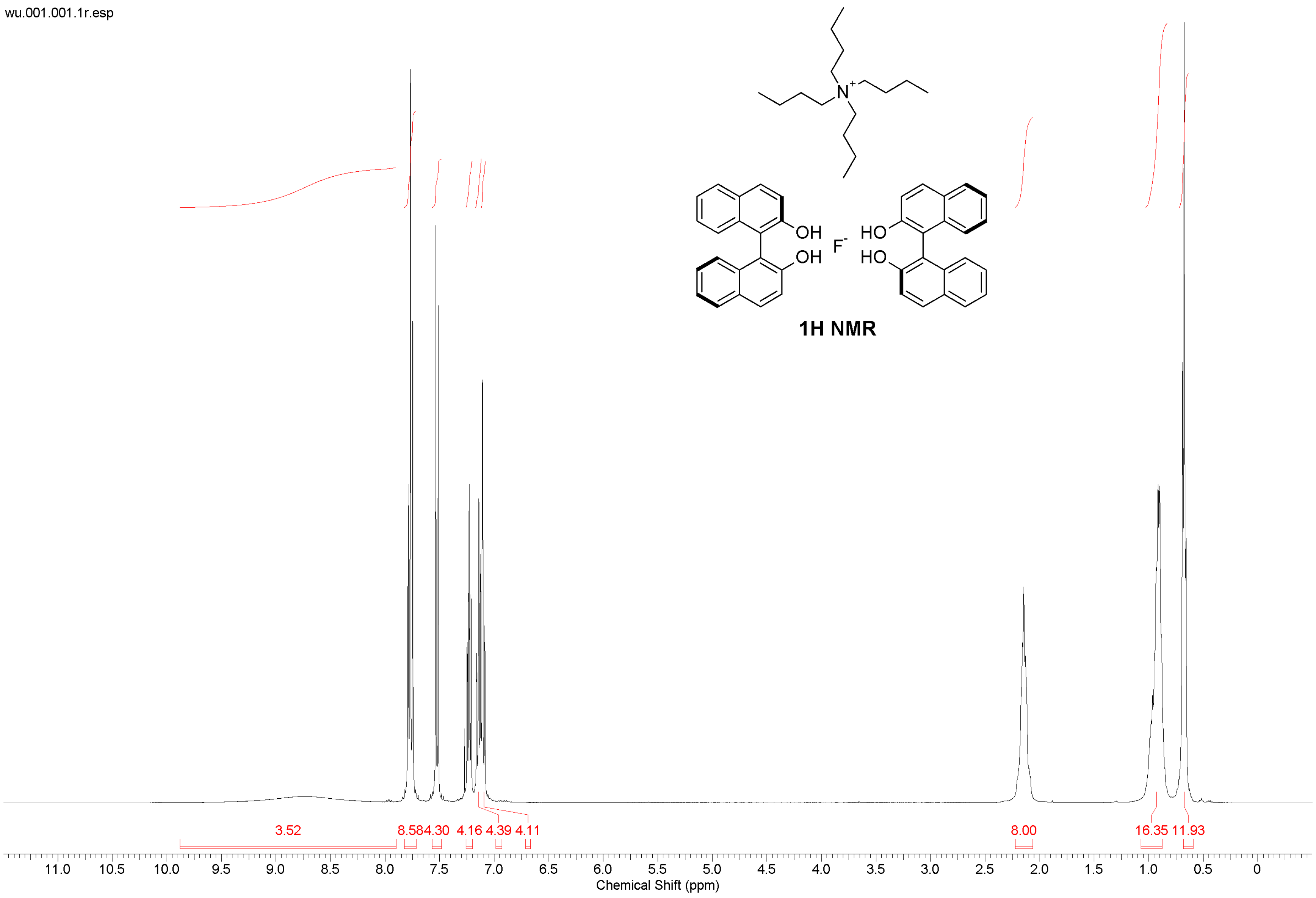
wu.001.001.1r.esp
1H NMR
OH
OH
F-
HO
HO
N+
3.52
8.58
4.30
4.16
4.39
4.11
8.00
16.35
11.93
11.0 10.5 10.0 9.5 9.0 8.5 8.0 7.5 7.0 6.5 6.0 5.5 5.0 4.5 4.0 3.5 3.0 2.5 2.0 1.5 1.0 0.5 0
Chemical Shift (ppm)

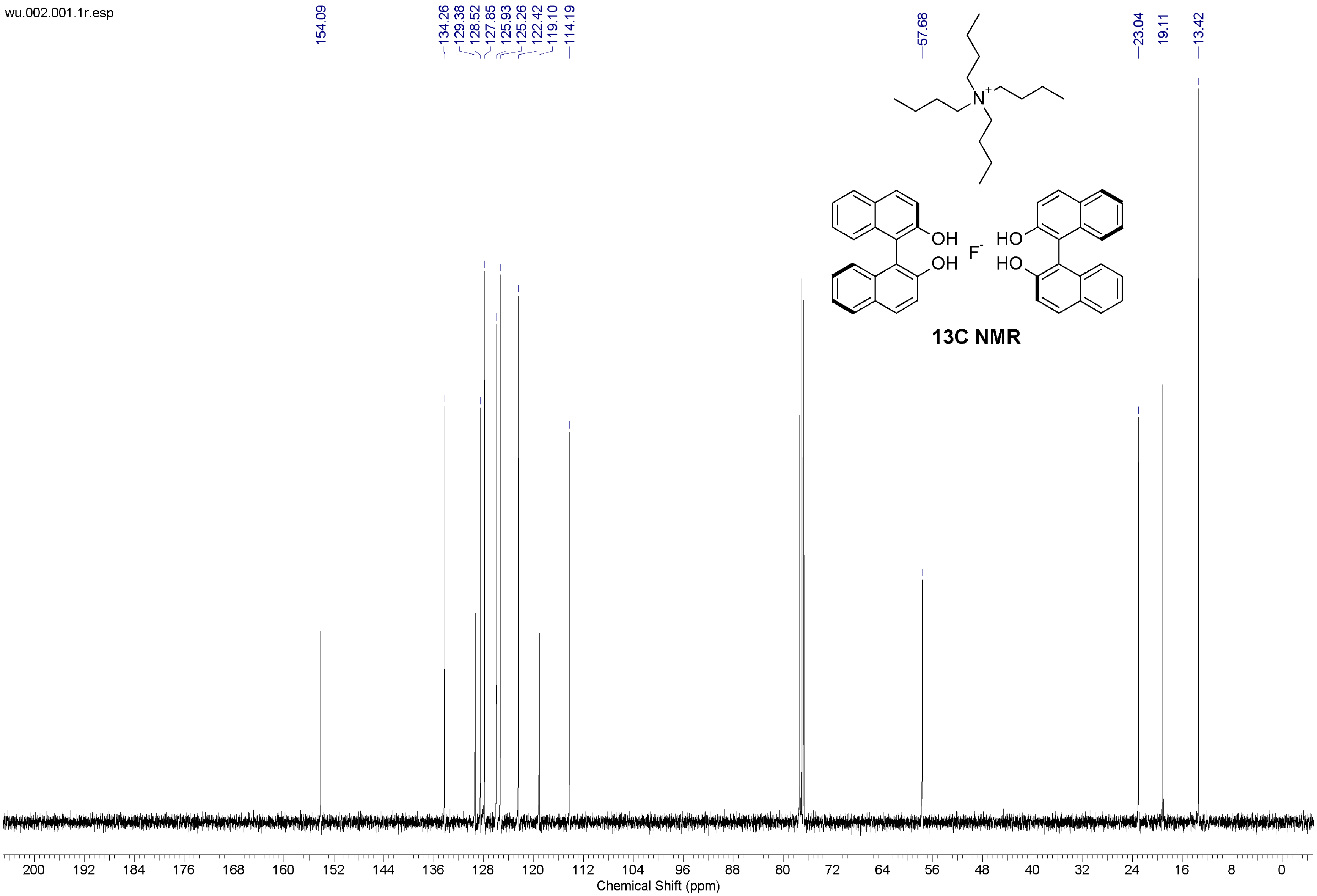

wu.002.001.1r.esp
154.09
134.26
129.38
128.52
127.85
125.93
125.26
122.42
119.10
114.19
57.68
23.04
19.11
13.42
OH
OH
F-
HO
HO
N+
13C NMR
200 192 184 176 168 160 152 144 136 128 120 112 104 96 88 80 72 64 56 48 40 32 24 16 8 0
Chemical Shift (ppm)

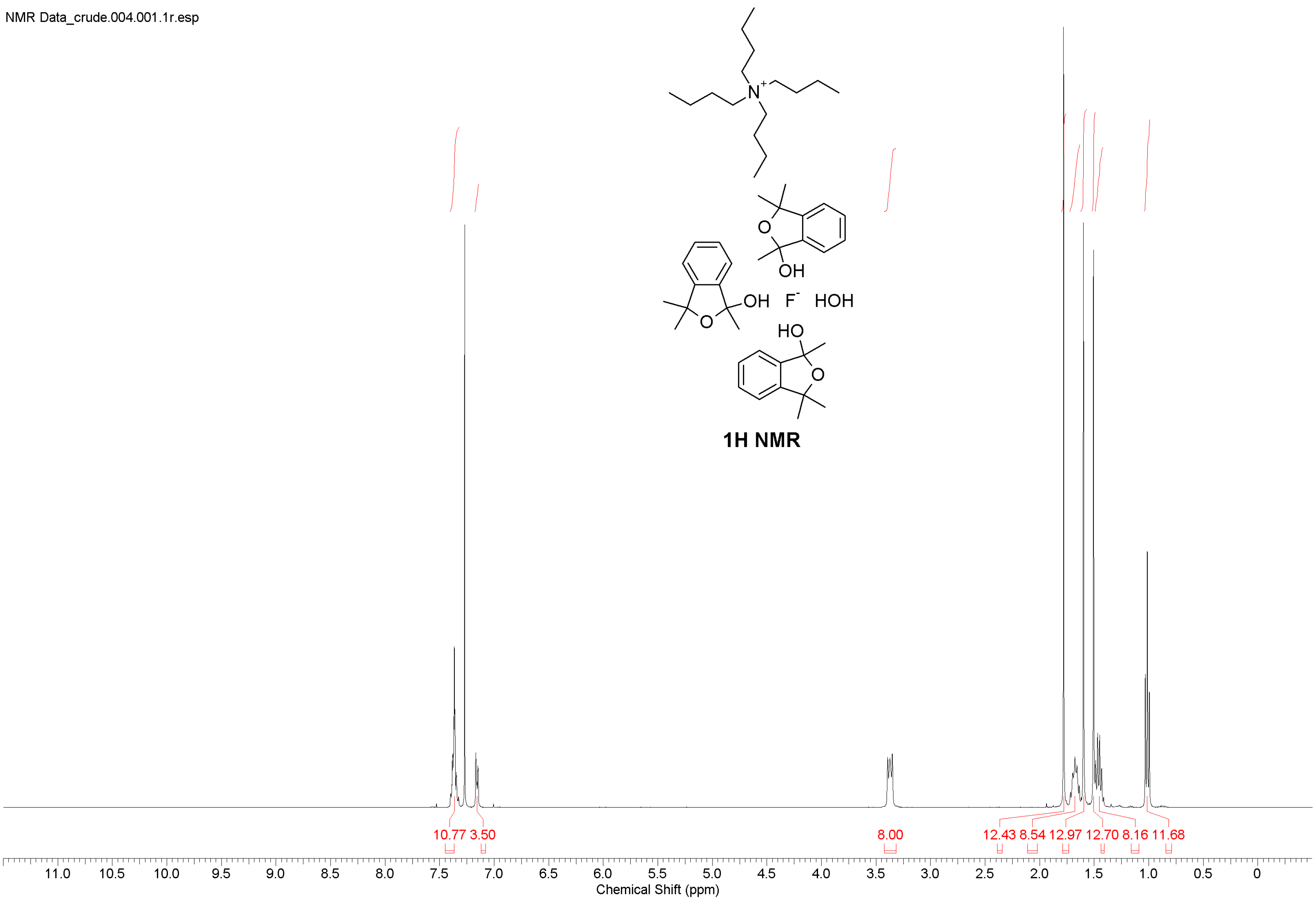

NMR Data_crude.004.001.1r.esp
N+
O
OH
OH
F-
HOH
HO
O
1H NMR
10.77
3.50
8.00
12.43
8.54
12.97
12.70
8.16
11.68
11.0 10.5 10.0 9.5 9.0 8.5 8.0 7.5 7.0 6.5 6.0 5.5 5.0 4.5 4.0 3.5 3.0 2.5 2.0 1.5 1.0 0.5 0
Chemical Shift (ppm)

NMR Data_hex wash.002.001.1r.esp

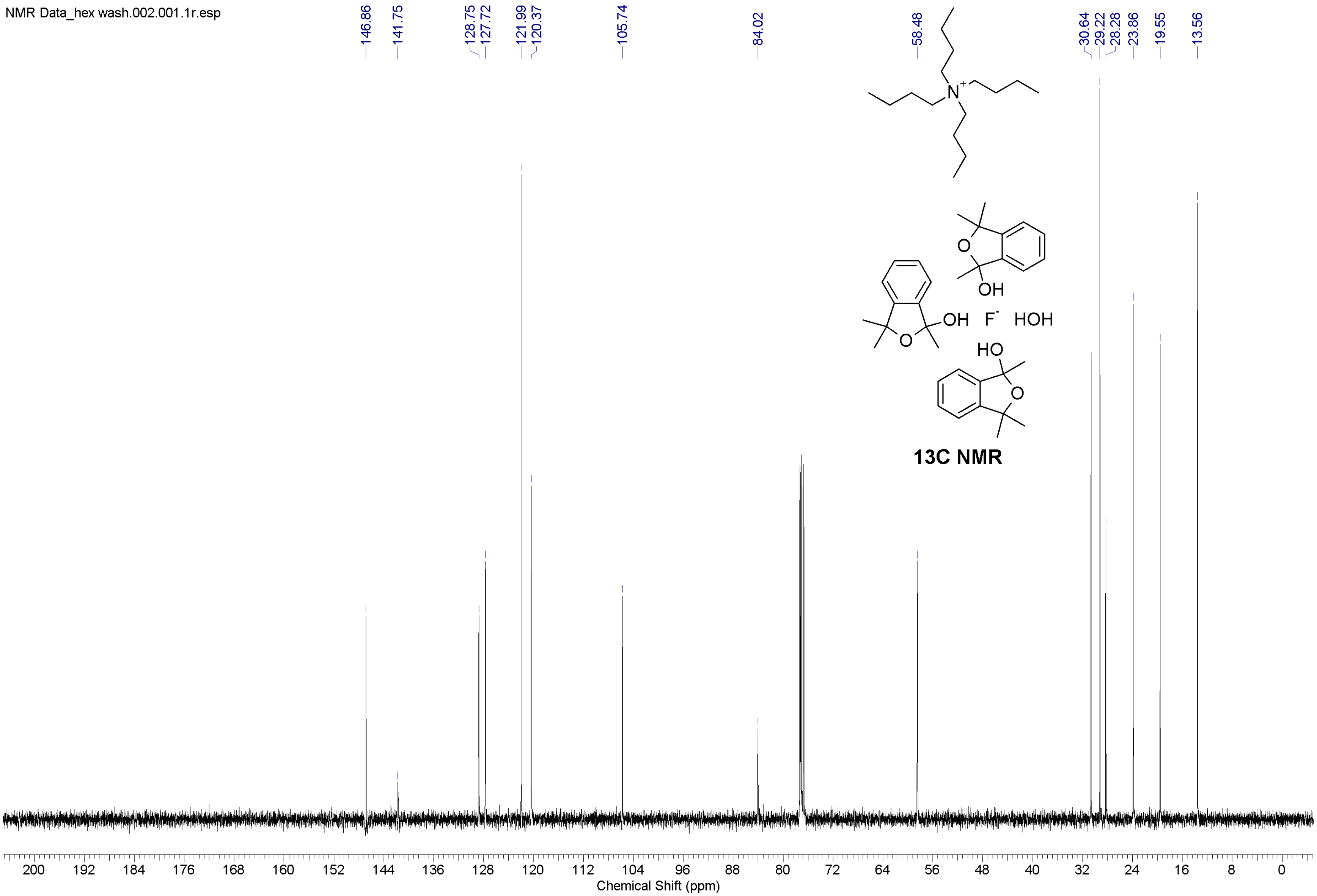

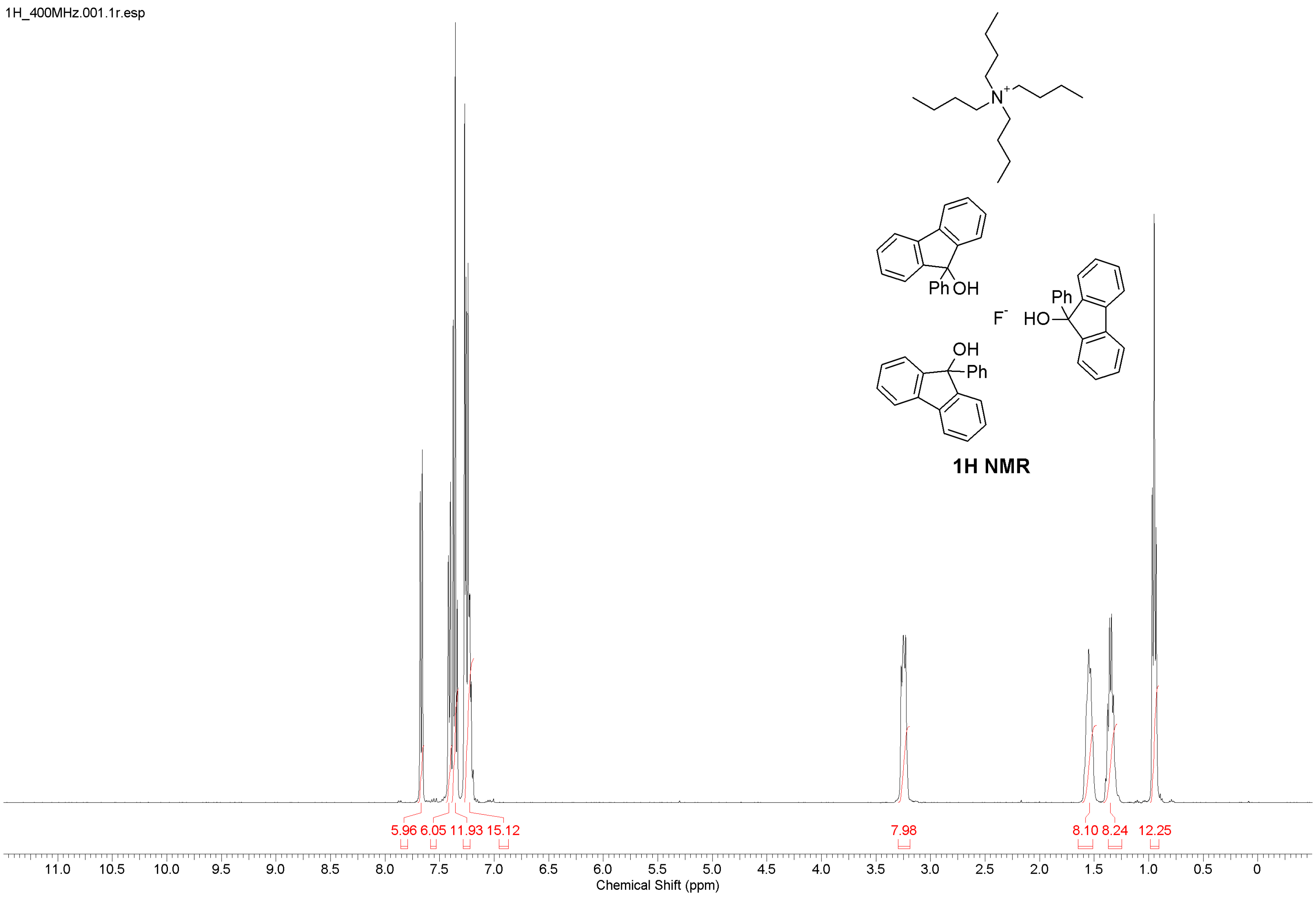

1H_400MHz.001.1r.esp
N+
F-
Ph OH
Ph
HO
OH
Ph
1H NMR
5.96 6.05 11.93 15.12
7.98
8.10 8.24 12.25
11.0 10.5 10.0 9.5 9.0 8.5 8.0 7.5 7.0 6.5 6.0 5.5 5.0 4.5 4.0 3.5 3.0 2.5 2.0 1.5 1.0 0.5 0
Chemical Shift (ppm)

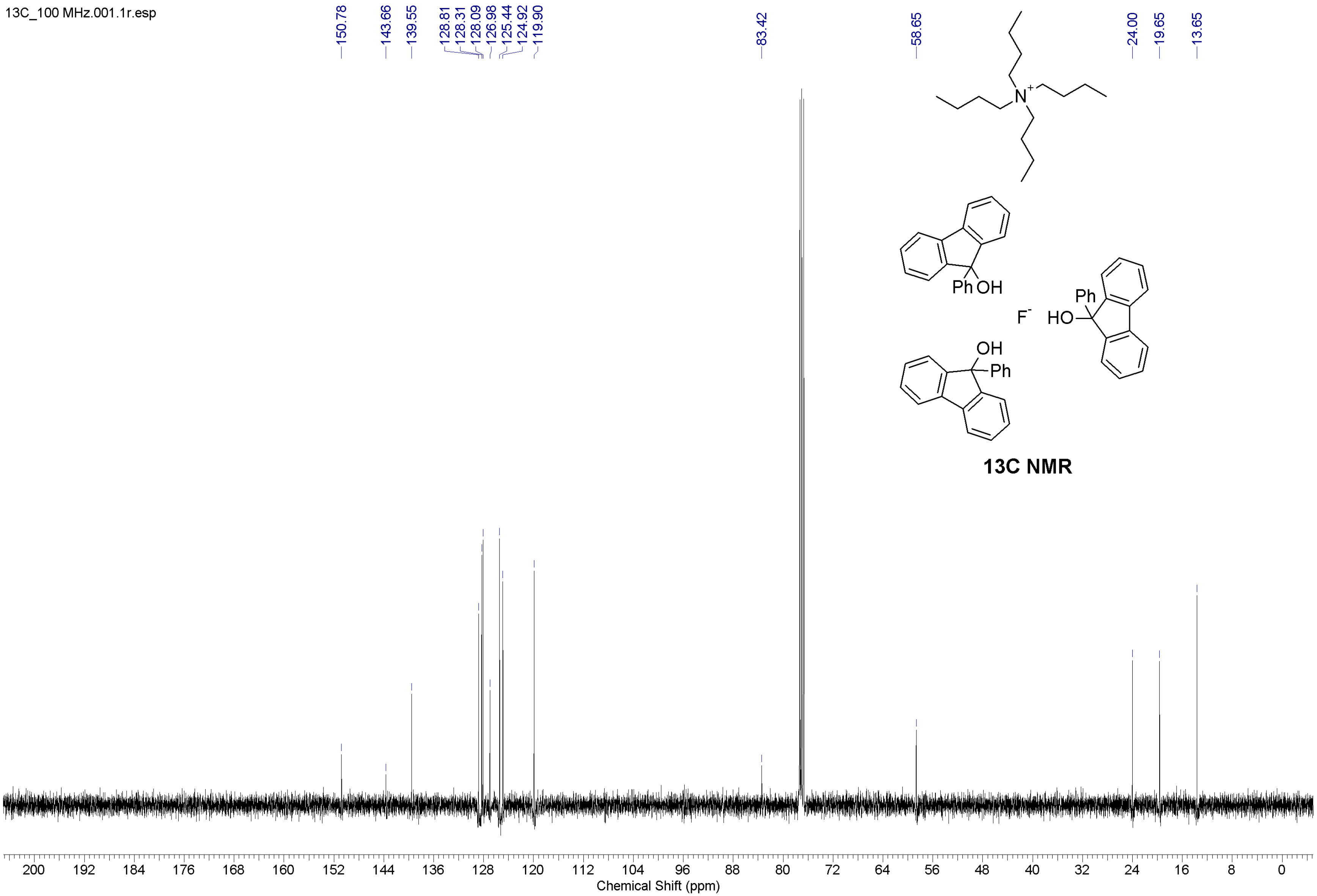
13C_100 MHz.001.1r.esp
150.78
143.66
139.55
128.81
128.31
128.09
126.98
125.44
124.92
119.90
83.42
58.65
24.00
19.65
13.65
N+
Ph OH
F-
Ph
HO
OH
Ph
13C NMR
200 192 184 176 168 160 152 144 136 128 120 112 104 96 88 80 72 64 56 48 40 32 24 16 8 0
Chemical Shift (ppm)

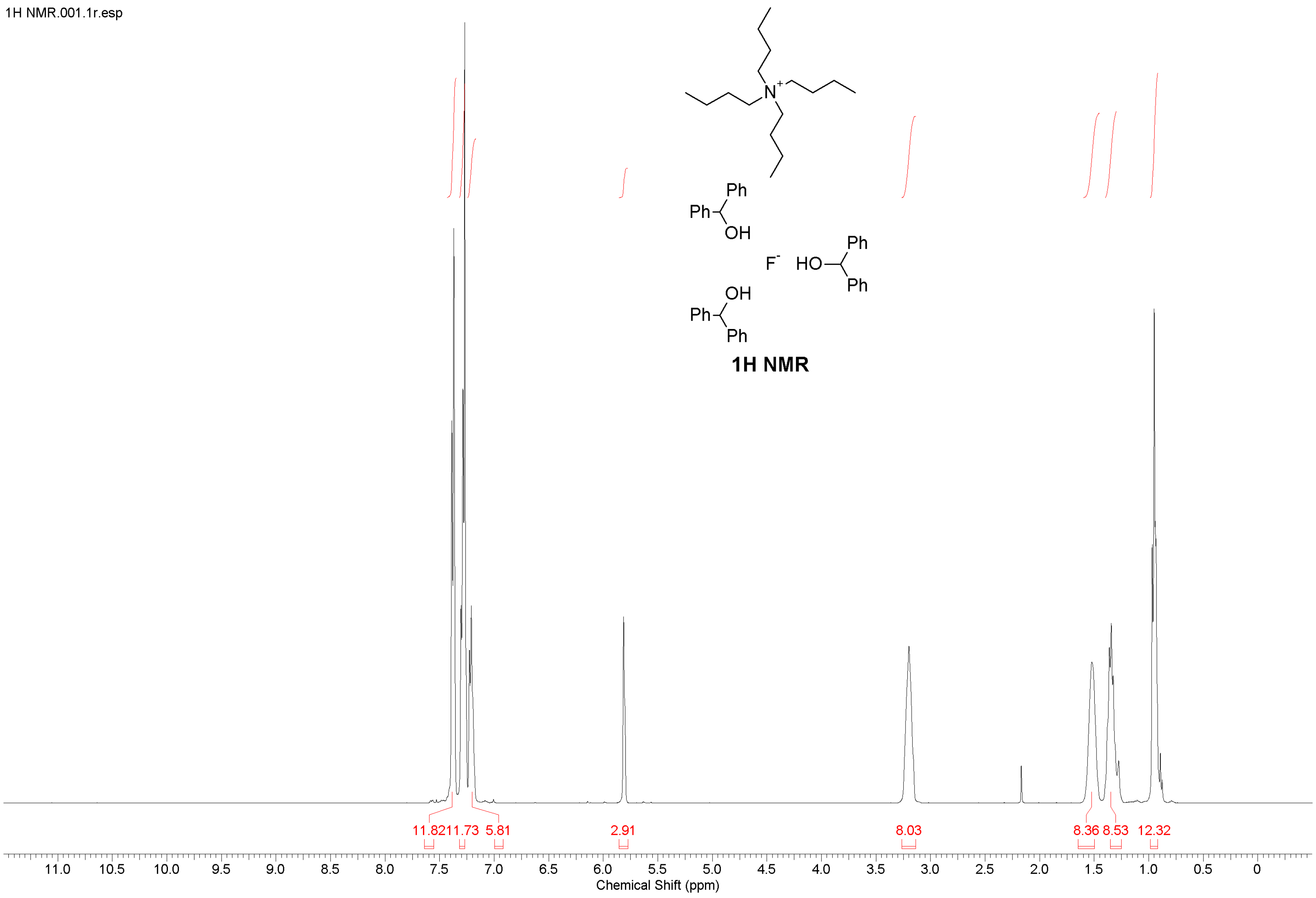

1H NMR.001.1r.esp
Ph
Ph
OH
F-
HO
Ph
Ph
OH
Ph
Ph
1H NMR
11.82
11.73
5.81
2.91
8.03
8.36
8.53
12.32
11.0
10.5
10.0
9.5
9.0
8.5
8.0
7.5
7.0
6.5
6.0
5.5
5.0
4.5
4.0
3.5
3.0
2.5
2.0
1.5
1.0
0.5
0
Chemical Shift (ppm)

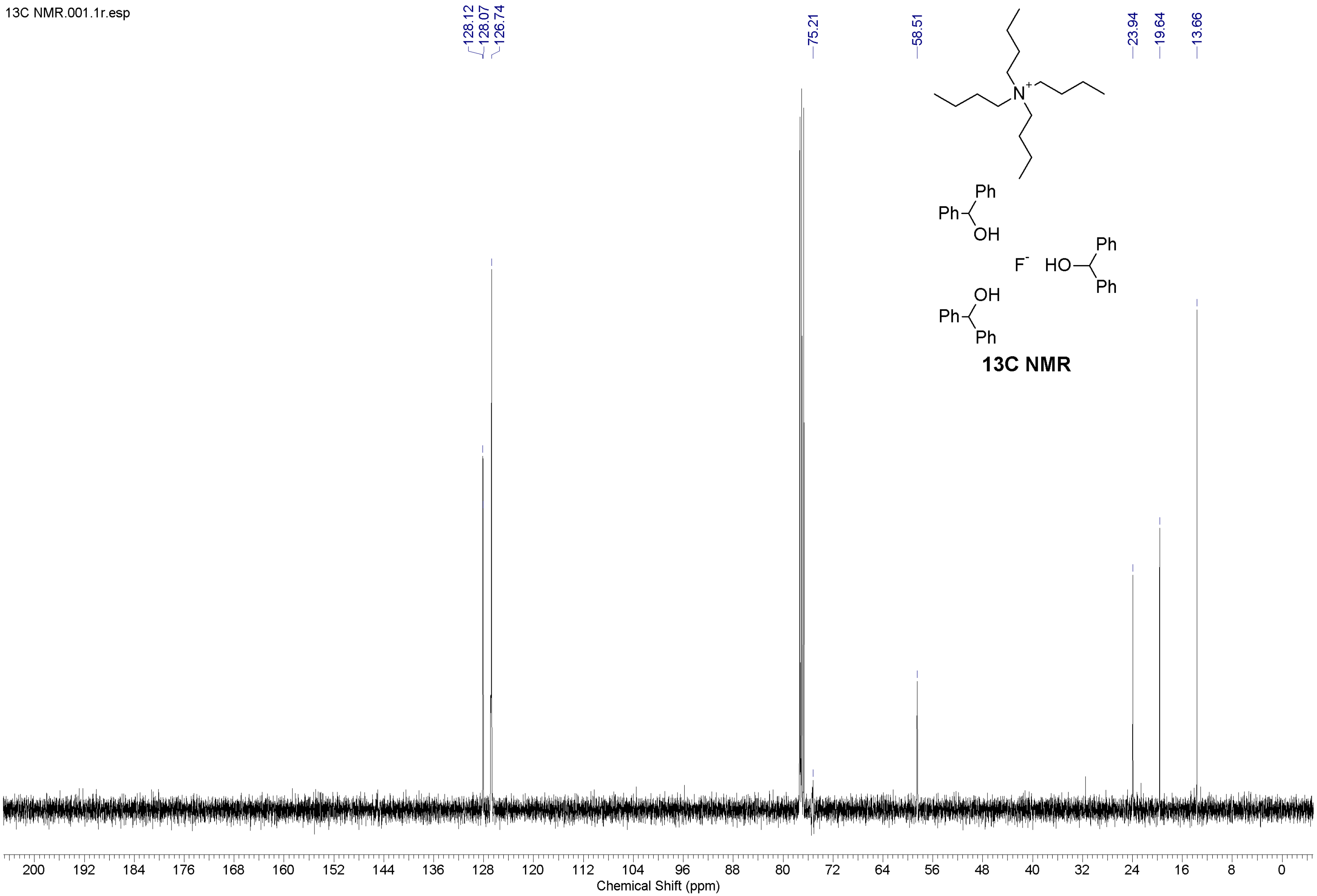
13C NMR.001.1r.esp
128.12
128.07
126.74
75.21
58.51
23.94
19.64
13.66
N+
Ph
OH
F-
HO
13C NMR
Chemical Shift (ppm)
200 192 184 176 168 160 152 144 136 128 120 112 104 96 88 80 72 64 56 48 40 32 24 16 8 0

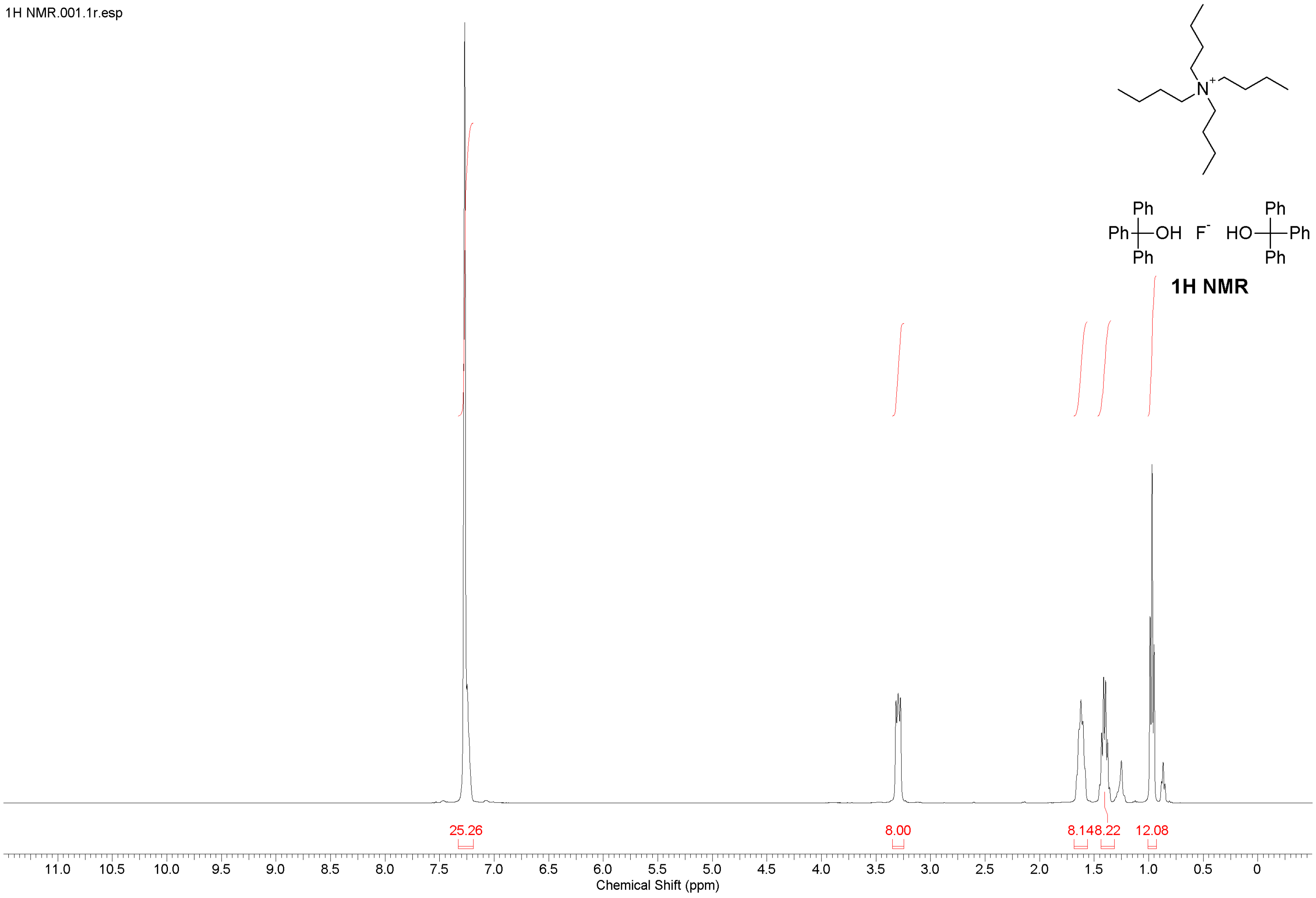
1H NMR.001.1r.esp
Ph
Ph
OH
F-
HO
Ph
Ph
Ph
Ph
1H NMR
25.26
8.00
8.14
8.22
12.08
11.0
10.5
10.0
9.5
9.0
8.5
8.0
7.5
7.0
6.5
6.0
5.5
5.0
4.5
4.0
3.5
3.0
2.5
2.0
1.5
1.0
0.5
0
Chemical Shift (ppm)

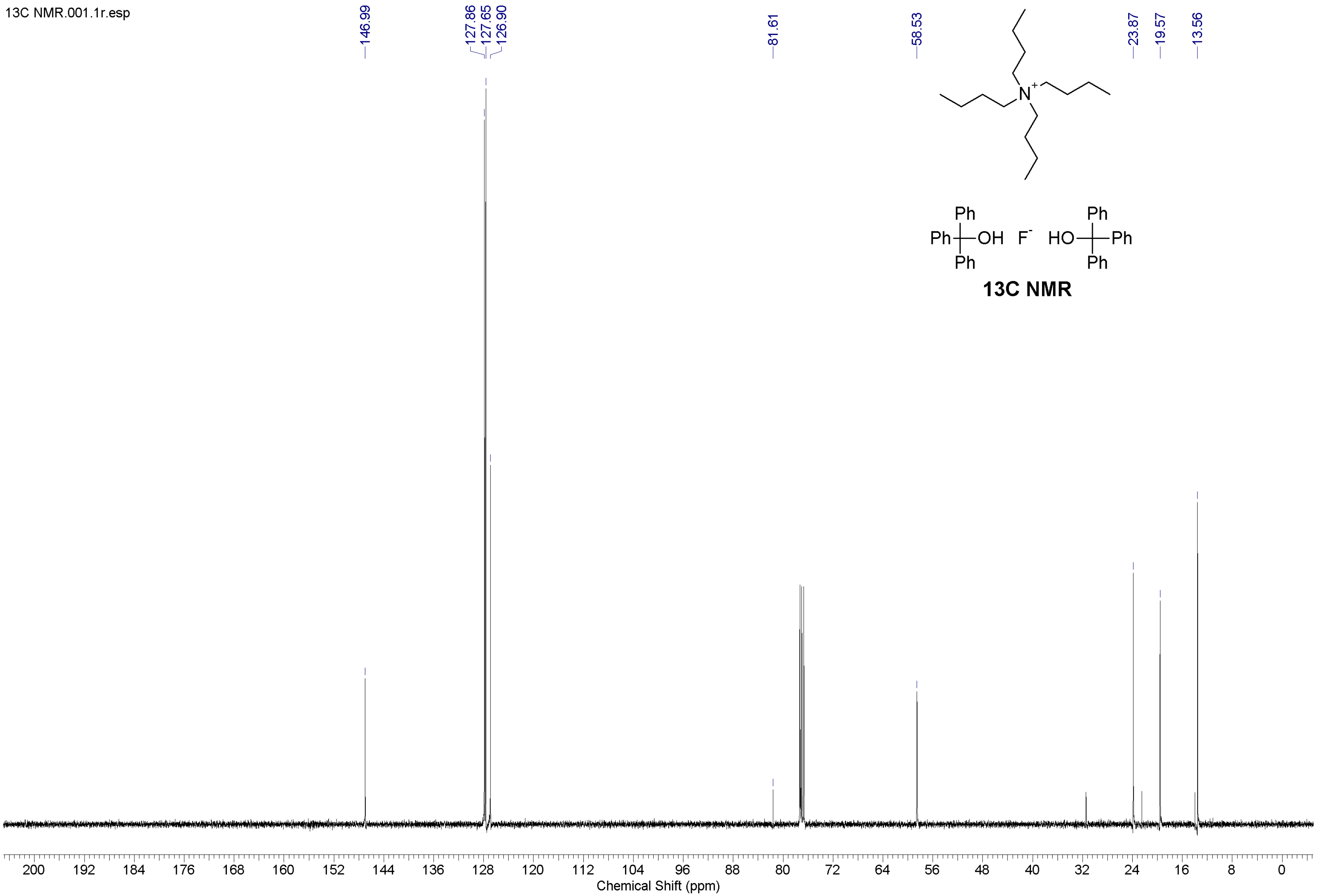
13C NMR.001.1r.esp
146.99
127.86
127.65
126.90
81.61
58.53
23.87
19.57
13.56
N+
Ph
Ph—OH F- HO—Ph
Ph
13C NMR
200 192 184 176 168 160 152 144 136 128 120 112 104 96 88 80 72 64 56 48 40 32 24 16 8 0
Chemical Shift (ppm)

1H_400 MHz.001.1r.esp

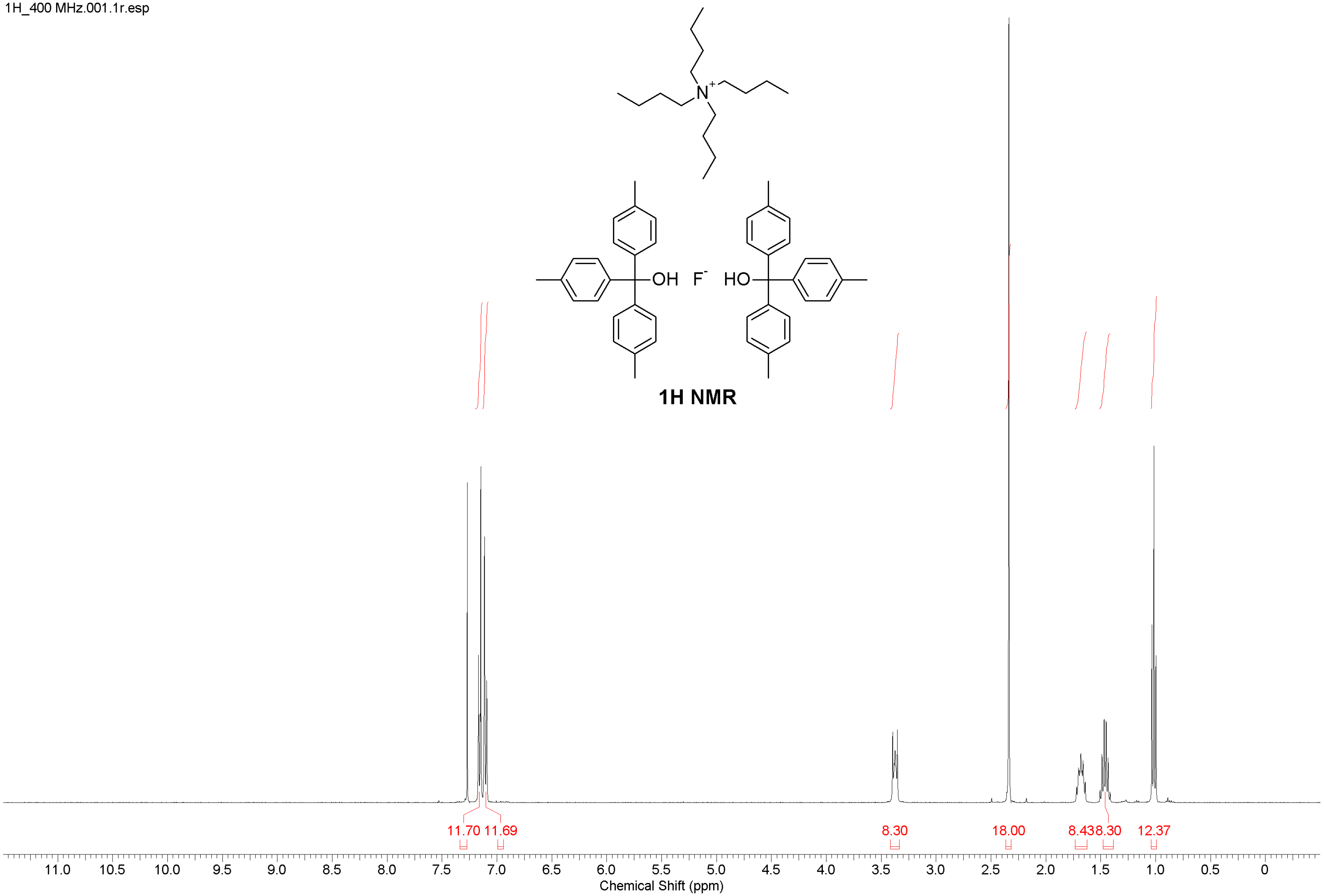

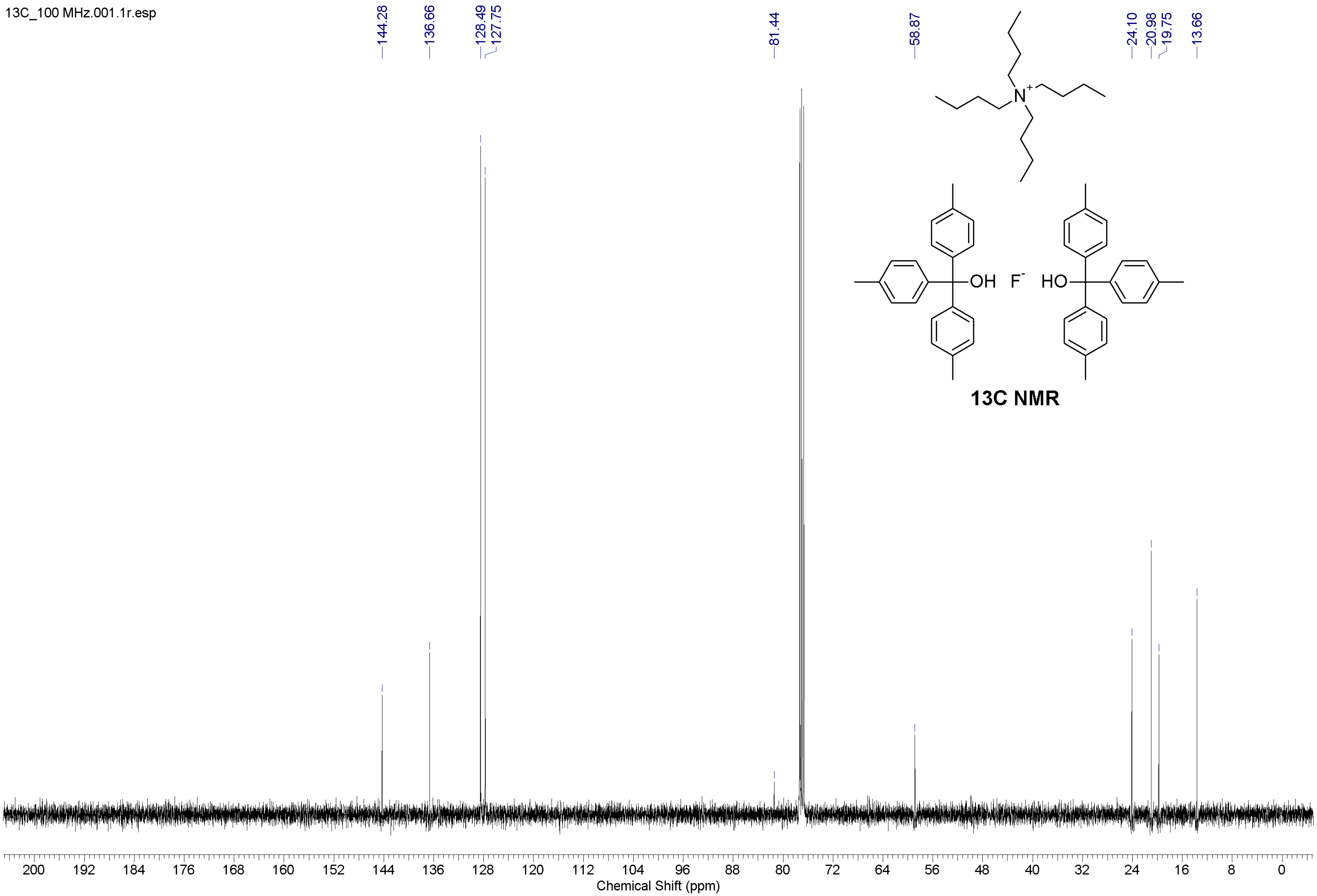

13C_100 MHz.001.1r.esp
144.28
136.66
128.49
127.75
81.44
58.87
24.10
20.98
19.75
13.66
N+
OH
F-
HO
13C NMR
200
192
184
176
168
160
152
144
136
128
120
112
104
96
88
80
72
64
56
48
40
32
24
16
8
0
Chemical Shift (ppm)

wu.001.001.1r.esp

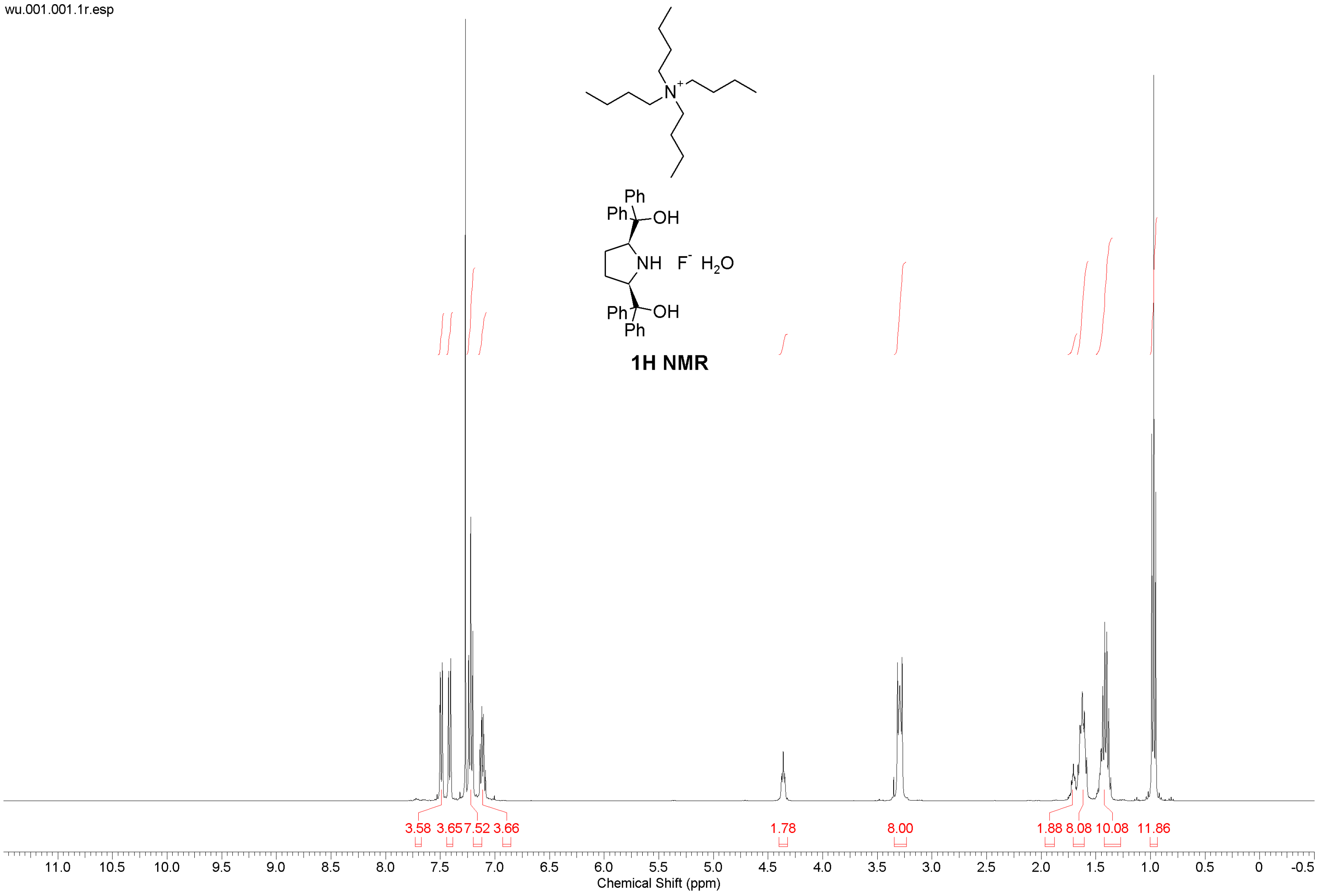

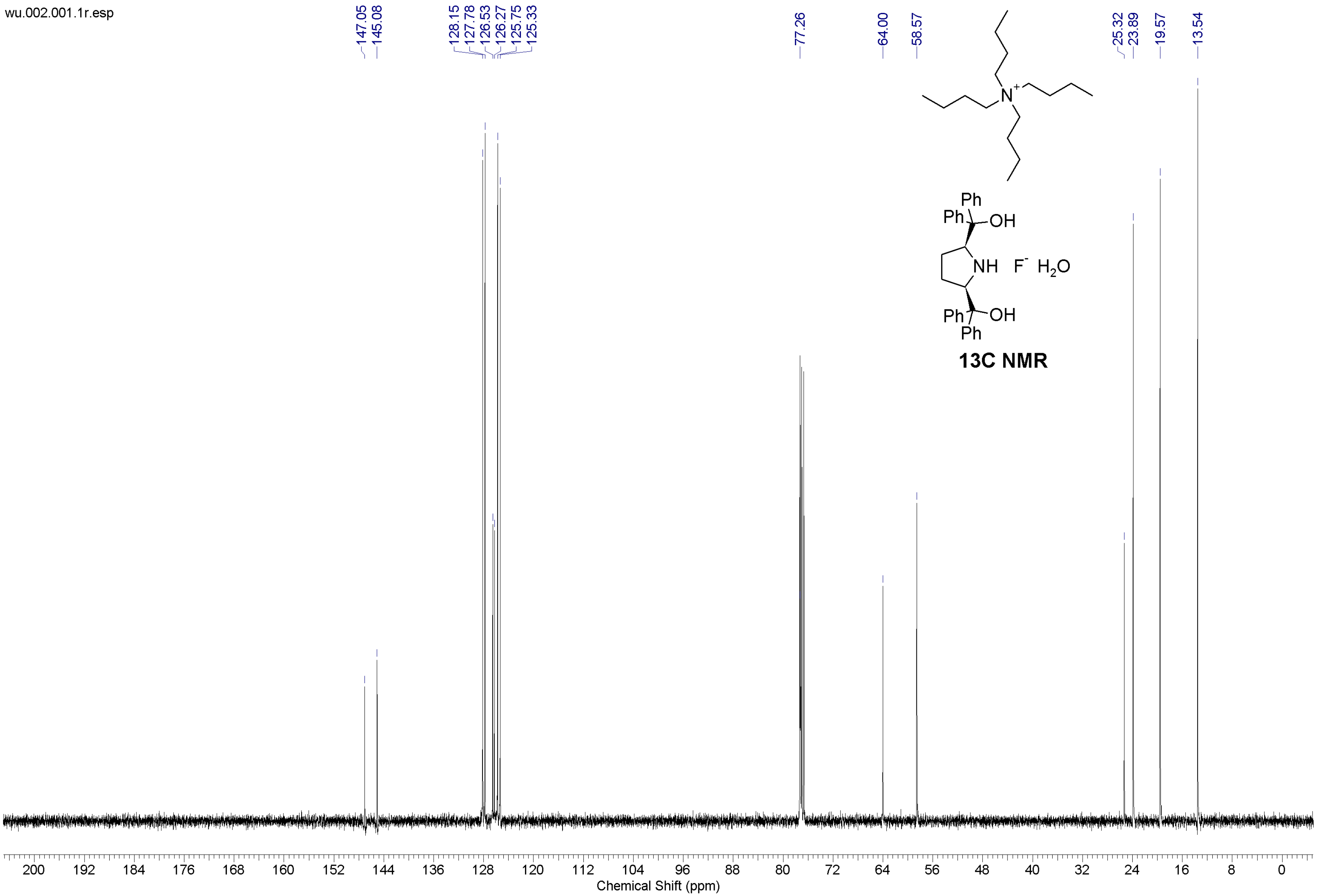
wu.002.001.1r.esp
147.05
145.08
128.15
127.78
126.53
126.27
125.75
125.33
77.26
64.00
58.57
25.32
23.89
19.57
13.54
N+
Ph
Ph
OH
NH
F-
H2O
Ph
OH
Ph
13C NMR
200 192 184 176 168 160 152 144 136 128 120 112 104 96 88 80 72 64 56 48 40 32 24 16 8 0
Chemical Shift (ppm)
